# Photoexcited Pd(ii) auxiliaries enable light-induced control in C(sp^3^)–H bond functionalisation†

**DOI:** 10.1039/c9sc05722f

**Published:** 2020-01-23

**Authors:** Milena L. Czyz, Geethika K. Weragoda, Tyra H. Horngren, Timothy U. Connell, Daniel Gomez, Richard A. J. O'Hair, Anastasios Polyzos

**Affiliations:** School of Chemistry, The University of Melbourne Parkville 3010 Victoria Australia anastasios.polyzos@unimelb.edu.au rohair@unimelb.edu.au; CSIRO Manufacturing Research Way Clayton VIC 3168 Australia; School of Science, RMIT University Melbourne Victoria 3000 Australia

## Abstract

Herein we report the photophysical and photochemical properties of palladacycle complexes derived from 8-aminoquinoline ligands, commonly used auxiliaries in C–H activation. Spectroscopic, electrochemical and computational studies reveal that visible light irradiation induces a mixed LLCT/MLCT charge transfer providing access to synthetically relevant Pd(iii)/Pd(iv) redox couples. The Pd(ii) complex undergoes photoinduced electron transfer with alkyl halides generating C(sp^3^)–H halogenation products rather than C–C bond adducts. Online photochemical ESI-MS analysis implicates participation of a mononuclear Pd(iii) species which promotes C–X bond formation *via* a distinct Pd(iii)/Pd(iv) pathway. To demonstrate the synthetic utility, we developed a general method for inert C(sp^3^)–H bond bromination, chlorination and iodination with alkyl halides. This new strategy in auxiliary-directed C–H activation provides predictable and controllable access to distinct reactivity pathways proceeding *via* Pd(iii)/Pd(iv) redox couples induced by visible light irradiation.

## Abstract 2

Visible light irradiation of 8-aminoquinoline Pd(ii) complexes initiates photoinduced electron transfer with alkyl halides, affording C–H halogenation over C–C bond adducts. A method for inert C(sp^3^)–H bond halogenation (Br, Cl and I) is reported.

## Graphical abstract

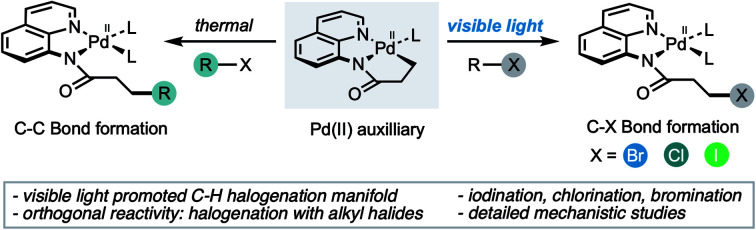

## Introduction

Advances in homogeneous palladium catalysis have identified efficient and versatile manifolds for the selective functionalisation of C–H bonds. In contrast to the direct functionalisation of C(sp^2^)–H bonds in aromatic compounds,^1^ approaches to functionalisation of isolated alkyl C–H bonds (unactivated C(sp^3^)–H bonds) remain scarce.^2^ The challenges of inert C(sp^3^)–H bond functionalisation derive from their high bond strengths, and lack of selectivity arising from the indistinguishable steric and electronic properties of proximal C–H bonds in alkanes.^2*b*,2*c*,3^ A bidentate directing group (DG) strategy has been developed to address these selectivity and thermodynamic requirements for C(sp^3^)–H bond scission.^4^ Pioneering work by Daugulis^5^ demonstrated that bidentate auxiliaries lower the energy barrier for C–H bond cleavage^6^ and result in the formation of stable cyclometallated intermediates.

The cyclometallated Pd(ii) complexes undergo concerted, two-electron oxidative addition with aryl or alkyl halides under thermal conditions to forge the new C–C bond.^7^ A limitation of this approach is the two-electron Pd(ii)/Pd(iv) redox cycle that generally imposes a high energy barrier to oxidative addition, usually mandating high temperatures, stoichiometric additives and extended reaction times.^7*h*,8^ A useful strategy to reduce this energy demand relies on the addition of a carbon-centred radical to cyclometallated Pd(ii) complexes to generate a Pd(iii) intermediate *via* a one-electron oxidative addition step.^9^ Recently, our research group reported that a Pd complex derived from a 5-chloro-8-aminoquinoline ligand undergoes single electron oxidation with an aryl radical to generate a Pd(iii) intermediate, under synergistic photoredox and palladium catalysis.^10^ The overall Pd(ii)/Pd(iii)/Pd(iv) redox chemistry reduced the thermodynamic and kinetic barriers to direct C(sp^3^)–H functionalisation, allowing selective C–H arylation to proceed at room temperature with a range of functionalised arenes.

**Fig. 1:**
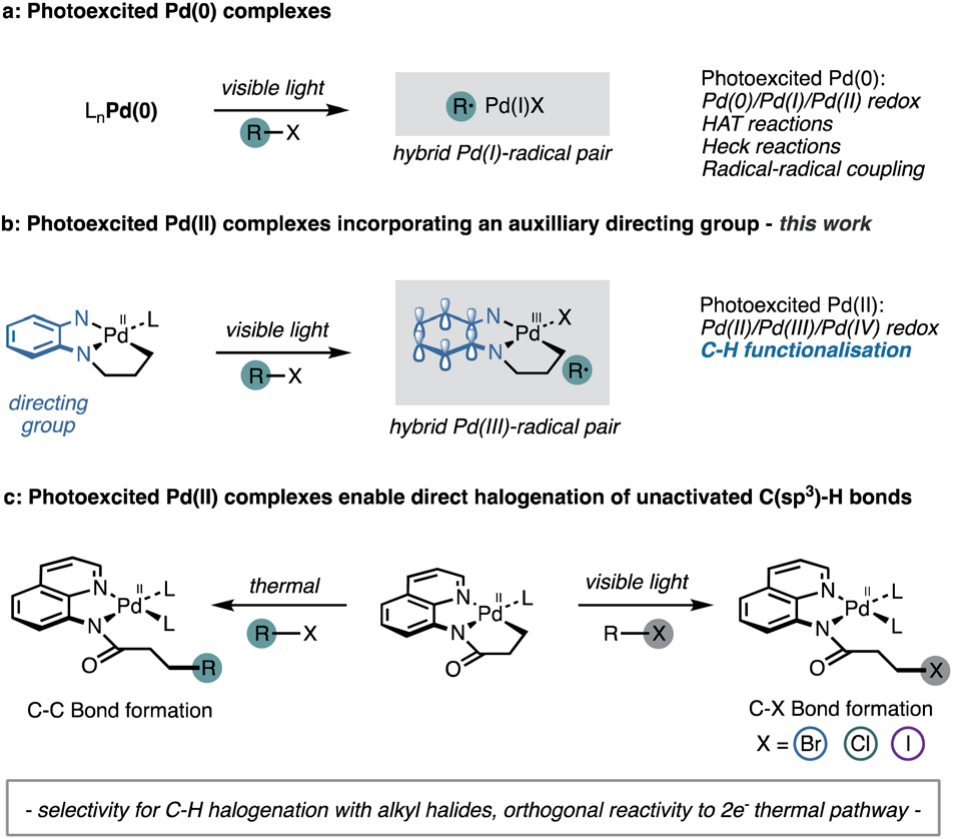
(a) Previous work. (b) Proposed hybrid Pd(iii)-radical pair. (c) Reactivity control *via* visible light irradiation.

The Gevorgyan group^11^ recently established that visible light excitation of Pd complexes induces single electron transfer (SET) processes in the absence of exogenous photosensitisers, to engage new reactivity through a Pd(0)/Pd(i) pathway (Fig. 1a).^12^ This approach also improves the efficiency of Pd-catalysed cross coupling chemistry by significantly accelerating reaction rates.^12*a*,13^ Thus, we wondered whether cyclometallated Pd(ii) complexes possess unexploited photochemical reactivity that promotes access to Pd(ii)/Pd(iii)/Pd(iv) redox couples *via* a direct excitation. We speculated that direct photoinduced one-electron oxidation of monomeric cyclometallated Pd(ii) complex, following irradiation with visible light, may provide access to hybrid Pd(iii)-radical species (Fig. 1b) following photoinduced electron transfer with an appropriate aryl or alkyl (pseudo)halide.^14^ The reactivity of this proposed hybrid Pd(iii)-radical species remains unexplored particularly within the context of C–H activation.

Herein we demonstrate that cyclometallated complexes of palladium derived from the 5-chloro-8-aminoquinoline ligand possess mixed MLCT/LLCT excited states that can participate in photoinduced electron-transfer reactions. These photoactivated palladium intermediates exhibit distinct, rather than analogous reactivity relative to the conventional and thermally induced Pd(ii)/Pd(iv) redox couples.^5,15^ Thus, these photoexcited complexes initiate electron transfer with alkyl halides leading to the halogenation of unactivated C(sp^3^)–H bonds (Fig. 1c). This unexpected reactivity enabled the development of synthetically relevant, and general reaction manifold for the direct halogenation of C–H bonds under mild reaction conditions.^16^ This offers a conceptually distinct approach towards directed C–H functionalisation, wherein the auxiliary plays a dual role of promoting selective activation of specific C–H bonds as well as absorbing visible light to allow one-electron changes to the oxidation states of the metal and ligand in the complex.

## Results and discussion

**Fig. 2:**
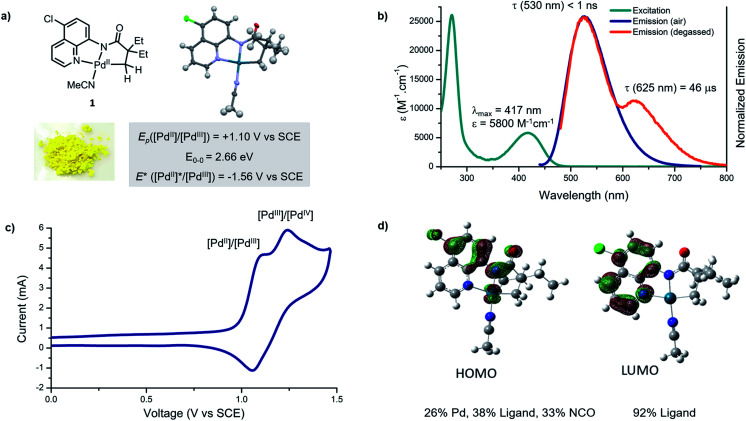
Photophysical and photochemical characteristics of palladacycle **1**. (a) X-ray crystal structure and summary of redox properties in the ground and excited states; (b) absorption and emission profiles in acetonitrile (20 μM); (c) cyclic voltammogram of palladacycle **1** (1 μM) measured in degassed acetonitrile, reported relative to SCE using Fe^+/0^ couple as an internal standard; (d) ground state frontier molecular orbitals for the palladacycle **1** (BP86/def2-TZVP).

Studies commenced with an investigation of the photophysical and electrochemical properties of cyclometallated Pd(ii) complex derived from 5-chloro-8-aminoquinoline ligand. We selected this palladacycle because complex **1** is readily isolable, highly coloured and stable to air and moisture. X-Ray diffraction analysis confirmed that the structure is monomeric, and the metal centre has a slightly distorted square planar geometry (Fig. 2a), consistent with related Pd(ii) complexes.^5*b*^

The ultraviolet-visible absorbance spectrum of palladacycle **1** in acetonitrile (Fig. 2b) reveals two absorption features; a sharp band in the ultraviolet (*λ*_max_ = 270 nm), and a broader band in the visible region (*λ*_max_ = 418 nm). Based on comparison with the free ligand and other cyclometallated Pd complexes reported in the literature,^17^ the high energy feature likely arises from ligand-based, metal-perturbed π–π* transitions. The visible feature is assigned to mixed ligand–ligand and metal-to-ligand charge transfers, that typically give bands of medium intensity (10^2^ to 10^4^ M^−1^ cm^−1^) and have been observed in planar Pd(ii) complexes featuring strong σ donors and low-lying, π-extended conjugated ligands.^17*b*^

Palladacycle **1** in acetonitrile is emissive at room temperature and displays a single luminescent band (*λ*_max_ = 530 nm) under air (Fig. 1b). A second, less-intense and overlapping emission band (*λ*_max_ = 630 nm) was observed after removal of oxygen. The excited state lifetimes of these two features are <1 ns and 46 μs, respectively. The long, triplet based emission lifetime results from the diminished spin orbit coupling of Pd relative to other metals such as Pt(II) and Ir(iii), and has been observed for Pd(ii) complexes with rigid metal chelating ring systems.^17*c*,17*d*^

Electrochemical methods were used to evaluate ground and excited state redox characteristics of palladacycle **1** (Fig. 1c). In acetonitrile, the complex features two closely overlapping quasi-reversible one electron oxidations at 1.10 and 1.24 V *vs.* SCE, which we attributed to Pd(ii)/Pd(iii) and Pd(iii)/Pd(iv) redox couples, respectively. The relatively high positive potential is consistent with a monomeric structure that does not benefit from Pd–Pd interactions,^18^ or polydentate ligands capable of stabilising Pd(iii) intermediates.^19^ Based on its electrochemistry and the value *E*_0–0_ = 2.66 eV (derived from the intersection of the excitation and emission profile),^20^ the singlet excited state reduction potential is estimated to be −1.56 V *vs.* SCE.

Time-dependent density functional theory (TD-DFT) calculations were performed to further probe the nature of the electronic transitions. The theoretical absorption spectra matched closely with the experimental results (Fig. S19†). The molecular orbital analysis revealed that the highest occupied molecular orbital (HOMO) receives large contributions from the π and the σ orbitals of the 8-aminoquinoline ligand (38%), and from Pd (26%) (Fig. 1d, S21 and S22†). On the other hand, the lowest unoccupied molecular orbital (LUMO) is π*-centred, indicating that the electronic transition at *ca.* 420 nm has mixed ligand-to-ligand (LLCT) and metal-to-ligand (MLCT) charge transfer character. Furthermore, the DFT computed ^1^MLCT transition in the visible manifold (420 nm, 2.81 eV) is in good agreement with the electrochemically estimated gap (2.88 eV), but is higher than the spectroscopically estimated band gap of 2.66 eV (Fig. S20†).

The reduction potential of −1.56 V *vs.* SCE suggests that the excited state palladacycle 1* should undergo exergonic electron transfer with activated alkyl iodides, such as perfluorobutyl iodide **2** (*E*_p_ = −1.42 V *vs.* SCE). Luminescence quenching experiments identified that **2** quenched both singlet and triplet emission bands of the 1* excited state (Fig. S6A†). The linear correlation of the Stern–Volmer is consistent with a purely dynamic quenching process (Fig. S6B†).^20^

The results of spectroscopic and electrochemical studies established that excitation of palladacycle **1** with visible light triggers a mixed ligand-to-ligand and metal-to-ligand charge transfer, followed by photoinitiated electron transfer with an activated alkyl iodide. To investigate the synthetic utility of this pathway, we probed the reactivity through combining palladacycle **1** with one equivalent of perfluorobutyl iodide **2** in acetonitrile and irradiating with 15 W blue LEDs at room temperature overnight (Table 1, entry 1). Unexpectedly, the initial experiment conditions generated the iodinated amide **3** in 54% isolated yield. Importantly, the iodinated product was not detected in the absence of irradiation at ambient temperature, or with heating at 65 °C (Table 1, entry 2–3). In both cases, cyclometallated complex **1** was recovered in quantitative yield, ruling out a 2-electron, S_N_2-type oxidative addition pathway. Irradiation with a wavelength beyond the absorption maximum of palladacycle **1** (530 nm, with the green LEDs) led to decreased conversion, which further supports the operation of a photon-initiated reaction (Table 1, entry 4). Furthermore, in the absence of the cyclometallated complex **1**, minimal homolysis of perfluorobutyl iodide **2** under blue light irradiation was observed by ^19^F NMR spectroscopy, suggesting that a homolytic cleavage of perfluorobutyl iodide, followed by trapping of Pd(ii) by an iodine radical, is also unlikely (Fig. S26†).^21^ Increasing perfluorobutyl iodide from one to two equivalents did not improve the yield of the reaction, providing further evidence that the halogenation reagent was not decomposing through direct photolysis, or unproductive radical chain propagation pathways. On the other hand, addition of one equivalent of oxone, increased the yield of **3** to 89% yield. Related oxidants, K_2_S_2_O_8_ and (NH_4_)_2_S_2_O_8_ were not effective (Table 1, entries 8–9), and the use of (diacetoxyiodo)benzene, a known 2-electron oxidant for C–H functionalization chemistry, led to extensive decomposition of the starting material (Table 1, entry 10). Conversely, application of a mild, one-electron oxidant, ferrocenium hexafluorophosphate, also led to a high yield of 86% (Table 1, entry 11). Silver salts were not compatible with reaction conditions and triggered formation of silver mirror within minutes of irradiation. (Table 1, entry 12) The reaction continued to furnish high yield of iodinated product **3** in the presence of molecular oxygen, suggesting reactivity is not dependent on the generation of a triplet excited state (Table 1, entry 7). Finally, we established that iodinated product **3** could not oxidise palladacycle **1** under photochemical conditions (Fig. S27†).

**Table 1:** Optimisation and control experiments^a^^b^

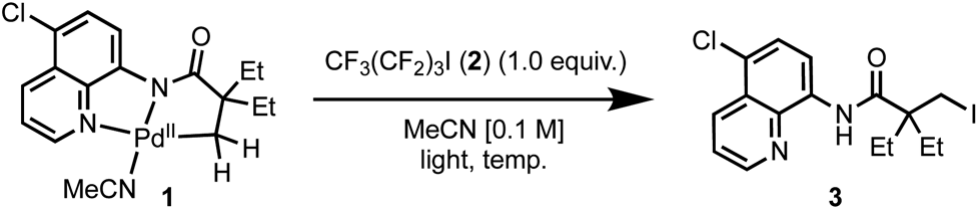				
Entry	Light	Additive (1.0 equiv.)	Temp. [°C]	Yield [%]
1	15 W blue LEDs (448 nm)	—	R.T.	55 (54)^c^
2	Dark	—	R.T.	N. D.
3	Dark	—	65	N. D.
4	15 W green LEDs (530 nm)	—	R.T.	6
5	15 W blue LEDs (448 nm)	Run with 2 equiv. of CF_3_(CF_2_)_3_I	R.T.	50
6	15 W blue LEDs (448 nm)	Oxone	R.T.	89^c^
7^d^	15 W blue LEDs (448 nm)	Oxone	R.T.	77^c^
8	15 W blue LEDs (448 nm)	K_2_S_2_O_8_	R.T.	58
9	15 W blue LEDs (448 nm)	(NH_4_)_2_S_2_O_8_	R.T.	50
10	15 W blue LEDs (448 nm)	PhI(OAc)_2_	R.T.	N. D.
11	15 W blue LEDs (448 nm)	[FeCp_2_]PF_6_	R.T.	86^c^
12	15 W blue LEDs (448 nm)	AgOAc	R.T.	33

a General conditions: palladacycle 1 (1.0 equiv.), CF_3_(CF_2_)_3_I (2) (1 equiv.), solvent (0.1 M in substrate), 16 h.

b Yield determined by ^1^H NMR spectroscopy using 2,5-dimethylfuran as an internal standard.

c Isolated yield.

d Under air.

### Further mechanistic considerations: reductive elimination

We next probed the underlying mechanism of this new C–H iodination reaction and the role of oxidants in improving the efficiency of the transformation. We posited an open-shell Pd(iii) complex as the key intermediate. Both, bimetallic Pd(iii) complexes with bridging acetate ligands and monomeric Pd(iii) complexes, have been reported to undergo reductive elimination to form carbon–heteroatom, carbon–carbon and carbon–halogen bonds.^22^ However, mononuclear Pd(iii) complexes have also been demonstrated to undergo instantaneous net disproportionation to generate Pd(iv) and Pd(ii) species, followed by reductive elimination from Pd(iv) intermediate to form a C–C or C–X bond.^22*e*,23^

**Fig. 3:**
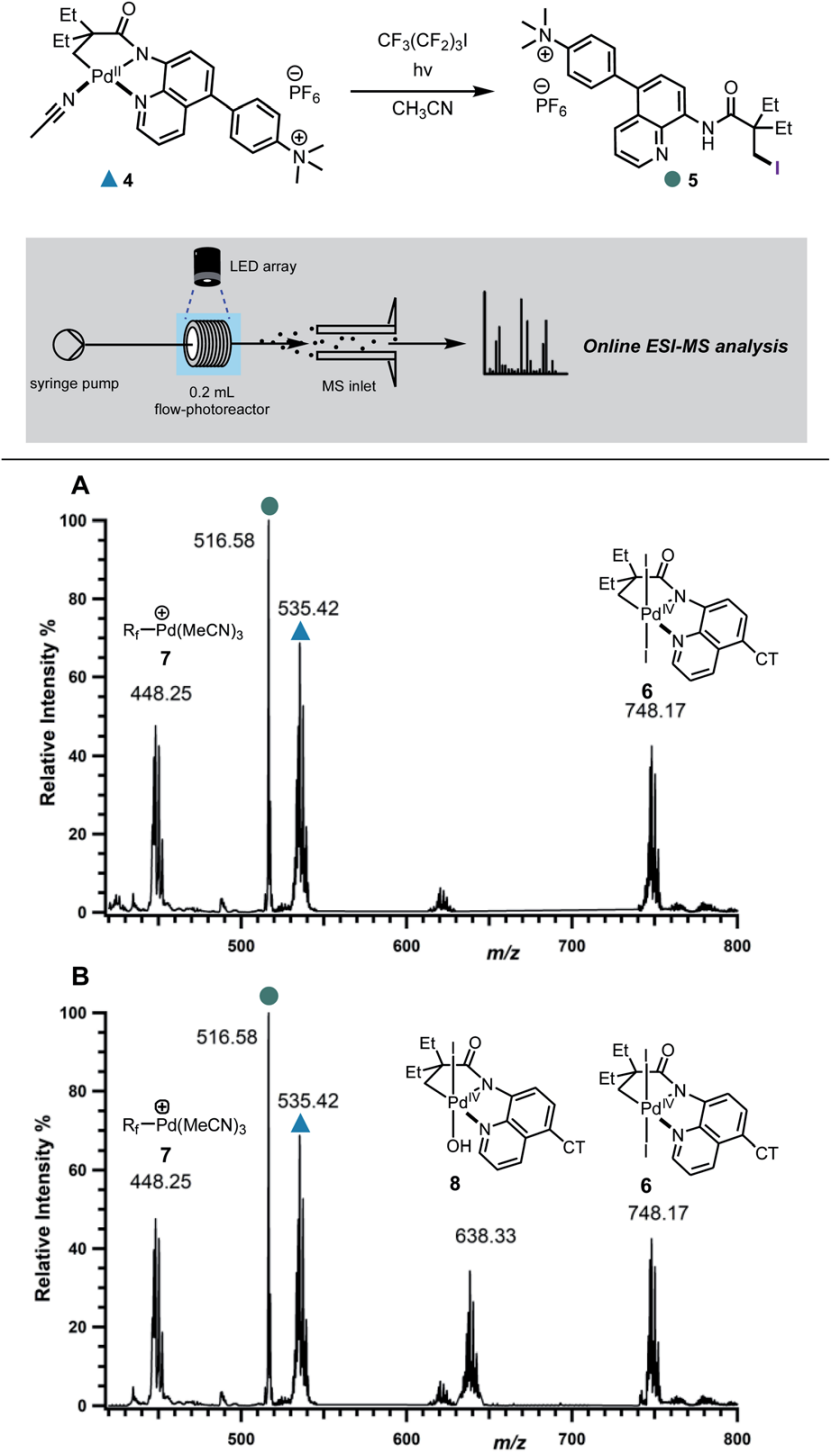
Mechanistic investigation *via* online photochemical ESI-MS analysis. Mass spectra of the reaction mixture in the: (A) absence of oxone taken at *t* = 10 min; (B) presence of oxone taken at *t* = 12 min.

In order to differentiate between these different scenarios, we turned to electrospray ionisation mass spectrometry (ESI-MS) as a means of gaining information about the key bond-forming events downstream from the initial excitation and single electron transfer events in the excited-state.^24^ A continuous flow photoreactor was interfaced to the ESI sources of two different mass spectrometers to enable real-time monitoring of the reaction and detection of key intermediates (Fig. 3 and S9†). To enable these studies, a charge-tag strategy was employed to enhance signal sensitivity in MS detection.^25^ This approach requires the installation a cationic alkylammonium group on the palladacycle, at a remote location away from the reaction site enabling signal monitoring in positive ion mode. The charge tag complex **4** was applied in the standard iodination protocol in continuous flow, which was then infused directly into the mass spectrometers. This allowed for continuous sampling of the reaction mixture over a period of 30 minutes.

Firstly, the reaction progress was analysed in the absence of oxone. Within 6 minutes of irradiation with blue LED lamp, the precursor peak for complex **4** (*m*/*z* 535) started depleting with the concurrent formation of a new peak at *m*/*z* 748, followed by those at *m*/*z* 448 and 516 (Fig. 3A, S10 and S17†). We assigned the peak at *m*/*z* 516 to the iodinated product **5**. On the other hand, the isotope pattern at *m*/*z* 748 was consistent with the formation of a new bis-iodinated palladium complex. To gain more information about its structure, collision induced dissociation (CID) was carried out. The MS/MS experiment revealed fragmentation of *m*/*z* 748 to give a new fragment at *m*/*z* 494, which corresponds to the loss of I_2_ (Fig. S12†). This is consistent with elimination of two iodide ions from a bis-iodinated Pd(iv) intermediate **6**. Importantly, the simulated HRMS pattern for **6** matched very closely with experimental results (Fig. S14†). Furthermore, plots of relative intensity *versus* time are consistent with the detected Pd(iv) complex **6** being a competent intermediate in the C–H iodination reaction (Fig. S17†).

The ion at *m*/*z* 448 was found to correspond to Pd(ii)-perfluorobutyl adduct **7**, which results from trapping of the Pd catalyst by the perfluorobutyl radical. Interestingly, products arising from trapping of the perfluorobutyl fragment with the cyclometallated adducts were not detected, suggesting that the radical might react with palladium following the reductive elimination step.^12*b*^

We next probed the role of oxone in improving the efficiency of the reaction and its involvement in the reductive elimination step. When oxone was added as an external oxidant, a new prominent peak with palladium isotope pattern was detected at *m*/*z* 638 (Fig. 3B and S11†), consistent with Pd(iv) intermediate **8**. This was further supported by the fragmentation pattern observed in the MS/MS experiment and analysis of the isotope pattern (Fig. S13 and S15†). Kinetic profiles revealed that both **8** and **6** were reactive intermediates, capable of undergoing reductive elimination to forge the new C–I bond (Fig. S18†).

Formation of Pd complex **8** in the presence of oxone can be rationalised by an inner-sphere, one-electron oxidation of Pd(iii)–I intermediate with a transfer of the hydroxyl group. It should be noted that oxone is typically considered to be a two-electron oxidant, however one electron-oxidation pathways are well-established, particularly in the presence of metals.^26^ On the other hand, the standard 2-electron reduction potential for oxone has been reported to be *E*° = 1.81 V,^27^ which prompted further investigation into the possibility of oxone interacting with Pd(ii) in the ground or excited state. Owing to a low solubility in acetonitrile, Stern–Volmer quenching experiments with oxone were deemed inconclusive; however, control experiments revealed that irradiation of complex **1** with one equivalent of oxone led to slow conversion of the starting material to hydroxylated product **9** (15% yield after 24 hours, see Table S4†). Furthermore, stirring a mixture of palladacycle, oxone and perfluorobutyl iodide at room temperature with the exclusion of light gave only 5% of the hydroxylated product **9** (Table S4†). These results suggest that ground- or excited-state oxidation of palladacycle by oxone is an unlikely mechanistic pathway for the iodination reaction.

To gain further evidence for the proposed mechanism, the reaction was analysed in the presence of ferrocenium hexafluorophosphate (Fc^+^), an unambiguous one electron, outer sphere oxidant. The new major intermediate was detected at *m*/*z* 331, consistent with one-electron oxidation of a transient Pd(iii)–I complex through an outer sphere mechanism to give cationic Pd(iv) complex **10** (Fig. S16†). This result supports our hypothesis that intermediates **6** and **8** derive from a common precursor.

**Fig. 4:**
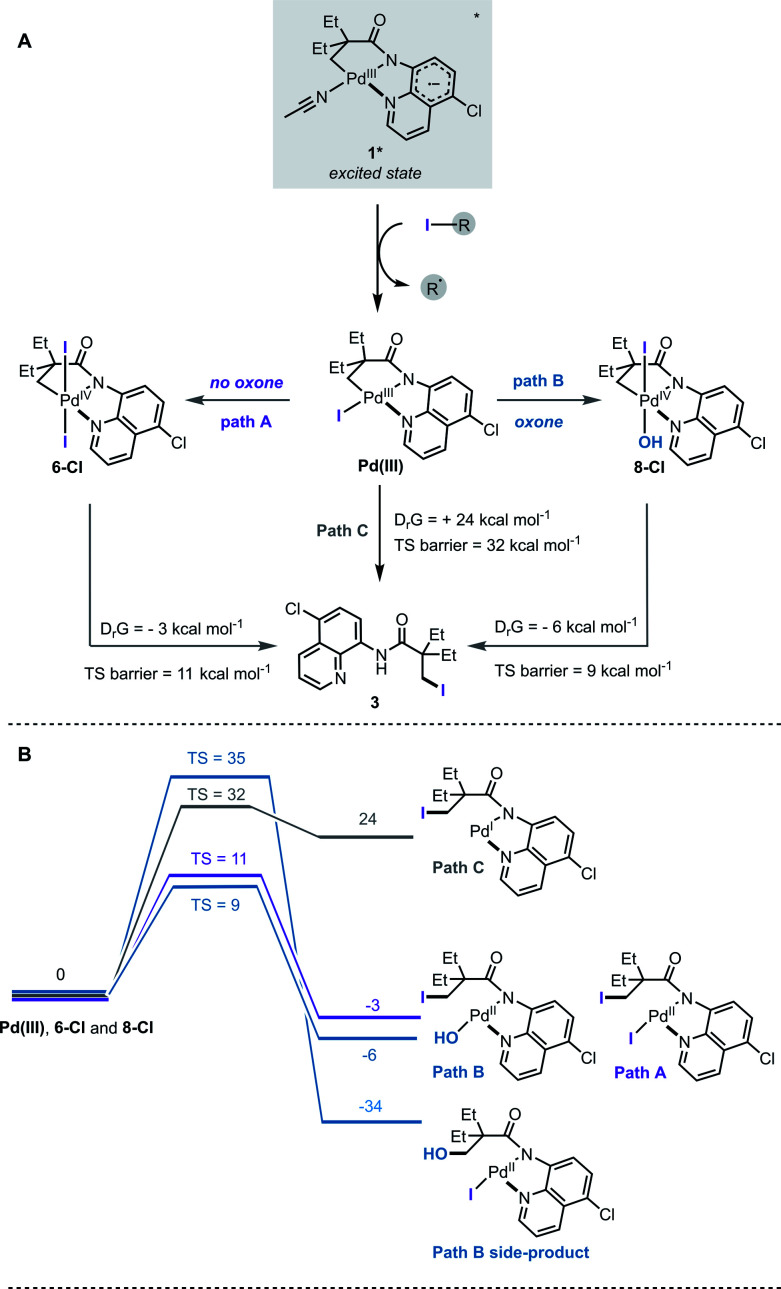
(A) Proposed mechanistic pathway in the absence of oxone (path A) and in the presence of oxone (path B), and *via* direct reductive elimination from Pd(iii) (path C); (B) DFT computed thermodynamics of reductive elimination to form C–I bond from intermediates **6-Cl**, **8-Cl** and from Pd(iii). All values in kcal mol^−1^.

Based on the ESI-MS data, the following mechanism for the halogenation reaction was proposed (Fig. 4A). Complex **1** absorbs light in the visible region and undergoes mixed LLCT/MLCT transitions to give a highly reducing (−1.56 V *vs.* SCE) singlet excited state, 1*. Single electron transfer to an alkyl halide generates a transient Pd(iii) intermediate and a perfluroalkyl radical. In path A, Pd(iii) undergoes net disproportionation to generate Pd(iv) complex **6-Cl**. For related Pd(iii) complexes, disproportionation has been reported to proceed either through a homolytic cleavage of Pd(iii)–ligand bond, or by direct ligand transfer between coordinatively unsaturated Pd(iii) complexes.^22*e*^ In path B, Pd(iii) is oxidised by an added oxidant to generate Pd(iv) complex **8-Cl**. Computational studies confirmed that solvent-assisted, concerted reductive elimination from both **6-Cl** and **8-Cl** are thermodynamically feasible, and forge the new C–I bond (Fig. 4B, S23 and S24†). Our DFT calculations also suggest that concerted C–OH reductive elimination from complex **8-Cl** has a higher energy barrier (TS barrier = 35 kcal mol^−1^) than C–I reductive elimination (TS barrier = 9 kcal mol^−1^), which agrees with the observed C–H iodination selectivity under both reaction conditions (Fig. 4B).

Observation of the Pd(iv) intermediates **6** and **8** by ESI-MS is also consistent with a relatively high energy barrier (TS = 32 kcal mol^−1^) for the reductive elimination from the Pd(iii)–I species (path C); however we cannot exclude that all three pathways proceed simultaneously under the reaction conditions.

### Reaction scope

Following investigation of the iodination mechanism, we examined the suitability of the cyclopalladated complex as a general platform for the halogenation of C(sp^3^)–H bonds. We hypothesised that a light-activated C–H activation pathway may provide a general route to direct halogenation (iodination, bromination and chlorination) of unactivated C(sp^3^)–H bonds, rather than direct alkylation that has been reported under thermal conditions.^5,7*i*,15^ Oxone was chosen as the co-oxidant for all of the reactions owing to the intense absorption of ferrocenium hexafluorophosphate in the visible region.

**Fig. 5:**
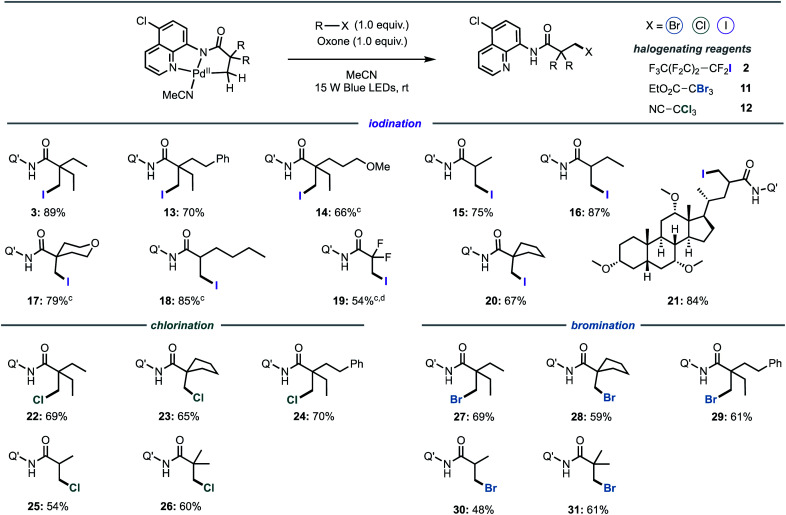
Light-promoted iodination, bromination and chlorination of aliphatic C–H bonds^a,b^. ^a^General conditions: R–X (1.0 equiv.) MeCN (0.1 M in palladacycle), r.t., 16 h, 15 W Blue LEDs (448 nm). ^b^Isolated yields. Q′ = 5-chloroquinoline. ^c^Palladacycle prepared *in situ* from the corresponding amide and Pd(OAc)_2_. ^d^Reaction run with 20 μL of DMF to improve solubility.

Firstly, we synthesised a series of palladacycles by stirring the corresponding aliphatic amides with Pd(OAc)_2_ in acetonitrile at room temperature. Pleasingly, structural changes to the carboxylic acid moiety did not affect photoreactivity of the corresponding palladacycles (Fig. 5). The reaction was found to be highly selective for the β-methyl C(sp^3^)–H bonds for all substrates containing both β-methyl and β-methylene C(sp^3^)–H bonds, with halogenation of the β-methyl group exclusively observed (Fig. 5, products **3**, **13–14**, **16**, **18**). Longer alkyl chains (Fig. 5, products **13**, **18**), ether groups (Fig. 5, products **14**, **17**), halogens (Fig. 5, product **19**), phenyl rings (Fig. 5, products **13**) and saturated carbocycles (Fig. 5, product **20**) and heterocycles (Fig. 5, product **17**) were well tolerated. Although β-halogenation of α-halogenated substrates has been reported to be exceptionally challenging,^16*e*^ α-difluoro compound was successfully iodinated under our reaction conditions to give **19** in a good yield.

Substrates bearing α-tertiary carbon centres are often incompatible with direct C–H halogenation due to competing α-halogenation of the more acidic proton, or elimination of the β-halogenated product.^16*e*^ Pleasingly, these substrates were well tolerated furnishing the desired products in excellent yields (Fig. 5, **15–16** and **18**). Competing side products were not evidenced by ^1^H-NMR spectroscopic analysis of reaction mixtures. Furthermore, it should be highlighted that most palladacycles derived from amides bearing α-tertiary carbon centres were not sufficiently stable to isolate, however these products could be prepared *in situ* from the corresponding amide and Pd(OAc)_2_ (see ESI for Experimental details†), suggesting that palladacycle stability does not exert a significant influence on the photophysical properties. Finally, we investigated the potential of the newly developed method for late-stage functionalisation of a complex, biologically active molecule and a cholic acid derivative was iodinated in an excellent 84% yield, and without epimerisation of the α-C–H bond (Fig. 5, product **21**).

We then turned our attention to the development of bromination and chlorination protocols. A series of activated alkyl bromides and chlorides were examined under optimised conditions. It was found that ethyl tribromoacetate **11** (*E*_p_ = −0.78 V *vs.* SCE) and trichloroacetonitrile **12** (*E*_p_ ≅ −1.0 V *vs.* SCE) were the most efficient brominating and chlorinating reagents, respectively. Both bromination and chlorination reactions were compatible across a range of different substrates and functional groups (Fig. 5). Furthermore, the reaction showed excellent levels of selectivity for mono-halogenated products **26** and **31**, with only traces of bis- and tris-halogenated products detected in the purified sample (see ESI for details†).

To further demonstrate synthetic utility of this methodology, we investigate possible methods for the selective removal of the auxiliary in the presence of the C–I bonds. Rao and co-workers reported that the 5-chloro-8-aminoquinoline directing group can be readily cleaved in the presence of C(sp^3^)–I bonds using BF_3_·Et_2_O or H_2_SO_4_ in methanol.^16*a*^ Indeed, exposing iodinated product **37** to acidic conditions cleanly produced deprotected ester **37a** with no evidence for the elimination or substitution of the alkyl iodide (See ESI for Experimental details†).

### Catalysis

**Scheme 1:**
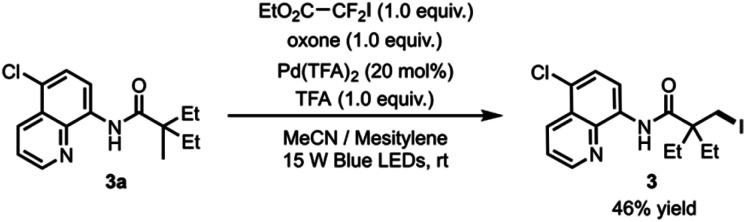
Catalytic iodination.

The newly discovered reactivity of the photoexcited palladacycle and elucidation of the iodination reaction mechanism paves the way for the development of C–H halogenation reaction that operates under catalytic conditions with respect to palladium. Formation of complex **7**, which results from trapping of the perfluorobutyl radical by the palladium catalyst (see Fig. 3), suggested that the catalyst might be deactivated following reductive elimination. Consistent with this hypothesis, our first attempt towards a catalytic reaction gave the desired iodinated product **3** in 20% yield when amide **3a** with 20 mol% Pd(TFA)_2_, perfluorobutyl iodide, oxone and trifluoroacetic acid in acetonitrile were irradiated with blue light. On the other hand, the use of ethyl difluoroiodoacetate as the iodination reagent provided indication of catalyst turnover, producing the desired product **3** in 30% yield under the same reaction conditions. Furthermore, the addition of mesitylene as a radical trap increased reaction yield to 46% (Scheme 1).

These results suggest that the development of an efficient catalytic process is possible, if the radical by-product is effectively deactivated. Our current efforts are focussed on the design of a family of halogenation reagents compatible with the catalytic reaction conditions.

## Conclusion

Here we established that cyclometallated Pd complexes derived from 5-chloro-8-aminoquinoline auxiliary, which are key intermediates for the functionalization of C(sp^3^)–H bonds under thermal conditions, also possess highly reactive photoexcited states that can be easily and predictably accessed *via* irradiation with blue light. From the excited state, the palladium complexes can participate in single electron transfer processes with organic substrates, providing access to highly reactive Pd(ii)/Pd(iii)/Pd(iv) redox couples. We have also established that Pd turnover is viable under light-induced conditions. These findings uncover a conceptually distinct and unconventional mechanistic paradigm for C–H activation chemistry, which should allow the design of a range of broadly useful C–H functionalisation reactions. Work is presently underway in our laboratory to identify new directing group auxiliaries to enable attenuation the photochemical properties of the resulting palladacycle intermediates, along with the development of catalytic C–H halogenation reactions.

## Conflicts of interest

There are no conflicts to declare.

## Supplementary Material

SC-011-C9SC05722F-s001

SC-011-C9SC05722F-s002
