# Supplementary material for: Photoexcited Pd(ii) auxiliaries enable light-induced control in C(sp3)–H bond functionalisation
Source: Chem Sci. 2020 Jan 23;11(9):2455–63. doi: 10.1039/c9sc05722f (PMC8157331; doi:10.1039/c9sc05722f)
Supplement: SC-011-C9SC05722F-s001 [file SC-011-C9SC05722F-s001.pdf]

Electronic Supplementary Material (ESI) for Chemical Science.
This journal is © The Royal Society of Chemistry 2020

Supporting Information

Photoexcited Pd(II) Auxiliaries Enable Light-Induced Control in C(sp^3)-H Bond Functionalisation

Milena L. Czyz,[a] Geethika K. Weragoda,[b] Tyra H. Horngren,[a] Timothy U. Connell,[c] Daniel Gomez,[c] Richard A. J. O'Hair[a]* and Anastasios Polyzos[a,b]*

[a] School of Chemistry, The University of Melbourne, Melbourne, Victoria 3010, Australia

[b] CSIRO Manufacturing, Research Way, Clayton VIC 3168, Australia, Victoria 3000, Australia

[c] RMIT University, Melbourne

*Correspondence to: anastasios.polyzos@unimelb.edu.au and rohair@unimelb.edu.au

Contents

Experimental Procedures 3

General Information 3

Steady-State UV-Vis Spectroscopy 5

Emission Lifetime Decay 6

Stern-Volmer Fluorescence Quenching Studies 7

Electrochemistry 9

  Palladacycle 1 9

  Reduction Potentials of Halogenating Reagents 10

Gas-Phase Mass Spectrometry Experiments (Photoreaction Screening) 11

Computational Studies 16

  Thermodynamics of the Reductive Elimination Step 21

  Cartesian coordinates and energies for all DFT calculated structures using M06/6-311G(d)/SDD(Pd) 24

Optimisation and Control Experiments 40

  Importance of Light, Oxidant and Halogenation Source 40

  Solvent Effect 40

  Homolysis of Perfluorobutyl Iodide 2 Under Blue Light Irradiation 41

  Stability of Palladacycle in the Presence of Iodinated Product 3 Under Blue Light Irradiation 41

Synthesis and Characterization 42

  General Procedure for the Preparation of *N*-(5-Chloroquinolin-8-yl) Amides 43

  Substrate Characterisation Data 44

  General Procedure for the Preparation of the Charge-Tag Complex 49

  General Procedure for the Preparation of Cyclopalladated Complexes 50

  Cyclopalladated Complexes Characterisation Data 51

  General Procedure for the Iodination Reaction 53

  Product Characterisation Data 54

  General Procedure for the Chlorination Reaction 59

  Product Characterization Data 60

  General Procedure for the Bromination Reaction 62

  Product Characterisation Data 63

  General Procedure for the Removal of Aminoquinoline Directing Group[8] 65

  Product Characterisation Data 66

References 66

^{1}H and ^{13}C NMR Spectra 67

X-Ray Data 151

Experimental Procedures

General Information

Synthesis and General Instrumentation: Unless otherwise stated, reagents and solvents were purchased from commercial sources and used without further purification. Cholic acid derivative **21a** was synthesized according to a literature procedure.[1]

Analytical Thin Layer Chromatography (TLC) was carried out using aluminium-backed Merck Kieselgel KG60 F254 silica plates. The plates were visualized by irradiation with short-wave ultraviolet light. Flash chromatography was performed on Grace Davidson Davisil LC60A 40-63 micron silica gel.

All light-promoted halogenation reactions were performed using a custom-built blue LED photoreactor (Figure S1). The custom reactor was comprised of a square metal frame (100 mm × 110 mm) with 14 blue LEDs (2 × SP-02-V4 LED assembly, each consisting of 7 LXML-PR02-A900 Royal-Blue LUXEON Rebel ES LEDs; www.luxeonstar.com). The photoreactor was fitted with PC cooling fans and heat sinks for temperature control (internal temperature of the reactor typically reaches 27 ± 5 °C overnight). The power of the light source can be varied using an intensity control.

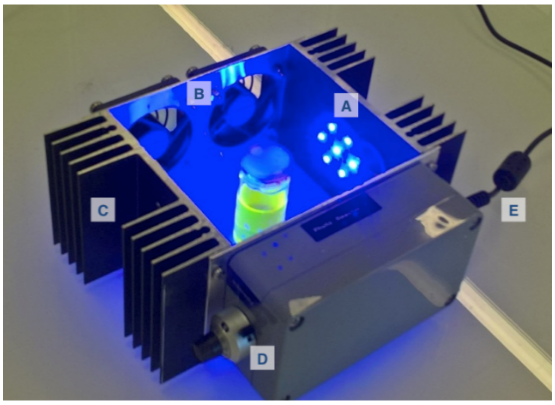

Figure S1. Custom-built LED photoreactor; a) blue LED assembly; b) PC cooling fans; c) heat sinks; d) intensity control; e) 12 V power supply.

^{1}H NMR and ^{13}C NMR spectra were recorded on Bruker 400 Ultrashield (400 MHz), Bruker 500, Ascend 400 (400 MHz) or Varian MR-400. Chemical shifts are expressed in parts per million (PPM) and are referenced to the internal solvent peaks.

Mass spectrometric analyses were performed on a Thermo Scientific Q Exactive mass spectrometer fitted with an ASAP ion source. Positive ions were recorded in an appropriate mass range at 140,000 mass resolution. The APCI probe was used without flow of solvent. The nitrogen nebulizing/desolvation gas used for vaporization was heated to 350 °C in these experiments. The sheath gas flow rate was set to 25, the auxiliary gas flow rate to 5 and the sweep gas flow rate to 2 (all arbitrary units). The discharge current was 4 mA and the capillary temperature was 320 °C.

UV-Vis Spectroscopy: Absorption spectra were measured using a Perkin Elmer Lambda 1050 spectrometer. Steady-state luminescent spectra were recorded using a Perkin Elmer LS50 fluorimeter. Samples were excited at 400 nm, slit widths were 2.7 nm and the area under the spectra were used for determining the Stern-Volmer quenching values.

Electrochemistry: The cell comprised a cylindrical glass vessel with a flat base and custom-built Teflon lid[2] with holes to fit the electrodes (glassy carbon working (CH Instruments), platinum wire counter and either silver wire or 'leakless' Ag/AgCl (eDAQ) reference). The cell was housed in a light-tight Faraday cage. Potentials were applied using an Autolab PGSTAT204 potentiostat, and referenced *in situ* to the ferrocenium/ferrocene ($Fc^{+/0}$) couple, and converted to the reported reference (SCE) using $Fc^{+/0}$ = +0.4 V.[3] Freshly distilled acetonitrile with 0.1 M $TBAPF_6$ supporting electrolyte was used as the solvent. Electrodes were cleaned/polished prior to each experiment. The working electrodes were polished using 0.05 µm alumina powder on a felt pad with deionized water. All electrodes were rinsed with deionized water, ethanol and acetone. The solvent was degassed with nitrogen or argon for 5 minutes.

Steady-State UV-Vis Spectroscopy

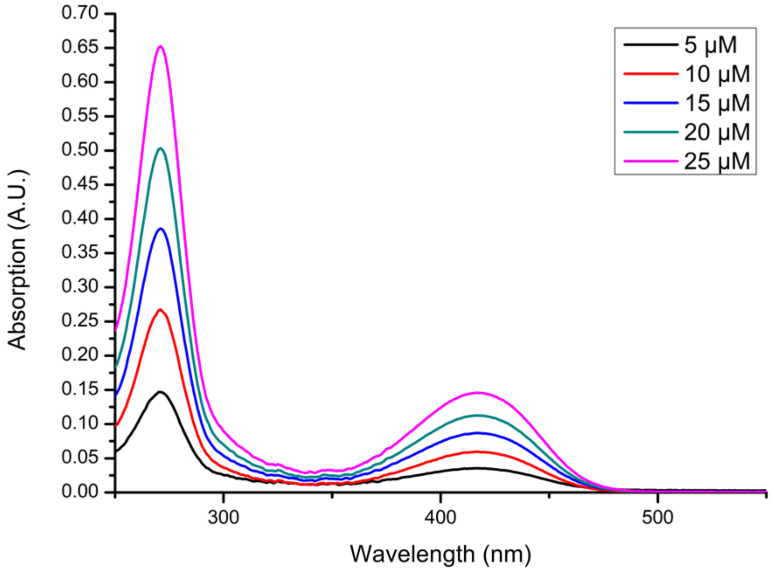

Figure S2: Steady-state absorption spectra of palladacycle **1** at different concentrations, collected at room temperature in acetonitrile. The stock solution of palladacycle **1** in acetonitrile (0.01 M) was diluted to a given concentration in volumetric flasks, and then transferred to a septum-capped quartz cuvette.

A

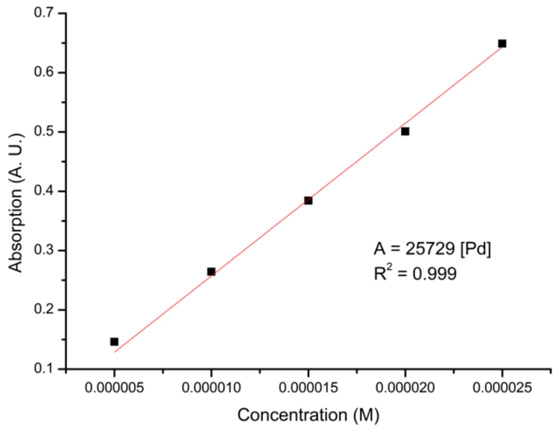

B

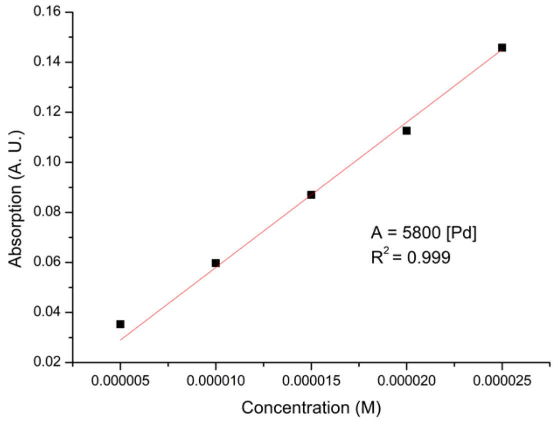

Figure S3: Extinction coefficient calibration curve at 272 nm (**A**) and 417 nm (**B**) for palladacycle **1** in acetonitrile.

The emission spectra were recorded under air (septum-capped quartz cuvette prior to any degassing) to give a single emission band at λ_{max} = 530 nm. The sample was then sparged with argon for approximately 15 minutes, while care was taken to avoid evaporation of solvent. Emission at λ_{max} = 530 nm was found not to be oxygen-sensitive; however, a second, overlapping emission band at λ_{max} = 625 nm was observed in the absence of oxygen.

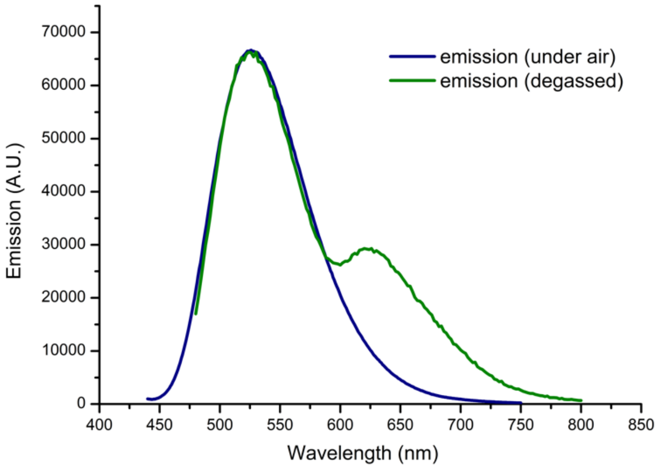

Figure S4: Photoluminescence emission spectra of palladacycle **1** in acetonitrile (20 μm) collected at room temperature, under air (blue) and after sparging with argon for 15 minutes (green). Excitation wavelength = 430 nm. Slit widths = 4 nm.

Emission Lifetime Decay

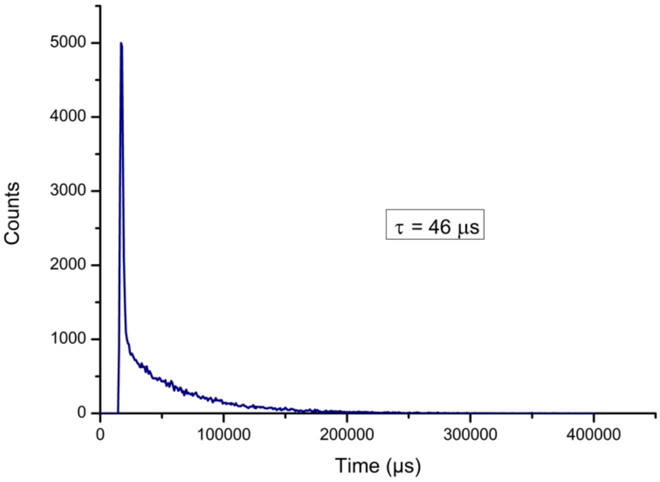

Figure S5: Emission lifetime decay of palladacycle **1** at 625 nm.

Stern-Volmer Fluorescence Quenching Studies

Samples for steady-state photoluminescence were prepared in the following manner: quencher was added to an acetonitrile solution of palladacycle **1** (0.01 mM) and made to a given concentration in a volumetric flask. The solution was then transferred to a septum-capped quartz cuvette. To observe quenching of the triplet excited state, the solution was deoxygenated immediately prior to measurement by sparging with argon for approximately 10 min. Fluorescence quenching studies were performed under air.

A

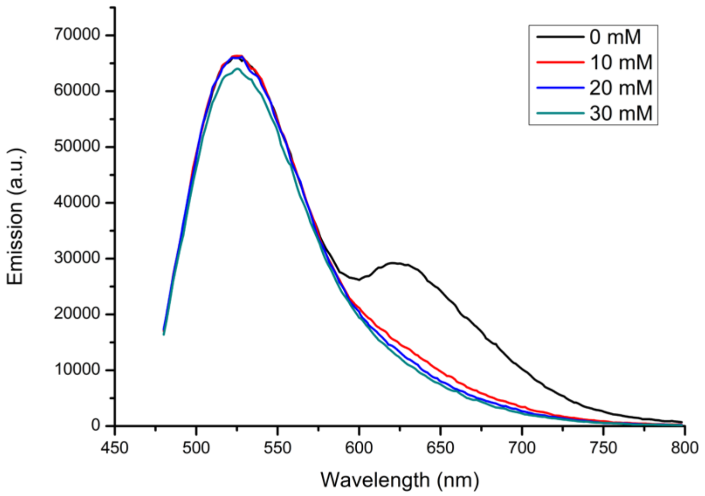

B

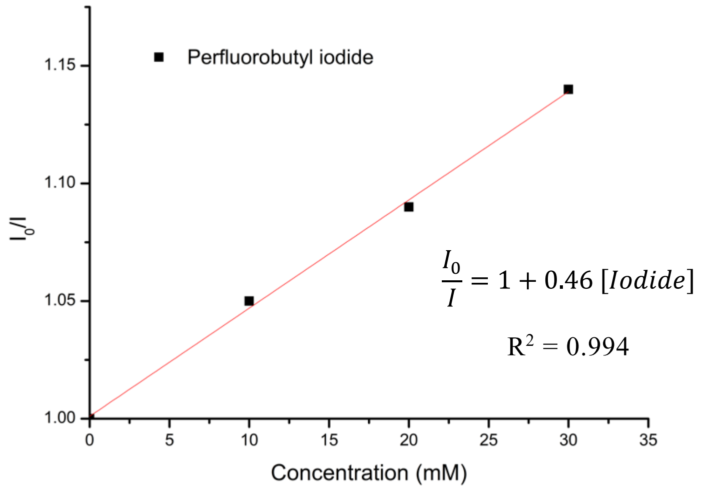

C

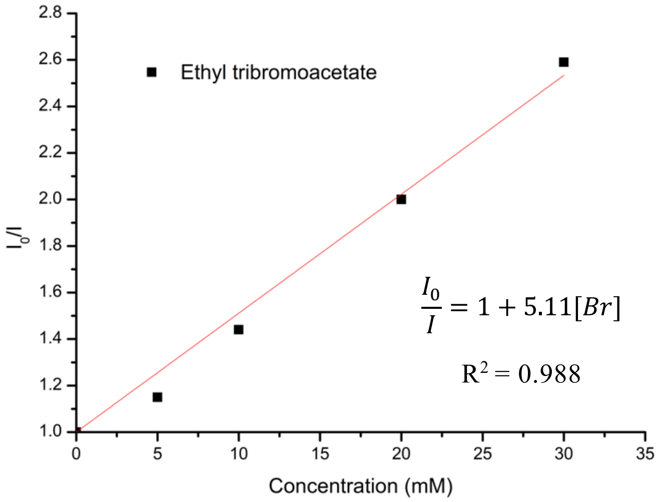

D

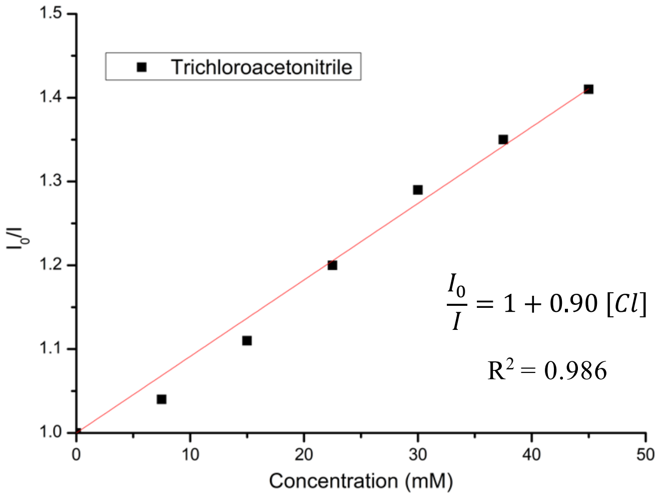

Figure S6: (A) Photoluminescence quenching of the singlet and triplet emission of palladacycle **1** by perfluorobutyl iodide **2**. (B) Stern-Volmer plot palladacycle **1** fluorescence intensity in the presence of perfluorobutyl iodide **2**. (C) Stern-Volmer plot palladacycle **1** fluorescence intensity in the presence of ethyl tribromoacetate **11**. (D) Stern-Volmer plot palladacycle **1** fluorescence intensity in the presence of trichloroacetonitrile **12**.

Electrochemistry

Palladacycle 1

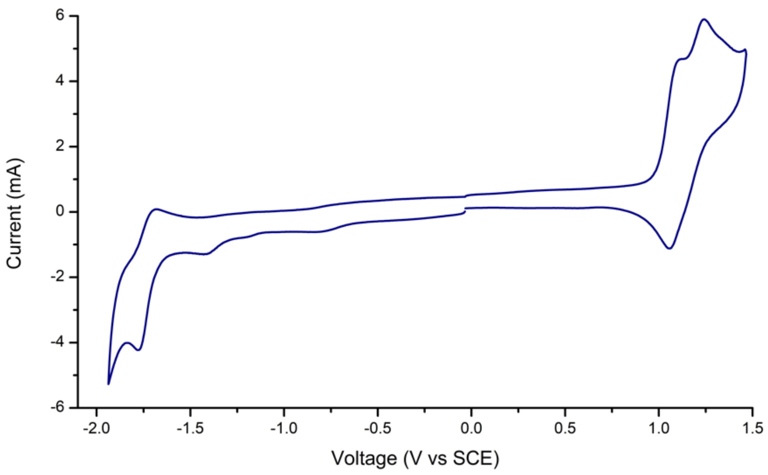

Figure S7: Cyclic voltammogram of palladacycle **1** (1 μm) measured in degassed acetonitrile (Bu_4NPF_6 as the supporting electrolyte), scan rate = 0.1 Vs^{-1}, reported relative to SCE using $Fe^{+/0}$ couple as an internal standard; reference electrode Ag^+/Ag, working electrode: glassy carbon; auxiliary electrode: Pt wire.

Two closely overlapping oxidation waves were observed at E_p = 1.10 and 1.24 V and a reduction wave was observed at E_p = -1.78 V. ΔE_p values, ($\Delta E_p > 0.059$) suggest that both oxidation and reduction are quasi-reversible one-electron processes.[4]

Table S1: Electrochemical data for palladacycle **1**

Sweep rate	E_{pc}	E_{pa}	ΔE_p
**Oxidation**			
**0.1**	1.10	1.03	0.070
	1.24	-	-
**Reduction**			
**0.1**	-1.69	-1.78	0.086

Reduction Potentials of Halogenating Reagents

A

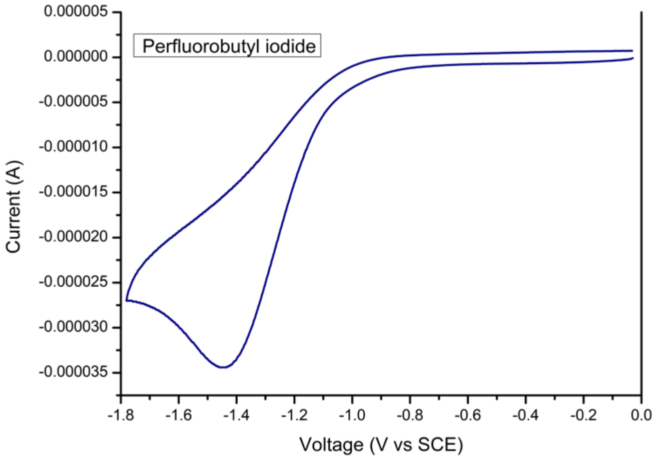

B

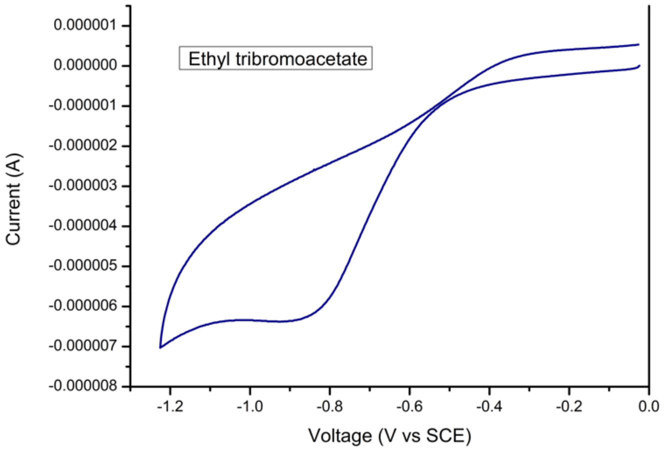

C

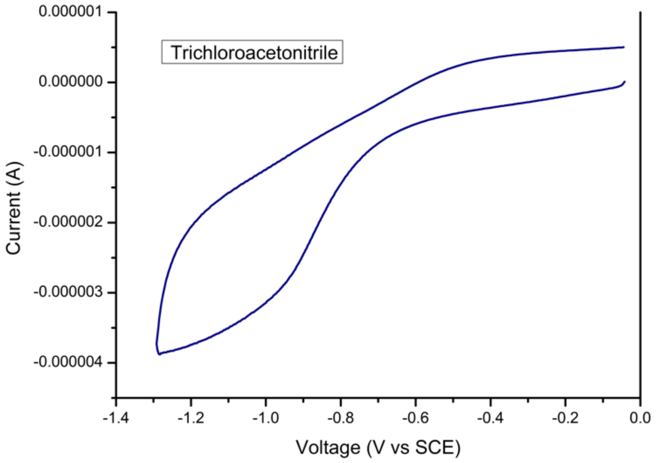

Figure S8: Cyclic voltammograms of perfluorobutyl iodide (**A**), ethyl tribromoacetate (**B**) and trichloroacetonitrile (**C**) measured in acetonitrile (Bu_4NPF_6 as the supporting electrolyte), scan rate = 0.1 Vs^{-1}, reported relative to SCE using $Fe^{+/0}$ couple as an internal standard; reference electrode Ag^+/Ag, working electrode: glassy carbon; auxiliary electrode: Pt wire.

Gas-Phase Mass Spectrometry Experiments (Photoreaction Screening)

The experiments were conducted using two different mass spectrometers equipped with electrospray ionization (ESI) sources. ESI high resolution mass spectra (HRMS) were collected on a Thermo OrbiTrap Fusion Lumos mass spectrometer. Tandem mass spectrometry experiments (MS/MS) were performed on a Thermo Scientific (Bremen, Germany) linear ion trap (LTQ). For both sets of mass spectrometry experiments the charge tag complex **4** was applied using the standard iodination protocol in continuous flow (20 min retention time), which was then infused directly into the ESI source to access online reaction monitoring platform (Figure S9). The reaction mixture was prepared in acetonitrile (100 μM) and pumped into the electrospray source through the 448 nm LED photoreactor (Figure S1). The progress of the reaction was monitored (I) in the absence of oxone and (II) in the presence of oxone. Furthermore MS/MS data were collected to gain structural information of the newly formed intermediates by fragmenting the precursor ions.

Typical electrospray source conditions were:

CID: Sheath Gas = 10 arbitrary units, Auxiliary Gas = 5 arbitrary units, Sweep Gas = 0 arbitrary units, Spray Voltage = 4 kV, Capillary Temp. = 250 °C, Capillary Voltage = 2 V, Tube Lens Voltage = 75 V. The precursor ion was mass selected with a window of 1 *m/z* and collision induced dissociation was carried out using the helium bath gas by activating the ion with an activation time of 30 ms. A normalized collision energy (NCE) was chosen to deplete the precursor ion to 10%.

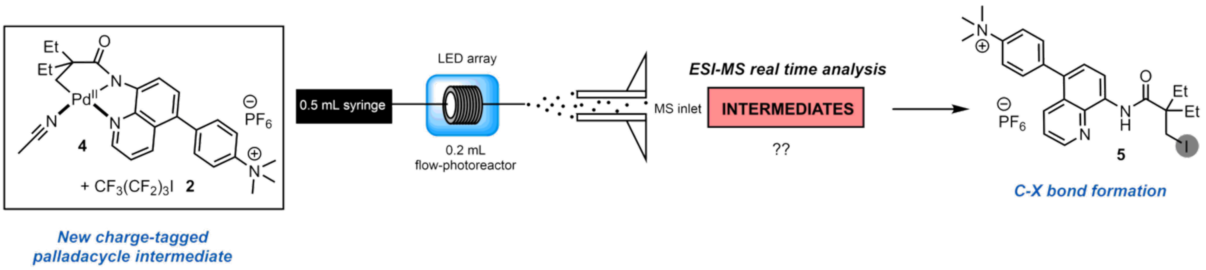

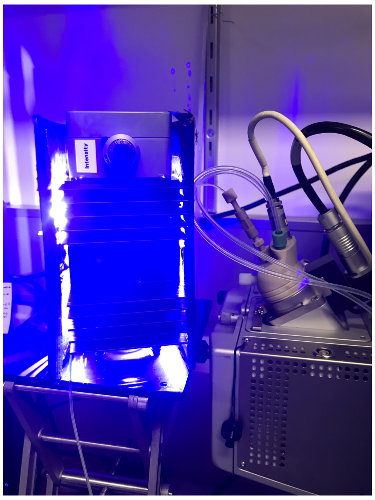

Figure S9: Schematic representation of the real-time photoreaction screening platform using the charge tagged palladacycle **4**. The progress of the reaction and intermediates were monitored at 100 μM concentration in acetonitrile on the positive-ion mode with the 448 nm (15 W) blue LED photoreactor.

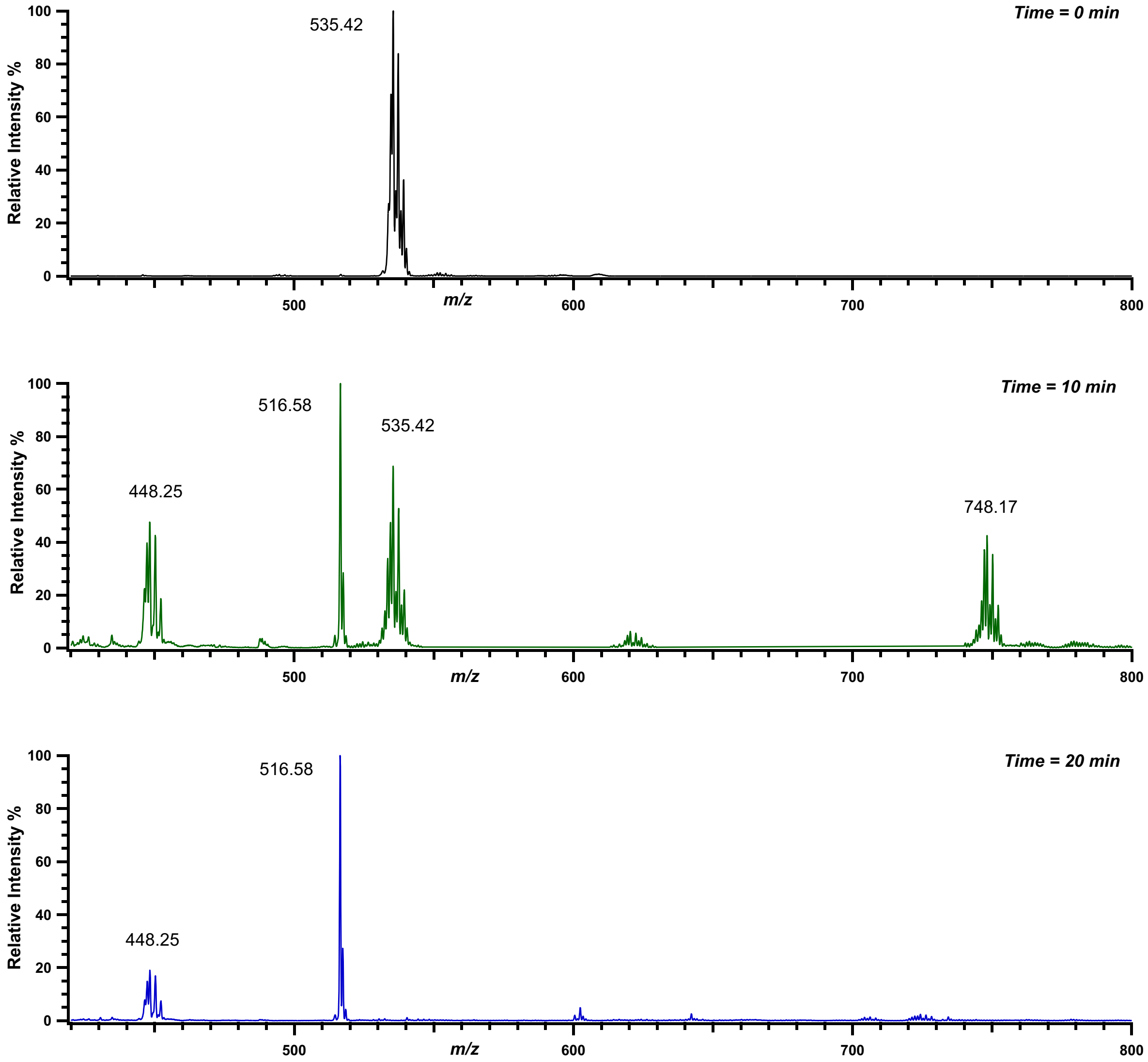

Figure S10: ESI-HRMS used to monitor the progress of the reaction in the absence of oxone: Mass spectra of the reaction mixture taken at different time points: (top) t = 0 min; (middle) t = 10 min and (bottom) t = 20 min.

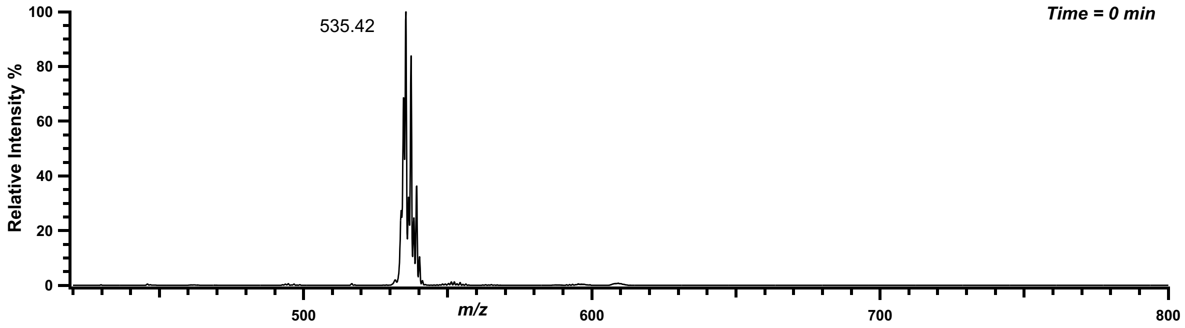

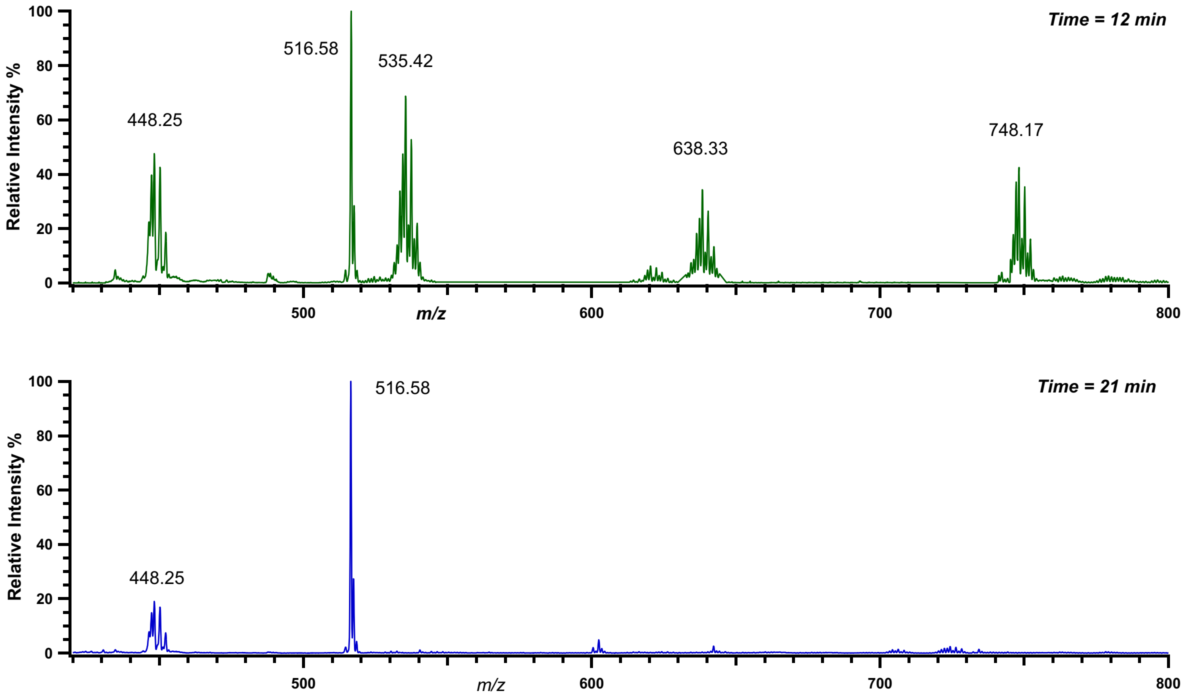

Figure S11: ESI-HRMS used to monitor the progress of the reaction in the presence of oxone: Mass spectra of the reaction mixture taken at different time scales (top) t = 0 min; (middle) t = 12 min and (bottom) t = 21 min.

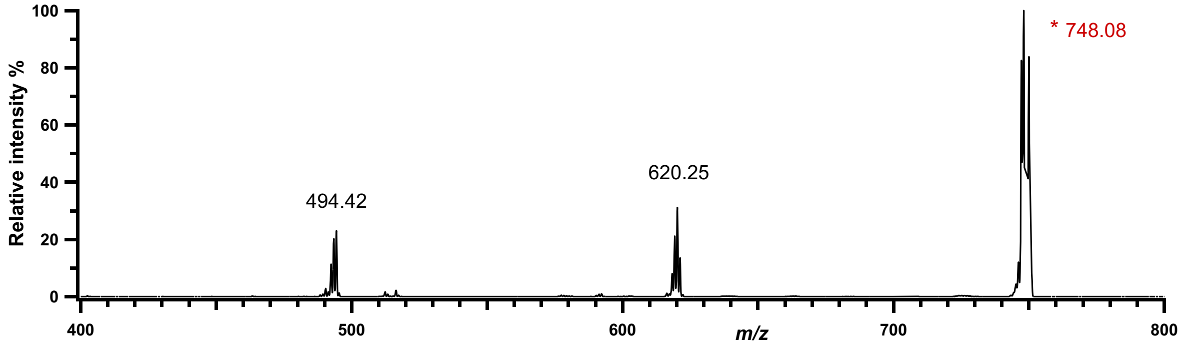

Figure S12: CID spectrum of palladium complex **6** at *m/z* 748, at a normalized collision energy of 10. The mass-selected precursor ions are designated by *.

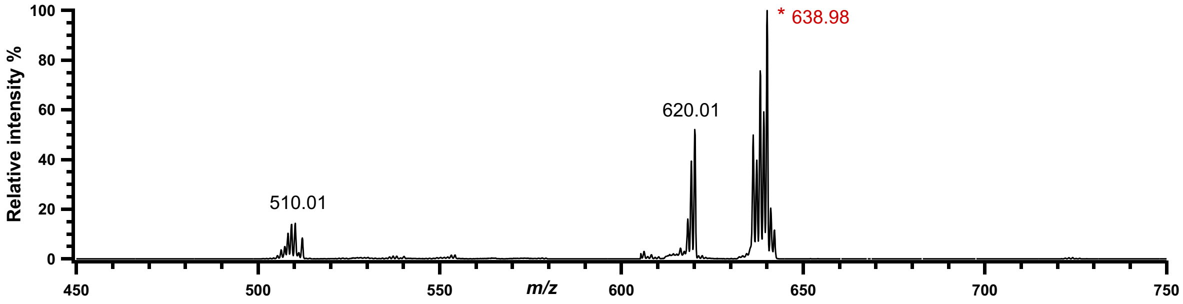

Figure S13: CID spectrum of palladium complex **8** at *m/z* 638, at a normalized collision energy of 12. The mass-selected precursor ions are designated by *.

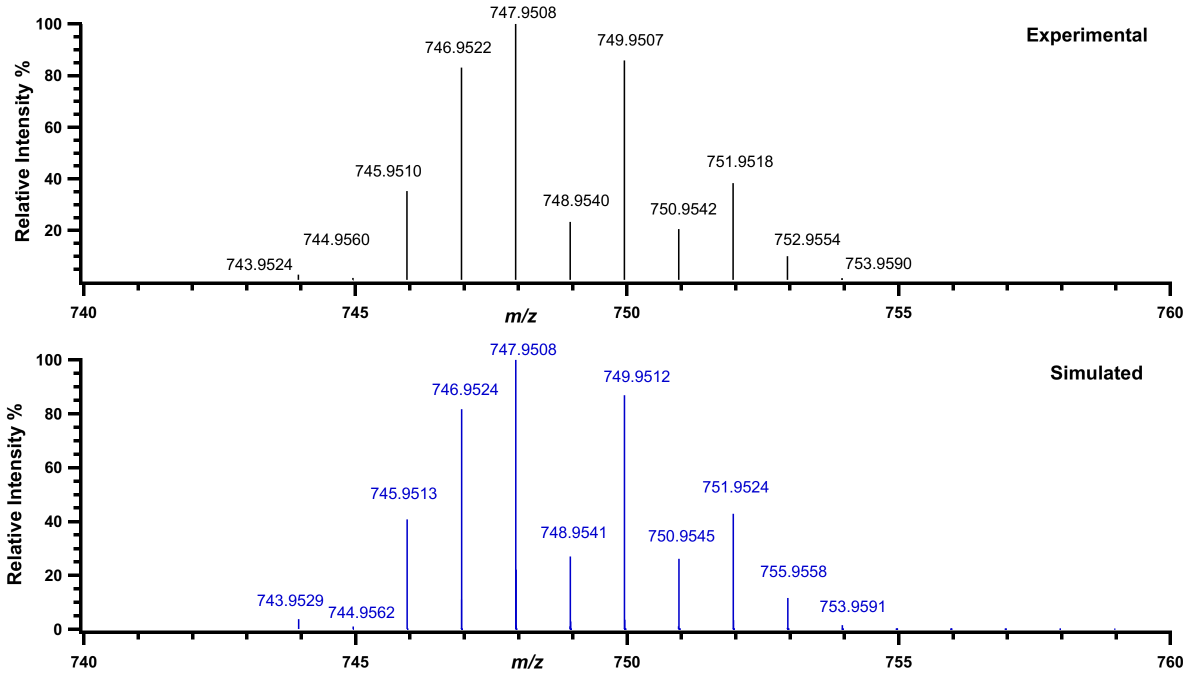

Figure S14: High resolution (black) and simulated (blue) mass spectra of the ion at *m/z* 748.

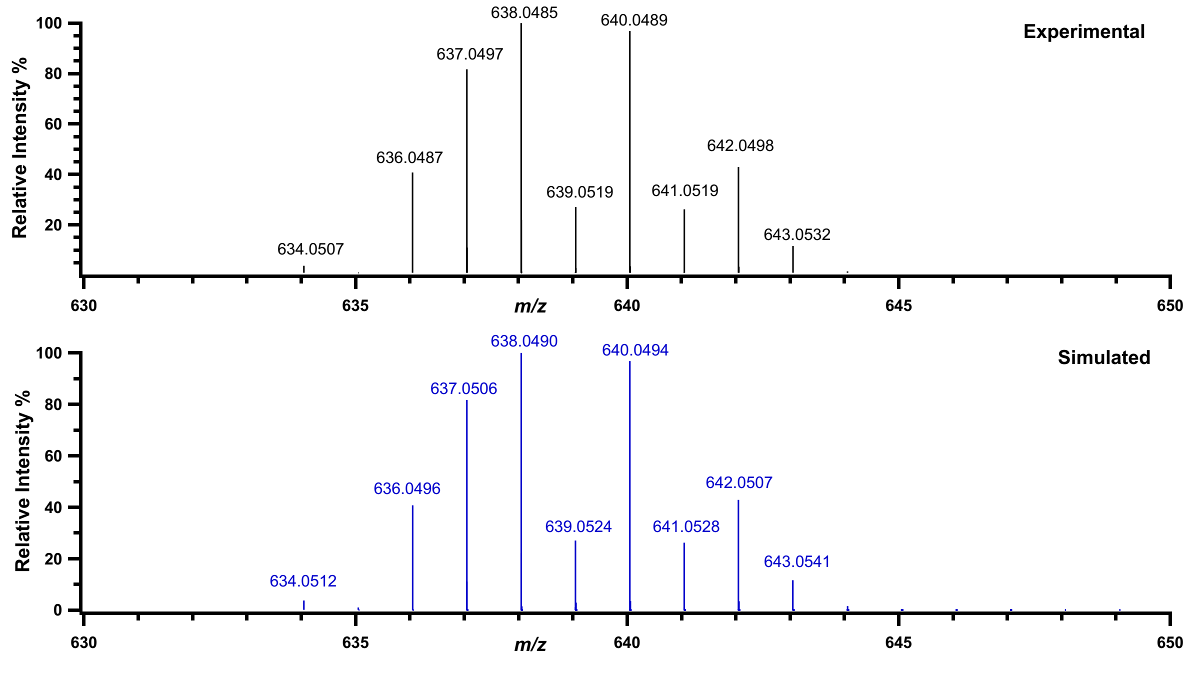

Figure S15: High resolution (black) and simulated (blue) mass spectra of the ion at *m/z* 638.

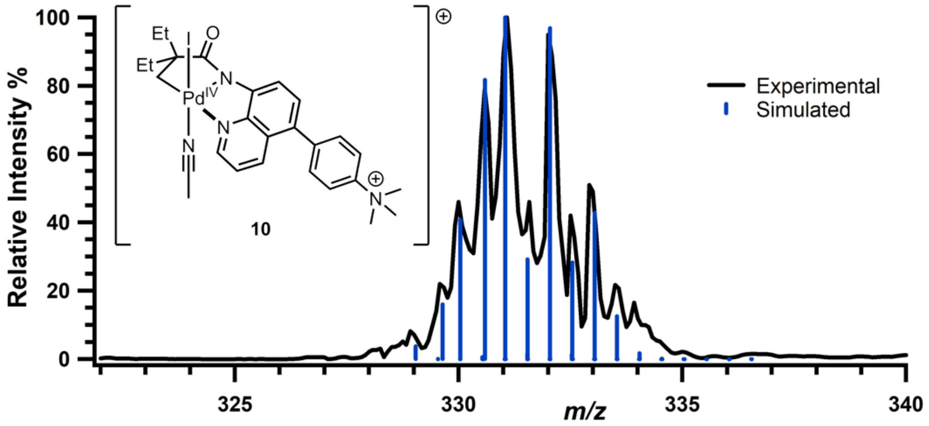

Figure S16: Low resolution (black) and simulated mass spectra of the ion at *m/z* 331.

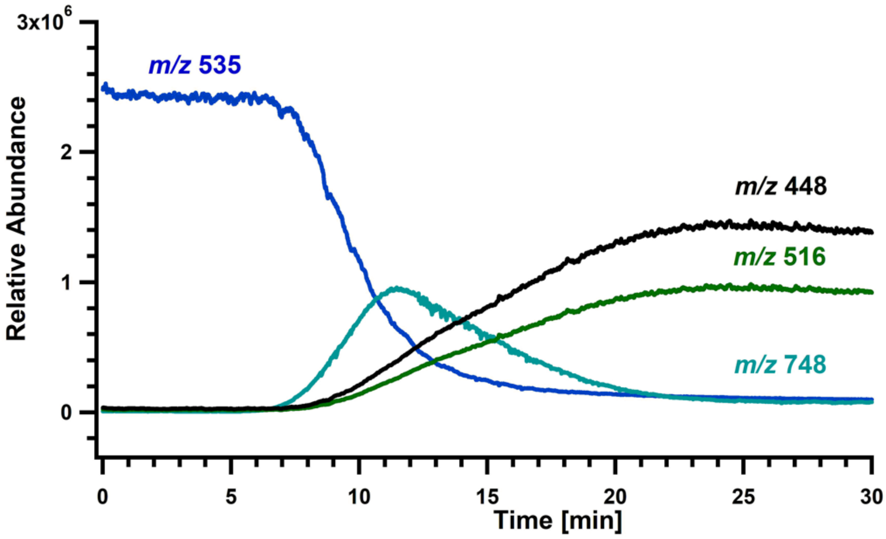

Figure S17: Mass spectra traces of relative abundance (of substrate, intermediates and product) vs time for the iodination reaction run in the *absence* of oxone.

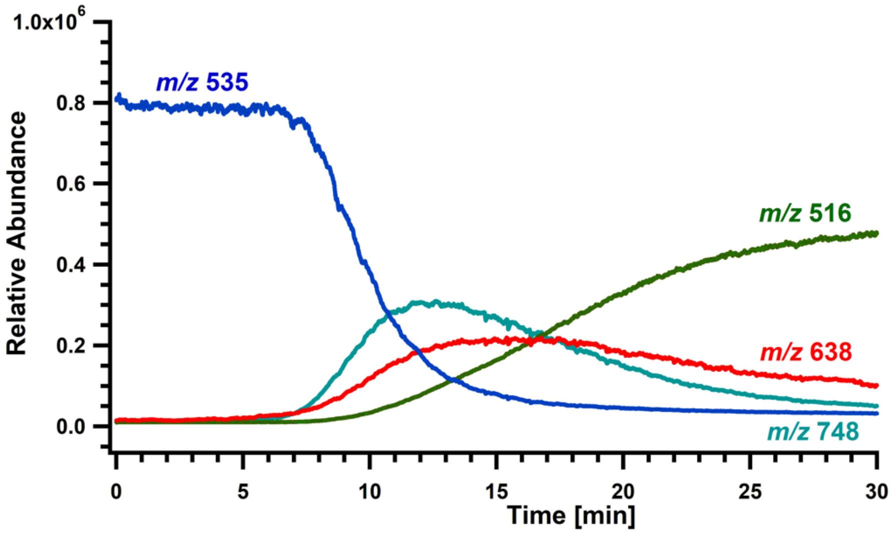

Figure S18: Mass spectra traces of relative abundance (of substrate, intermediates and product) vs time for the iodination reaction run in the *presence* of oxone.

Computational Studies

Density Functional Theory (DFT) calculations were carried out with the Gaussian09[5] program. Ground state singlet optimizations were performed on the crystal structure with M06 functional. SDD basis set was chosen for Pd and I while 6-311+G(d) was used for the other atoms. The CPCM formalism for the Self Consistent Reaction Field (SCRF) model of solvation was employed to account for solvation in acetonitrile. All ground and transition states were verified by vibrational frequency analysis at the same level of theory, with all minima showing no imaginary frequencies and all transition states showing only one imaginary frequency. Intrinsic reaction coordinate (IRC) calculations were used to confirm the connectivity between transition structures and minima. The cartesian coordinates and corresponding energies for all optimized ground and transition state structures, including the imaginary frequencies of the transition states are available in the Supporting Information.

Time-dependent density functional theory (TD-DFT) calculations were performed on the ground state optimized structure to probe the nature of the electronic transitions in acetonitrile. Different combinations of functionals (M06, ωB97X-D, B3LYP) and basis sets were tested and compared with the experimental absorption spectrum. M06/6-311+G(d)/SDD(Pd) provided the best reproducible spectrum to the experimental absorption spectrum (Figure S19).

Molecular orbital analysis were performed at the BP86/def2-TZVP level,[6] which has been shown to give realisable results. QMForge program was used to analyse the molecular orbitals at the ground state and the triplet excited states.

Table S2: Comparison of bond lengths and bond angles of the crystal structure of the palladacycle **1** with the DFT computed ground state optimised structures at different level of theories and 6-311+G(d)/SDD(Pd) basis set.

Bond Length	Crystal Structure	M06	B3LYP	ωB97X-D
Pd-N40	1.96	1.97	1.20	1.98
Pd-N39	2.11	2.14	2.19	2.17
Pd-N41	2.02	2.03	2.04	2.04
Pd-C17	2.01	2.02	2.03	2.01
N40-C15	1.37	1.36	1.37	1.36
C15-O42	1.23	1.23	1.23	1.22
N40-C13	1.39	1.39	1.39	1.38
C13-C11	1.38	1.38	1.39	1.38
C9-C11	1.40	1.40	1.40	1.40
C8-C9	1.37	1.36	1.37	1.36
C7-C8	1.40	1.41	1.41	1.41
C7-C14	1.41	1.41	1.42	1.41
C14-N39	1.37	1.36	1.37	1.36
N39-C1	1.32	1.32	1.32	1.31
C1-C3	1.39	1.40	1.40	1.40
C3-C5	1.35	1.36	1.37	1.36

C5-C7	1.42	1.41	1.41	1.41
C8-Cl	1.74	1.75	1.76	1.75
C15-C16	1.53	1.53	1.76	1.53
C16-C17	1.54	1.54	1.54	1.54
Bond Angle				
N40-Pd-C17	83.72	83.77	83.90	83.78
N39-Pd-N40	81.01	79.95	79.73	80.00
Pd-N40-C15-O42	179.25	179.43	179.59	178.70

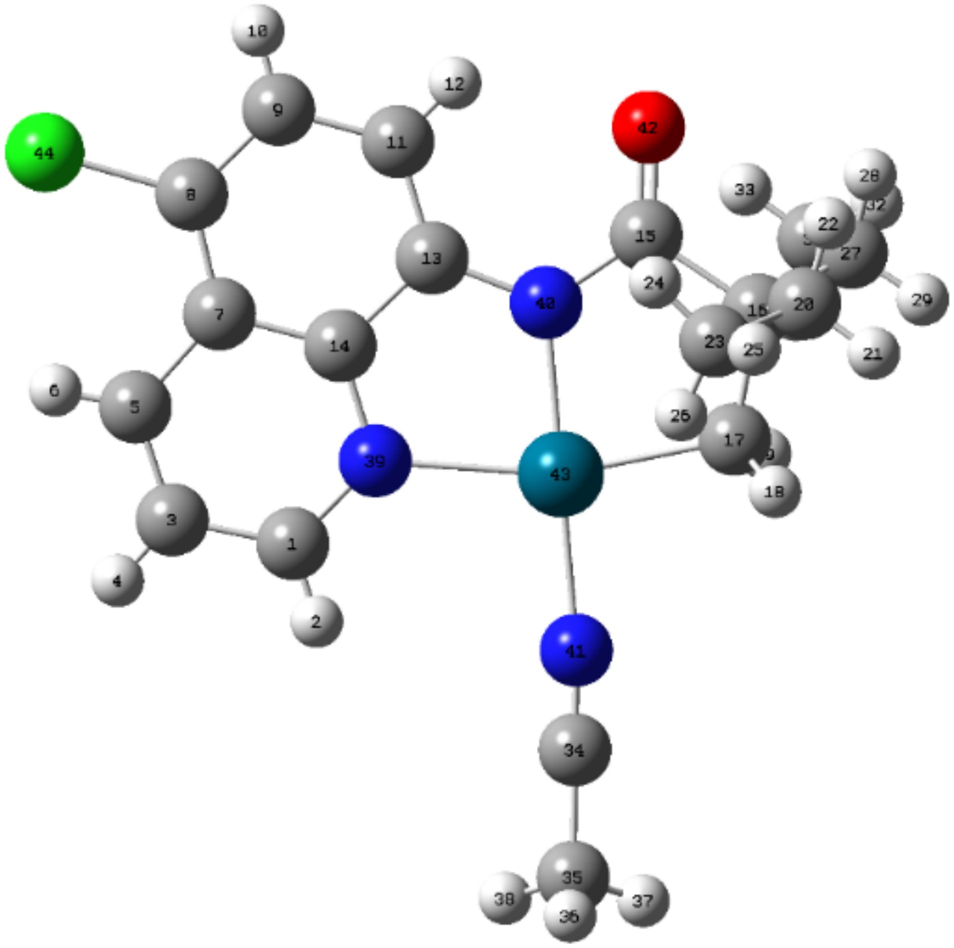

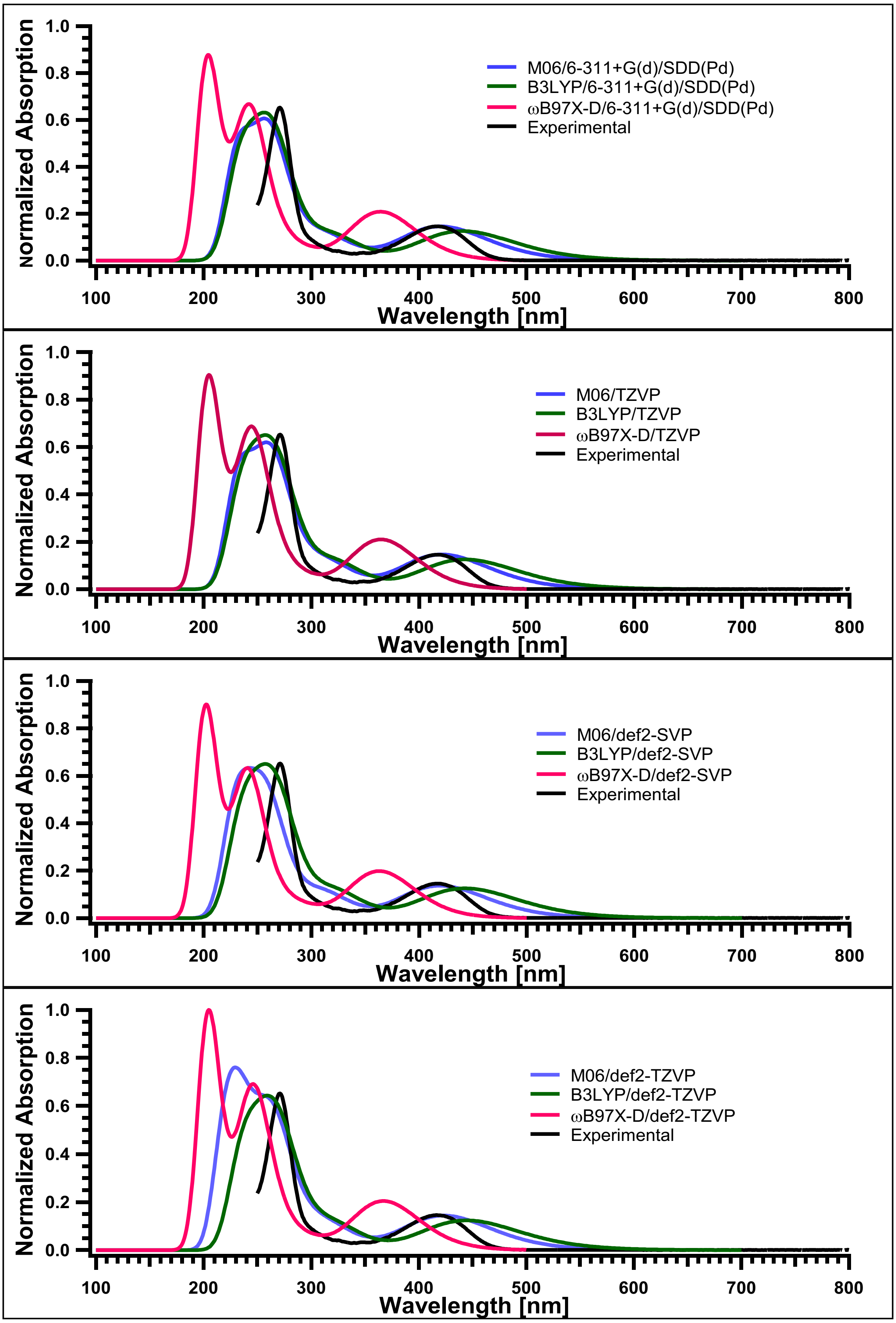

Figure S19: Comparison of experimental absorption spectrum with the TD-DFT spectra of palladacycle **1** calculated at various level of theories and basis sets.

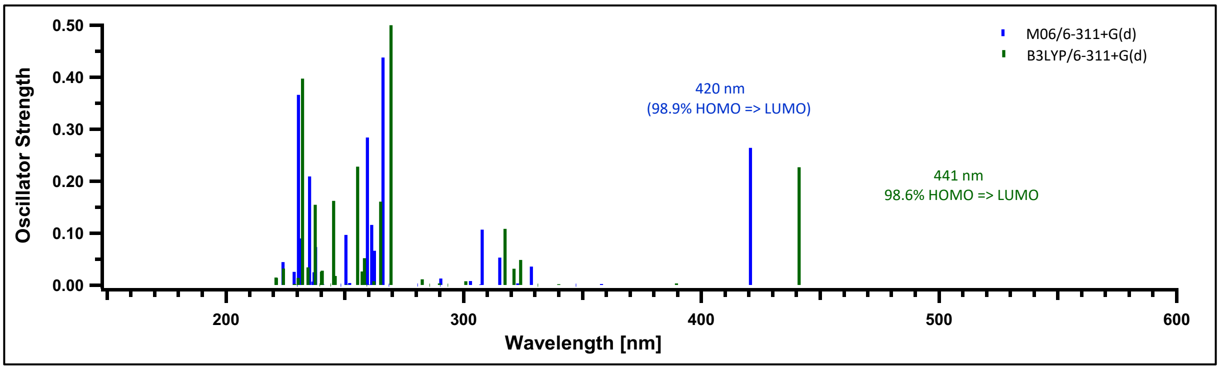

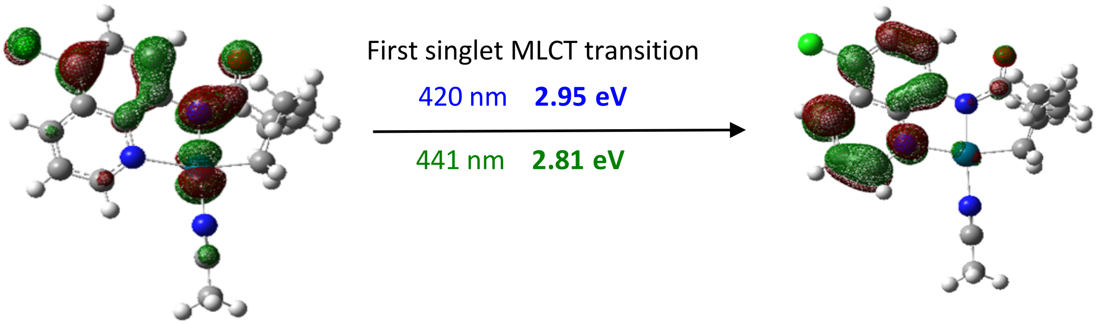

DFT computed values

Method 1: M06 = 2.95 eV

Method 2: B3LYP = 2.81 eV

Value derived from the intersection of the excitation and emission profiles

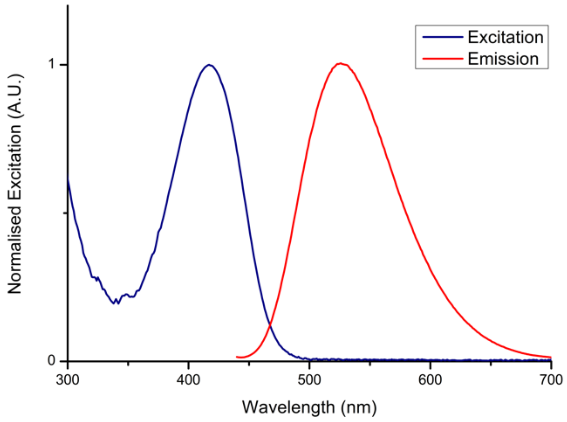

Intersection λ = 467 nm

467 nm = 2.66 eV

Value derived from the electrochemical peak potentials[7] (see Figure S6)

$$E_{MLCT} = -E_{P1} + E_{P2}$$
$$= -1.10\ V - 1.78\ V$$
$$= -2.88\ V$$

Figure S20: Comparison of first singlet MLCT transition ([1]MLCT) energy calculated using TD-DFT method, intersection of the absorption and singlet emission profiles, and electrochemical peak potentials. (a) [1]MLCT transition calculated using M06/6-311G(d)/SDD(Pd) [Blue] and B3LYP/6-311G(d)/SDD(Pd) [Green] (b) Molecular orbitals for first HOMO-LUMO transition at the visible manifold (c) electrochemical peak potentials of palladacycle **1**.

Table S3: Calculated molecular orbital energies (BP86/def2-TZVP)

Orbital	Energy (eV)
LUMO + 6	-0.588
LUMO + 5	-0.936
LUMO + 4	-1.208
LUMO + 3	-1.401
LUMO + 2	-1.578
LUMO + 1	-1.729
LUMO	-2.756
HOMO	-4.71
HOMO - 1	-5.028
HOMO - 2	-5.153
HOMO - 3	-5.559
HOMO - 4	-5.905
HOMO - 5	-5.959
HOMO - 6	-6.378

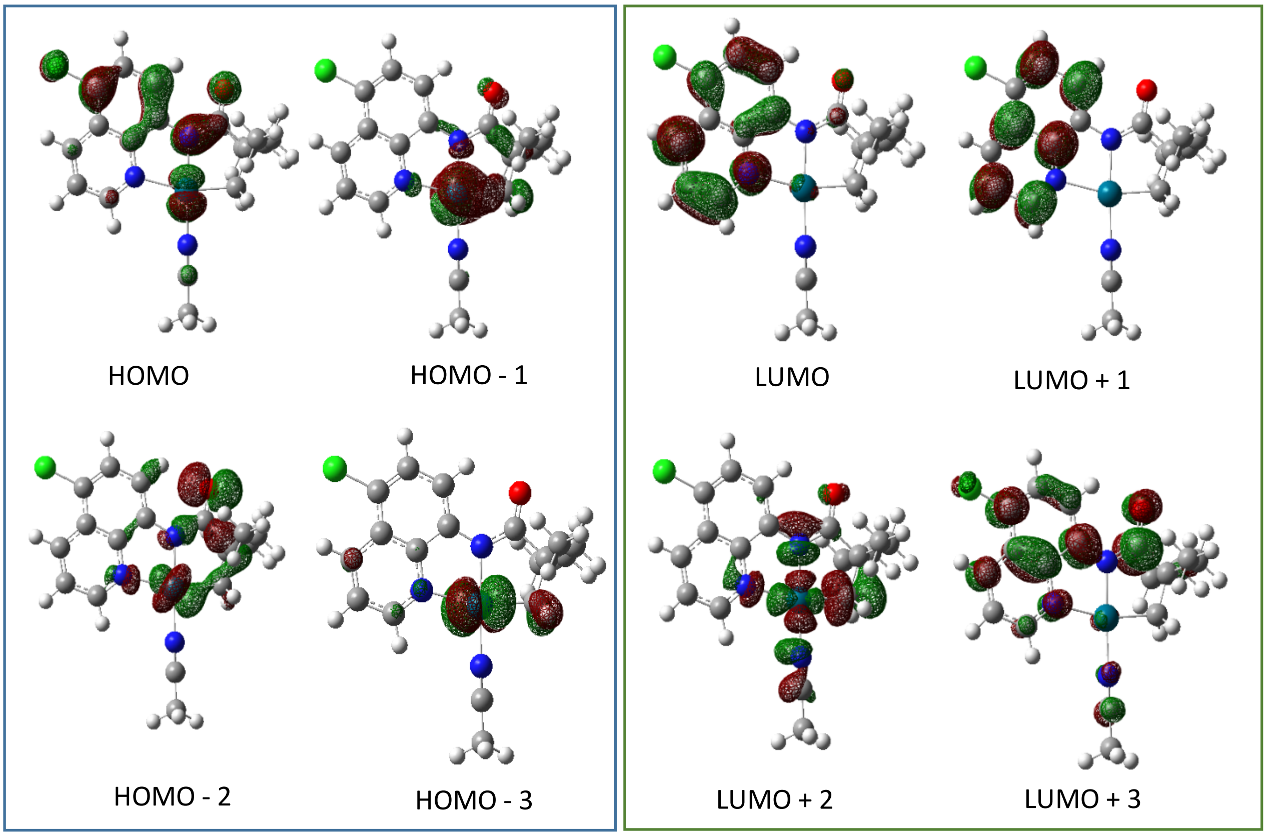

Figure S21: Ground state frontier molecular orbitals for the palladacycle **1** (BP86/def2-TZVP).

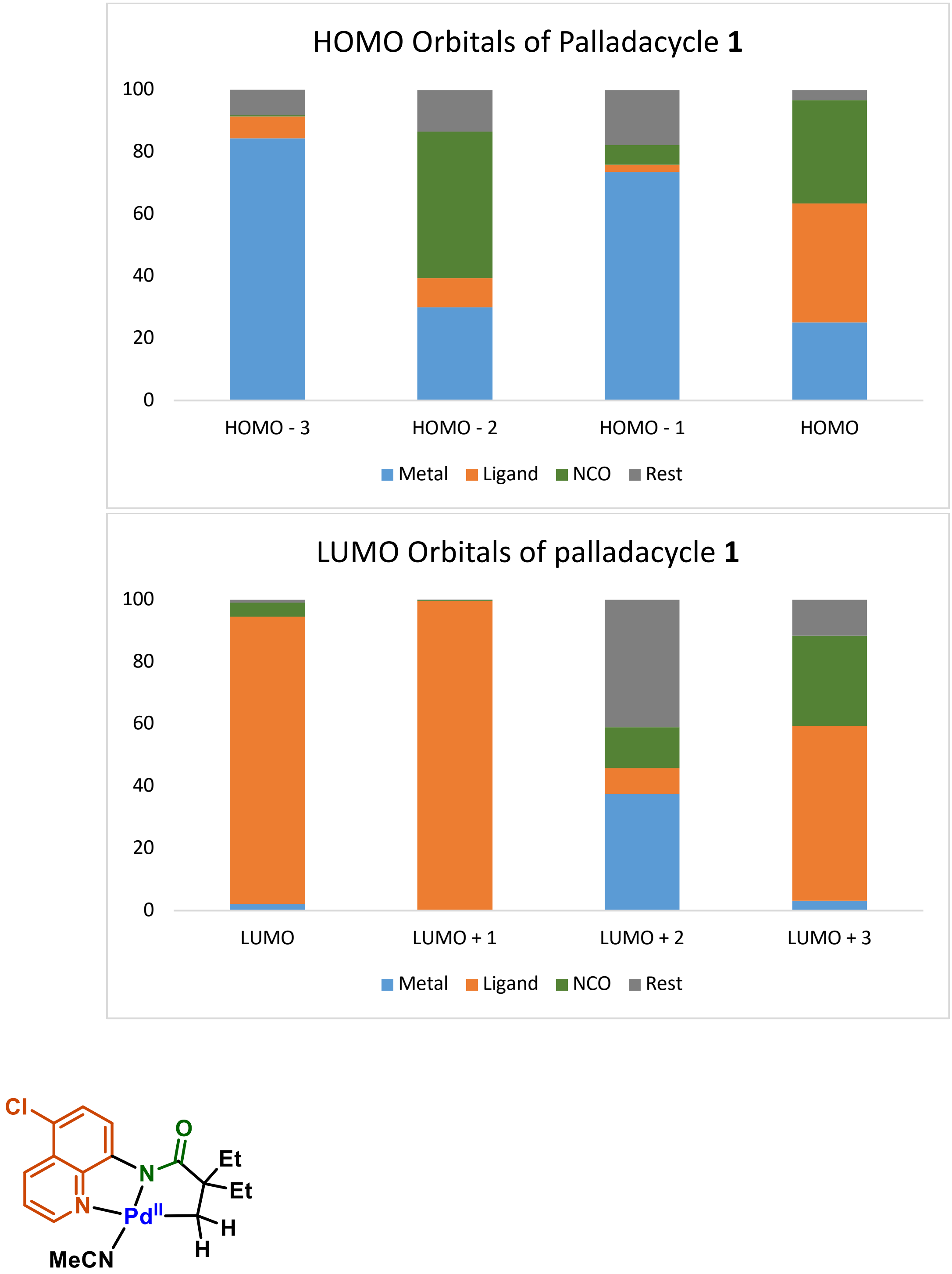

Figure S22: Contribution to the respective molecular orbitals of the metal and ligands in palladacycle **1** (BP86/def2-TZVP).

Thermodynamics of the Reductive Elimination Step

Thermodynamics of the reductive elimination steps in acetonitrile were computed using M06/6-311G(d)/SDD(Pd) and results are summarised in Figure S23, S24 and S25.

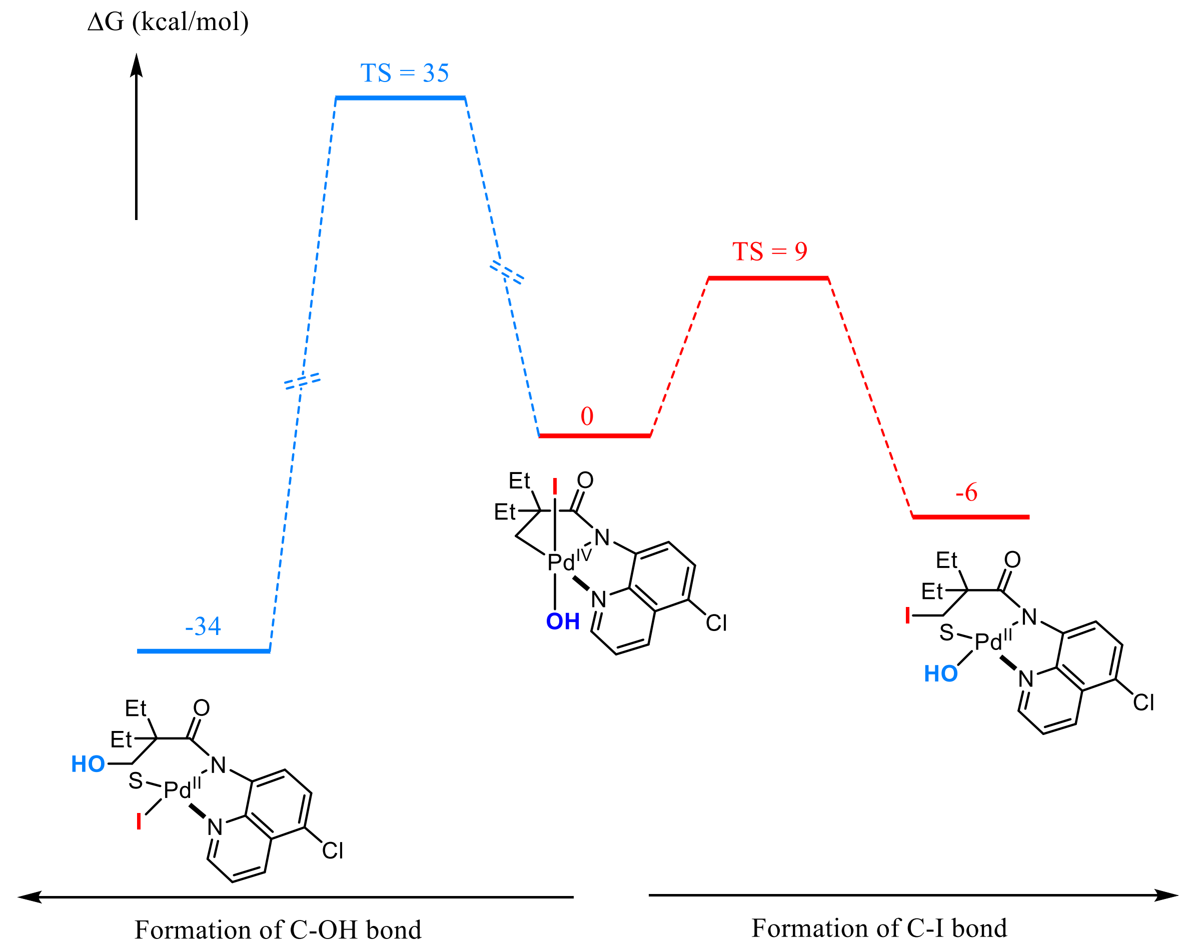

Δ_rE = -36 kcal.mol^{-1}	ΔE (TS) = 33 kcal.mol^{-1}	Δ_rE = -6 kcal.mol^{-1}	ΔE (TS) = 9 kcal.mol^{-1}
Δ_rG = -34 kcal.mol^{-1}	ΔG (TS) = 35 kcal.mol^{-1}	Δ_rG = -6 kcal.mol^{-1}	ΔG (TS) = 9 kcal.mol^{-1}
Δ_rH = -34 kcal.mol^{-1}	ΔH (TS) = 33 kcal.mol^{-1}	Δ_rH = -5 kcal.mol^{-1}	ΔH (TS) = 8 kcal.mol^{-1}

Figure S23: DFT computed comparison of thermodynamics (M06/6-311G(d)/SDD(Pd)) of reductive elimination to forge C-I vs C-OH bonds from Pd(IV).

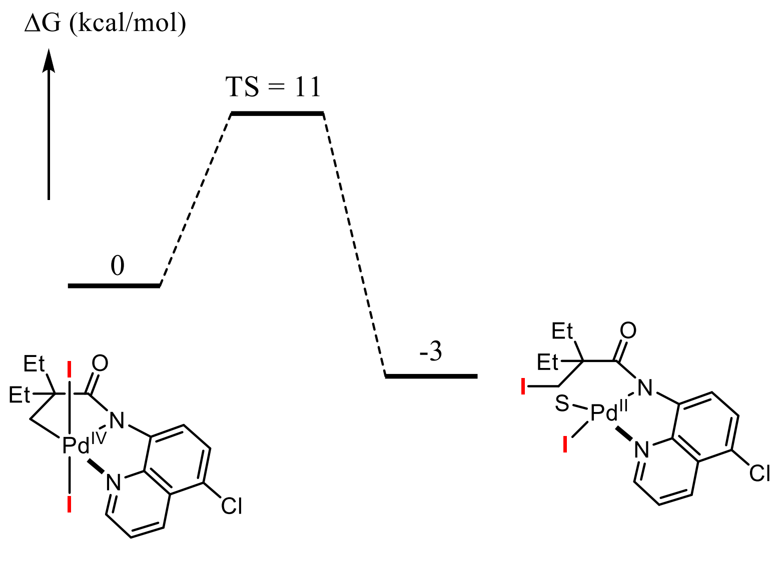

$\Delta_r E$ = -6 kcal.mol^{-1} ΔE (TS) = 10 kcal.mol^{-1}

$\Delta_r G$ = -3 kcal.mol^{-1} ΔG (TS) = 11 kcal.mol^{-1}

$\Delta_r H$ = -6 kcal.mol^{-1} ΔH (TS) = 10 kcal.mol^{-1}

Figure S24: DFT computed thermodynamics (M06/6-311G(d)/SDD(Pd)) of reductive elimination to form C-I bond from Pd(IV).

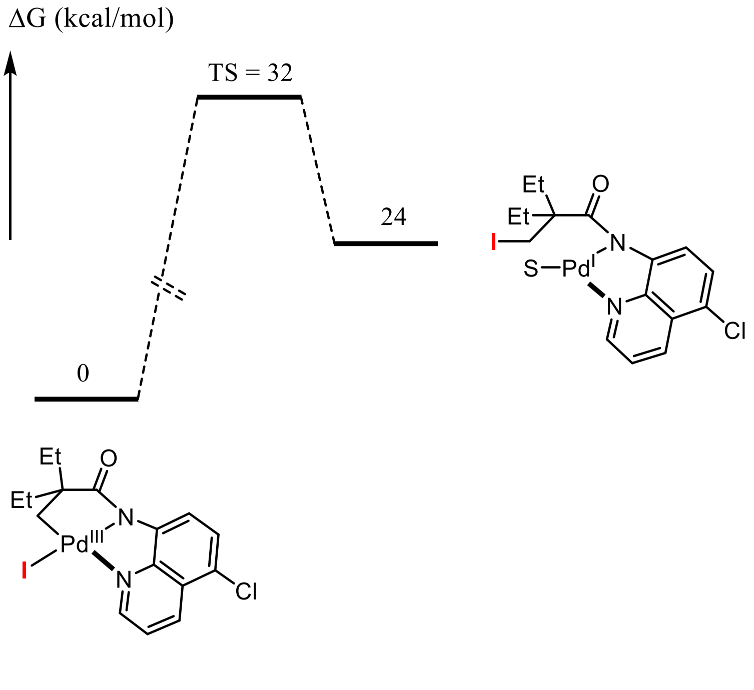

$\Delta_r E$ = 23 kcal.mol^{-1} ΔE (TS) = 30 kcal.mol^{-1}

$\Delta_r G$ = 24 kcal.mol^{-1} ΔG (TS) = 32 kcal.mol^{-1}

$\Delta_r H$ = 23 kcal.mol^{-1} ΔH (TS) = 29 kcal.mol^{-1}

Figure S25: DFT computed thermodynamics (M06/6-311G(d)/SDD(Pd)) of reductive elimination of I from Pd(III). This suggests that the reductive elimination to forge C-I bond from Pd(III) complex is endergonic and has a higher TS barrier.

Cartesian coordinates and energies for all DFT calculated structures using M06/6-311G(d)/SDD(Pd)

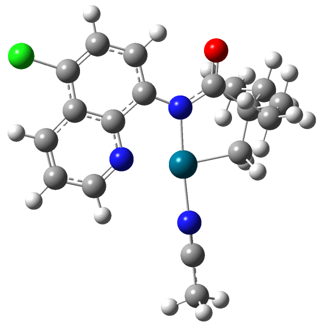

Zero-point correction = 0.345244 (Hartree/Particle)
Thermal correction to Energy = 0.369736
Thermal correction to Enthalpy = 0.370680
Thermal correction to Gibbs Free Energy = 0.289209
Sum of electronic and zero-point Energies = -1524.793668
Sum of electronic and thermal Energies = -1524.769176
Sum of electronic and thermal Enthalpies = -1524.768232
Sum of electronic and thermal Free Energies = -1524.849703

C	1.452587	2.642133	0.004272
H	0.735059	3.460918	0.005866
C	2.831897	2.894860	0.003656
H	3.193756	3.917885	0.004753
C	3.694526	1.834603	0.001657
H	4.768568	1.998586	0.001074
C	3.195712	0.514390	0.000322
C	4.003443	-0.645241	-0.001765
C	3.436617	-1.888769	-0.002899
H	4.073964	-2.769199	-0.004501
C	2.044136	-2.053629	-0.002022
H	1.625038	-3.050260	-0.002968
C	1.188449	-0.962557	-0.000026
C	1.785908	0.342707	0.001147
C	-0.957771	-2.131666	-0.001564
C	-2.465908	-1.864963	-0.000775
C	-2.814105	-0.367702	0.003282
H	-3.393641	-0.078849	0.889451
H	-3.397547	-0.074990	-0.879019
C	-3.051893	-2.555386	1.241491
H	-4.141052	-2.397306	1.229264
H	-2.894623	-3.638174	1.139501
C	-2.472112	-2.090705	2.565081
H	-1.400549	-2.316026	2.633327

H -2.962019 -2.591365 3.407041
H -2.588977 -1.011079 2.715370
C -3.051924 -2.548580 -1.246756
H -2.893907 -3.631830 -1.151133
H -4.141192 -2.391328 -1.233149
C -2.473104 -2.075833 -2.567919
H -2.591300 -0.995529 -2.712084
H -2.962724 -2.572364 -3.412503
H -1.401290 -2.299493 -2.637810
C -2.788284 3.508720 -0.001293
C -3.530833 4.742798 -0.007170
H -3.686382 5.092931 1.016103
H -4.504260 4.593023 -0.480074
H -2.983372 5.508435 -0.561972
N 0.954584 1.425831 0.003031
N -0.198439 -0.996284 0.000938
N -2.196434 2.524280 0.002553
O -0.521901 -3.276769 -0.004302
Pd -1.132752 0.770619 0.003067
Cl 5.749779 -0.496245 -0.003011

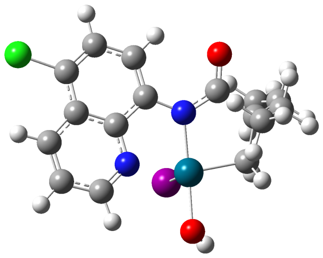

Zero-point correction = 0.312999 (Hartree/Particle)
Thermal correction to Energy = 0.336542
Thermal correction to Enthalpy = 0.337486
Thermal correction to Gibbs Free Energy = 0.259317
Sum of electronic and zero-point Energies = -1479.290721
Sum of electronic and thermal Energies = -1479.267178
Sum of electronic and thermal Enthalpies = -1479.266234
Sum of electronic and thermal Free Energies = -1479.344403

C -1.962254 -1.931926 1.566736
H -1.374150 -2.692124 2.078512
C -3.356736 -2.031057 1.459777
H -3.873685 -2.877840 1.898652
C -4.037952 -1.048948 0.793481
H -5.118372 -1.102427 0.692140
C -3.342372 0.039905 0.226907
C -3.947131 1.095399 -0.495325
C -3.189943 2.101604 -1.028159

H	-3.672503	2.898943	-1.586396
C	-1.796399	2.122983	-0.870648
H	-1.223445	2.934903	-1.298179
C	-1.144359	1.130999	-0.160143
C	-1.933923	0.069365	0.384589
C	1.130353	2.054871	-0.160319
C	2.563627	1.692940	0.255600
C	2.755998	0.221547	0.600488
H	3.149829	0.033362	1.606481
H	3.343348	-0.339408	-0.128147
C	2.873776	2.551045	1.503542
H	3.919452	2.359628	1.784282
H	2.824270	3.603593	1.194557
C	3.527502	2.114470	-0.863511
H	4.551752	1.906235	-0.520917
H	3.454030	3.204986	-0.964379
N	-1.290499	-0.925665	1.055844
N	0.222041	1.067797	0.088298
O	0.865465	3.165539	-0.588558
Pd	0.905015	-0.646103	0.869896
C	1.964345	2.332012	2.695972
H	1.976467	1.292032	3.046037
H	0.923524	2.592529	2.471139
C	3.286043	1.468515	-2.214196
H	2.276528	1.679506	-2.587652
H	3.403844	0.379583	-2.188782
H	3.993911	1.851512	-2.956323
H	2.280905	2.953859	3.539352
I	1.140535	-1.872096	-1.426291
Cl	-5.678475	1.113923	-0.725875
O	1.453682	-2.273442	1.866252
H	2.201936	-2.707751	1.438409

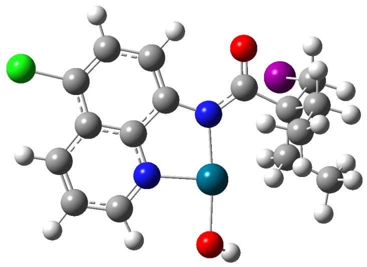

Zero-point correction = 0.313878 (Hartree/Particle)
Thermal correction to Energy = 0.337389
Thermal correction to Enthalpy = 0.338334
Thermal correction to Gibbs Free Energy = 0.259312
Sum of electronic and zero-point Energies = -1479.298831
Sum of electronic and thermal Energies = -1479.275320

Sum of electronic and thermal Enthalpies = -1479.274375
Sum of electronic and thermal Free Energies = -1479.353397

C	2.853876	2.160849	-1.102624
H	2.426486	3.106495	-1.431071
C	4.199435	1.814462	-1.281353
H	4.869234	2.513582	-1.770149
C	4.641466	0.598441	-0.835644
H	5.681341	0.312287	-0.966408
C	3.753173	-0.296605	-0.202340
C	4.101053	-1.573425	0.295463
C	3.161818	-2.365447	0.895779
H	3.448837	-3.343616	1.271936
C	1.830281	-1.944251	1.039770
H	1.113414	-2.594409	1.521760
C	1.421707	-0.706155	0.574630
C	2.407101	0.112017	-0.050273
C	-0.923971	-0.745842	1.210474
C	-2.265255	0.032240	1.177232
C	-3.335892	-0.939859	0.711217
H	-4.338655	-0.513603	0.741645
H	-3.302527	-1.855101	1.300183
C	-2.258703	1.304662	0.317305
H	-1.552471	2.051430	0.790277
H	-1.979823	1.037578	-0.719687
C	-2.623242	0.350347	2.648648
H	-2.635344	-0.602821	3.190304
H	-3.650576	0.735004	2.690854
N	2.013724	1.337544	-0.514197
N	0.146041	-0.147620	0.633025
O	-0.904034	-1.836302	1.768042
Pd	0.064729	1.699203	-0.177536
C	-3.559779	2.089525	0.221598
H	-3.923811	2.410166	1.202399
H	-4.345863	1.502271	-0.263747
C	-1.680179	1.314345	3.345903
H	-1.717840	2.324660	2.922233
H	-0.638783	0.973861	3.299264
H	-1.946666	1.404409	4.403404
H	-3.407491	2.988141	-0.383557
I	-3.091854	-1.641822	-1.353866
Cl	5.744693	-2.146393	0.145107
O	0.190951	3.538313	-0.958295
H	-0.619537	3.714168	-1.449399

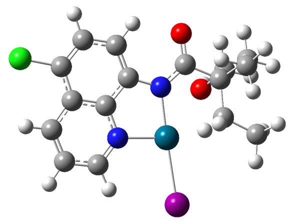

Zero-point correction = 0.316242 (Hartree/Particle)
Thermal correction to Energy = 0.339626
Thermal correction to Enthalpy = 0.340570
Thermal correction to Gibbs Free Energy = 0.261970
Sum of electronic and zero-point Energies = -1479.344949
Sum of electronic and thermal Energies = -1479.321565
Sum of electronic and thermal Enthalpies = -1479.320621
Sum of electronic and thermal Free Energies = -1479.399221

C	-1.542571	-2.459149	0.095032
H	-0.745127	-3.195647	0.120180
C	-2.887780	-2.843510	0.111830
H	-3.135252	-3.898627	0.151903
C	-3.858552	-1.882295	0.075938
H	-4.908536	-2.160153	0.086824
C	-3.502600	-0.519548	0.026039
C	-4.426351	0.550158	-0.009281
C	-3.989780	1.844435	-0.052039
H	-4.713407	2.654496	-0.077482
C	-2.621568	2.152083	-0.064074
H	-2.308199	3.185566	-0.100543
C	-1.665732	1.150777	-0.032508
C	-2.123380	-0.194879	0.012780
C	0.364629	2.496811	-0.157731
C	1.907406	2.490309	-0.084253
C	2.406533	2.927024	-1.456583
H	3.491355	3.110226	-1.414072
H	1.918255	3.877750	-1.716215
C	2.502197	1.127792	0.286992
H	2.108781	0.808116	1.266200
H	2.267918	0.416603	-0.574506
C	2.320473	3.567883	0.929210
H	1.876475	4.512259	0.593020
H	3.407997	3.708985	0.878772
N	-1.178929	-1.191869	0.046507
N	-0.281325	1.303660	-0.019655
O	-0.208922	3.562543	-0.333419
Pd	0.715620	-0.443837	0.013336
C	4.018693	1.021315	0.358448
H	4.424706	1.683447	1.129599

H	4.492371	1.270611	-0.595606
C	1.897376	3.289483	2.359657
H	2.406992	2.418367	2.787396
H	0.817390	3.111701	2.436585
H	2.131018	4.142263	3.004415
H	4.308736	-0.001701	0.615677
I	2.142565	-2.718208	0.022993
Cl	-6.141183	0.219890	0.001942
O	2.095745	1.915837	-2.389607
H	2.417386	2.177445	-3.257759

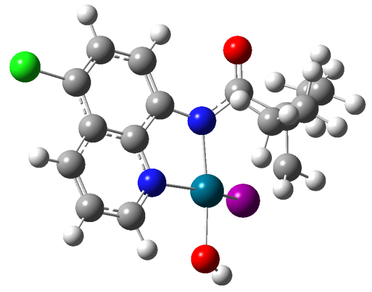

Zero-point correction = 0.312884 (Hartree/Particle)
Thermal correction to Energy = 0.335957
Thermal correction to Enthalpy = 0.336902
Thermal correction to Gibbs Free Energy = 0.260081
Sum of electronic and zero-point Energies = -1479.276896
Sum of electronic and thermal Energies = -1479.253823
Sum of electronic and thermal Enthalpies = -1479.252879
Sum of electronic and thermal Free Energies = -1479.329699

C	-2.033013	-2.274137	1.159502
H	-1.373365	-3.023464	1.594593
C	-3.427490	-2.411493	1.121296
H	-3.890588	-3.287486	1.562963
C	-4.178318	-1.438942	0.517374
H	-5.259857	-1.531605	0.470667
C	-3.554773	-0.309182	-0.053829
C	-4.221782	0.752547	-0.708902
C	-3.519270	1.803609	-1.228860
H	-4.051144	2.603713	-1.736209
C	-2.119306	1.871853	-1.123142
H	-1.595094	2.722329	-1.538092
C	-1.409020	0.870298	-0.490886
C	-2.144788	-0.230452	0.032286
C	0.670545	1.970842	-0.032592
C	1.939599	1.813402	0.820509
C	2.434324	0.423839	1.077796
H	2.025684	-0.122440	1.931873
H	3.513206	0.304436	1.054964

C 1.519730 2.341157 2.237207
H 2.369453 2.190409 2.917611
H 1.412061 3.427209 2.121859
C 3.043063 2.750105 0.313146
H 3.845356 2.768132 1.065026
H 2.608433 3.755977 0.308732
N -1.436104 -1.225542 0.639745
N -0.033920 0.831232 -0.246897
O 0.313385 3.094049 -0.351650
Pd 0.606130 -1.005311 0.357613
C 0.250533 1.773611 2.841286
H 0.280511 0.686513 2.974415
H -0.632145 2.008862 2.235057
C 3.625311 2.463588 -1.057071
H 2.844419 2.366150 -1.821243
H 4.224160 1.548691 -1.078499
H 4.280519 3.285766 -1.363196
H 0.089084 2.211268 3.831542
I 2.814294 -1.127502 -1.113972
Cl -5.961174 0.716394 -0.868069
O 0.897532 -2.827196 1.122994
H 1.773170 -3.159351 0.894565

Imaginary Vibrational Frequency = -160.3659 cm-1

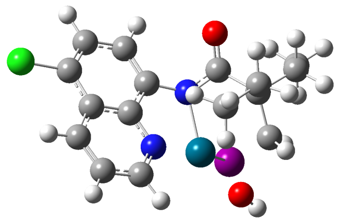

Zero-point correction = 0.313415 (Hartree/Particle)
Thermal correction to Energy = 0.336079
Thermal correction to Enthalpy = 0.337023
Thermal correction to Gibbs Free Energy = 0.261240
Sum of electronic and zero-point Energies = -1479.236999
Sum of electronic and thermal Energies = -1479.214335
Sum of electronic and thermal Enthalpies = -1479.213391
Sum of electronic and thermal Free Energies = -1479.289174

C -1.361948 -1.341896 1.926252
H -0.648971 -1.984870 2.438020
C -2.688147 -1.216369 2.367553
H -3.012587 -1.772446 3.240476
C -3.550542 -0.403216 1.681819
H -4.584280 -0.304249 2.001220
C -3.097156 0.314409 0.555227

C	-3.873940	1.197539	-0.236181
C	-3.326599	1.868801	-1.294842
H	-3.948017	2.537520	-1.883502
C	-1.966825	1.710176	-1.628247
H	-1.542230	2.271203	-2.454657
C	-1.174526	0.859610	-0.902942
C	-1.746065	0.146250	0.181463
C	1.009709	1.691651	-0.581547
C	2.267147	1.384245	0.296384
C	2.840689	-0.009636	0.576034
H	3.270613	-0.075497	1.570431
H	3.485346	-0.428834	-0.189704
C	2.011382	2.078679	1.650518
H	2.960667	2.065085	2.204670
H	1.792172	3.131511	1.445082
C	3.488385	2.091540	-0.360268
H	4.383767	1.883516	0.243692
H	3.298084	3.166190	-0.245818
N	-0.917288	-0.693295	0.872811
N	0.214800	0.669594	-1.057933
O	0.729410	2.863221	-0.743655
Pd	0.835888	-1.051039	-0.251506
C	0.910406	1.480490	2.500491
H	1.074459	0.414751	2.702255
H	-0.073017	1.582565	2.025830
C	3.739791	1.763097	-1.817454
H	2.874989	2.016901	-2.441626
H	3.964148	0.704863	-1.985812
H	4.592236	2.338934	-2.190806
H	0.856191	1.990689	3.467137
I	2.196560	-1.931811	-2.386694
Cl	-5.561772	1.426336	0.141992
O	2.137214	-1.872636	1.138584
H	2.816318	-2.401855	0.695171

Imaginary Vibrational Frequency = -356.8993 cm-1

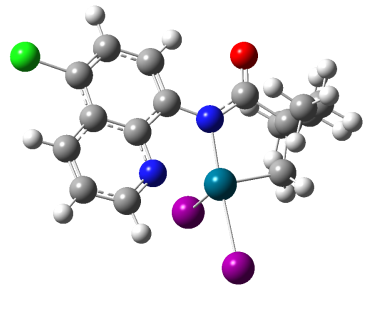

Zero-point correction = 0.300075 (Hartree/Particle)
Thermal correction to Energy = 0.324008

Thermal correction to Enthalpy = 0.324952
Thermal correction to Gibbs Free Energy = 0.243771
Sum of electronic and zero-point Energies = -1414.939969
Sum of electronic and thermal Energies = -1414.916035
Sum of electronic and thermal Enthalpies = -1414.915091
Sum of electronic and thermal Free Energies = -1414.996273

C	1.466880	-2.505587	-0.702041
H	0.616519	-3.170469	-0.848657
C	2.774640	-3.004591	-0.655249
H	2.950698	-4.068451	-0.772980
C	3.807001	-2.130232	-0.450475
H	4.830716	-2.490110	-0.399781
C	3.547484	-0.753388	-0.295997
C	4.540037	0.226916	-0.055507
C	4.200028	1.541309	0.112552
H	4.975249	2.276894	0.306875
C	2.864638	1.957595	0.036826
H	2.624438	3.005031	0.161082
C	1.849044	1.053395	-0.229021
C	2.198025	-0.326724	-0.373194
C	-0.032965	2.623410	-0.440893
C	-1.554859	2.639920	-0.604467
C	-2.223511	1.291700	-0.367597
H	-2.848479	0.949361	-1.199464
H	-2.794390	1.225747	0.560354
C	-1.818605	3.084597	-2.062706
H	-2.908041	3.158712	-2.189923
H	-1.425352	4.104425	-2.168412
C	-2.145678	3.705417	0.331288
H	-3.229560	3.747339	0.149572
H	-1.744130	4.676699	0.014426
N	1.192930	-1.225084	-0.570715
N	0.507021	1.363304	-0.385866
O	0.615222	3.653316	-0.467578
Pd	-0.775047	-0.184462	-0.392568
C	-1.224401	2.203253	-3.143516
H	-1.568408	1.162457	-3.080243
H	-0.129027	2.189551	-3.104890
C	-1.869928	3.506729	1.809311
H	-0.793882	3.484847	2.020643
H	-2.297947	2.576225	2.197930
H	-2.298827	4.326969	2.393833
H	-1.509712	2.570533	-4.134431
Cl	6.217026	-0.239068	0.043787
I	-2.474090	-2.219260	-0.713868
I	-0.876392	-0.385509	2.230634

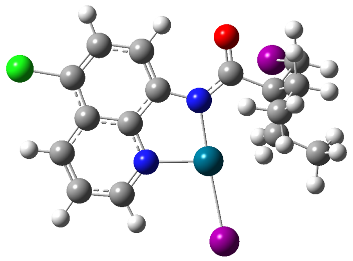

Zero-point correction = 0.301042 (Hartree/Particle)
Thermal correction to Energy = 0.324797
Thermal correction to Enthalpy = 0.325741
Thermal correction to Gibbs Free Energy = 0.243586
Sum of electronic and zero-point Energies = -1414.949152
Sum of electronic and thermal Energies = -1414.925397
Sum of electronic and thermal Enthalpies = -1414.924453
Sum of electronic and thermal Free Energies = -1415.006608

C	2.893853	1.747867	-0.677969
H	2.498158	2.742757	-0.857796
C	4.239453	1.447778	-0.914925
H	4.896531	2.226185	-1.286821
C	4.695622	0.182608	-0.671738
H	5.737255	-0.069886	-0.847601
C	3.814251	-0.806658	-0.191851
C	4.182930	-2.143179	0.086187
C	3.258207	-3.039004	0.543603
H	3.558665	-4.062004	0.752070
C	1.922078	-2.665492	0.750076
H	1.214225	-3.396898	1.113207
C	1.497642	-1.372654	0.497164
C	2.462432	-0.441965	0.024555
C	-0.846386	-1.566444	1.131408
C	-2.209730	-0.841205	1.209816
C	-3.257538	-1.787957	0.648487
H	-4.272001	-1.402369	0.749256
H	-3.182448	-2.764493	1.124548
C	-2.250053	0.513742	0.497259
H	-1.570640	1.231372	1.061889
H	-1.967820	0.380106	-0.560564
C	-2.551071	-0.704267	2.713438
H	-2.518676	-1.712405	3.143376
H	-3.591348	-0.368317	2.811598
N	2.045935	0.842851	-0.226997
N	0.207740	-0.863027	0.638382
O	-0.777418	-2.719736	1.532044
Pd	0.064728	1.090250	0.165532
C	-3.568359	1.274467	0.511625

H	-3.925687	1.467094	1.527525
H	-4.344398	0.725299	-0.031030
C	-1.634674	0.211104	3.505522
H	-1.713160	1.260973	3.198710
H	-0.582033	-0.082789	3.415736
H	-1.893513	0.173365	4.567968
H	-3.444441	2.240419	0.013519
I	-0.218736	3.705967	-0.359759
I	-3.035768	-2.228586	-1.488568
Cl	5.833189	-2.658494	-0.156512

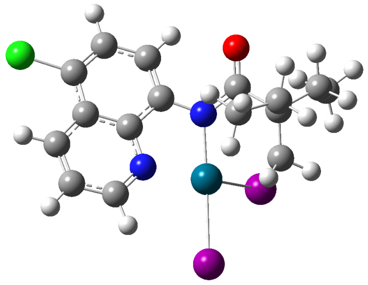

Zero-point correction = 0.299910 (Hartree/Particle)
Thermal correction to Energy = 0.323253
Thermal correction to Enthalpy = 0.324197
Thermal correction to Gibbs Free Energy = 0.245111
Sum of electronic and zero-point Energies = -1414.923986
Sum of electronic and thermal Energies = -1414.900643
Sum of electronic and thermal Enthalpies = -1414.899699
Sum of electronic and thermal Free Energies = -1414.978785

C	1.758917	-2.450278	-0.526742
H	0.967464	-3.164505	-0.740345
C	3.102991	-2.844713	-0.515228
H	3.354724	-3.876559	-0.735552
C	4.067913	-1.924039	-0.214486
H	5.114887	-2.212032	-0.186900
C	3.706839	-0.588817	0.056548
C	4.617376	0.448014	0.372860
C	4.177278	1.720548	0.607606
H	4.892388	2.499170	0.857229
C	2.812390	2.039758	0.527690
H	2.491440	3.058718	0.700046
C	1.879154	1.071970	0.218729
C	2.330776	-0.257911	0.006937
C	0.027488	2.399208	-0.546590
C	-1.371078	2.287064	-1.169578
C	-2.173712	1.070874	-0.830851
H	-2.082217	0.190904	-1.473165
H	-3.220763	1.241903	-0.599586

C -1.108402 2.180514 -2.711386
H -2.081059 2.059440 -3.208200
H -0.736017 3.169162 -3.008641
C -2.155966 3.587529 -0.947148
H -3.064023 3.542082 -1.565782
H -1.542242 4.390768 -1.370224
N 1.387366 -1.213554 -0.266616
N 0.509449 1.271142 0.037466
O 0.665182 3.427312 -0.701167
Pd -0.555417 -0.455692 0.118777
C -0.135958 1.119288 -3.186265
H -0.459724 0.096495 -2.961428
H 0.863455 1.253691 -2.755407
C -2.519957 3.951302 0.478623
H -1.642129 3.960586 1.135717
H -3.254731 3.269606 0.916333
H -2.956972 4.954858 0.505012
H -0.030615 1.183596 -4.273823
Cl 6.324677 0.098670 0.476599
I -1.866348 -2.798662 -0.051082
I -2.318444 0.521965 1.841827

Imaginary Vibrational Frequency = -160.4599 cm-1

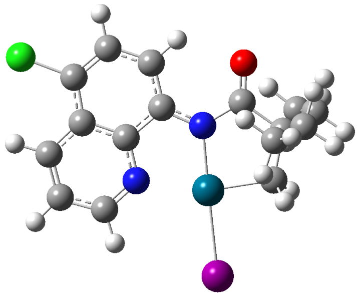

Zero-point correction = 0.298506 (Hartree/Particle)
Thermal correction to Energy = 0.320584
Thermal correction to Enthalpy = 0.321529
Thermal correction to Gibbs Free Energy = 0.242193
Sum of electronic and zero-point Energies = -1403.533063
Sum of electronic and thermal Energies = -1403.510984
Sum of electronic and thermal Enthalpies = -1403.510040
Sum of electronic and thermal Free Energies = -1403.589376

C 1.319736 -2.606882 0.022530
H 0.473326 -3.292302 0.027229
C 2.635387 -3.080966 0.021343
H 2.825315 -4.148852 0.025699
C 3.663314 -2.175907 0.013847
H 4.695390 -2.513708 0.012056

C	3.379322	-0.797215	0.008101
C	4.369604	0.222611	-0.001805
C	4.019498	1.555365	-0.008476
H	4.798527	2.311570	-0.016888
C	2.687290	1.945841	-0.004285
H	2.438293	2.997896	-0.008671
C	1.652746	0.996025	0.006825
C	2.022730	-0.403002	0.011194
C	-0.262697	2.541920	0.009772
C	-1.785701	2.526966	-0.015781
C	-2.398834	1.119000	0.010855
H	-3.034149	0.927380	-0.860633
H	-3.000711	0.944094	0.909157
C	-2.211234	3.271261	-1.295452
H	-3.309945	3.311485	-1.299270
H	-1.862916	4.310527	-1.221458
C	-2.262980	3.339854	1.201652
H	-3.361586	3.365497	1.166573
H	-1.924283	4.377584	1.078512
N	1.026388	-1.320776	0.017519
N	0.320109	1.253725	0.012135
O	0.376549	3.567366	0.022358
Pd	-0.940538	-0.294730	0.011181
C	-1.703453	2.657954	-2.587607
H	-2.017063	1.614369	-2.705083
H	-0.607513	2.678364	-2.643235
C	-1.791921	2.810057	2.543849
H	-0.699382	2.854259	2.635950
H	-2.092288	1.769322	2.710802
H	-2.206450	3.403082	3.365232
H	-2.078251	3.210175	-3.455207
I	-2.659826	-2.316337	-0.002346
Cl	6.044970	-0.200246	-0.006859

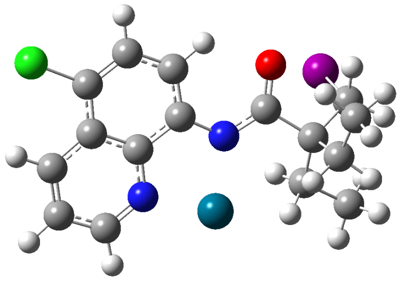

Zero-point correction = 0.299063 (Hartree/Particle)
Thermal correction to Energy = 0.321117
Thermal correction to Enthalpy = 0.322061
Thermal correction to Gibbs Free Energy = 0.244135
Sum of electronic and zero-point Energies = -1403.495709
Sum of electronic and thermal Energie s= -1403.473654

Sum of electronic and thermal Enthalpies = -1403.472710
Sum of electronic and thermal Free Energies = -1403.550636

C	3.393563	2.289486	-0.804572
H	3.141086	3.323981	-1.027770
C	4.705773	1.821891	-0.945657
H	5.487589	2.495445	-1.280954
C	4.966132	0.512801	-0.653708
H	5.972883	0.116963	-0.753320
C	3.929515	-0.340197	-0.219293
C	4.116888	-1.703133	0.099623
C	3.064529	-2.467281	0.514228
H	3.222522	-3.515425	0.755388
C	1.778026	-1.926314	0.635094
H	0.970303	-2.563686	0.963522
C	1.504404	-0.594413	0.344200
C	2.620594	0.206104	-0.096415
C	-0.856156	-0.599414	0.821101
C	-2.122742	0.280768	1.022776
C	-3.344235	-0.594796	0.827033
H	-4.276222	-0.088687	1.081644
H	-3.259595	-1.530373	1.371771
C	-2.202272	1.546995	0.149044
H	-1.505188	2.308158	0.581345
H	-1.893018	1.310699	-0.879595
C	-2.104212	0.736932	2.509888
H	-2.948955	1.421867	2.665914
H	-1.197483	1.345132	2.640834
N	2.406804	1.518367	-0.400891
N	0.275101	0.037904	0.446753
O	-0.956687	-1.798171	1.084117
Pd	0.364803	2.089441	-0.165276
C	-3.548939	2.255464	0.093549
H	-3.933262	2.504641	1.088215
H	-4.307054	1.657884	-0.422513
C	-2.143364	-0.342968	3.575217
H	-1.322689	-1.058177	3.468145
H	-3.080623	-0.910101	3.557148
H	-2.063632	0.112066	4.567971
H	-3.450724	3.195002	-0.458756
I	-3.641738	-1.281716	-1.243652
Cl	5.706490	-2.429930	-0.037022

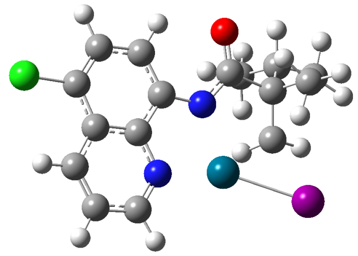

Zero-point correction = 0.298468 (Hartree/Particle)
Thermal correction to Energy = 0.320118
Thermal correction to Enthalpy = 0.321063
Thermal correction to Gibbs Free Energy = 0.245372
Sum of electronic and zero-point Energies = -1403.486050
Sum of electronic and thermal Energies = -1403.464400
Sum of electronic and thermal Enthalpies = -1403.463455
Sum of electronic and thermal Free Energies = -1403.539146

C	-1.895259	-2.694530	-0.447713
H	-1.161441	-3.492723	-0.537747
C	-3.268177	-2.962449	-0.530032
H	-3.610096	-3.980256	-0.686439
C	-4.149037	-1.924317	-0.407433
H	-5.219705	-2.100664	-0.463912
C	-3.673781	-0.611543	-0.205595
C	-4.502265	0.525420	-0.069003
C	-3.958215	1.763547	0.125085
H	-4.611603	2.626017	0.227805
C	-2.569534	1.945704	0.195629
H	-2.169293	2.937906	0.352490
C	-1.692234	0.878187	0.068451
C	-2.268314	-0.419685	-0.133307
C	0.402163	2.048152	0.326072
C	1.932400	1.903778	0.240936
C	2.455436	0.703058	-0.496471
H	3.524176	0.779930	-0.680420
H	1.951671	0.429195	-1.426235
C	2.531868	2.070616	1.654447
H	3.578519	1.736118	1.619605
H	2.569292	3.146394	1.867922
C	2.459938	3.094368	-0.614166
H	3.555150	3.120809	-0.530149
H	2.082043	3.993349	-0.114936
N	-1.422702	-1.481015	-0.257669
N	-0.309978	0.928576	0.089238
O	-0.051131	3.153103	0.619193
Pd	0.648416	-0.965141	-0.032850
C	1.788226	1.385844	2.787194

H	1.656962	0.311423	2.625995
H	0.793100	1.823142	2.926857
C	2.032609	3.107864	-2.070693
H	0.946407	2.990114	-2.170402
H	2.508912	2.319046	-2.662873
H	2.298810	4.061690	-2.536864
H	2.334081	1.511557	3.728266
I	3.193698	-1.440854	0.694846
Cl	-6.243827	0.353921	-0.145160

Imaginary Vibrational Frequency = -172.5706 cm-1

Optimisation and Control Experiments

Importance of Light, Oxidant and Halogenation Source

Table S4: Summary of Control Experiments

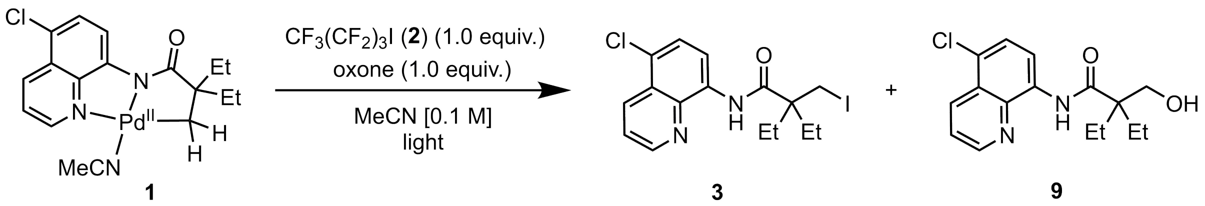

**Deviations from standard conditions**	Conversion of **1**	Yield **3** [%]	Yield **9** [%]
None	> 99	99	trace
No light	10	N.D.	5
No iodination reagent	20	N.D.	15
No oxone	> 99	55	N.D.
No oxone, no light	N.R.	N.D.	N.D.

Solvent Effect

Table S5: Solvent screen

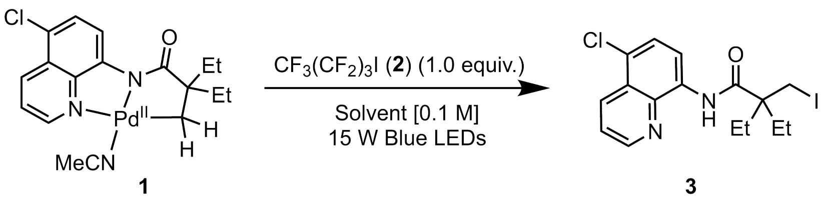

Solvent	Yield **3** [%]	Unreacted palladacycle **1** [%]
MeCN	55 (54 isolated)	ND
$CHCl_3$	50	ND
Acetone	37	21
1,2-DCE	38	ND
Chlorobenzene	41	ND
EtOAc	37	ND
DMF	34	ND
MeOH	43	ND

Homolysis of Perfluorobutyl Iodide 2 Under Blue Light Irradiation

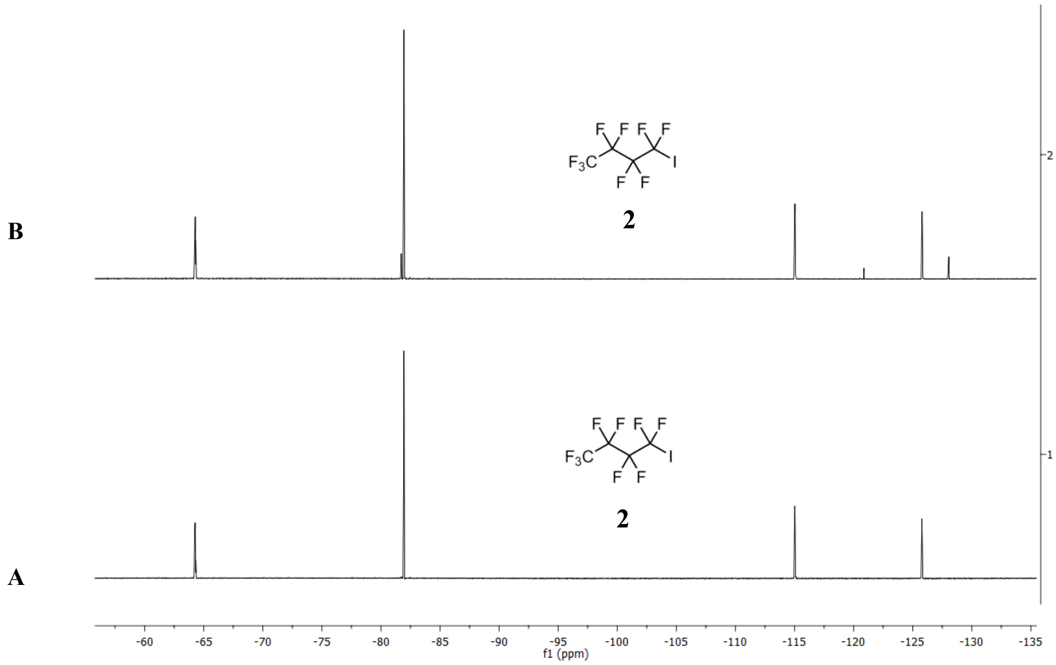

Figure S26: ^{19}F NMR (CD_3CN, 376 MHz) spectrum of (**A**) Perfluorobutyl iodide in CD_3CN (**B**) Perfluorobutyl iodide in CD_3CN after irradiation with 15 W blue LEDs for 24 hours, in a standard NMR tube.

Stability of Palladacycle in the Presence of Iodinated Product 3 Under Blue Light Irradiation

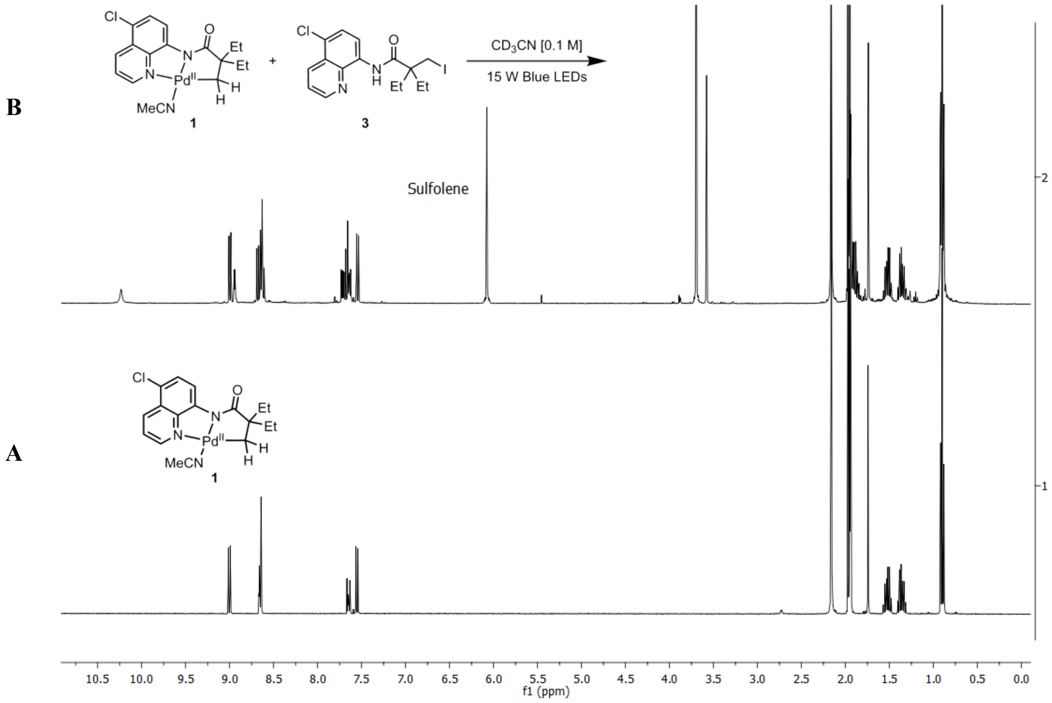

Figure S27: ^{1}H NMR (CD_3CN, 400 MHz) spectrum of (**A**) Palladacycle **1** in CD_3CN (**B**) A reaction mixture containing palladacycle **1** and product **3** in CD_3CN, and sulfolene as an internal standard, after irradiation with 15 W blue LEDs for 24 hours; no decomposition of either **1** or **3** is evident;

Synthesis and Characterization

5-Chloro-8-aminoquinoline

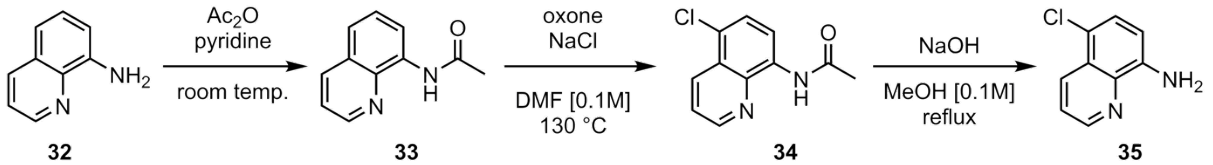

Pyridine (3 drops) was added to a solution of 8-aminoquinoline **32** (3.0 g, 20.8 mmol) in acetic anhydride (26 mL). The reaction mixture was stirred at room temperature for 12 hours. It was then quenched with saturated aqueous $NaHCO_3$ solution, and the mixture was left to stir for 1 hour. It was extracted with CH_2Cl_2 (3×), and the combined organic extracts were washed with brine (3×) and dried ($MgSO_4$). The solvent was removed under reduced pressure to give *N*-(quinolin-8-yl)acetamide **33** as a yellow solid, which was used without further purification.

Oxone (8.0 g, 26 mmol, 1.5 equiv.) and NaCl (4.1 g, 70 mmol, 4.0 equiv.) were added to a solution of *N*-(quinolin-8-yl)acetamide (3.2 g, 17.5 mmol) in DMF (170 mL). The reaction flask was sealed and heated at 130 °C for 12 hours. After cooling to room temperature, the reaction mixture was diluted with water and extracted with EtOAc (3×). The combined organic extracts were washed with saturated aqueous LiCl solution (3×) and dried ($MgSO_4$). The solvent was removed under reduced pressure to yield **34** a pale brown solid, which was used without further purification.

N-(5-Chloroquinolin-8-yl)acetamide **34** (2.7 g, 12.2 mmol) was added to a solution of NaOH (4.8 g, 122 mmol, 10 equiv.) in MeOH (120 mL) and the reaction mixture was heated at reflux for 12 hours. The reaction mixture was cooled to room temperature, extracted with CH_2Cl_2 (3×) and dried ($MgSO_4$). The solvent was removed under reduced pressure to give a yellow solid. Purification by flash chromatography on deactivated silica, eluting 30% EtOAc/Hexane gave the title compound **35** as a bright yellow solid (1.7 g, 49% yield over 3 steps).

^{1}H NMR ($CDCl_3$, 400 MHz): δ = 8.79 (dd, *J* = 4.2, 1.6 Hz, 1H), 8.47 (dd, *J* = 8.5, 1.6 Hz, 1H), 7.49 (dd, *J* =8.5, 4.2 Hz, 1H), 7.40 (d, *J* = 8.1 Hz, 1H), 6.83 (d, *J* = 8.4 Hz, 1H), 5.02 (br s, 2H).

^{13}C NMR ($CDCl_3$, 100 MHz): δ = 147.9, 143.4, 138.9, 133.0, 127.4, 126.7, 122.2, 118.2, 109.6.

HRMS (ESI) *m/z* calc. for $C_{10}H_7ClN_2$ $[M+H]^+$ 179.0376 found 179.0370.

General Procedure for the Preparation of *N*-(5-Chloroquinolin-8-yl) Amides

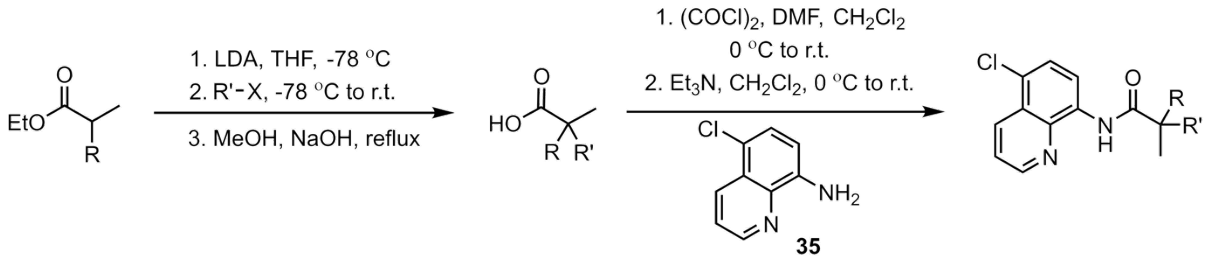

A solution of LDA in THF (C = 2.0 M, 15 mL, 30 mmol, 1.2 equiv.) was added dropwise to a solution of carboxylate ester (3.0 g, 23 mmol, 1.0 equiv.) in THF at -78 °C. The solution was stirred at -78 °C for 3 h. Alkyl halide (2.0 equiv.) was then added dropwise at -78 °C, and the reaction mixture was stirred at room temperature overnight. It was quenched with water and extracted with Et_2O (3×). The combined organic layers were washed with brine (3×) and dried ($MgSO_4$). The solvent was removed under reduced pressure, and the residue was taken up in MeOH (50 mL) and combined with solid NaOH (9.2 g, 230 mmol, 10 equiv.). The reaction mixture was heated at reflux overnight. After cooling to room temperature, the solution was diluted with water and washed with Et_2O (3×). The aqueous layer was acidified to pH 1 with 3 M aqueous HCl solution and extracted with CH_2Cl_2 (3×), and dried ($MgSO_4$). The solvent was removed under reduced pressure to give the crude carboxylic acid, which was used directly for the next step without further purification.

Oxalyl chloride (5.04 mmol, 0.43 mL, 1.8 equiv.) was added dropwise to a solution of the carboxylic acid (4.2 mmol, 1.5 equiv.) in CH_2Cl_2 (5 mL) and DMF (3 drops) at 0 °C. The reaction mixture was stirred at room temperature overnight. The solvent was removed under reduced pressure, and the residue dried under vacuum, taken up in CH_2Cl_2 (5 mL) and added dropwise to a solution of 5-chloro-8-aminoquinoline **35** (0.5 g, 2.8 mmol, 1.0 equiv.) and Et_3N (0.6 mL, 4.2 mmol, 1.5 equiv.) in CH_2Cl_2 (5 mL) at 0 °C. The reaction mixture was stirred at room temperature for 5 h. It was diluted with CH_2Cl_2, washed with brine (3×) and dried ($MgSO_4$). The solvent was removed under reduced pressure, and the

residue was purified by flash chromatography on deactivated silica, eluting with EtOAc/*n*-pentane), to give the *N*-(5-chloroquinolin-8-yl)amides.

Substrate Characterisation Data

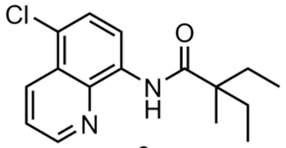

3a

***N*-(5-Chloroquinolin-8-yl)-2-ethyl-2-methylbutanamide (3a):** obtained as a white solid (66%)

^{1}H NMR (CDCl$_3$, 400 MHz): δ = 10.16 (br s, 1H), 8.86 (dd, *J* = 4.2, 1.3 Hz, 1H), 8.77 (d, *J* = 8.4 Hz, 1H), 8.55 (dd, *J* = 8.5, 1.2 Hz, 1H), 7.59 (d, *J* = 8.4 Hz, 1H), overlapping with 7.56 (dd, *J* = 8.5, 4.2 Hz, 1H), 1.92 – 1.83 (m, 2H), 1.67 – 1.58 (m, 2H), 1.34 (s, 3H), 0.93 (t, *J* = 7.5 Hz, 6H).

^{13}C NMR (CDCl$_3$, 100 MHz): δ = 176.2, 148.8, 139.5, 134.1, 133.5, 127.4, 126.1, 124.0, 122.4, 116.2, 48.1, 32.6, 20.3, 9.1.

HRMS (ESI) *m/z* calc. for $C_{16}H_{20}ClN_2O$ [M+H]$^+$ 291.1264 found 291.1257.

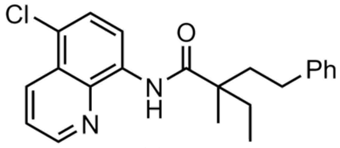

13a

***N*-(5-Chloroquinolin-8-yl)-2-ethyl-2-methyl-4-phenylbutanamide (13a):** obtained as a colourless oil (55%)

^{1}H NMR (CDCl$_3$, 400 MHz): δ = 10.24 (br s, 1H), 8.87 (dd, *J* = 4.2, 1.5 Hz, 1H), 8.78 (d, *J* = 8.3 Hz, 1H), 8.57 (dd, *J* = 8.6, 1.5 Hz, 1H), 7.61 (d, *J* = 8.4 Hz, 1H), 7.58 (dd, *J* = 8.6, 4.2 Hz, 1H), 7.26 – 7.13 (m, 5H), 2.74 – 2.57 (m, 2H), 2.20 – 2.21 (m, 1H), 1.98 – 1.83 (m, 2H), 1.76 – 1.68 (m, 2H), 1.46 (s, 3H), 0.98 (t, *J* = 7.5 Hz, 3H).

^{13}C NMR (CDCl$_3$, 100 MHz): δ = 175.7, 148.9, 142.4, 139.5, 133.9, 133.5, 128.5, 128.4, 127.4, 126.1, 126.0, 125.9, 124.2, 122.4, 116.4, 47.9, 42.2, 33.1, 31.3, 20.9, 9.1.

HRMS (ESI) *m/z* calc. for $C_{22}H_{24}ClN_2O$ [M+H]$^+$ 367.1577 found 367.1572.

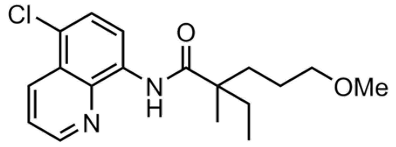

14a

***N*-(5-Chloroquinolin-8-yl)-2-ethyl-5-methoxy-2-methylpentanamide (14a):** obtained as a yellow oil (70%)

^{1}H NMR ($CDCl_3$, 400 MHz): δ = 10.17 (br s, 1H), 8.85 (dd, *J* = 4.2, 1.6 Hz, 1H), 8.75 (d, *J* = 8.4 Hz, 1H), 8.55 (dd, *J* = 8.5, 1.6 Hz, 1H), 7.58 (d, *J* = 8.4 Hz, 1H), 7.56 (dd, *J* = 8.5, 4.2 Hz, 1H), 3.40 – 3.35 (m, 2H), 3.28 (s, 3H), 1.92 – 1.83 (m, 2H), 1.72 – 1.55 (m, 4H), 1.37 (s, 3H), 0.94 (t, *J* = 7.5, 3H).

^{13}C NMR ($CDCl_3$, 100 MHz): δ = 176.0, 148.8, 139.5, 134.0, 133.5, 127.4, 126.0, 124.1, 122.4, 116.3, 73.1, 58.6, 47.5, 36.4, 33.0, 25.0, 20.7, 9.1.

HRMS (ESI) *m/z* calc. for $C_{18}H_{23}ClN_2O_2$ $[M]^+$ 334.1448 found 334.1444.

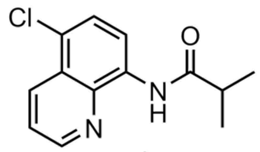

15a

***N*-(5-Chloroquinolin-8-yl)isobutyramide (15a):** obtained as a white solid (80%)

^{1}H NMR ($CDCl_3$, 400 MHz): δ = 9.85 (br s, 1H), 8.84 (dd, *J* = 4.2, 1.4 Hz, 1H), 8.74 (d, *J* = 8.4 Hz, 1H), 8.56 (dd, *J* = 8.5, 1.4 Hz, 1H), 7.59 (d, *J* = 8.4 Hz), overlapping with 7.57 (dd, *J* = 8.5, 4.2 Hz, 1H), 2.76 (septet, *J* = 6.9 Hz, 1H), 1.35 (d, *J* = 6.9 Hz, 6H).

^{13}C NMR ($CDCl_3$, 100 MHz): δ = 175.9, 148.7, 139.1, 134.0, 133.6, 127.4, 126.0, 124.2, 122.4, 116.5, 37.3, 19.8.

HRMS (ESI) *m/z* calc. for $C_{13}H_{14}ClN_2O$ $[M+H]^+$ 249.0795 found 249.0804.

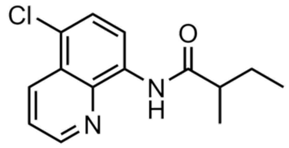

16a

***N*-(5-Chloroquinolin-8-yl)-2-methylbutanamide (16a):** obtained as a white solid (90%)

^{1}H NMR ($CDCl_3$, 400 MHz): δ = 9.82 (br s, 1H), 8.85 (dd, *J* = 4.2, 1.4 Hz, 1H), 8.76 (d, *J* = 8.4 Hz, 1H), 8.56 (dd, *J* = 8.5, 1.3 Hz, 1H), 7.59 (d, *J* = 8.4 Hz, 1H), overlapping with 7.57 (dd, J = 8.5, 4.2 Hz, 1H), 2.56 – 2.50 (m, 1H), 1.90 – 1.83 (m, 1H), 1.65 – 1.58 (m, 1H), 1.32 (d, *J* = 6.9 Hz, 3H), 1.01 (t, *J* = 7.4 Hz, 3H).

^{13}C NMR ($CDCl_3$, 100 MHz): δ = 175.5, 148.7, 139.1, 134.0, 133.6, 127.4, 126.0, 124.2, 122.4, 116.5, 44.7, 27.6, 17.7, 12.1.

HRMS (ESI) *m/z* calc. for $C_{14}H_{16}ClN_2O$ $[M+H]^+$ 263.0951 found 263.1005.

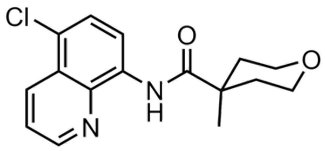

17a

***N*-(5-Chloroquinolin-8-yl)-4-methyltetrahydro-2H-pyran-4-carboxamide (17a):** obtained as a white solid (83%)

^{1}H NMR ($CDCl_3$, 400 MHz): δ = 10.18 (br s, 1H), 8.85 (dd, *J* = 4.2, 1.3 Hz, 1H), 8.73 (d, *J* = 8.4 Hz, 1H), 8.57 (dd, *J* = 8.5, 1.4 Hz, 1H), 7.61 (d, *J* = 8.4 Hz, 1H), overlapping with 7.58 (dd, *J* = 8.5, 4.2 Hz, 1H), 3.89 – 3.80 (m, 2H), 3.73 (ddd, *J* = 11.7, 8.5, 3.0 Hz, 2H), 2.26 (dt, *J* = 13.9, 3.8 Hz, 2H), 1.74 (ddd, *J* = 13.1, 8.5, 3.8 Hz, 2H), 1.45 (s, 3H).

^{13}C NMR ($CDCl_3$, 100 MHz): δ = 175.4, 148.9, 139.3, 133.8, 133.6, 127.4, 126.1, 124.4, 122.5, 116.4, 65.0, 42.1, 35.6, 26.1.

HRMS (ESI) *m/z* calc. for $C_{16}H_{18}ClN_2O_2$ $[M+H]^+$ 305.1057 found 305.1045

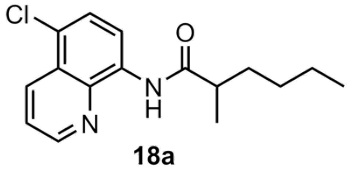

18a

***N*-(5-Chloroquinolin-8-yl)-2-methylhexanamide (18a):** obtained as a colourless oil (88%)

^{1}H NMR ($CDCl_3$, 400 MHz): δ = 9.81 (br s, 1H), 8.85 (dd, *J* = 4.2, 1.5 Hz, 1H), 8.76 (d, *J* = 8.4 Hz, 1H), 8.56 (dd, *J* = 8.5, 1.4 Hz, 1H), 7.59 (d, *J* = 8.4 Hz, 1H), overlapping with 7.57 (dd, *J* = 8.5, 4.2 Hz, 1H), 2.63 – 2.55 (m, 1H), 1.90 – 1.76 (m, 1H), 1.61 – 1.49 (m, 1H), 1.41 – 1.34 (m, 4H), 1.32 (d, *J* = 6.9 Hz, 3H), 0.89 (t, *J* = 7.0 Hz, 3H).

^{13}C NMR ($CDCl_3$, 100 MHz): δ = 175.7, 148.7, 139.1, 134.0, 133.6, 127.4, 126.0, 124.2, 122.4, 116.5, 43.2, 34.3, 29.8, 22.9, 18.1, 14.1.

HRMS (ESI) *m/z* calc. for $C_{16}H_{20}ClN_2O$ $[M+H]^+$ 291.1264 found 291.1267

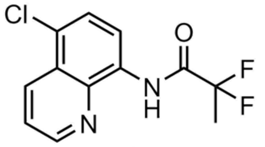

19a

***N*-(5-Chloroquinolin-8-yl)-2,2-difluoropropanamide (19a):** obtained as a white solid (76%)

^{1}H NMR ($CDCl_3$, 400 MHz): δ = 10.66 (br s, 1H), 8.90 (dd, *J* = 4.2, 1.3 Hz, 1H), 8.69 (d, *J* = 8.4 Hz, 1H), 8.58 (dd, *J* = 8.5, 1.3 Hz, 1H), 7.63 (d, *J* = 8.4 Hz, 1H), overlapping with 7.61 (dd, *J* = 8.4, 4.2 Hz, 1H), 1.97 (t, *J* = 19.2 Hz, 3H).

^{19}F NMR ($CDCl_3$, 376 MHz): δ = -98.78 (qd, *J* = 19.1, 2.6 Hz).

^{13}C NMR ($CDCl_3$, 100 MHz): δ = 162.7 (t, *J* = 29.1 Hz) 149.4, 139.3, 133.6, 132.2, 127.1, 126.1, 122.8, 117.4 (t, *J* = 249.6 Hz), 117.2, 21.2 (t, *J* = 25.5 Hz).

HRMS (ESI) *m/z* calc. for $C_{12}H_{10}ClF_2N_2O$ $[M+H]^+$ 271.0450 found 271.0448

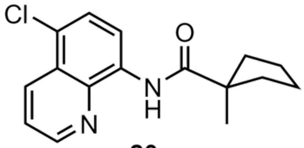

20a

***N*-(5-Chloroquinolin-8-yl)-1-methylcyclopentanecarboxamide (20a):** obtained as a colourless solid (77%)

^{1}H NMR ($CDCl_3$, 400 MHz): δ = 10.13 (br s, 1H), 8.85 (dd, *J* = 4.2, 1.6 Hz, 1H), 8.73 (d, *J* = 8.4 Hz, 1H), 8.55 (dd, *J* = 8.5, 1.6 Hz, 1H), 7.58 (d, *J* = 8.4 Hz, 1H), 7.56 (dd, *J* = 8.5, 4.2 Hz, 1H), 2.29 – 2.26 (m, 2H), 1.84 – 1.79 (m, 4H), 1.72 – 1.66 (m, 2H), 1.47 (s, 3H).

^{13}C NMR ($CDCl_3$, 100 MHz): δ = 177.6, 148.8, 139.4, 134.3, 133.5, 127.4, 126.0, 124.0, 122.4, 116.2, 51.6, 38.1, 25.7, 25.1.

HRMS (ESI) *m/z* calc. for $C_{16}H_{18}ClN_2O$ $[M+H]^+$ 289.1108 found 289.1101

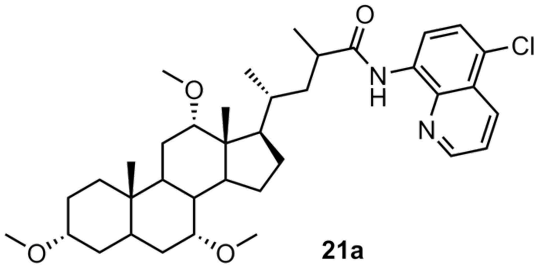

21a

(4R)-*N*-(5-Chloroquinolin-8-yl)-2-methyl-4-((3R,7R,10S,12S,13R,17R)-3,7,12-trimethoxy-10,13-dimethylhexadecahydro-1H-cyclopenta[a]phenanthren-17-yl)pentanamide (21a): obtained as a white solid

^{1}H NMR ($CDCl_3$, 400 MHz): δ = 9.82 (br s, 1H), 9.87 (dd, *J* = 4.4, 1.6 Hz, 1H), 8.73 (d, *J* = 8.4 Hz, 1H), 8.62 (dd, *J* = 8.8, 1.6 Hz, 1H), 7.60 – 7.55 (m, 2H), 3.93 (m, 1H), 3.32 (s, 3H), 3.25 (s, 3H), 3.20 (s, 3H), 3.14 – 3.13 (m,1H), 3.01 – 2.95 (m, 1H), 2.67 – 2.62 (m, 1H), 2.23 – 1.87 (m, 6H), 1.84 – 1.66 (m, 7H), 1.61 -1.43 (m, 5H), 1.34 – 1.56 (m, 7H), overlapping with 1.27 (d, *J* = 6.8 Hz, 3H), 1.07 – 0.96 (m, 1H) overlapping with 1.00 (d, *J* = 6.0 Hz, 3H), 0.93 – 0.83 (m, 5H), 0.69 (s, 3H).

^{13}C NMR ($CDCl_3$, 100 MHz): δ = 176.3, 148.7, 139.2, 134.1, 133.5, 127.4, 126.0, 124.1, 122.4, 116.5, 82.1, 80.8, 77.1, 56.0, 55.8, 55.3, 47.4, 46.4, 42.8, 42.1, 40.4, 39.8, 35.4, 35.0, 34.5, 33.9, 28.1, 27.9, 27.8, 26.9, 23.3, 23.0, 22.1, 17.7, 17.0, 14.3, 12.7.

HRMS (ESI) *m/z* calc. for $C_{37}H_{54}ClN_2O_4$ $[M+H]^+$ 625.3772 found 625.3765

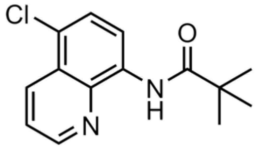

26a

***N*-(5-Chloroquinolin-8-yl)pivalamide (26a):** obtained as a colorless solid (97% yield)

^{1}H NMR ($CDCl_3$, 400 MHz): δ = 10.21 (br s, 1H), 8.85 (dd, *J* = 4.2, 1.6 Hz, 1H), 8.74 (d, *J* = 8.4 Hz, 1H), 8.55 (dd, *J* = 8.5, 1.6 Hz, 1H), 7.58 (d, *J* = 8.4 Hz, 1H), 7.56 (dd, *J* = 8.5, 4.2 Hz, 1H), 1.42 (s, 9H).

^{13}C NMR ($CDCl_3$, 100 MHz): δ = 177.4, 148.8, 139.5, 134.1, 133.5, 127.4, 126.0, 124.1, 122.4, 116.3, 40.5, 27.8.

HRMS (ESI) *m/z* calc. for $C_{14}H_{16}ClN_2O$ $[M+H]^+$ 263.0951, found 263.0946.

General Procedure for the Preparation of the Charge-Tag Complex

N-(5-(4-(Dimethylamino)phenyl)quinolin-8-yl)-2-ethyl-2-methylbutanamide (36)

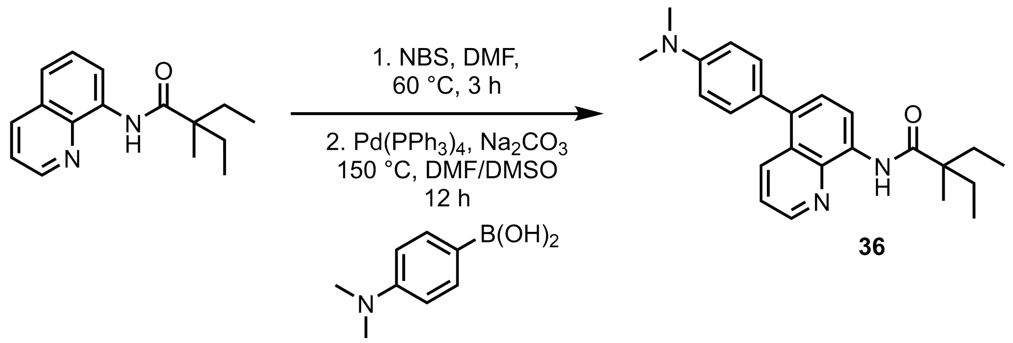

N-Bromosuccinimide (0.23 g, 1.29 mmol, 1.1 equiv.) was added in one portion to a solution of 2-ethyl-2-methyl-*N*-(quinolin-8-yl)butanamide (0.3 g, 1.17 mmol, 1.0 equiv.) in anhydrous DMF (5 mL) under nitrogen, and the reaction mixture was heated at 60 °C for 3 hours. After cooling to room temperature, $Pd(PPh_3)_4$ (0.14 g, 0.12 mmol, 0.1 equiv.), 4-(dimethylamino)phenyl)boronic acid (0.39 g, 2.34 mmol, 2.0 equiv.), sodium carbonate (0.25 g, 2.34 mmol, 2.0 equiv.) and anhydrous DMSO (5 mL) were added to the reaction mixture, and it was heated at 150 °C for 12 hours. The suspension was filtered through a plug of Celite with ethyl acetate and the filtrate was washed with brine (5×) and dried ($MgSO_4$). Purification by flash chromatography on deactivated silica, eluting with 10% EtOAc/*n*-hexane, gave the title compound **36** as a yellow solid (0.35 g, 80% yield).

^{1}H NMR ($CDCl_3$, 400 MHz): δ = 10.30 (br s, 1H), 8.86 (d, *J* = 8.0 Hz, 1H), 8.82 (dd, *J* = 4.0, 1.2 Hz, 1H), 8.38 (dd, *J* = 8.8, 1.2 Hz, 1H), 7.49 (d, *J* = 8.0 Hz, 1H), 7.40 (dd, *J* = 8.4, 4.0 Hz, 1H), 7.35 (d, *J* = 8.8 Hz, 2H), 6.85 (d, *J* = 8.8 Hz, 2H), 3.03 (s, 6H), 1.96 – 1.87 (m, 2H), 1.69 – 1.60 (m, 2H), 1.38 (s, 3H), 0.96 (t, *J* = 7.2, 3H).

^{13}C NMR ($CDCl_3$, 100 MHz): δ = 176.0, 150.0, 148.0, 139.1, 135.2, 134.4, 133.4, 130.9, 127.7, 127.3, 126.7, 121.2, 116.0, 112.5, 48.1, 40.7, 32.7, 20.3, 9.2.

HRMS (ESI) *m/z* calc. for $C_{24}H_{29}N_3O$ $[M+H]^+$ 376.2389, found 376.2384.

4-(8-(2-Ethyl-2-methylbutanamido)quinolin-5-yl)-*N,N,N*-trimethylbenzenaminium hexafluorophosphate (4a)

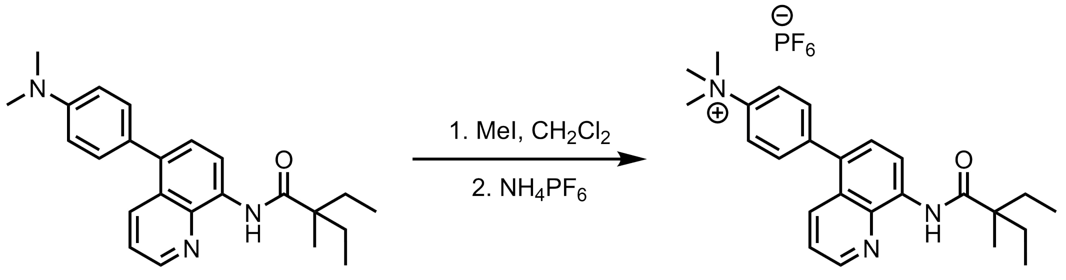

A solution of iodomethane (0.66 mL, 10.6 mmol, 20 equiv.) and *N*-(5-(4-(dimethylamino)phenyl)quinolin-8-yl)-2-ethyl-2-methylbutanamide (0.2 g, 0.53 mmol, 1.0 equiv.) in dichloromethane (10 mL), was heated at reflux for 24 hours. A white precipitate was collected by vacuum filtration, suspended in dichloromethane and washed with saturated, aqueous NH_4PF_6 solution (3×). The solvent was removed under reduced pressure to give the title compound **4a** as a beige solid (0.26 g, 93 % yield).

^{1}H NMR (CD_3CN, 400 MHz): δ = 10.28 (br s, 1H), 8.92 (dd, J = 4.4, 1.6 Hz, 1H), 8.80 (d, J = 8.0 Hz, 1H), 8.22 (dd, J = 8.4 Hz, 1.2 Hz, 1H), 7.91 – 7.88 (m, 2H), 7.72 – 7.69 (m, 2H), 7.56 (dd, J = 8.4, 4.4 Hz, 1H), overlapping with 7.53 (d, J = 8.0 Hz, 1H), 3.62 (s, 9H), 1.86 – 1.80 (m, 2H), 1.64 – 1.58 (m, 2H), 1.31 (s, 3H), 0.89 (t, J = 7.6 Hz, 6H).

^{13}C NMR (CD_3CN, 100 MHz): δ = 176.1, 149.4, 146.5, 141.9, 138.9, 135.4, 134.4, 132.2, 131.7, 128.7, 126.3, 123.0, 120.8, 115.5, 57.6, 48.3, 32.7, 19.9, 8.9.

HRMS (ESI) *m/z* calc. for $[C_{25}H_{32}N_3O]^+$ 390.2540 found 390.2536.

General Procedure for the Preparation of Cyclopalladated Complexes

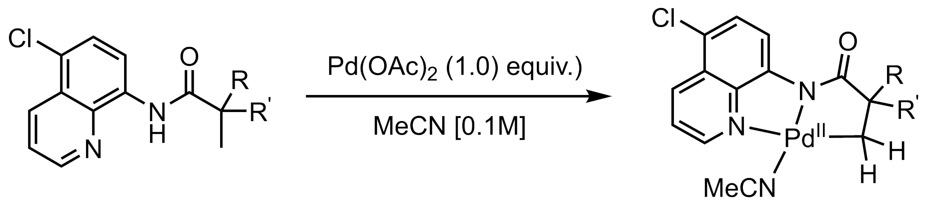

Amide (1.0 mmol) was dissolved in MeCN (10 mL). $Pd(OAc)_2$ (1 mmol, 1.0 equiv.) was added to the solution, and it was stirred under N_2 atmosphere at room temperature until a

precipitate formed. The precipitate was collected by vacuum filtration and dried under vacuum to yield the cyclopalladated complex as a bright yellow solid.

Cyclopalladated Complexes Characterisation Data

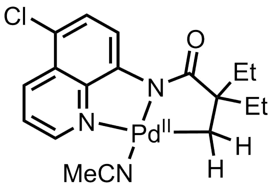

1

^{1}H NMR ($CDCl_3$, 400 MHz): δ = 9.10 (d, *J* = 8.6 Hz, 1H), 8.52 (d, *J* = 8.4 Hz, 1H), 8.33 (d, *J* = 3.8 Hz, 1H), 7.51 (d, *J* = 8.6 Hz, 1H), 7.40 (dd, *J* = 8.3, 4.5 Hz, 1H), 2.36 (s, 2H), 1.80 (s, 3H), 1.63 (dq, *J* = 14.3, 7.2 Hz, 2H), 1.42 (dq, *J* = 14.2, 7.2 Hz, 2H), 0.96 (t, *J* = 7.3 Hz, 6H).

^{13}C NMR ($CDCl_3$, 100 MHz): δ = 188.3, 146.7, 146.2, 145.6, 134.8, 128.9, 127.4, 121.3, 120.3, 118.6, 56.6, 34.1, 18.6, 9.6, 3.6.

HRMS (ESI) *m/z* calc. for $C_{18}H_{21}ClN_3OPd$ $[M+H]^+$ 436.0408 found 436.0405

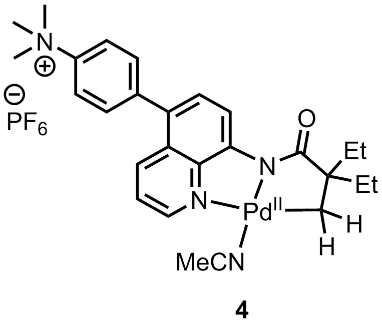

4

^{1}H NMR (CD_3CN, 400 MHz): δ = 9.15 (d, *J* = 8.4 Hz, 1H), 8.67 (dd, *J* = 4.4, 1.2 Hz, 1H), 8.33 (dd, *J* = 8.8, 1.2 Hz, 1H), 7.91 – 7.89 (m, 2H), 7.72 – 7.70 (m, 2H), 7.56 (dd, *J* = 8.8, 4.4 Hz, 1H), 7.49 (d, *J* = 8.4 Hz, 1H), 3.62 (s, 9H), 1.98 (s, 3H), 1.77 (s, 2H), 1.61 – 1.52 (m, 2H), 1.43 – 1.35 (m, 2H), 0.93 (t, *J* = 7.6 Hz, 6H).

^{13}C NMR (CD_3CN, 100 MHz): δ = 188.5, 148.4, 148.0, 146.6, 145.5, 142.3, 136.3, 132.6, 130.7, 129.1, 128.5, 122.7, 121.2, 120.2, 58.1, 57.2, 34.9, 17.7, 9.8.

HRMS (ESI) *m/z* calc. for $[C_{27}H_{33}N_4OPd^+]$ 535.1684 found 535.1680

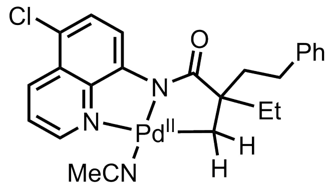

13b

^{1}H NMR ($CDCl_3$, 400 MHz): δ = 9.14 (d, J = 8.8 Hz, 1H), 8.57 (d, J = 8.4 Hz, 1H), 8.40 (d, J = 4.4 Hz, 1H), 7.55 (d, J = 8.8 Hz, 1H), 7.45 (dd, J = 8.8, 4.4 Hz, 1H), 7.24 – 7.20 (m, 4H), 7.14 – 7.11 (m, 1H), 2.94 – 2.86 (m, 1H), 2.71 – 2.63 (m, 1H), 2.37 (s, 3H), 1.93 – 1.89 (m, 2H), 1.74 – 1.67 (m, 3H), 1.53 – 1.48 (m, 1H), 0.99 (t, J = 7.2 H, 3H)

^{13}C NMR ($CDCl_3$, 100 MHz): δ = 187.9, 146.7, 146.3, 145.7, 144.1, 135.0, 129.1, 128.7, 128.2, 127.5, 125.4, 121.3, 120.5, 120.4, 56.4, 43.7, 34.5, 31.7, 19.1, 9.5, 3.6, 3.5.

HRMS (ESI) *m/z* calc. for $C_{24}H_{25}ClN_3OPd$ $[M+H]^+$ 512.0721 found 512.0734

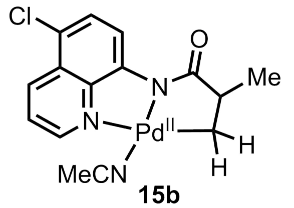

15b

^{1}H NMR ($CDCl_3$, 400 MHz): δ = 9.00 (d, J = 8.7 Hz, 1H), 8.53 (d, J = 8.6 Hz, 1H), 8.29 (d, J = 4.4 Hz, 1H), 7.52 (d, J = 8.2 Hz, 1H), 7.38 (dd, J = 8.4, 4.5 Hz, 1H), 2.79 (h, J = 7.7, 7.2 Hz, 1H), 2.37 (s, 3H), 2.05 (t, J = 8.5 Hz, 1H), 1.57 (t, J = 8.2 Hz, 1H), 1.24 (d, J = 6.8 Hz, 3H).

^{13}C NMR ($CDCl_3$, 100 MHz): δ =187.8, 146.7, 146.3, 145.5, 134.9, 129.0, 127.4, 121.3, 120.4, 120.1, 118.6, 47.0, 21.2, 16.2, 3.6.

HRMS (ESI) *m/z* calc. for $C_{15}H_{15}ClN_3OPd$ $[M+H]^+$ 393.9939 found 393.9931

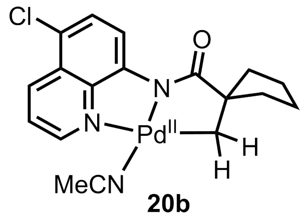

20b

^{1}H NMR ($CDCl_3$, 400 MHz): δ = 9.08 – 9.00 (m, 1H), 8.48 (d, J = 8.4 Hz, 1H), 8.24 (d, J = 4.3 Hz, 1H), 7.50 (d, J = 8.6 Hz, 1H), 7.34 (dd, J = 8.3, 4.5 Hz, 1H), 2.37 – 2.34 (m, 2H), 2.08 – 1.97 (m, 2H), 1.86 (s, 2H), 1.79 – 1.58 (m, 6H).

^{13}C NMR ($CDCl_3$, 100 MHz): δ = 189.4, 146.9, 146.2, 145.4, 134.7, 128.8, 127.3, 121.4, 119.9, 118.7, 60.5, 41.9, 25.5, 25.3, 3.6.

HRMS (ESI) *m/z* calc. for $C_{18}H_{19}ClN_3OPd$ $[M+H]^+$ 434.0252 found 434.0260

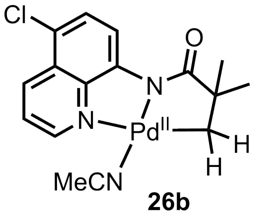

26b

^{1}H NMR ($CDCl_3$, 400 MHz): δ = 9.04 (d, *J* = 8.6 Hz, 1H), 8.53 (d, *J* = 8.5 Hz, 1H), 8.33 (s, 1H), 7.52 (d, *J* = 8.6 Hz, 1H), 7.40 (dd, *J* = 8.4, 4.5 Hz, 1H), 2.36 (s, 2H), 1.84 (s, 3H), 1.26 (s, 6H).

^{13}C NMR ($CDCl_3$, 100 MHz): δ = 189.7, 147.0, 146.3, 145.5, 134.9, 129.0, 127.4, 121.4, 120.5, 120.2, 118.7, 49.5, 30.2, 25.5, 3.6.

HRMS (ESI) *m/z* calc. for $C_{16}H_{17}ClN_3OPd$ $[M+H]^+$ 408.0095 found 408.0093.

General Procedure for the Iodination Reaction

Procedure A:

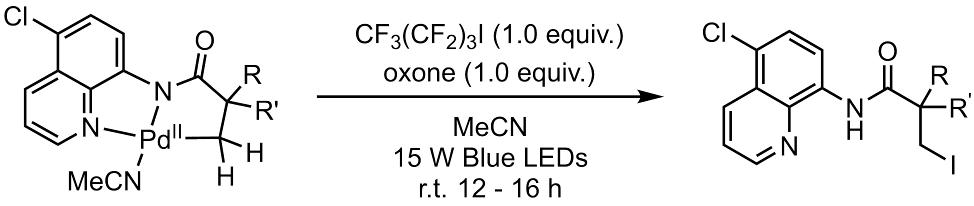

Cyclopalladated complex (0.1 mmol) and oxone (30.7 mg, 0.1 mmol) were placed in a pressure relief sample vial and taken up in MeCN (1 mL). The reaction mixture was sparged with N_2 for 5 minutes. Perfluorobutyl iodide (0.1 mmol, 1 equiv.) was added via air-tight syringe and the solution was sparged with N_2 for 1 minute. The cap was wrapped in parafilm and the reaction mixture was stirred for 16 hours at room temperature, whilst being irradiated with 15 W Blue LEDs. The reaction mixture was diluted with CH_2Cl_2 and washed with brine. The aqueous layer was re-extracted with CH_2Cl_2 (3×) and dried ($MgSO_4$). The solvent was removed under reduced pressure and the residue was purified by flash chromatography on deactivated silica, eluting 10-20% EtOAc/hexane.

Procedure B:

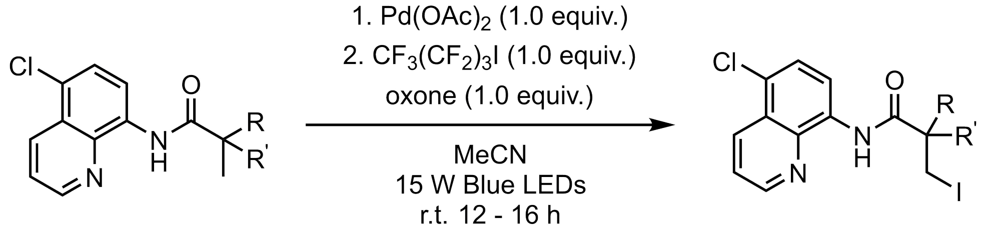

Amide (0.1 mmol) and $Pd(OAc)_2$ (0.1 mmol) were placed in a pressure relief sample vial and dissolved in acetonitrile (1 mL). The solution was stirred at room temperature for 3 hours. Oxone (30.7 mg, 0.1 mmol) was added and the reaction mixture was sparged with N_2 for 5 minutes. Perfluorobutyl iodide (0.1 mmol, 1 equiv.) was added via air-tight syringe and the solution was sparged with N_2 for 1 minute. The cap was wrapped in parafilm and the reaction mixture was stirred for 16 hours at room temperature, whilst being irradiated with 15 W Blue LEDs. The reaction mixture was diluted with CH_2Cl_2 and washed with brine. The aqueous layer was re-extracted with CH_2Cl_2 (3×) and dried ($MgSO_4$). The solvent was removed under reduced pressure and the residue was purified by flash chromatography on deactivated silica, eluting 10-20% EtOAc/hexane.

Product Characterisation Data

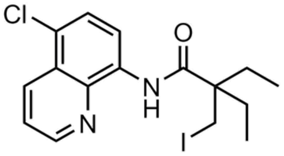

3

***N*-(5-Chloroquinolin-8-yl)-2-ethyl-2-(iodomethyl)butanamide (3):** Prepared according to Procedure A. Obtained as a white solid (37 mg, 89%)

^{1}H NMR ($CDCl_3$, 400 MHz): δ = 10.24 (br s, 1H), 8.88 (dd, *J* = 4.2, 1.4 Hz, 1H), 8.74 (d, *J* = 8.4 Hz, 1H), 8.58 (dd, *J* = 8.5, 1.4 Hz, 1H), 7.61 (d, *J* = 8.5 Hz, 2H), 7.58 (dd, *J* = 8.4, 4.2 Hz, 1H) 3.58 (s, 2H), 2.01-1.85 (m, 4H), 0.93 (t, *J* = 7.4 Hz, 6H).

^{13}C NMR ($CDCl_3$, 100 MHz): δ =171.9, 149.0, 139.4, 133.6, 133.5, 127.4, 126.1, 124.6, 122.5, 116.5, 51.3, 28.8, 13.1, 8.8.

HRMS (ESI) *m/z* calc. for $C_{16}H_{19}ClIN_2O$ $[M+H]^+$ 417.0231 found 417.0226.

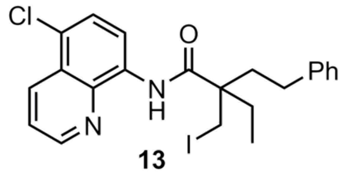

13

***N*-(5-Chloroquinolin-8-yl)-2-ethyl-2-(iodomethyl)-4-phenylbutanamide (13):** Prepared according to General Procedure A. Obtained as a white solid (34 mg, 70%)

^{1}H NMR ($CDCl_3$, 400 MHz): δ = 10.31 (br s, 1H), 8.88 (dd, *J* = 4.2, 1.5 Hz, 1H), 8.74 (d, *J* = 8.4 Hz, 1H), 8.59 (dd, *J* = 8.5, 1.5 Hz, 1H), 7.63 (d, *J* = 8.4 Hz, 1H), overlapping with 7.60 (dd, *J* = 8.5, 4.2 Hz, 1H), 7.22 (m, 5H), 3.78 – 3.58 (m, 2H), 2.65 – 2.57 (m, 2H), 2.27 – 2.07 (m, 2H), 2.03 – 1.96 (m, 2H), 0.98 (t, *J* = 7.4 Hz, 3H).

^{13}C NMR ($CDCl_3$, 100 MHz): δ = 171.3, 149.0, 141.4, 139.4, 133.6, 133.4, 128.6, 128.6, 127.4, 126.2, 126.1, 124.8, 122.6, 116.7, 50.8, 39.0, 31.0, 29.7, 13.2, 8.6.

HRMS (ESI) *m/z* calc. for $C_{22}H_{23}ClIN_2O$ $[M+H]^+$ 493.0544 found 493.0541.

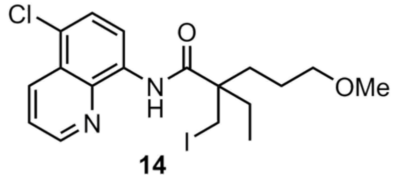

14

***N*-(5-Chloroquinolin-8-yl)-2-ethyl-2-(iodomethyl)-5-methoxypentanamide (14):** Prepared according to Procedure B. Obtained as a white solid (30 mg, 66%)

^{1}H NMR ($CDCl_3$, 400 MHz): δ = 10.24 (br s, 1H), 8.88 (dd, *J* = 4.2, 1.4 Hz, 1H), 8.72 (d, *J* = 8.4 Hz, 1H), 8.58 (dd, *J* = 8.5, 1.4 Hz, 1H), 7.60 (d, *J* = 8.4 Hz, 1H), overlapping with 7.59 (dd, *J* = 8.5, 4.2 Hz, 1H). 3.64 – 3.54 (m, 2H), 3.40 (t, *J* = 6.4 Hz, 2H), 3.28 (s, 3H), 2.01-1.83 (m, 4H), 1.64 – 1.49 (m, 2H), 0.94 (t, *J* = 7.4 Hz, 3H).

^{13}C NMR ($CDCl_3$, 100 MHz): δ = 171.7, 149.0, 139.4, 133.6, 133.5, 127.4, 126.1, 124.7, 122.5, 116.6, 72.6, 58.6, 50.7, 32.8, 29.1, 24.7, 13.2, 8.6.

HRMS (ESI) *m/z* calc. for $C_{18}H_{23}ClIN_2O$ $[M+H]^+$ 461.0493 found 461.0488.

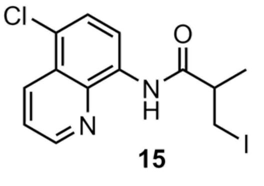

15

***N*-(5-Chloroquinolin-8-yl)-3-iodo-2-methylpropanamide (15):** Prepared according to Procedure A. Obtained as a white solid (28 mg, 75%)

^{1}H NMR ($CDCl_3$, 400 MHz): δ = 9.89 (s, 1H), 8.87 (dd, *J* = 4.2, 1.6 Hz, 1H), 8.74 (d, *J* = 8.4 Hz, 1H), 8.58 (dd, *J* = 8.5, 1.6 Hz, 1H), 7.64 – 7.55 (m, 2H), 3.57 (dd, *J* = 9.8, 7.5 Hz, 1H), 3.33 (dd, *J* = 9.8, 6.2 Hz, 1H), 2.99 (h, *J* = 6.9 Hz, 1H), 1.47 (d, *J* = 6.9 Hz, 3H).

^{13}C NMR ($CDCl_3$, 100 MHz) δ = 172.1, 148.8, 139.0, 133.8, 133.4, 127.4, 126.1, 124.9, 122.6, 117.0, 45.9, 19.4, 7.4.

HRMS (ESI) *m/z* calc. for $C_{13}H_{13}ClIN_2O$ $[M+H]^+$ 374.9761 found 374.9768.

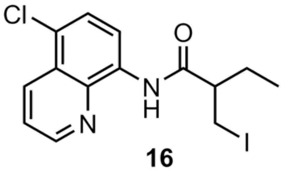

16

***N*-(5-Chloroquinolin-8-yl)-2-(iodomethyl)butanamide (16):** Prepared according to Procedure B. Obtained as a white solid (34 mg, 87%)

^{1}H NMR ($CDCl_3$, 400 MHz): δ = 9.88 (br s, 1H), 8.87 (dd, *J* = 4.2, 1.4 Hz, 1H), 8.77 (d, *J* = 8.4 Hz, 1H), 8.58 (dd, *J* = 8.5, 1.3 Hz, 1H), 7.61 (d, *J* = 8.4 Hz, 1H), 7.59 (dd, *J* = 8.5, 4.2 Hz, 1H), 3.57 – 3.52 (m, 1H), 3.34 (dd, *J* = 9.8, 5.3 Hz, 1H), 2.85 – 2.78 (m, 1H), 1.96 – 1.71 (m, 2H), 1.05 (t, *J* = 7.4 Hz, 3H).

^{13}C NMR ($CDCl_3$, 100 MHz) δ = 171.8, 148.9, 139.1, 133.6, 133.4, 127.4, 126.1, 124.8, 122.5, 116.8, 53.6, 27.4, 11.9, 5.5.

HRMS (ESI) *m/z* calc. for $C_{14}H_{15}ClIN_2O$ $[M+H]^+$ 388.9918 found 388.9913.

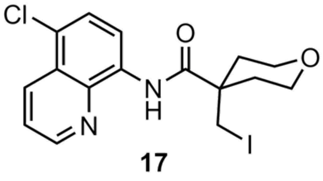

17

***N*-(5-Chloroquinolin-8-yl)-4-(iodomethyl)tetrahydro-2H-pyran-4-carboxamide (17)** Prepared according to Procedure B. Obtained as a white solid (34 mg, 79%)

^{1}H NMR ($CDCl_3$, 400 MHz): δ = 10.26 (br s, 1H), 8.87 (dd, *J* = 4.1, 1.3 Hz, 1H), 8.74 (d, *J* = 8.4 Hz, 1H), 8.60 (dd, *J* = 8.5, 1.3 Hz, 1H), 7.63 (d, *J* = 8.4 Hz, 1H), 7.60 (dd, *J* = 8.5, 4.1 Hz, 1H), 3.92 – 3.87 (m, 2H), 3.75 – 3.66 (m, 2H), 3.51 (s, 2H), 2.41 – 2.37 (m, 2H), 1.92 – 1.85 (m, 2H).

^{13}C NMR ($CDCl_3$, 100 MHz): δ = 171.3, 149.0, 139.3, 133.9, 133.1, 127.4, 126.2, 125.1, 122.6, 117.0, 65.0, 46.5, 35.0, 15.7.

HRMS (ESI) *m/z* calc. for $C_{16}H_{17}ClIN_2O_2$ $[M+H]^+$ 431.0023 found 431.0016.

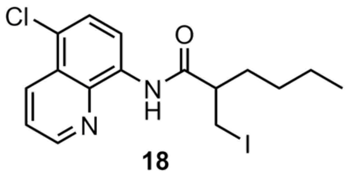

18

***N*-(5-Chloroquinolin-8-yl)-2-(iodomethyl)hexanamide (18):** Prepared according to Procedure B. Obtained as a white solid (35 mg, 85%)

^{1}H NMR ($CDCl_3$, 400 MHz): δ = 9.87 (br s, 1H), 8.88 (dd, *J* = 4.2, 1.6 Hz, 1H), 8.76 (d, *J* = 8.4 Hz, 1H), 8.58 (dd, *J* = 8.5, 1.6 Hz, 1H), 7.61 (d, *J* = 8.4 Hz, 1H), 7.59 (dd, *J* = 8.5, 4.2 Hz, 1H), 3.56 – 3.51 (m, 1H), 3.33 (dd, *J* = 9.8, 5.3 Hz, 1H), 2.85 – 2.78 (m, 1H), 1.86 – 1.72 (m, 1H), 1.77 – 1.63 (m, 1H), 1.45 – 1.33 (m, 4H), 0.88 (t, *J* = 7.1 Hz, 3H).

^{13}C NMR ($CDCl_3$, 100 MHz): δ = 172.0, 148.9, 139.1, 133.7, 133.4, 127.4, 126.1, 124.8, 122.5, 116.9, 52.2, 33.9, 29.6, 22.7, 14.0, 5.9.

HRMS (ESI) *m/z* calc. for $C_{16}H_{19}ClIN_2O$ $[M+H]^+$ 417.0231 found 417.0223.

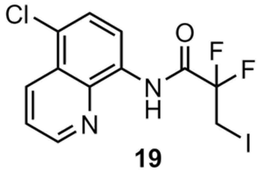

19

***N*-(5-Chloroquinolin-8-yl)-2,2-difluoro-3-iodopropanamide (19):** Prepared according to Procedure B. Obtained as a white solid (21 mg, 54%)

^{1}H NMR ($CDCl_3$, 400 MHz): δ = 10.77 (br s, 1H), 8.92 (dd, *J* = 4.2, 1.6 Hz, 1H), 8.70 (d, *J* = 8.4 Hz, 1H), 8.61 (dd, *J* = 8.5, 1.6 Hz, 1H), 7.66 (d, *J* = 8.4 Hz, 1H), 7.65 (dd, *J* = 8.8, 4.4 Hz, 1H), 3.81 (t, *J* = 15.0 Hz, 2H).

^{19}F NMR ($CDCl_3$, 376 MHz): δ = -100.78 (td, *J* = 15.0, 2.3 Hz).

^{13}C NMR ($CDCl_3$, 125 MHz): δ = 160.0 (t, *J* = 28.3 MHz), 149.6, 139.4, 133.7, 132.0, 127.1, 126.6, 126.3, 122.9, 117.5, 114.7 (t, *J* = 253.8 MHz), 29.9, -0.09 (t, *J* = 5.4 MHz).

HRMS (ESI) *m/z* calc. for $C_{12}H_9ClF_2IN_2O$ $[M+H]^+$ 396.9416 found 396.9407.

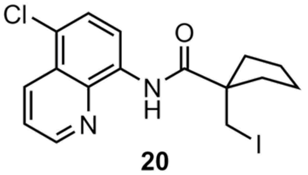

20

***N*-(5-Chloroquinolin-8-yl)-1-(iodomethyl)cyclopentanecarboxamide (20):** Prepared according to Procedure A. Obtained as a white solid (28 mg, 67%)

^1H NMR ($CDCl_3$, 400 MHz): δ = 10.20 (br s, 1H), 8.87 (dd, *J* = 4.2, 1.4 Hz, 1H), 8.75 (d, *J* = 8.4 Hz, 1H), 8.57 (dd, *J* = 8.5, 1.4 Hz, 1H), 7.61 (d, *J* = 8.4 Hz, 1H), 7.58 (dd, *J* = 8.5, 4.2 Hz, 1H), 3.60 (s, 2H), 2.41 – 2.38 (m, 2H), 1.98 – 1.83 (m, 6H).

^{13}C NMR ($CDCl_3$, 100 MHz) δ = 173.6, 149.0, 139.4, 133.6, 133.6, 127.4, 126.1, 124.6, 122.5, 116.5, 57.1, 37.2, 25.1, 15.6.

HRMS (ESI) *m/z* calc. for $C_{16}H_{17}ClIN_2O$ $[M+H]^+$ 415.0074 found 415.0065.

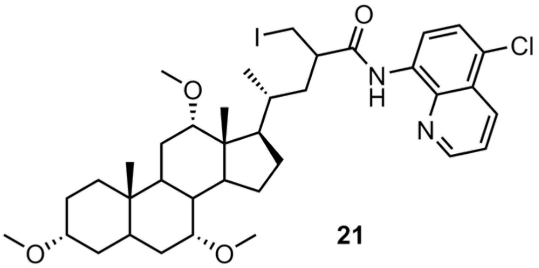

21

(4R)-*N*-(5-Chloroquinolin-8-yl)-2-(iodomethyl)-4-((3R,7R,10S,12S,13R,17R)-3,7,12-trimethoxy-10,13-dimethylhexadecahydro-1H-cyclopenta[a]phenanthren-17-yl)pentanamide (21): Prepared according to Procedure B. Obtained as a yellow oil (63 mg, 84%)

^1H NMR ($CDCl_3$, 400 MHz): δ = 9.89 (s, 1H), 9.89 (dd, *J* = 4.4, 1.6 Hz, 1H), 8.75 (d, *J* = 8.4 Hz, 1H), 8.58 (dd, *J* = 8.4, 1.6 Hz, 1H), 7.62 – 7.57 (m, 2H), 3.49 – 3.31 (m, 4H), overlapping with 3.32 (s, 3H), 3.24 (s, 3H), 3.20 (s, 3H), 3.15 – 3.13 (m, 1H), 3.02 – 2.90 (m, 2H), 2.22 – 1.43 (m, 17H), 1.35 – 1.15 (m, 5H), 1.02 (d, *J* = 6.4 Hz, 3H), 0.93 – 0.86 (m, 1H), overlapping with 0.89 (s, 3H), 0.68 (s, 3H).

^{13}C NMR ($CDCl_3$, 100 MHz): δ = 172.5, 148.9, 139.2, 133.6, 133.5, 127.4, 126.1, 124.7, 122.5, 116.8, 82.1, 80.9, 77.1, 56.0, 55.8, 55.6, 49.4, 47.3, 46.5, 42.8, 42.1, 40.2, 39.8, 35.4, 35.1, 34.6, 34.3, 28.1, 28.0, 27.9, 26.9, 23.3, 23.0, 22.1, 18.0, 12.7, 5.8.

HRMS (ESI) *m/z* calc. for $C_{37}H_{53}ClIN_2O_4$ $[M+H]^+$ 751.2739 found 751.2724.

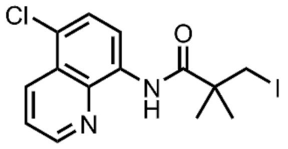

37

3-Iodo-*N*-(5-chloroquinolin-8-yl)-2,2-dimethylpropanamide (37): Obtained as a white solid (24 mg, 62%)

^{1}H NMR (400 MHz, $CDCl_3$) δ 10.25 (s, 1H), 8.86 (dd, J = 4.2, 1.5 Hz, 1H), 8.73 (d, J = 8.4 Hz, 1H), 8.55 (dd, J = 8.5, 1.6 Hz, 1H), 7.62 – 7.53 (m, 2H), 3.52 (s, 2H), 1.58 (s, 6H).

^{13}C NMR (101 MHz, $CDCl_3$) δ 173.1, 148.9, 139.4, 133.5, 127.3, 126.04, 124.7, 122.5, 116.6, 44.7, 25.9, 17.7.

HRMS (ESI) *m/z* calc. for $C_{14}H_{15}ClIN_2O$ $[M+H]^+$ 388.9917 found 388.9908

General Procedure for the Chlorination Reaction

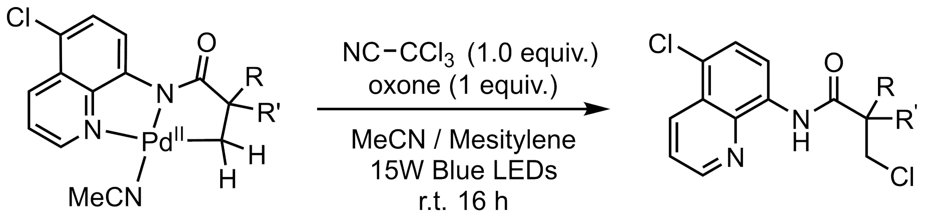

Cyclopalladated complex (0.1 mmol), oxone (30.7 mg, 0.1 mmol) and mesitylene (0.139 mL, 1.0 mmol) were placed in a pressure relief sample vial and taken up in MeCN (1 ml). The reaction mixture was sparged with N_2 for 5 minutes. Trichloroacetonitrile (0.01 mL, 0.1 mmol) was then added and the solution was sparged with N_2 for 1 minute. The cap was wrapped in parafilm and the reaction mixture was stirred for 16 hours at room temperature, whilst being irradiated with 15W Blue LEDs. The reaction mixture was diluted with CH_2Cl_2 and washed with brine. The aqueous layer was re-extracted with

CH_2Cl_2 (3×) and dried ($MgSO_4$). The solvent was removed under reduced pressure and the residue was purified by flash chromatography on deactivated silica, eluting 10-20% EtOAc/hexane.

Product Characterization Data

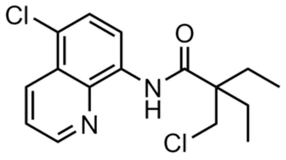

22

2-(Chloromethyl)-*N*-(5-chloroquinolin-8-yl)-2-ethylbutanamide (22): Obtained as a white solid (22.4 mg, 69%)

^{1}H NMR ($CDCl_3$, 400 MHz): δ = 10.25 (br s, 1H), 8.88 (dd, *J* = 4.2, 1.5 Hz, 1H), 8.74 (d, *J* = 8.4 Hz, 1H), 8.58 (dd, *J* = 8.5, 1.5 Hz, 1H), 7.64 – 7.54 (m, 2H), 3.88 (s, 2H), 1.98 (dq, *J* = 14.9, 7.5 Hz, 2H), 1.86 (dq, *J* = 14.6, 7.4 Hz, 2H), 0.96 (t, *J* = 7.4 Hz, 6H).

^{13}C NMR ($CDCl_3$, 100 MHz): δ = 173.22, 148.93, 139.32, 133.75, 133.49, 127.42, 126.14, 124.63, 122.50, 116.72, 52.83, 46.71, 26.63, 8.50.

HRMS (ESI) *m/z* calc. for $C_{16}H_{19}Cl_2N_2O$ $[M+H]^+$ 325.0874 found 325.0868.

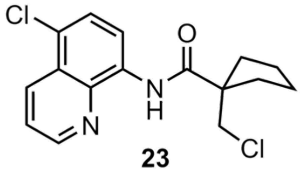

23

1-(Chloromethyl)-*N*-(5-chloroquinolin-8-yl)cyclopentanecarboxamide (23):
Obtained as a white solid (19.4 mg, 60%)

^{1}H NMR ($CDCl_3$, 400 MHz): δ = 10.26 (br s, 1H), 8.87 (dd, *J* = 4.2, 1.5 Hz, 1H), 8.75 (d, *J* = 8.4 Hz, 1H), 8.57 (dd, *J* = 8.5, 1.5 Hz, 1H), 7.66 – 7.51 (m, 2H), 3.86 (s, 2H), 2.38 – 2.26 (m, 2H), 2.03 – 1.75 (m, 6H).

^{13}C NMR ($CDCl_3$, 100 MHz) δ = 173.67, 148.96, 139.43, 133.78, 133.53, 127.36, 126.07, 124.53, 122.47, 116.53, 57.93, 50.84, 34.98, 25.15.

HRMS (ESI) *m/z* calc. for $C_{16}H_{17}Cl_2N_2O$ $[M+H]^+$ 323.0718 found 323.0718.

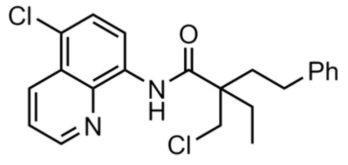

24

2-(Chloromethyl)-*N*-(5-chloroquinolin-8-yl)-2-ethyl-4-phenylbutanamide (24): Obtained as a white solid (28.1 mg, 70%)

^{1}H NMR (CDCl$_3$, 400 MHz): δ = 10.32 (s, 1H), 8.88 (dd, *J* = 4.2, 1.5 Hz, 1H), 8.73 (d, *J* = 8.4 Hz, 1H), 8.59 (dd, *J* = 8.5, 1.5 Hz, 1H), 7.72 – 7.52 (m, 2H), 7.22 (dq, *J* = 16.8, 8.7, 8.0 Hz, 5H), 3.97 (q, *J* = 11.6 Hz, 2H), 2.64 (dtd, *J* = 30.2, 13.0, 5.0 Hz, 2H), 2.25 (td, *J* = 13.2, 12.3, 5.4 Hz, 1H), 2.12 – 2.00 (m, 2H), 1.95 (dq, *J* = 14.6, 7.4 Hz, 1H), 1.01 (t, *J* = 7.4 Hz, 6H).

^{13}C NMR (CDCl$_3$, 100 MHz): δ = 172.80, 149.01, 141.50, 139.42, 133.64, 133.45, 128.59, 128.53, 127.36, 126.20, 126.15, 124.73, 122.57, 116.69, 52.45, 46.76, 36.79, 30.74, 27.29, 8.47.

HRMS (ESI) *m/z* calc. for $C_{22}H_{23}Cl_2N_2O$ [M+H]$^+$ 401.1187 found 401.1186.

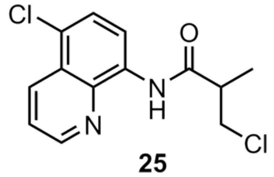

25

3-Chloro-*N*-(5-chloroquinolin-8-yl)-2-methylpropanamide (25): Obtained as a white solid (15.3 mg, 54%)

^{1}H NMR (CDCl$_3$, 400 MHz): δ = 9.96 (br s, 1H), 8.87 (dd, *J* = 4.2, 1.3 Hz, 1H), 8.75 (d, *J* = 8.4 Hz, 1H), 8.59 (dd, *J* = 8.5, 1.3 Hz, 1H), 7.66 – 7.51 (m, 2H), 3.91 (dd, *J* = 10.8, 7.8 Hz, 1H), 3.68 (dd, *J* = 10.8, 5.7 Hz, 1H), 3.01 (h, *J* = 7.0 Hz, 1H), 1.45 (d, *J* = 7.0 Hz, 3H).

^{13}C NMR (CDCl$_3$, 100 MHz): δ = 171.98, 148.80, 138.97, 133.82, 133.50, 127.42, 126.12, 124.85, 122.54, 116.98, 46.49, 45.62, 16.36.

HRMS (ESI) *m/z* calc. for $C_{13}H_{13}Cl_2N_2O$ [M+H]$^+$ 283.0405 found 283.0399

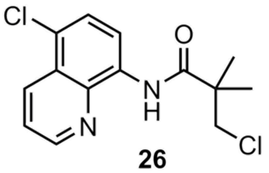

26

3-Chloro-*N*-(5-chloroquinolin-8-yl)-2,2-dimethylpropanamide (26): Obtained as a white solid (19.5 mg, 65%)

^{1}H NMR ($CDCl_3$, 400 MHz): δ = 10.3 (br s, 1H), 8.88 (dd, *J* = 4.0, 1.6 Hz, 1H), 8.75 (d, *J* = 8.4 Hz, 1H), 8.57 (dd, *J* = 8.8, 1.6 Hz, 1H), 7.60 (d, *J* = 8.4 Hz, 1H), 7.59 (dd, *J* = 8.8, 4.0 Hz, 1H), 3.78 (s, 2H), 1.54 (s, 6H).

^{13}C NMR ($CDCl_3$, 100 MHz): δ = 173.6, 149.0, 139.4, 133.6, 127.3, 126.1, 124.7, 122.5, 116.6, 52.9, 46.0, 23.8.

HRMS (ESI) *m/z* calc. for $C_{14}H_{15}Cl_2N_2O$ $[M+H]^+$ 297.0561 found 297.0556.

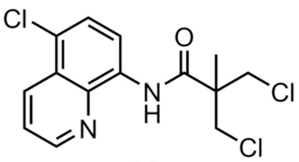

26c

HRMS (ESI) *m/z* calc. for $C_{14}H_{14}Cl_3N_2O$ $[M+H]^+$ 331.0172 found 331.0166.

General Procedure for the Bromination Reaction

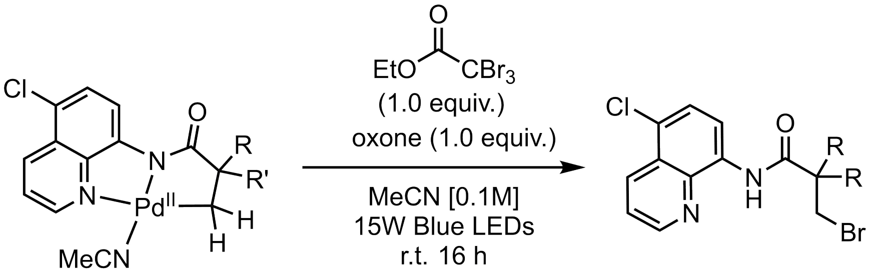

Cyclopalladated complex (0.1 mmol), ethyl tribromoacetate (32.4 mg, 0.1 mmol) and oxone (30.7mg, 0.1 mmol) were placed in a pressure relief sample vial and taken up in MeCN (1ml). The reaction mixture was sparged with N_2 for 1 minute. The cap was wrapped in parafilm and the reaction mixture was stirred for 16 hours, whilst being irradiated with 15W Blue LEDs at room temperature. The reaction mixture was diluted with CH_2Cl_2 and washed with brine. The aqueous layer was re-extracted with CH_2Cl_2 (3×) and dried ($MgSO_4$). The solvent was removed under reduced pressure and the residue

was purified by flash chromatography on deactivated silica, eluting 10-20% EtOAc/hexane.

Product Characterisation Data

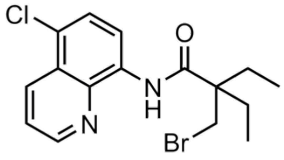

27

2-(Bromomethyl)-*N*-(5-chloroquinolin-8-yl)-2-ethylbutanamide (27): Obtained as a white solid (22.6 mg, 61%)

^{1}H NMR ($CDCl_3$, 400 MHz): δ = 10.25 (br s, 1H), 8.88 (dd, *J* = 4.2, 1.4 Hz, 1H), 8.74 (d, *J* = 8.4 Hz, 1H), 8.58 (dd, *J* = 8.5, 1.4 Hz, 1H), 7.60 (d, *J* = 8.4 Hz, 1H), overlapping with 7.58 (dd, *J* = 8.5, 4.2 Hz, 1H), 3.76 (s, 2H), 2.02 – 1.86 (m, 4H), 0.95 (t, *J* = 7.4 Hz, 6H).

^{13}C NMR ($CDCl_3$, 100 MHz): δ = 172.8, 149.0, 139.4, 133.7, 133.5, 127.4, 126.1, 124.6, 122.5, 116.7, 52.2, 37.0, 27.4, 8.6.

HRMS (ESI) *m/z* calc. for $C_{16}H_{19}BrClN_2O$ $[M+H]^+$ 369.0369 found 369.0359.

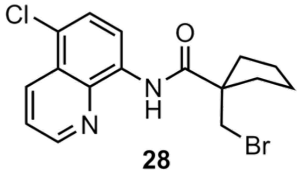

28

1-(Bromomethyl)-*N*-(5-chloroquinolin-8-yl)cyclopentanecarboxamide (28):
Obtained as a white solid (18.4 mg, 50%)

^{1}H NMR ($CDCl_3$, 400 MHz) δ = 10.25 (s, 1H), 8.88 (dd, *J* = 4.3, 1.6 Hz, 1H), 8.76 (d, *J* = 8.4 Hz, 1H), 8.60 (dd, *J* = 8.5, 1.6 Hz, 1H), 7.62 (d, *J* = 8.4 Hz, 1H), overlapping with 7.59 (dd, *J* = 8.5, 4.3 Hz, 1H), 3.77 (s, 2H) 2.44 – 2.29 (m, 2H), 2.04 – 1.77 (m, 6H).

^{13}C NMR ($CDCl_3$, 100 MHz) δ = 173.6, 148.90 139.3, 133.8, 133.7, 127.5, 126.1, 125.0, 124.6, 122.5, 116.8, 57.6, 40.3, 36.0, 25.1.

HRMS (ESI) *m/z* calc. for $C_{16}H_{17}BrClN_2O$ $[M+H]^+$ 367.0213 found 367.0211.

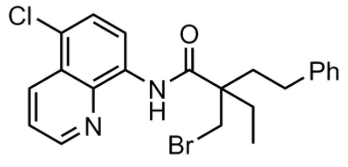

29

2-(Bromomethyl)-*N*-(5-chloroquinolin-8-yl)-2-ethyl-4-phenylbutanamide (29): Obtained as a white solid (22.2 mg, 50%)

^{1}H NMR (CDCl$_3$, 400 MHz): δ = 10.31 (s, 1H), 8.88 (dd, *J* = 4.2, 1.2 Hz, 1H), 8.74 (d, *J* = 8.4 Hz, 1H), 8.60 (dd, *J* = 8.5, 1.3 Hz, 1H), 7.63 (d, *J* = 8.4 Hz, 1H), overlapping with 7.58 (dd, *J* = 8.5, 4.2 Hz, 1H), 7.35 – 7.04 (m, 5H), 3.85 (q, *J* =12.7 Hz, 2H) 2.73 – 2.54 (m, 2H), 2.26 (td, *J* = 13.1, 12.2, 5.4 Hz, 1H), 2.73-2.54 (m, 3H), 1.00 (t, *J* = 7.4 Hz, 3H).

^{13}C NMR (CDCl$_3$, 100 MHz): δ = 172.4, 149.0, 141.4, 139.4, 133.7, 133.4, 128.6, 127.4, 126.2, 124.8, 122.6, 116.7, 51.8, 37.5, 37.0, 30.8, 28.1, 8.5.

HRMS (ESI) *m/z* calc. for $C_{22}H_{22}BrClN_2O$ $[M]^+$ 444.0604 found 444.0601.

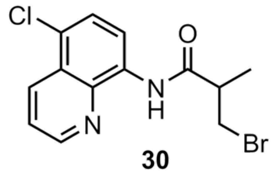

30

3-Bromo-*N*-(5-chloroquinolin-8-yl)-2-methylpropanamide (30): Obtained as a white solid (15.98 mg, 48%)

^{1}H NMR (CDCl$_3$, 400 MHz): δ = 10.00 (s, 1H), 8.88 (dd, *J* = 4.3, 1.6 Hz, 1H), 8.77 (d, *J* = 8.4 Hz, 1H), 8.63 (dd, *J* = 8.5, 1.6 Hz, 1H), 7.68 – 7.53 (m, 2H), 3.77 (dd, *J* = 10.0, 7.8 Hz, 1H), 3.54 (dd, *J* = 10.0, 5.8 Hz, 1H), 3.10 (dq, *J* = 13.9, 7.0 Hz, 1H), 1.47 (d, *J* = 6.9 Hz, 3H).

^{13}C NMR (CDCl$_3$, 100 MHz): δ = 172.1, 148.6, 138.7, 134.2, 133.4, 127.6, 126.2, 125.0, 122.5, 117.4, 45.5, 34.5, 17.6.

HRMS (ESI) *m/z* calc. for $C_{13}H_{13}BrClN_2O$ $[M+H]^+$ 326.9900 found 326.9895.

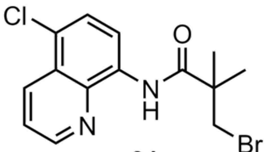

31

3-Bromo-*N*-(5-chloroquinolin-8-yl)-2,2-dimethylpropanamide (31): Obtained as a white solid (18.76 mg, 55%)

^{1}H NMR ($CDCl_3$, 400 MHz): δ = 10.28 (br s, 1H), 8.87 (dd, *J* = 4.2, 1.5 Hz, 1H), 8.75 (d, *J* = 8.4 Hz, 1H), 8.58 (dd, *J* = 8.5, 1.5 Hz, 1H), 7.64 – 7.54 (m, 2H), 3.68 (s, 2H), 1.58 (s, 6H).

^{13}C NMR ($CDCl_3$, 100 MHz): δ = 173.5, 148.9, 139.4, 133.7, 133.5, 127.4, 126.11, 124.8, 122.5, 116.7, 45.5, 42.3, 38.4, 24.7.

HRMS (ESI) *m/z* calc. for $C_{14}H_{15}BrClN_2O$ $[M+H]^+$ 341.0056 found 341.0054.

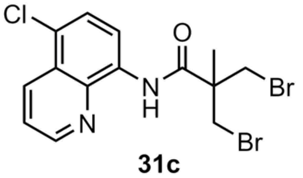

31c

HRMS (ESI) *m/z* calc. for $C_{14}H_{14}Br_2ClN_2O$ $[M+H]^+$ 418.9161 found 418.9159.

General Procedure for the Removal of Aminoquinoline Directing Group[8]

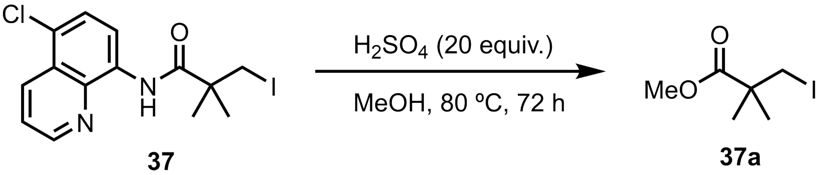

3-Iodo-*N*-(5-chloroquinolin-8-yl)-2,2-dimethylpropanamide (0.15 mmol) and concentrated sulfuric acid (3 mmol, 20 equiv.) were added to a sample vial and taken up in MeOH (1.5 ml). The vial was sealed and heated at 80 °C for 72 hours. After cooling to room temperature, the reaction mixture was diluted with CH_2Cl_2 and washed with brine. The aqueous layer was extracted with CH_2Cl_2 (3×) and the combined organic extracts dried ($MgSO_4$). The solvent was removed under reduced pressure and the residue was purified by flash chromatography on silica, eluting with 15% EtOAc/hexane.

Product Characterisation Data

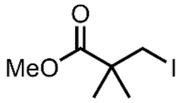

37a

3-Iodo-2,2-dimethylpropionate methyl ester (37a): Obtained as a transparent oil (15 mg, 42%)

^{1}H NMR (400 MHz, $CDCl_3$) δ 3.72 (s, 3H), 3.34 (s, 2H), 1.33 (s, 6H).

^{13}C NMR (100 MHz, $CDCl_3$) δ 175.2, 52.5, 43.6, 25.68, 16.5.

Staring material **37** was also recovered in 58% yield.

References

[1] W.-H. Rao, B.-B. Zhan, K. Chen, P.-X. Ling, Z.-Z. Zhang, B.-F. Shi, *Org. Lett.* **2015**, *17*, 3552-3555.
[2] E. Kerr, E. H. Doeven, G. J. Barbante, C. F. Hogan, D. J. Hayne, P. S. Donnelly, P. S. Francis, *Chem. Sci.* **2016**, *7*, 5271-5279.
[3] N. G. Connelly, W. E. Geiger, *Chem. Rev.* **1996**, *96*, 877-910.
[4] P. T. Kissinger, W. R. Heineman, *J. Chem. Educ.* **1983**, *60*, 702.
[5] M. J. G. R. Frisch, G. Trucks, H. B. Schlegel, G. E. Scuseria, M. Robb, J. Cheeseman, G. Scalmani, V. Barone, B. Mennuci, G. A. Petersson, H. Nakatsuji, M. Caricato, X. Li, H. P. Hratchian, A. F. Izmaylov, J. Bloino, G. Zheng, J. L. Sonnenberg, M. Hada, D. Fox, **2010**.
[6] a) J. P. Perdew, *Phys. Rev. B* **1986**, *33*, 8822-8824; b) A. D. Becke, *Phys. Rev. A* **1988**, *38*, 3098-3100; c) L. Chen, E. H. Doeven, D. J. D. Wilson, E. Kerr, D. J. Hayne, C. F. Hogan, W. Yang, T. T. Pham, P. S. Francis, *ChemElectroChem* **2017**, *4*, 1797-1808.
[7] B. J. Shields, B. Kudisch, G. D. Scholes, A. G. Doyle, *J. Am. Chem. Soc.* **2018**, *140*, 3035-3039.
[8] X. Yang, Y. Sun, T.-y. Sun, Y. Rao, *Chem. Commun.* **2016**, *52*, 6423-6426.
[9] G. Sheldrick, *Acta Cryst C* **2015**, *71*, 3-8.

^{1}H and ^{13}C NMR Spectra

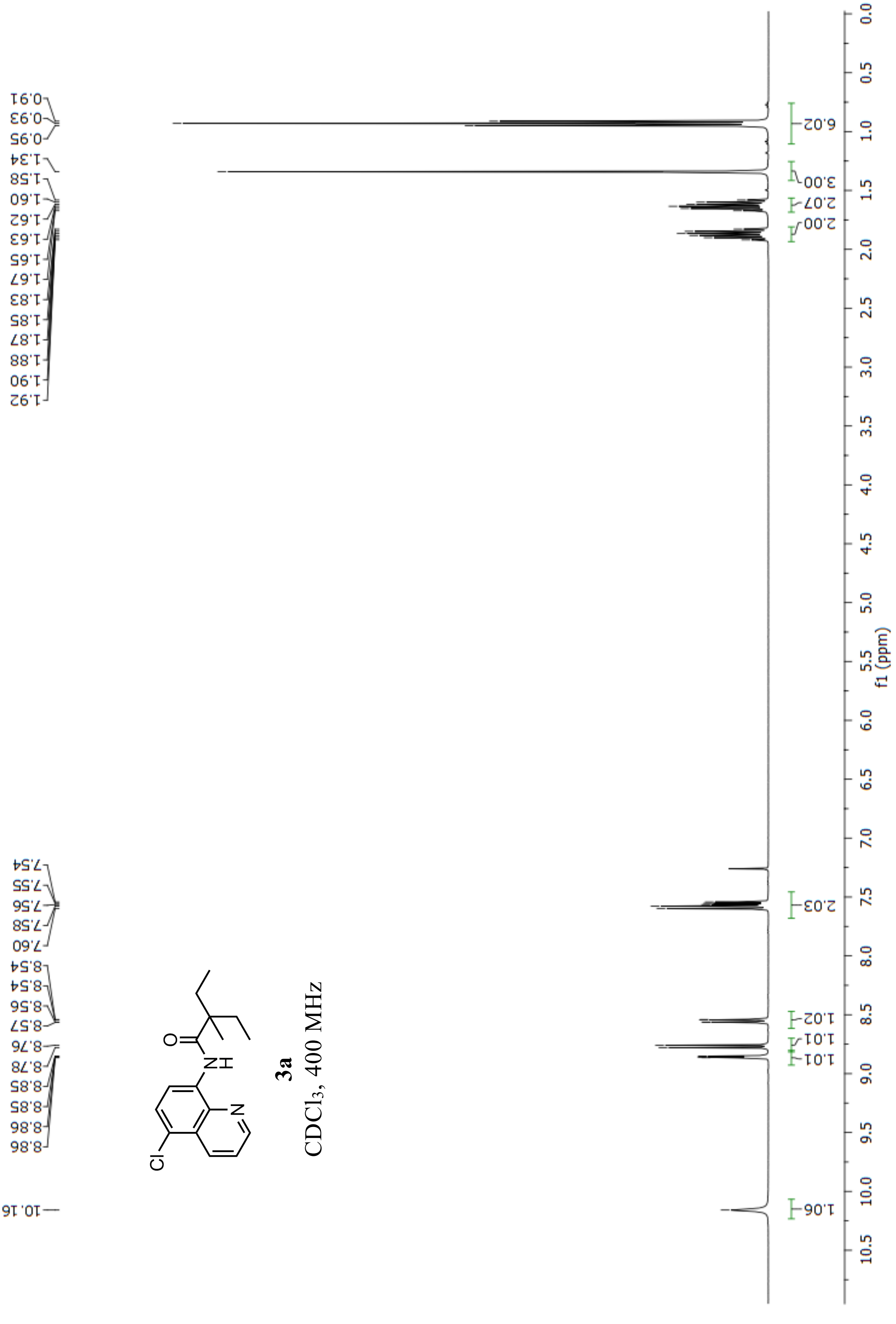

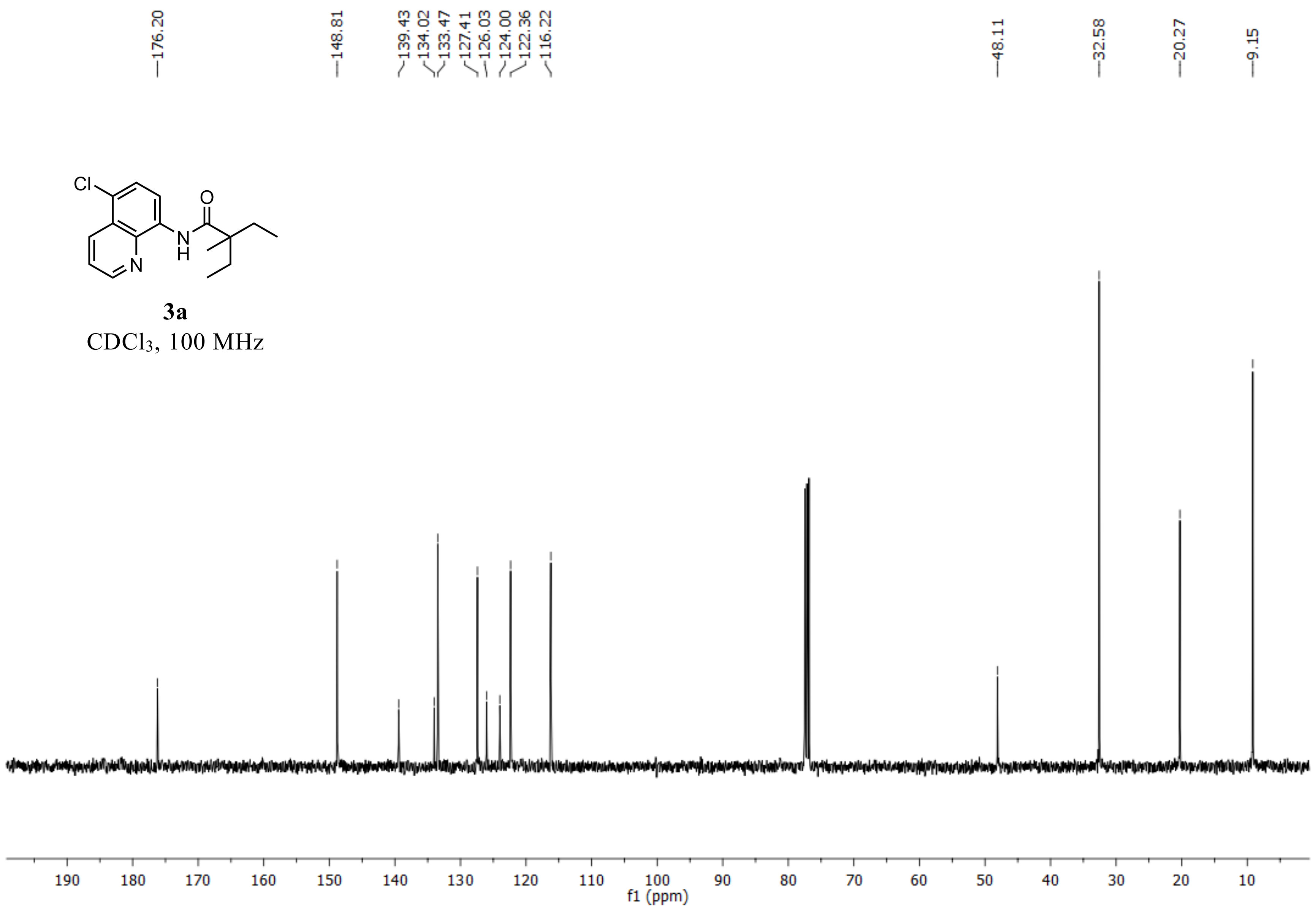

3a

$CDCl_3$, 100 MHz

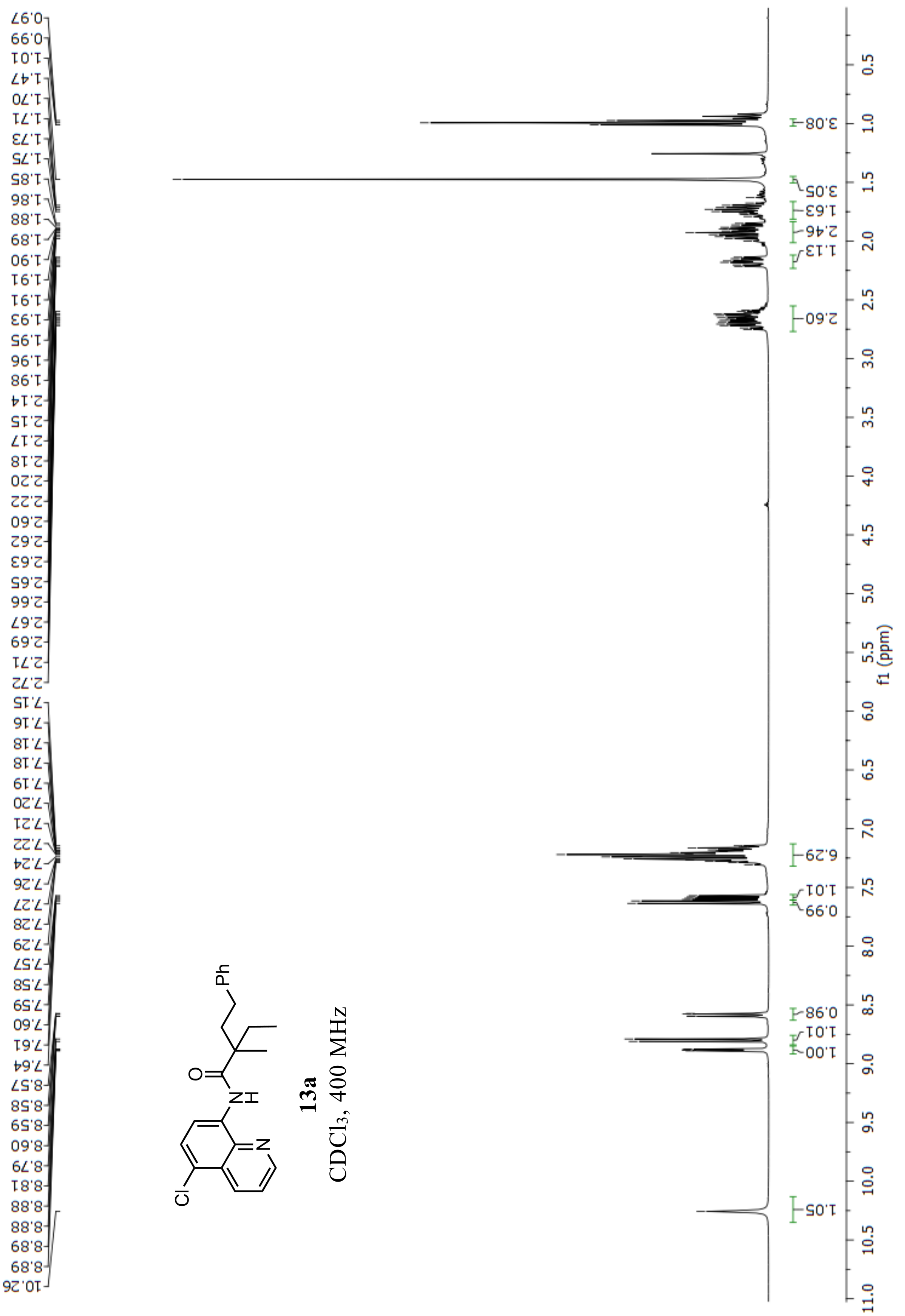
13a
CDCl3, 400 MHz
f1 (ppm)

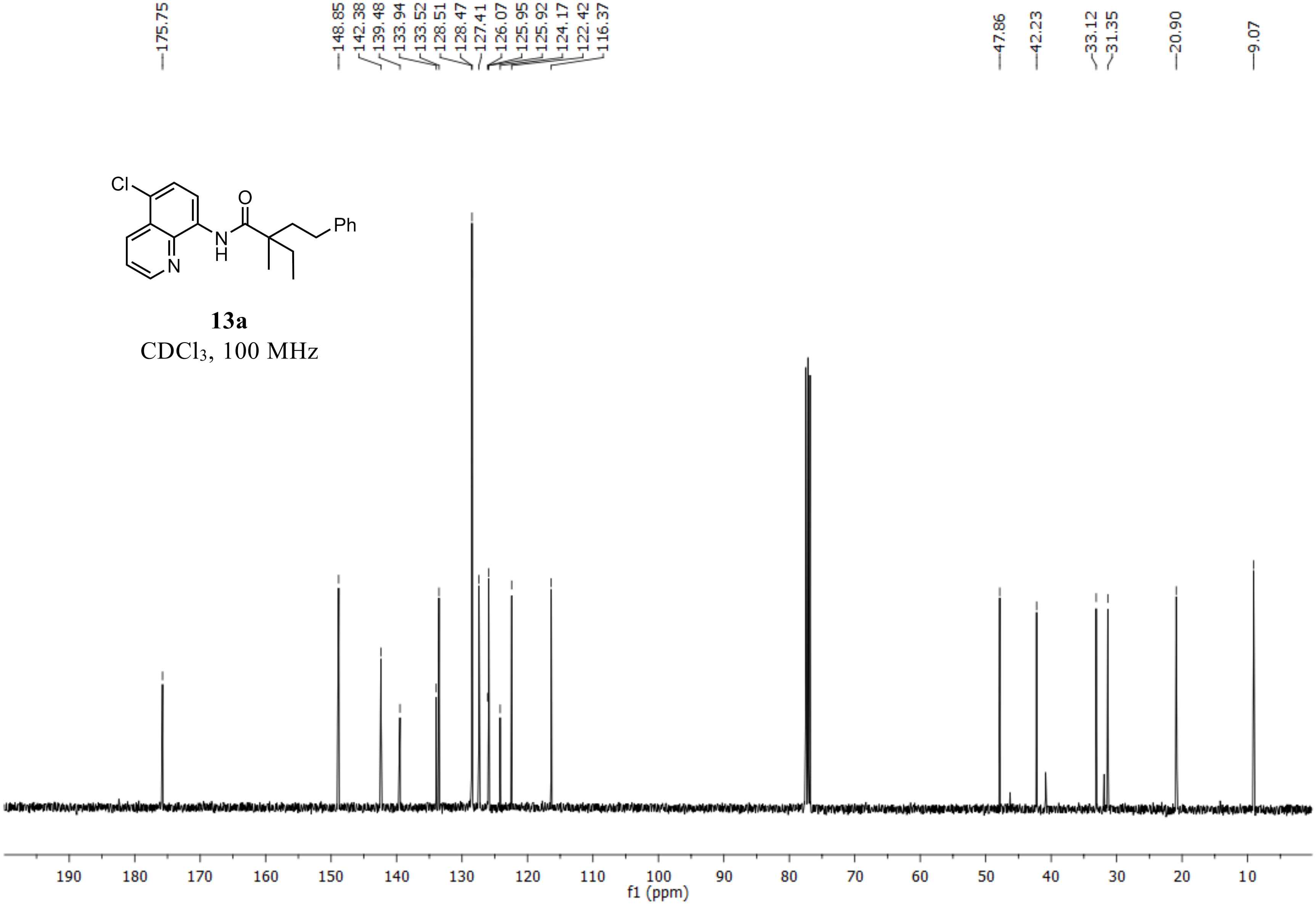

13a

$CDCl_3$, 100 MHz

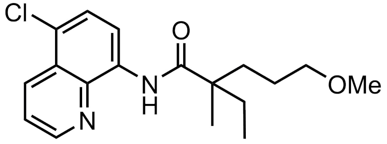

14a

$CDCl_3$, 400 MHz

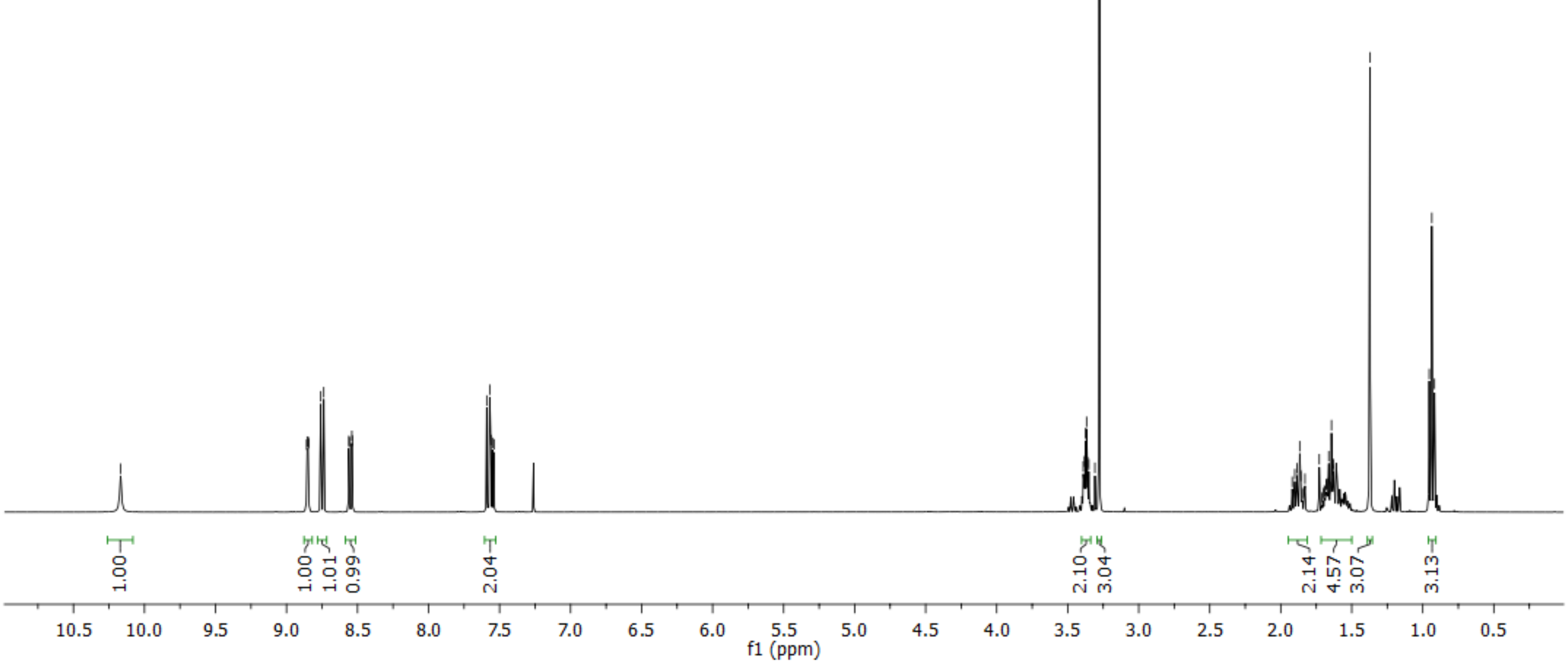

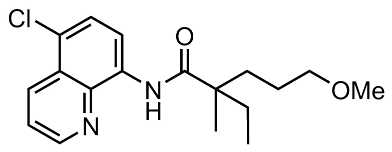

14a

$CDCl_3$, 100 MHz

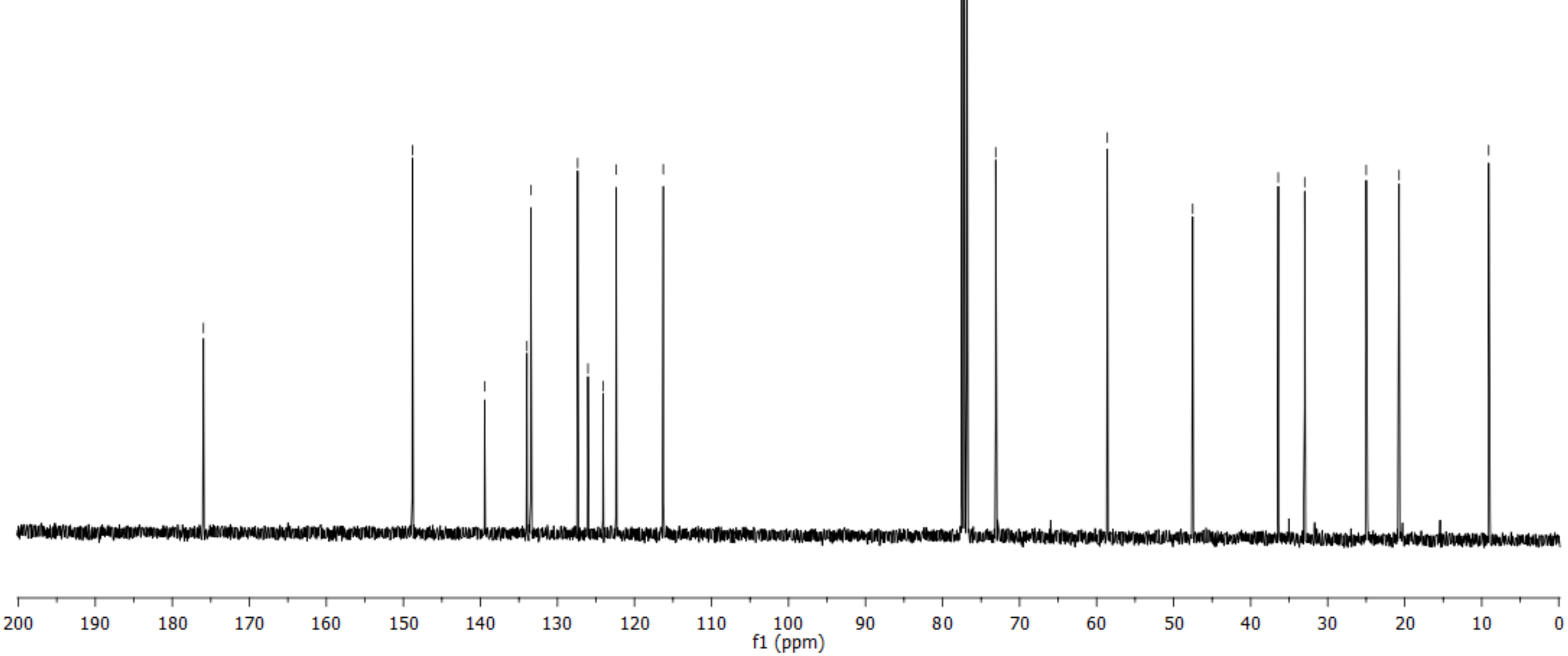

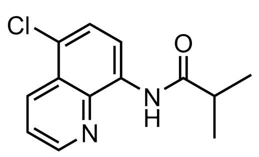

15a

$CDCl_3$, 400 MHz

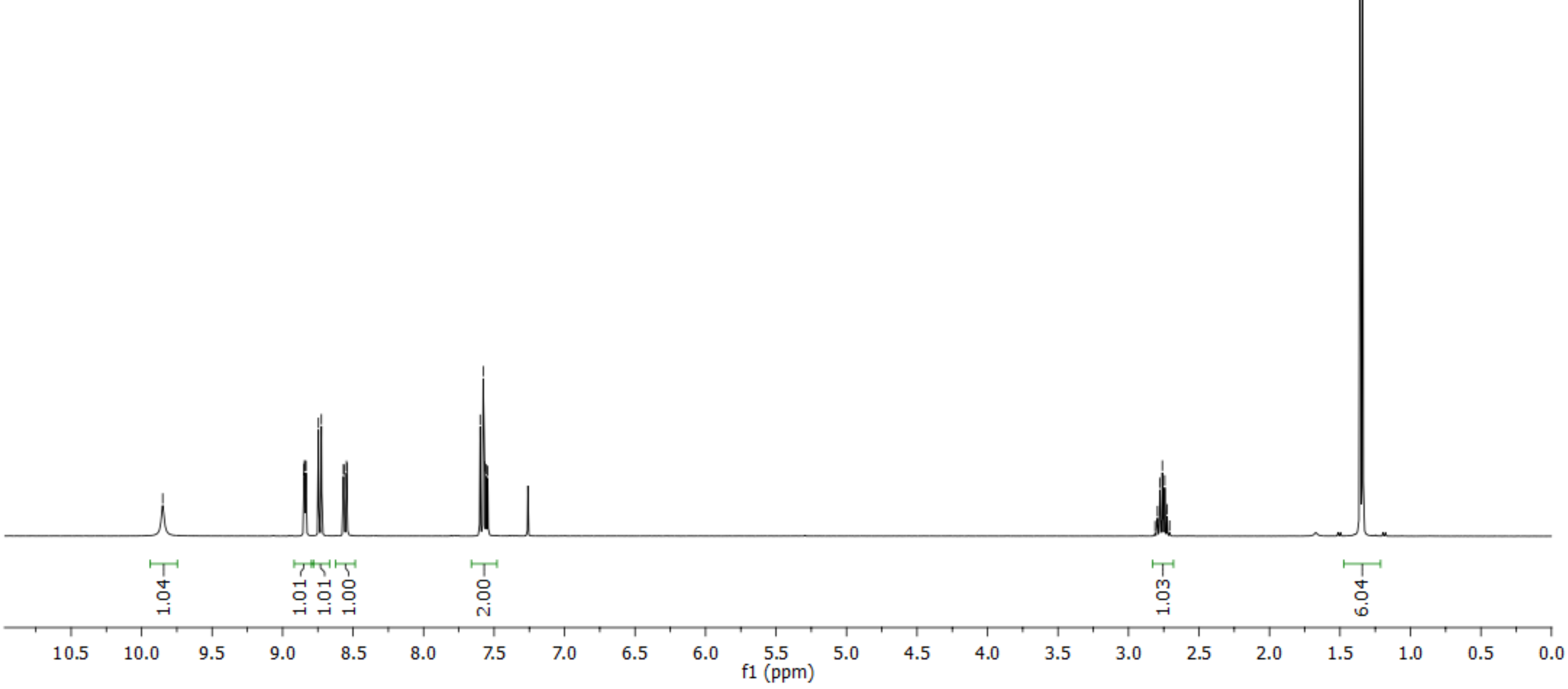

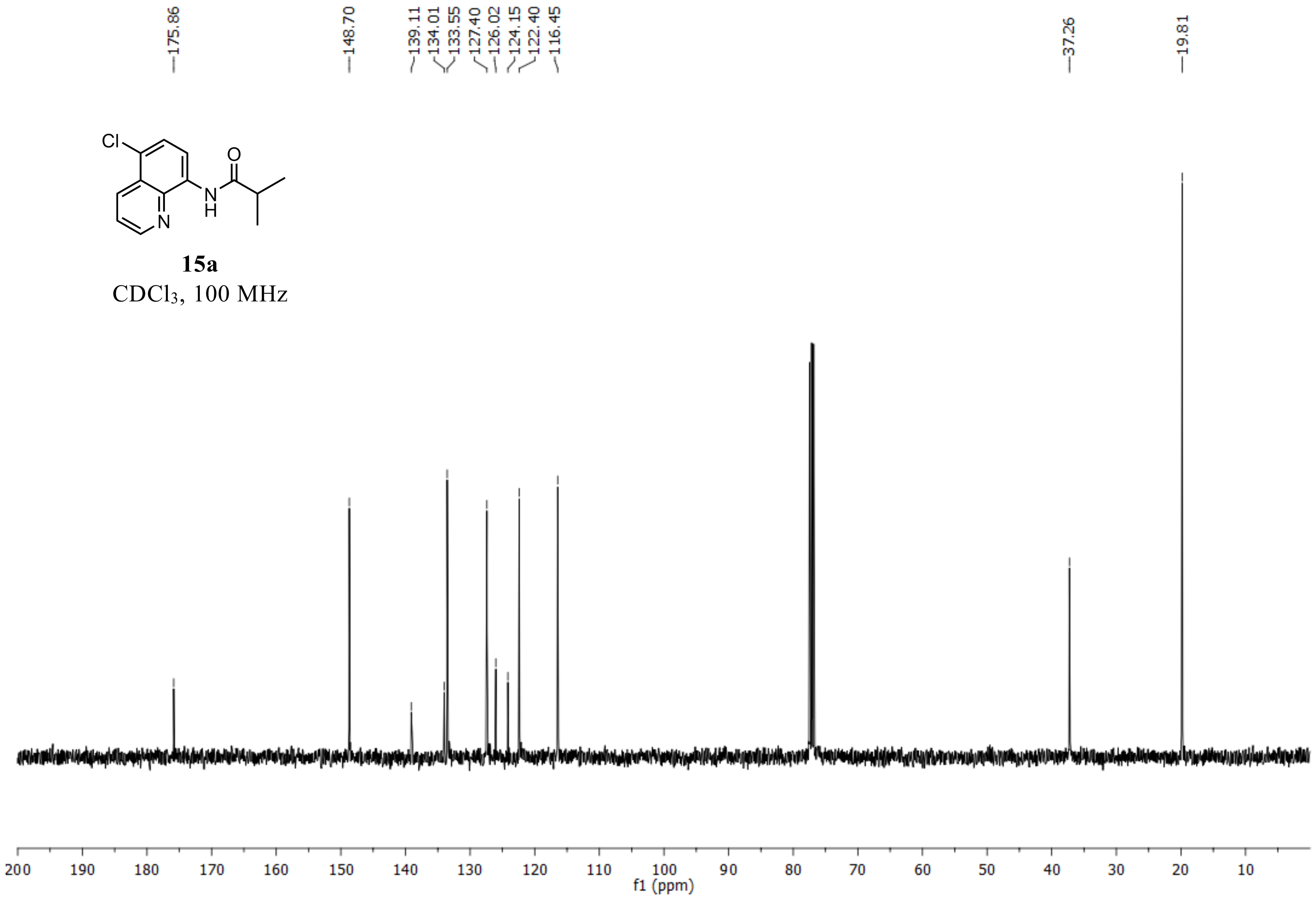

175.86
148.70
139.11
134.01
133.55
127.40
126.02
124.15
122.40
116.45
37.26
19.81
Cl
O
N
H
N
15a
CDCl3, 100 MHz
200 190 180 170 160 150 140 130 120 110 100 90 80 70 60 50 40 30 20 10
f1 (ppm)

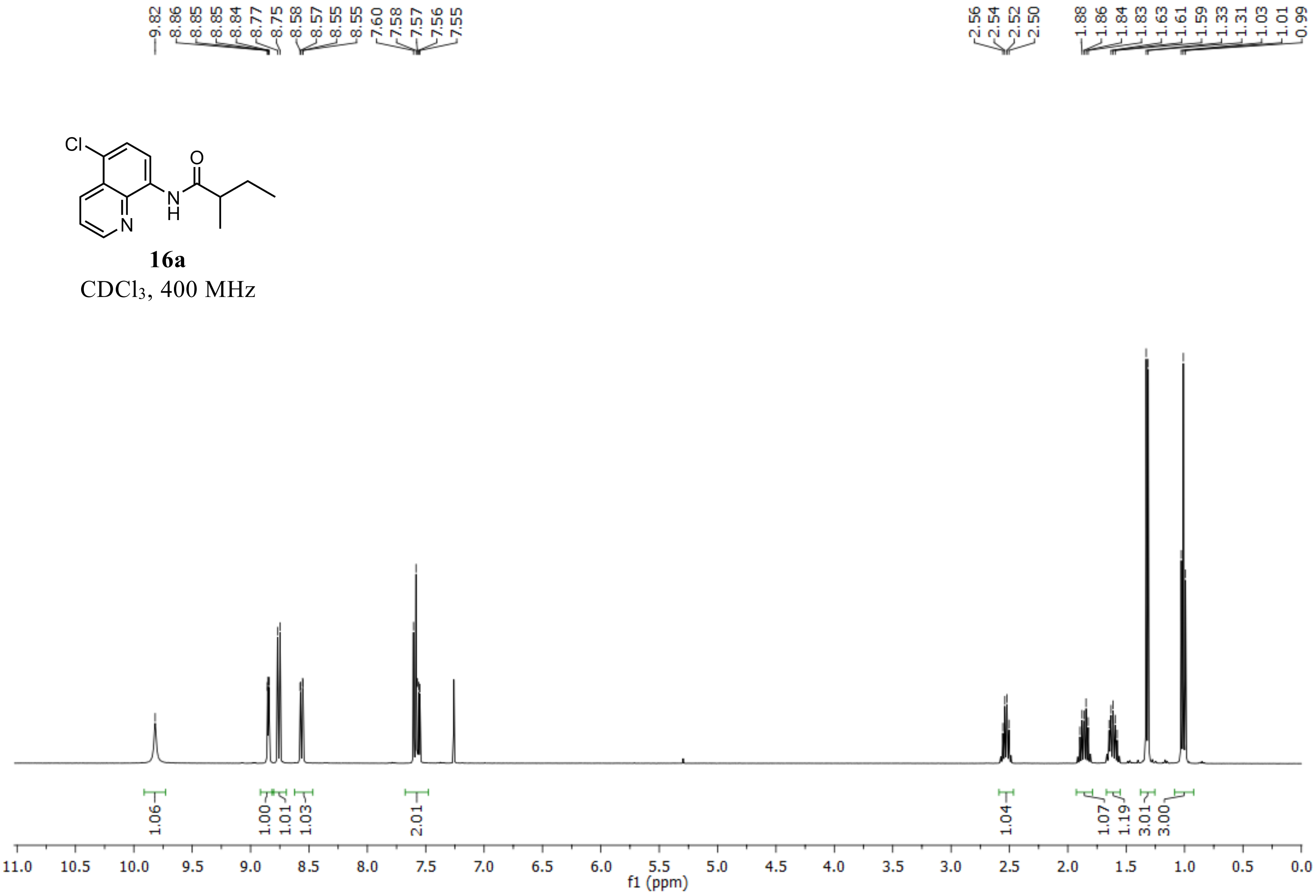

16a
$CDCl_3$, 400 MHz

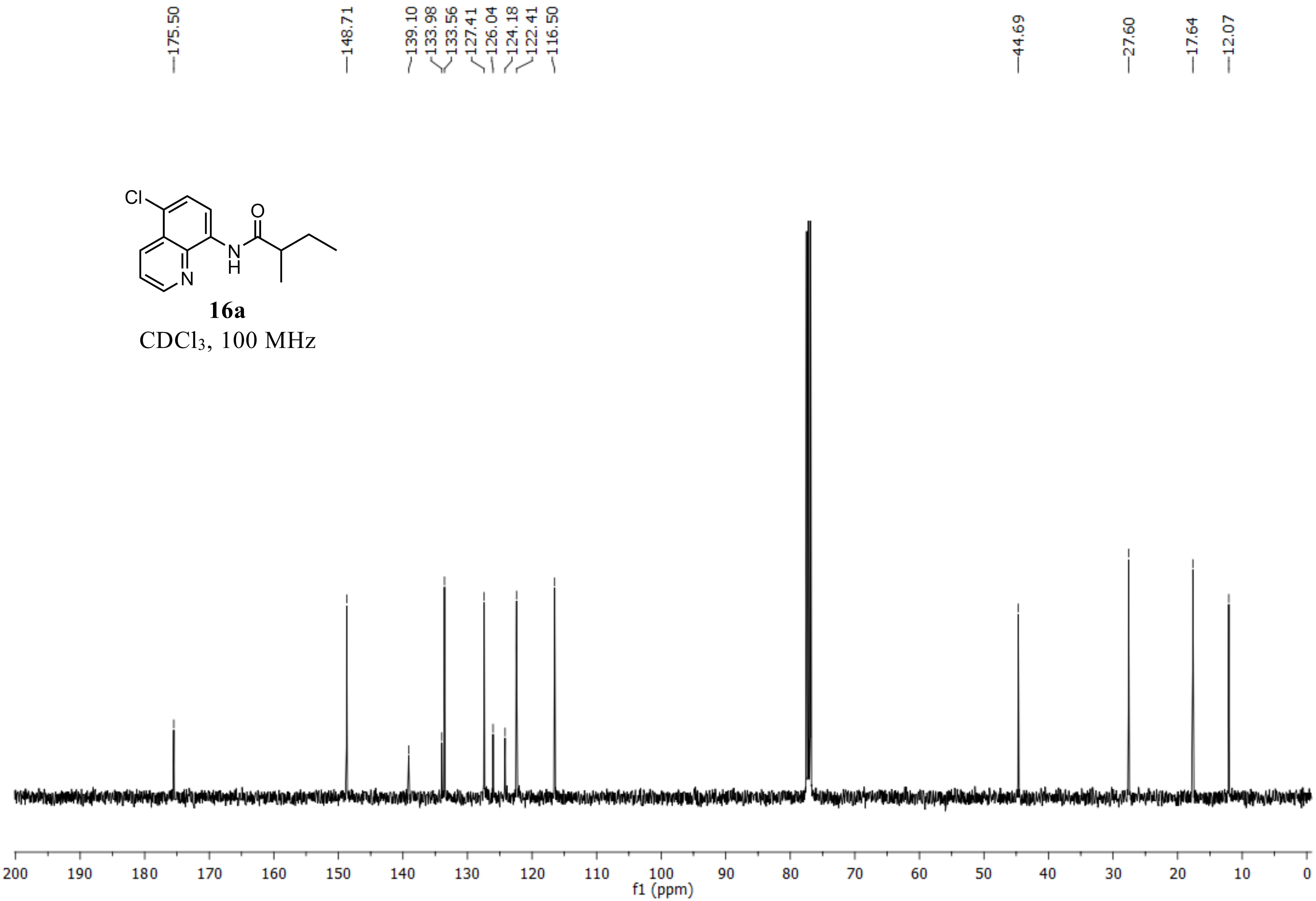

16a
$CDCl_3$, 100 MHz

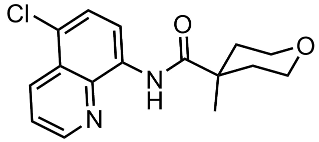

17a
CDCl$_3$, 400 MHz

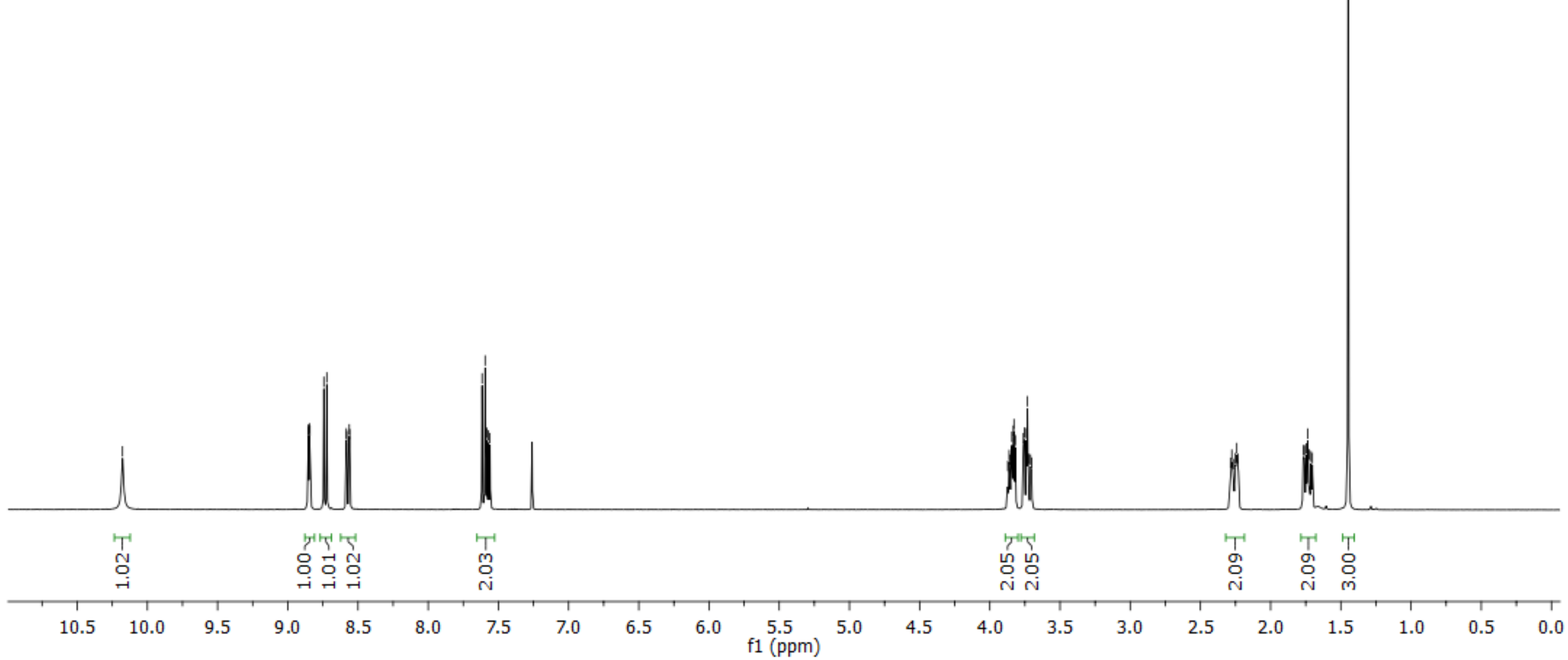

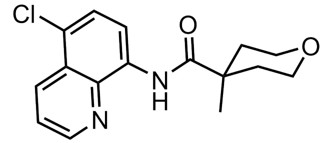

17a
$CDCl_3$, 100 MHz

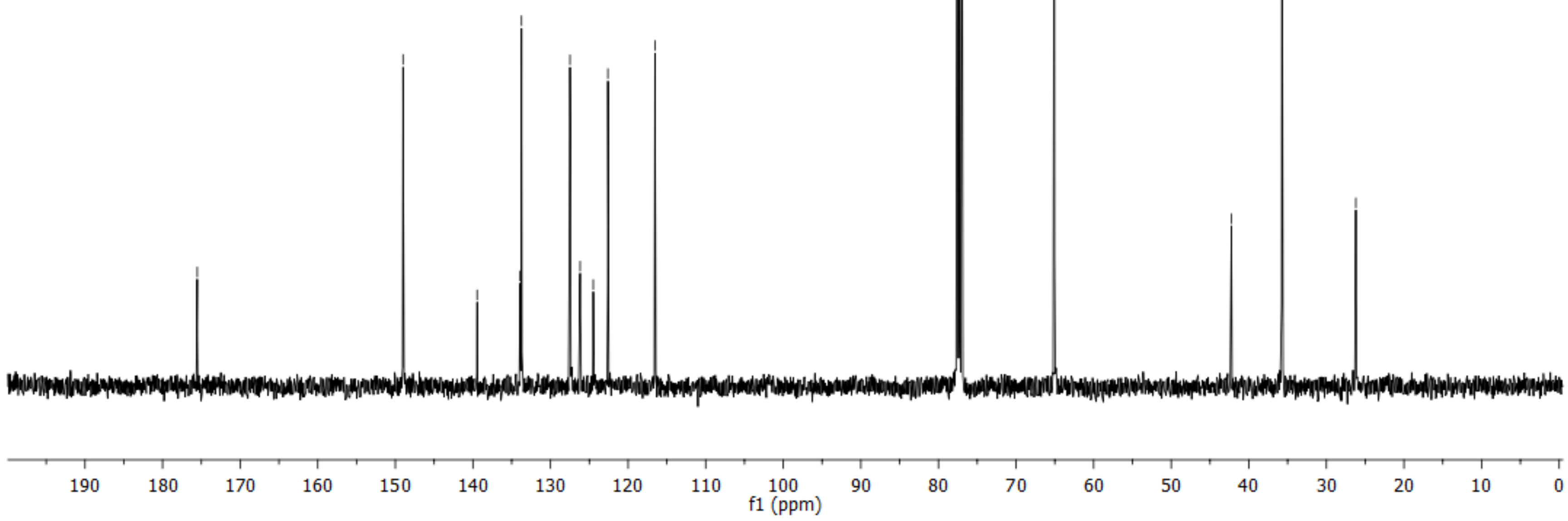

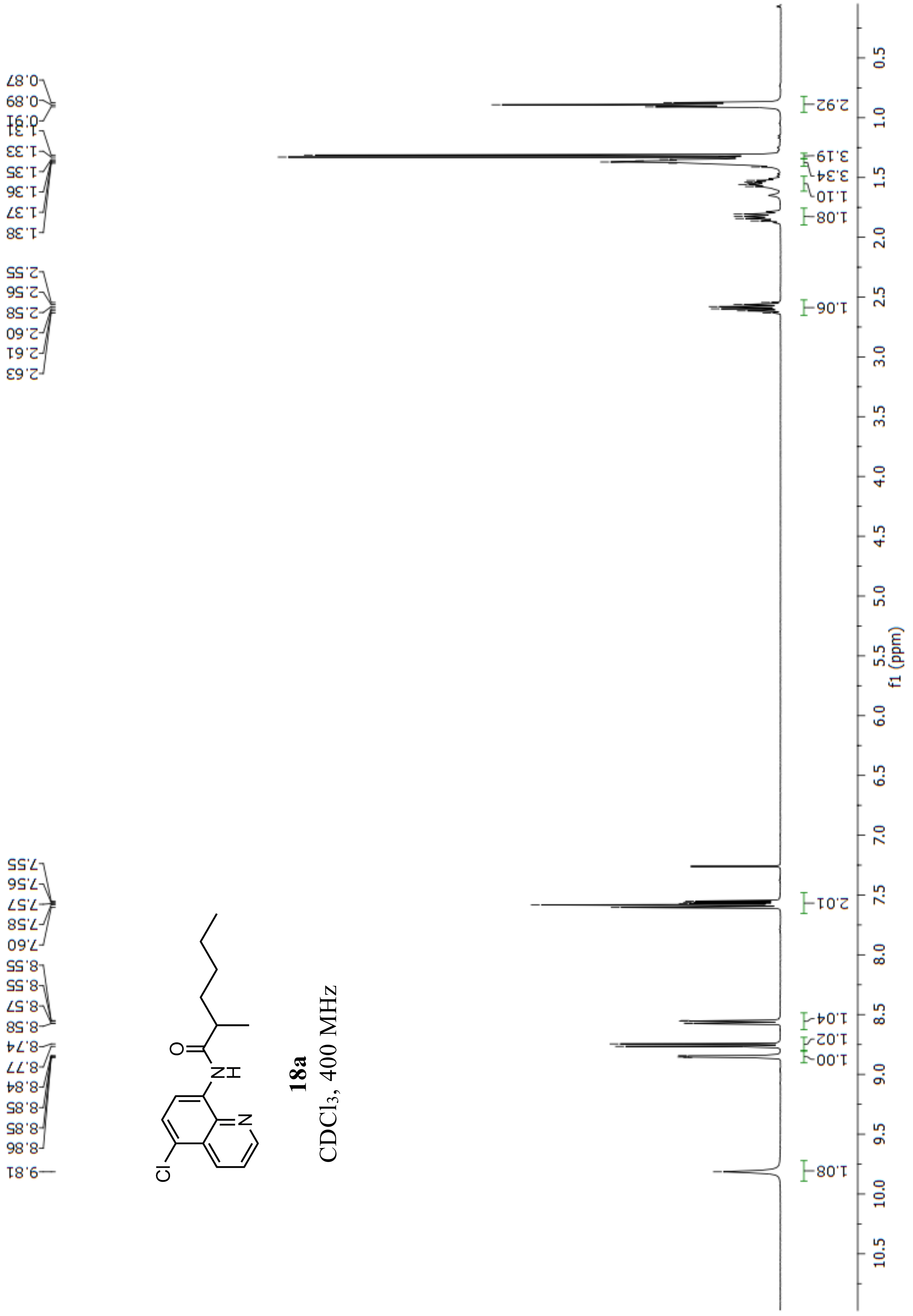

18a
$CDCl_3$, 400 MHz

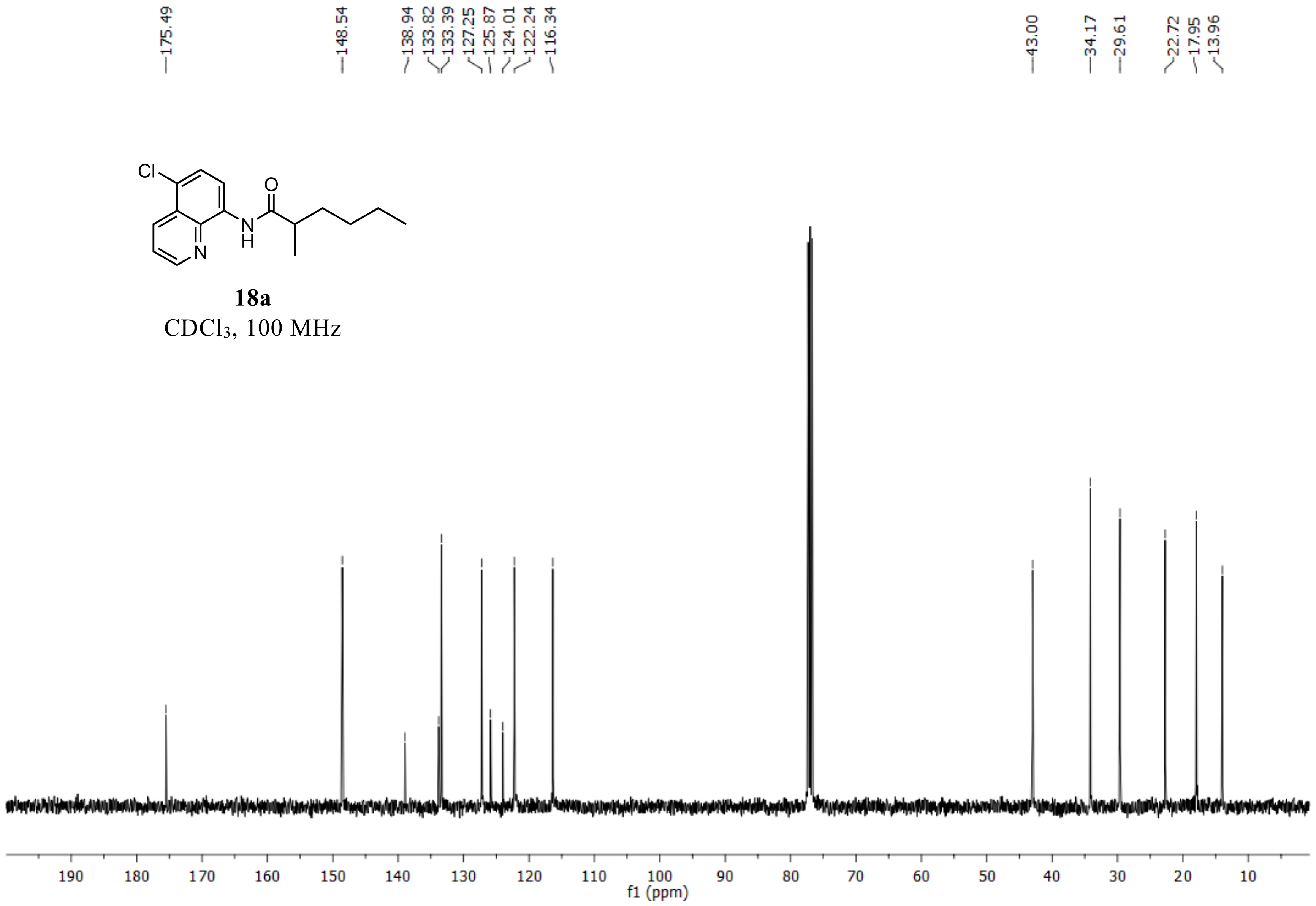

18a
$CDCl_3$, 100 MHz

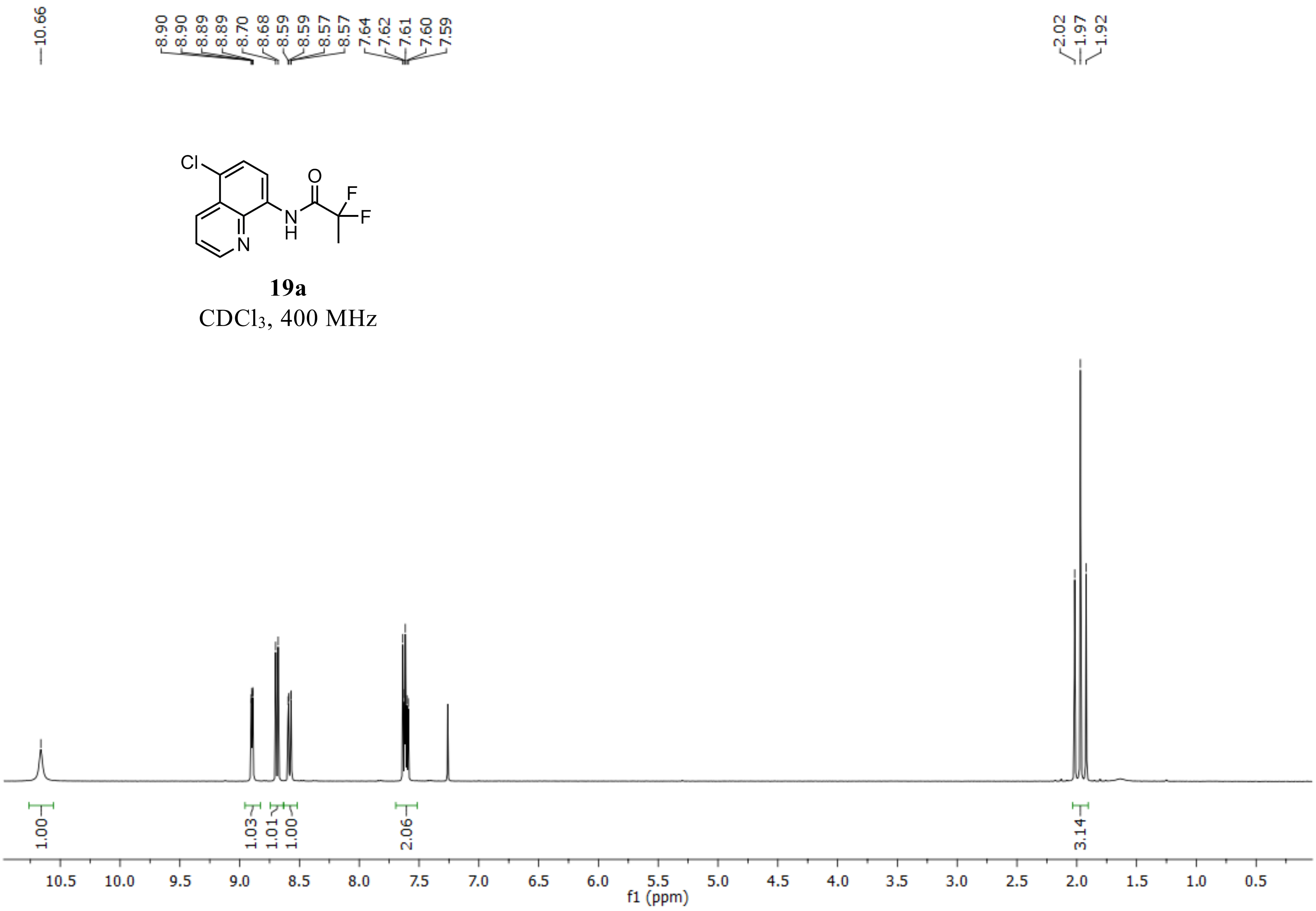

19a

$CDCl_3$, 400 MHz

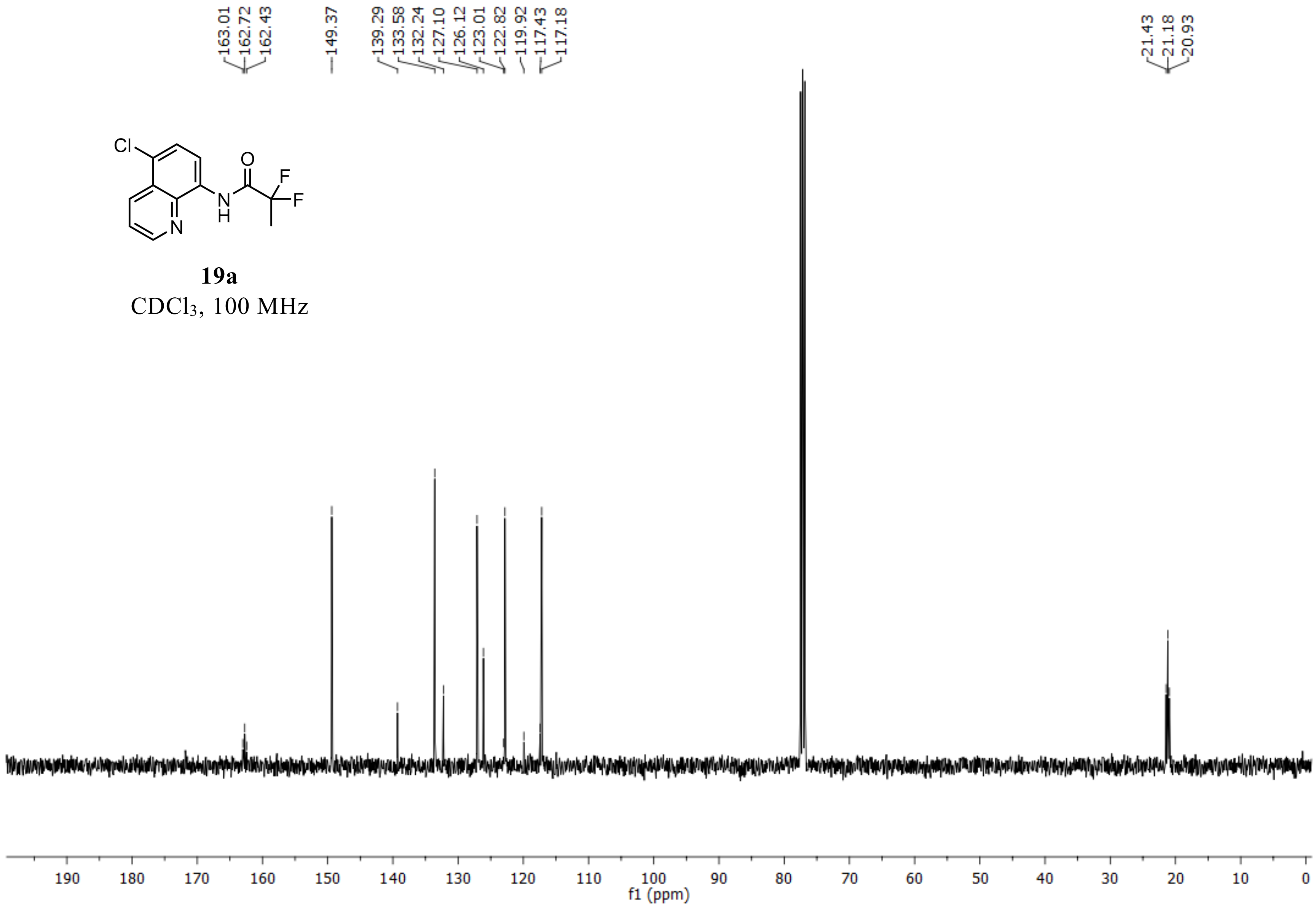

19a
$CDCl_3$, 100 MHz

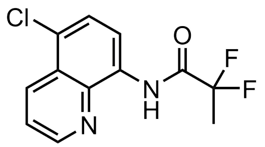

19a
$CDCl_3$, 376 MHz

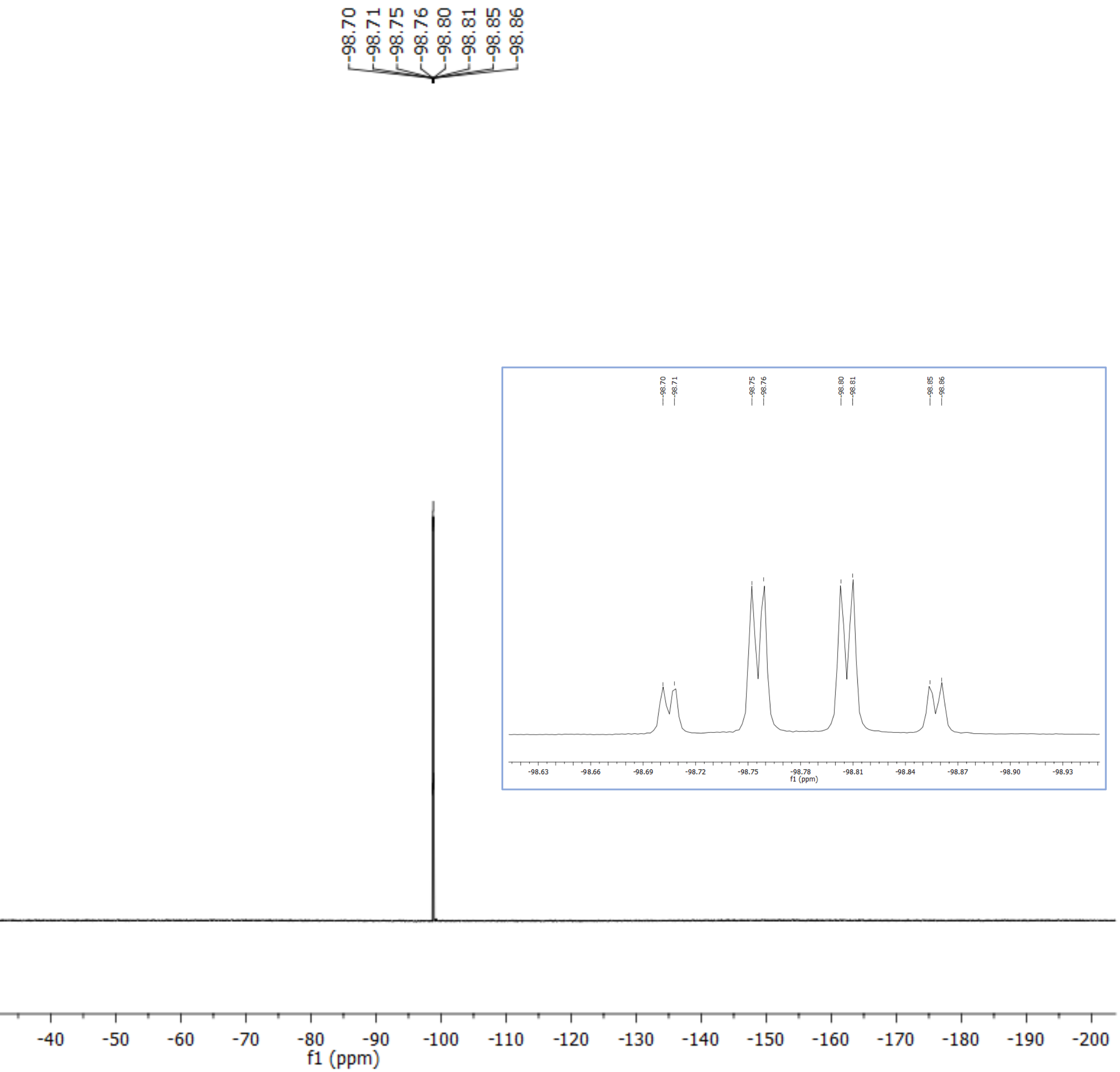

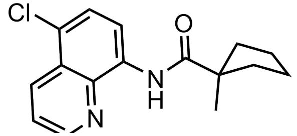

20a

$CDCl_3$, 400 MHz

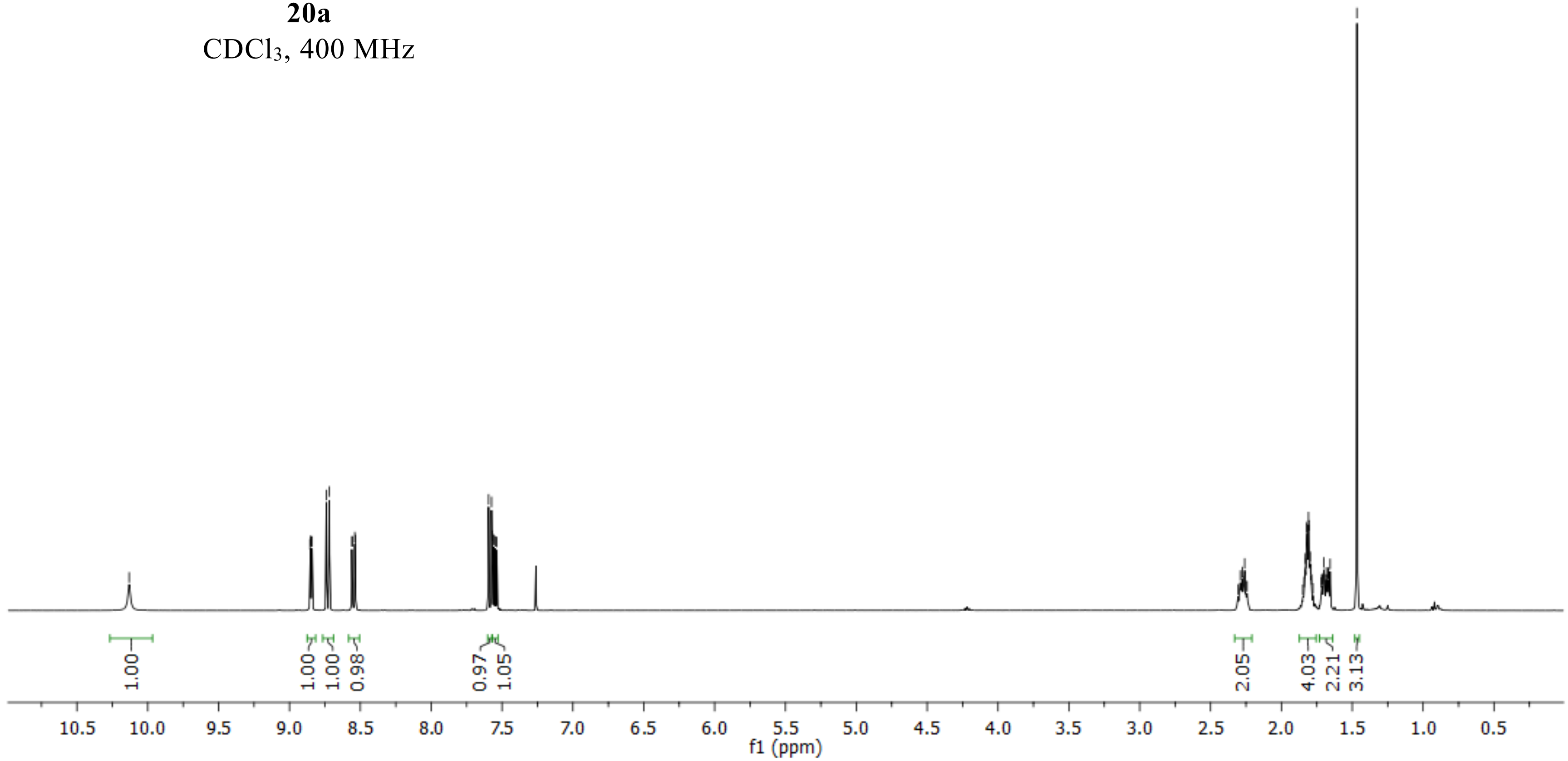

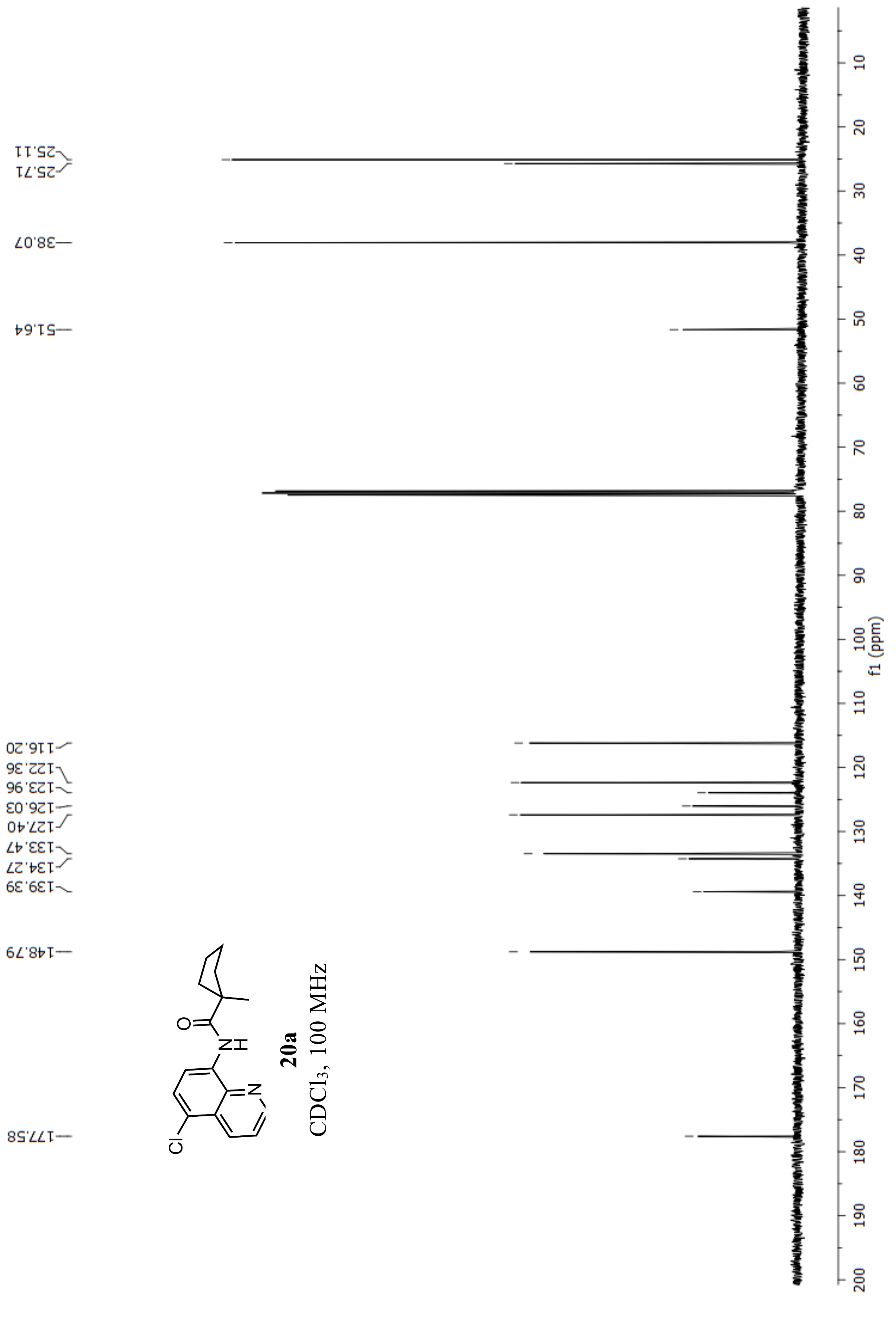
25.11
25.71
38.07
51.64
116.20
122.36
123.96
126.03
127.40
133.47
134.27
139.39
148.79
177.58
Cl
N
N
H
O
20a
CDCl3, 100 MHz
f1 (ppm)

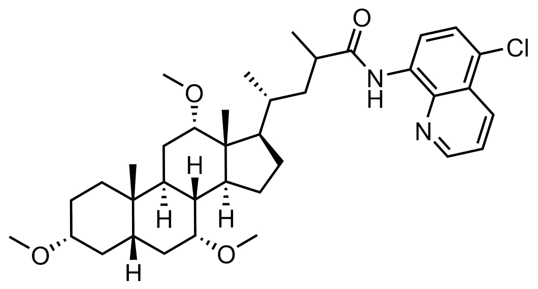

21a

$CDCl_3$, 400 MHz

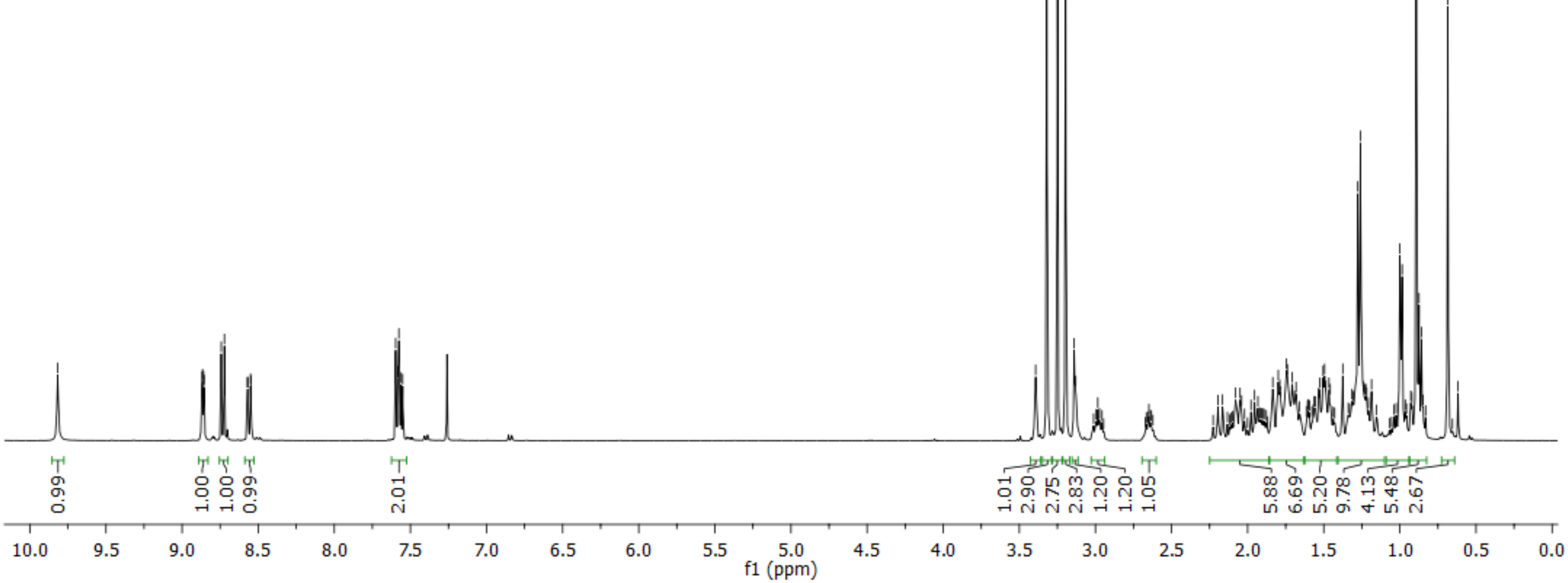

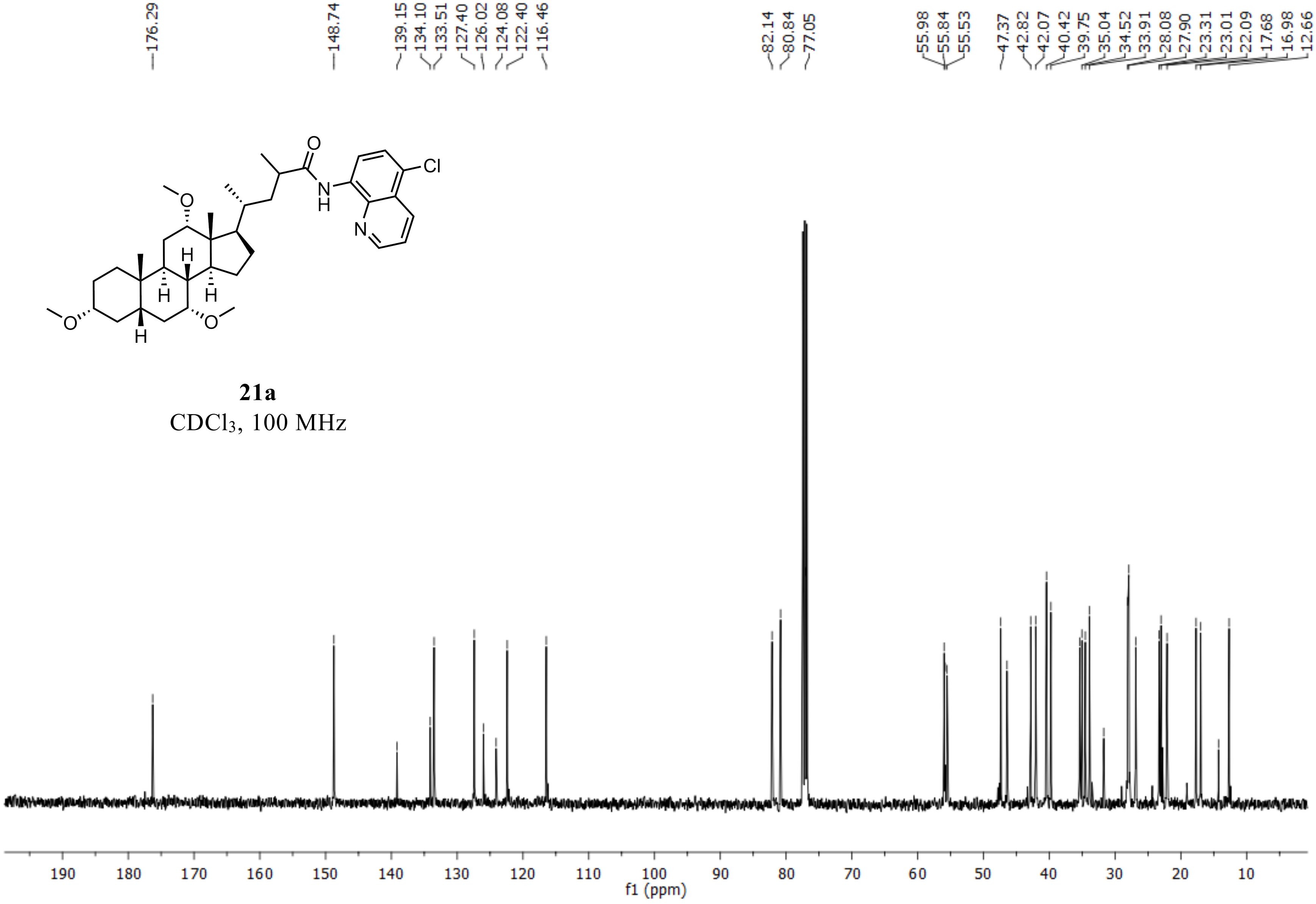

21a
$CDCl_3$, 100 MHz

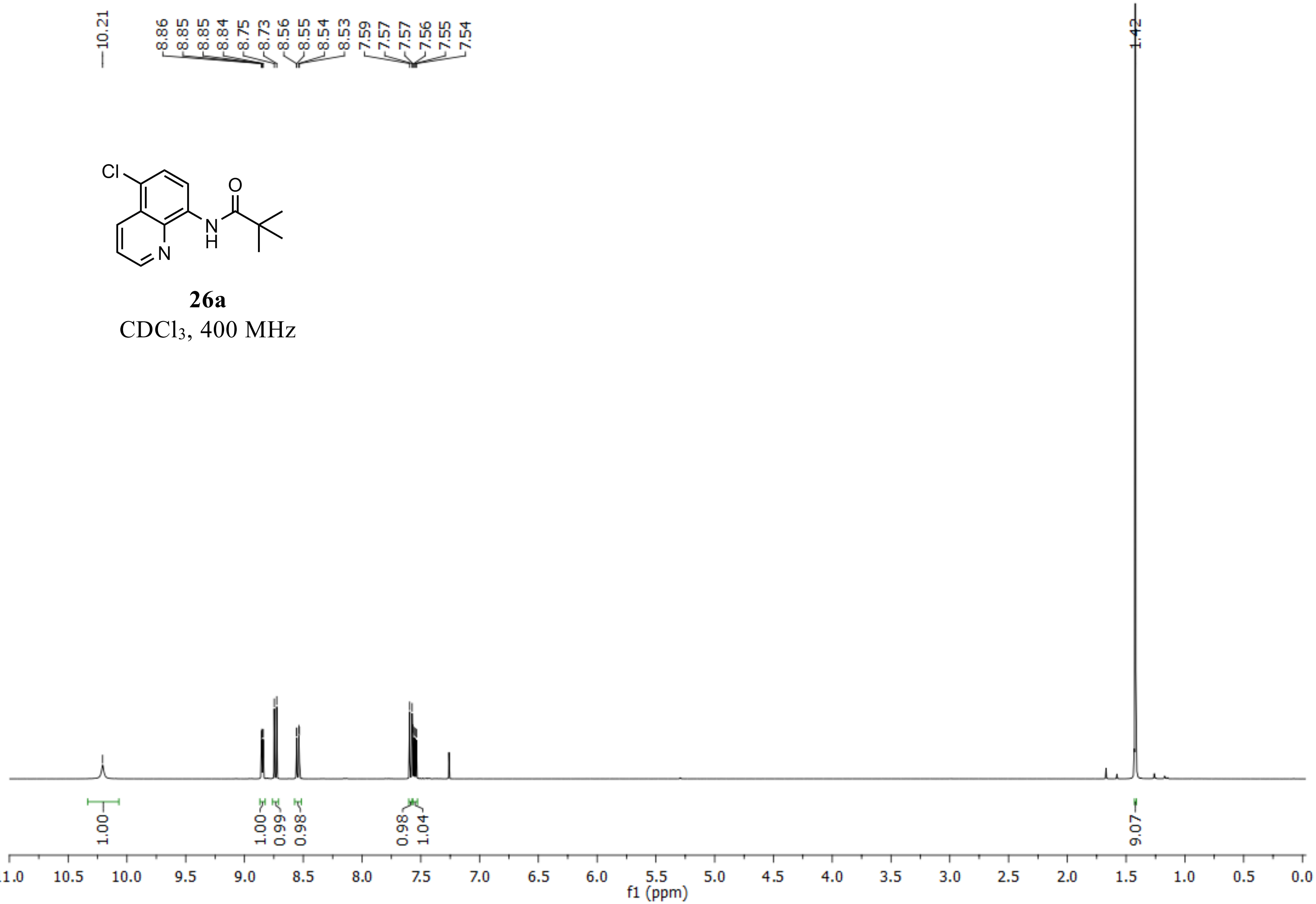

26a

$CDCl_3$, 400 MHz

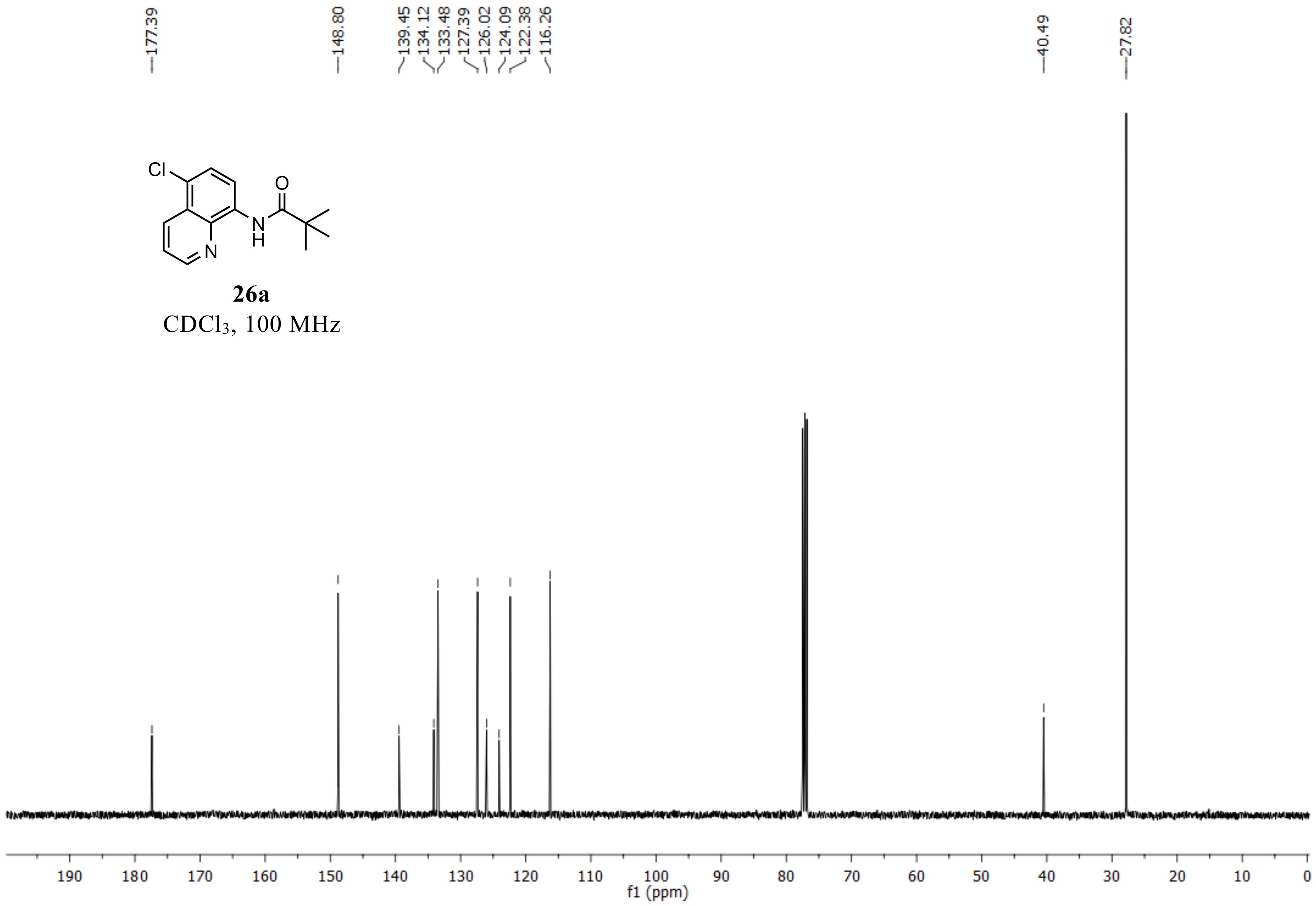

26a

$CDCl_3$, 100 MHz

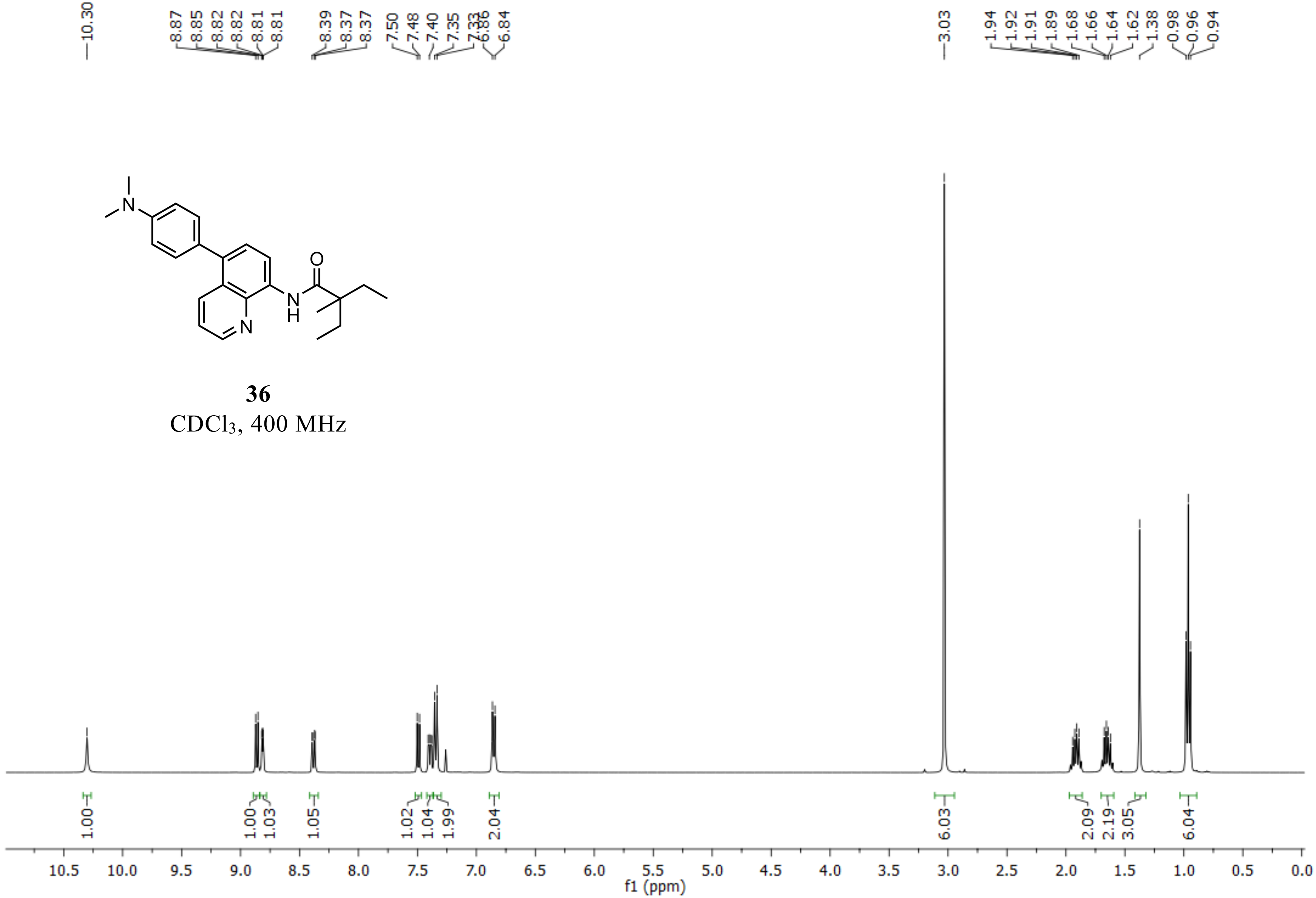

36
$CDCl_3$, 400 MHz

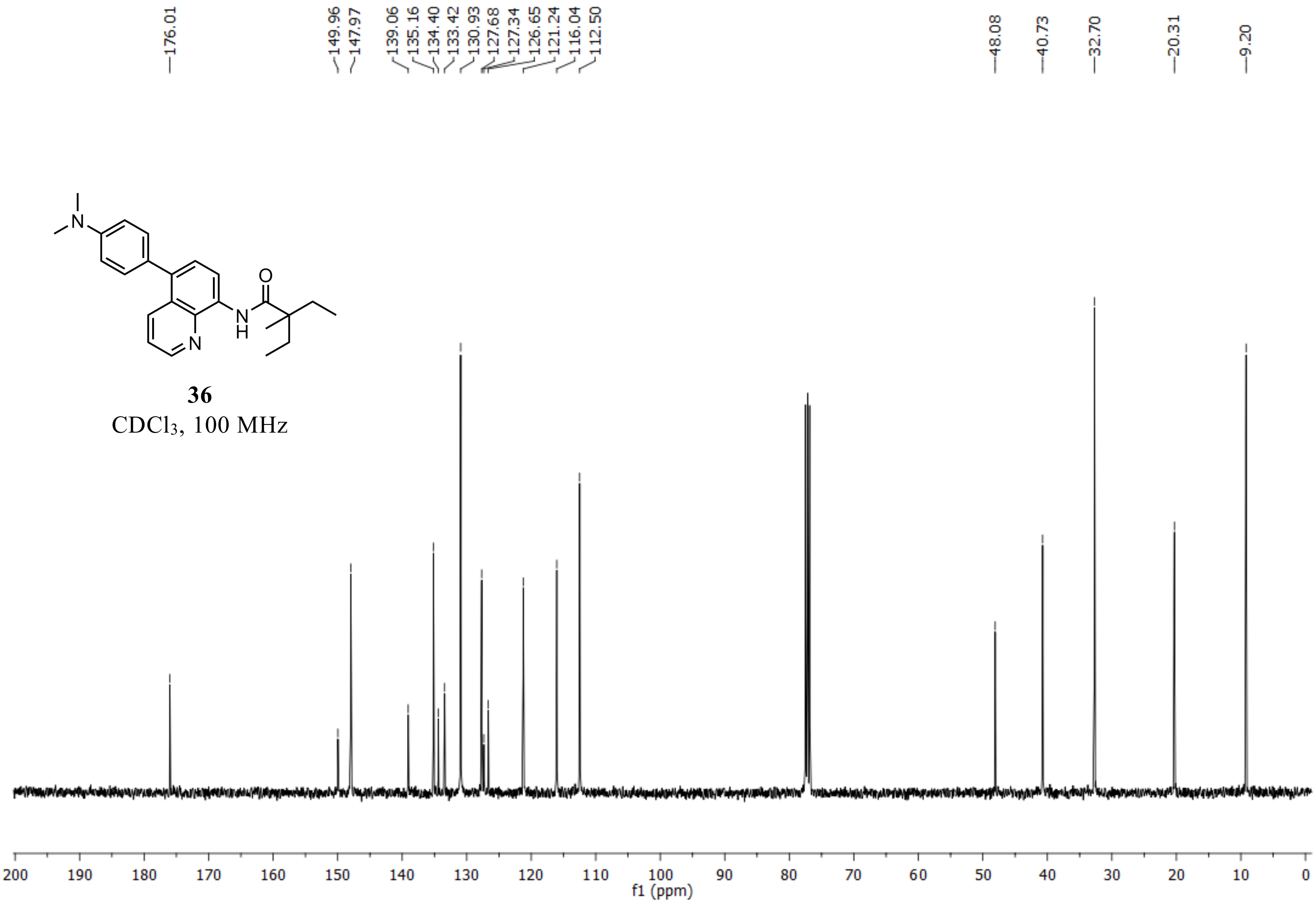

36

$CDCl_3$, 100 MHz

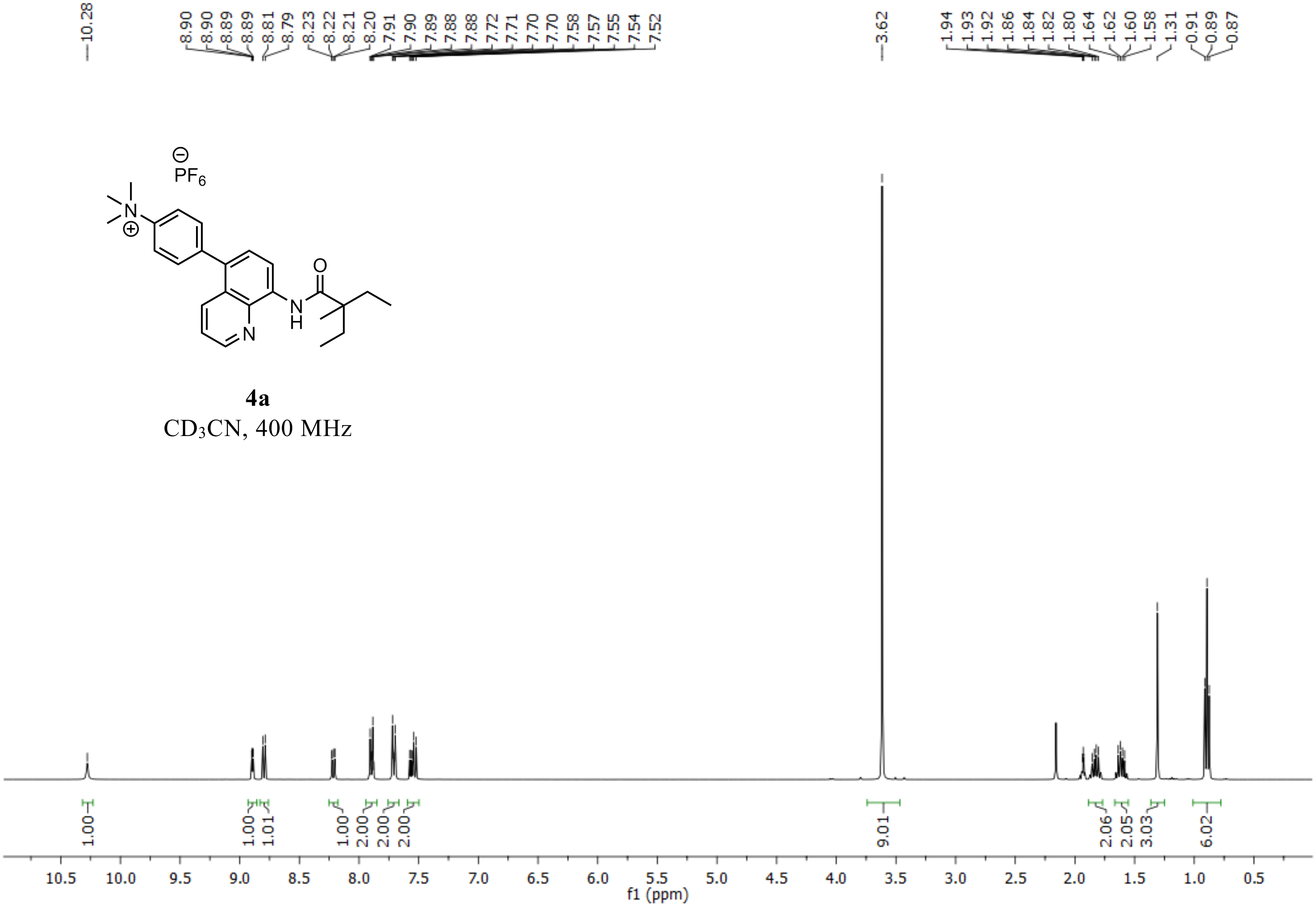

4a

CD_3CN, 400 MHz

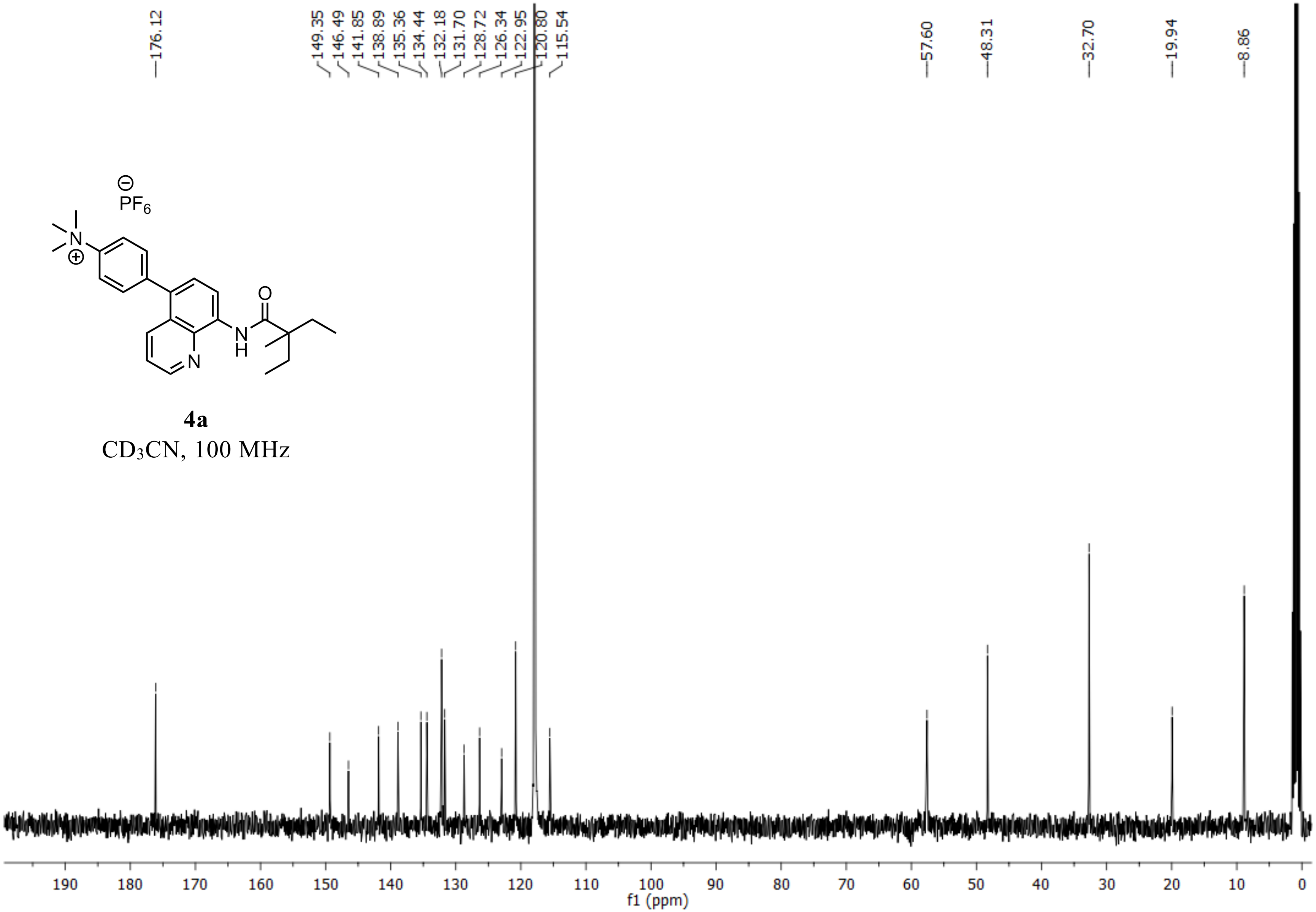

4a

CD_3CN, 100 MHz

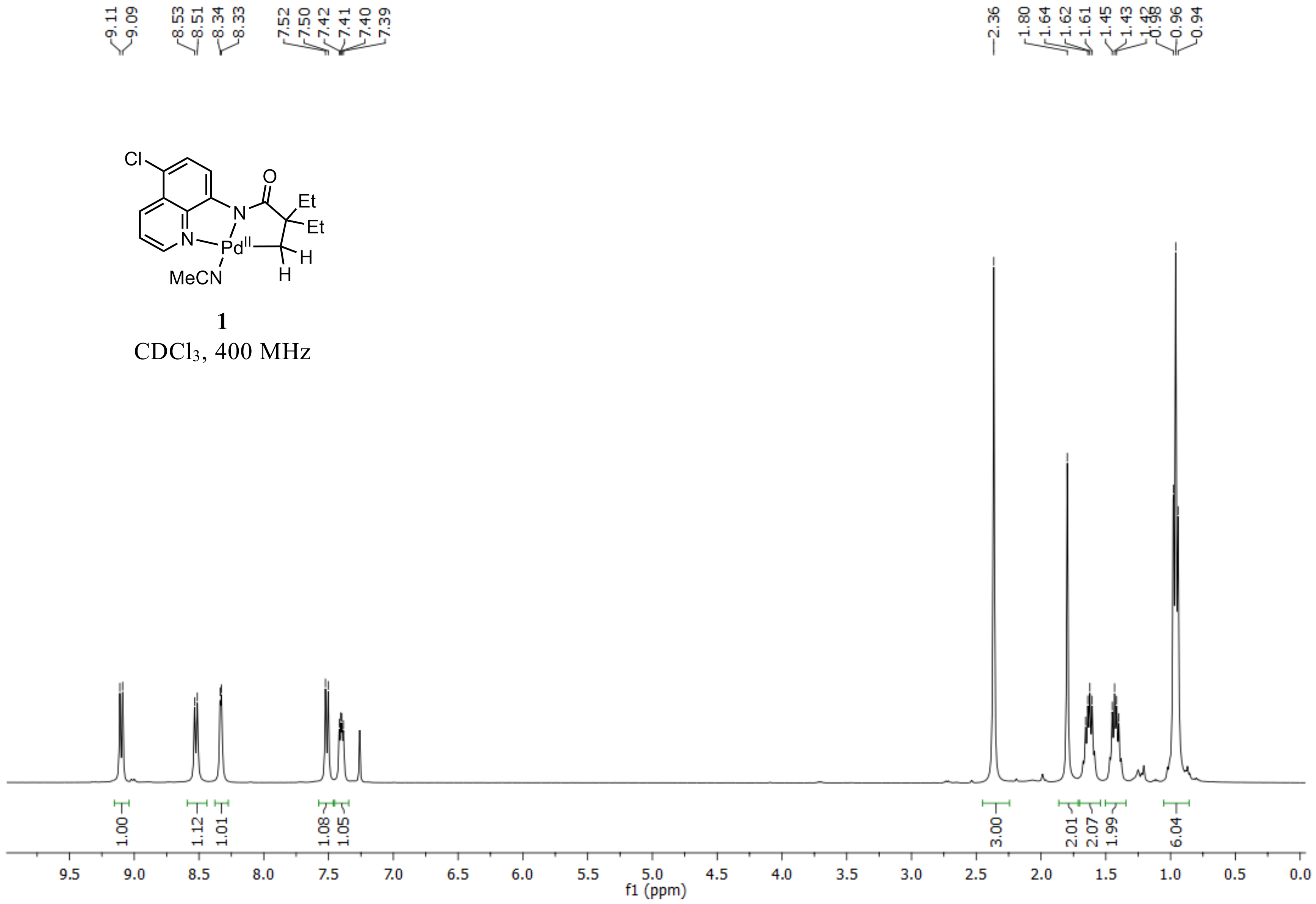

1

$CDCl_3$, 400 MHz

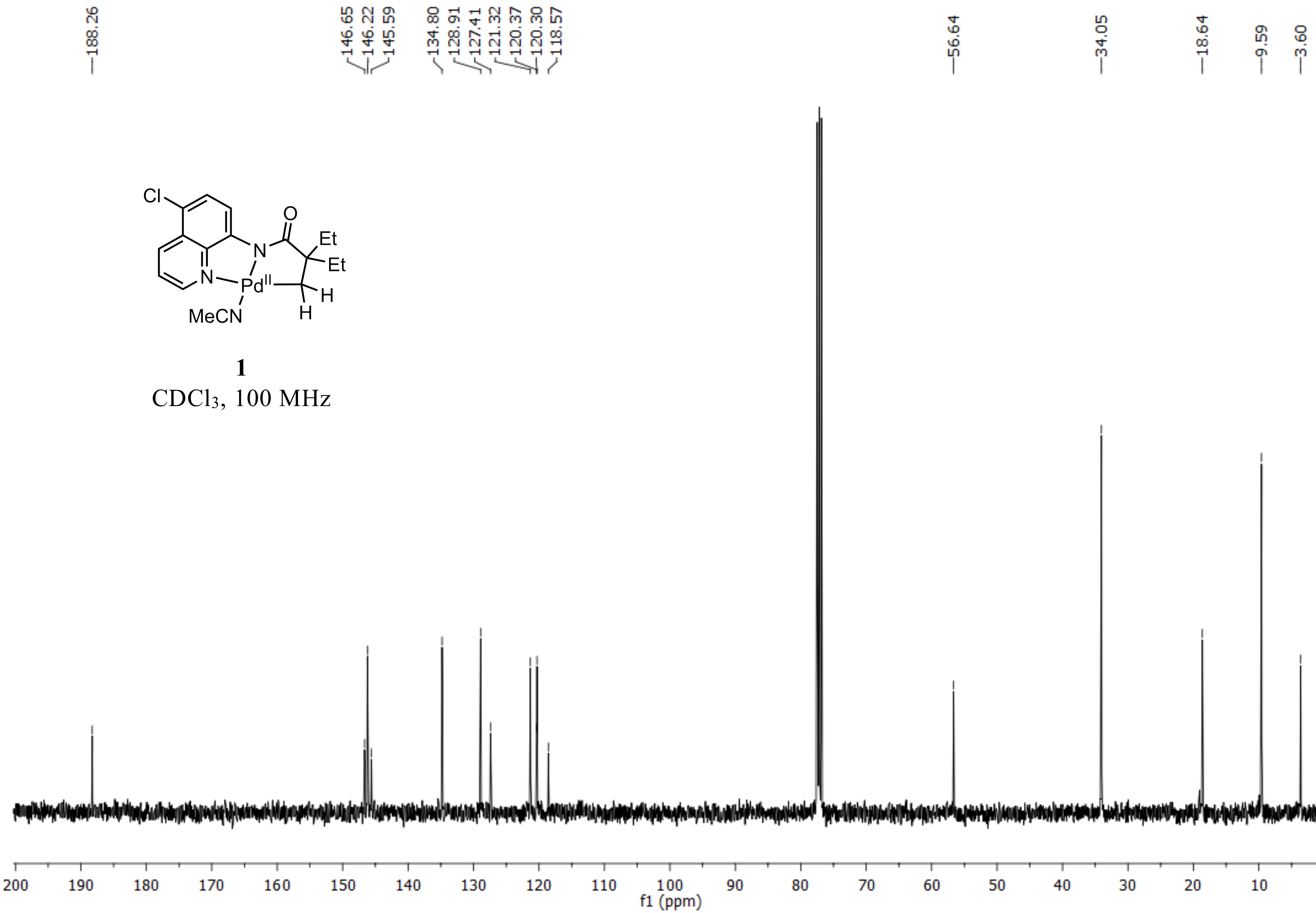

1

$CDCl_3$, 100 MHz

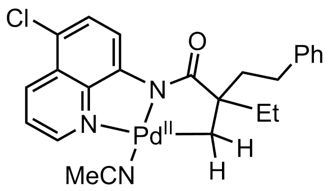

13b

$CDCl_3$, 400 MHz

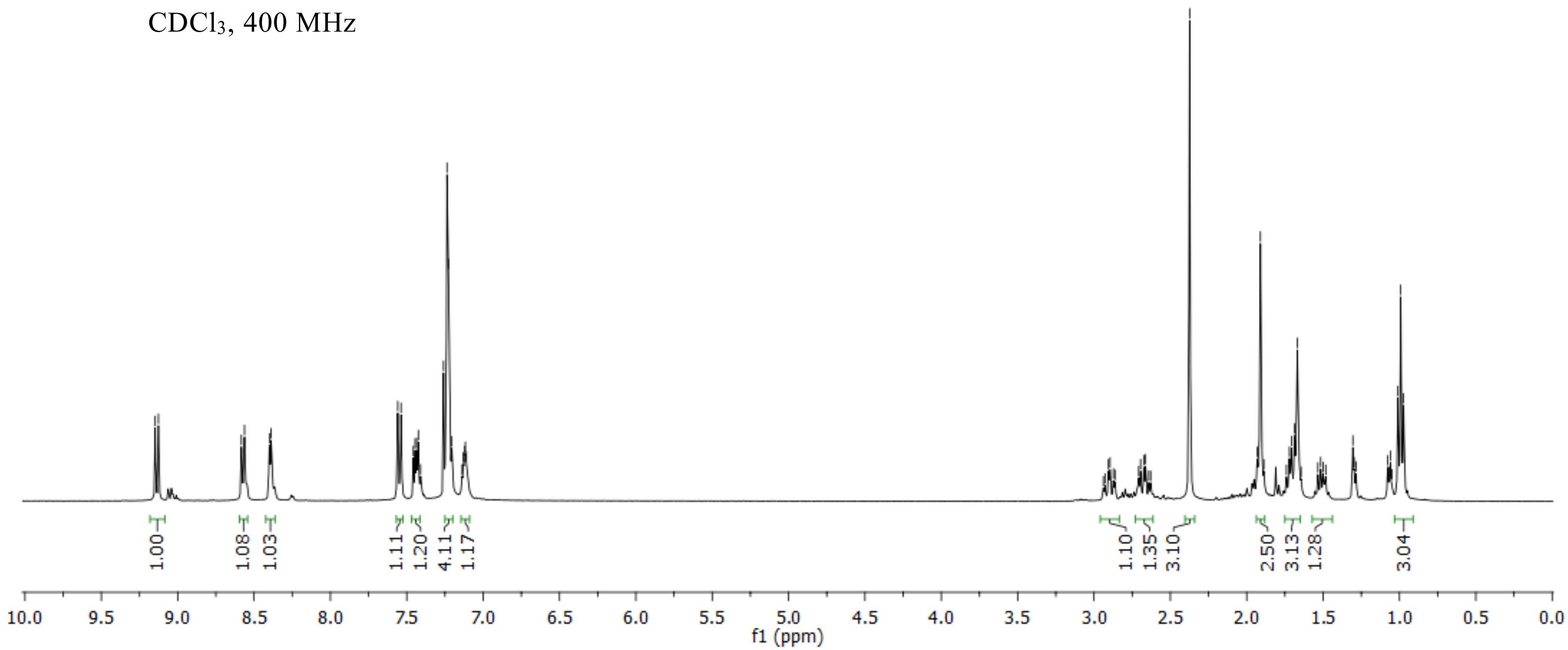

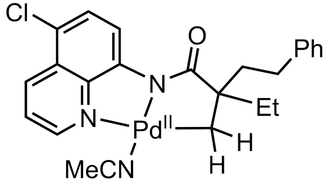

13b
$CDCl_3$, 100 MHz

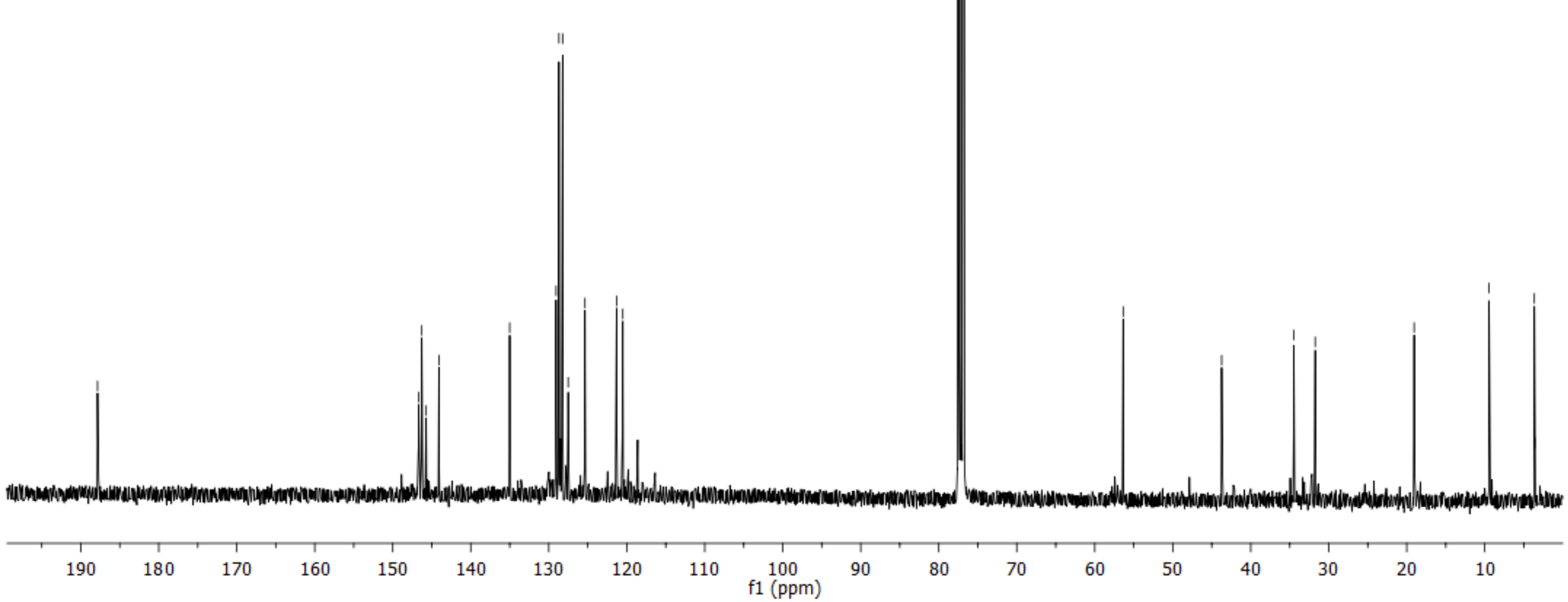

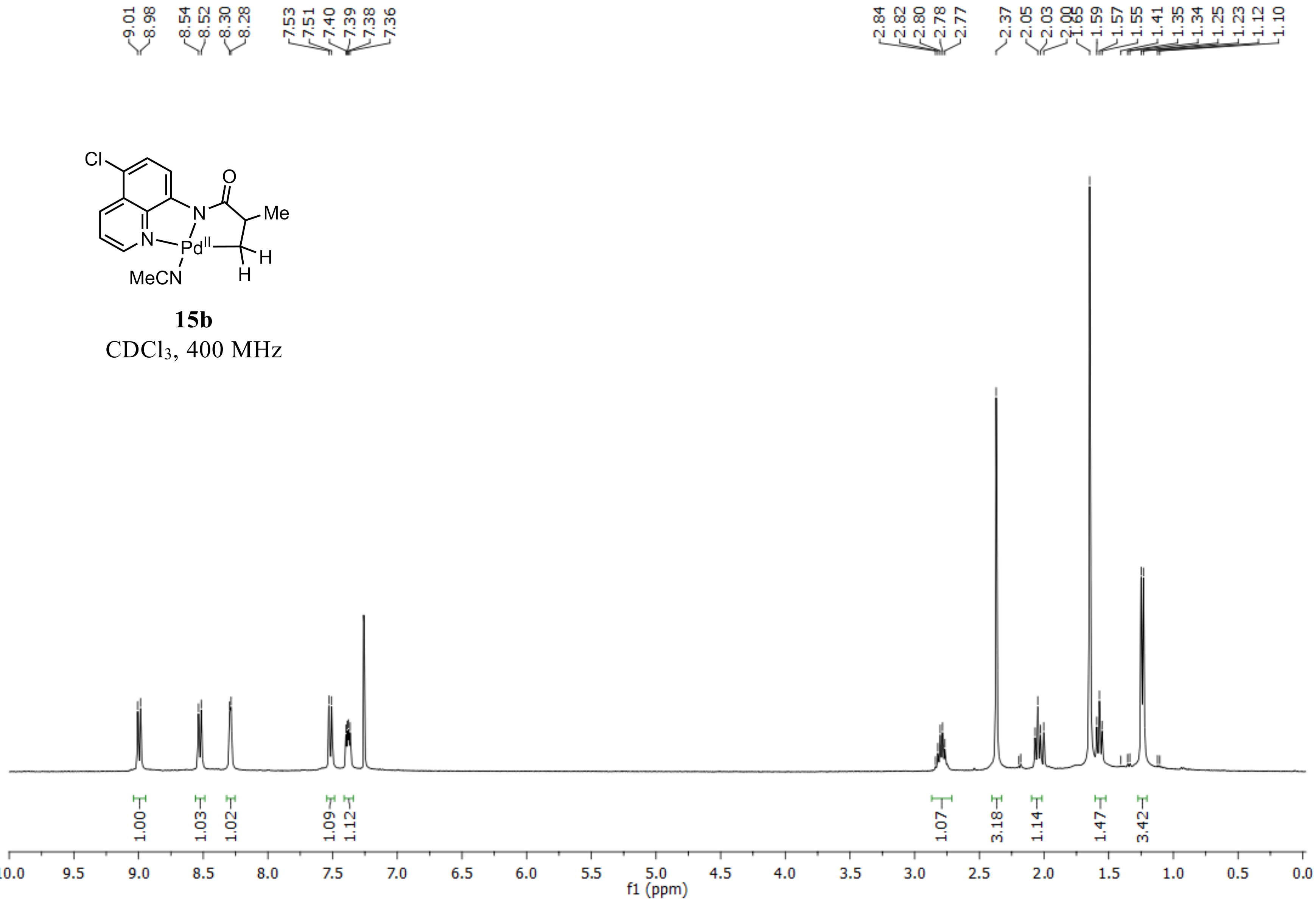

15b
$CDCl_3$, 400 MHz

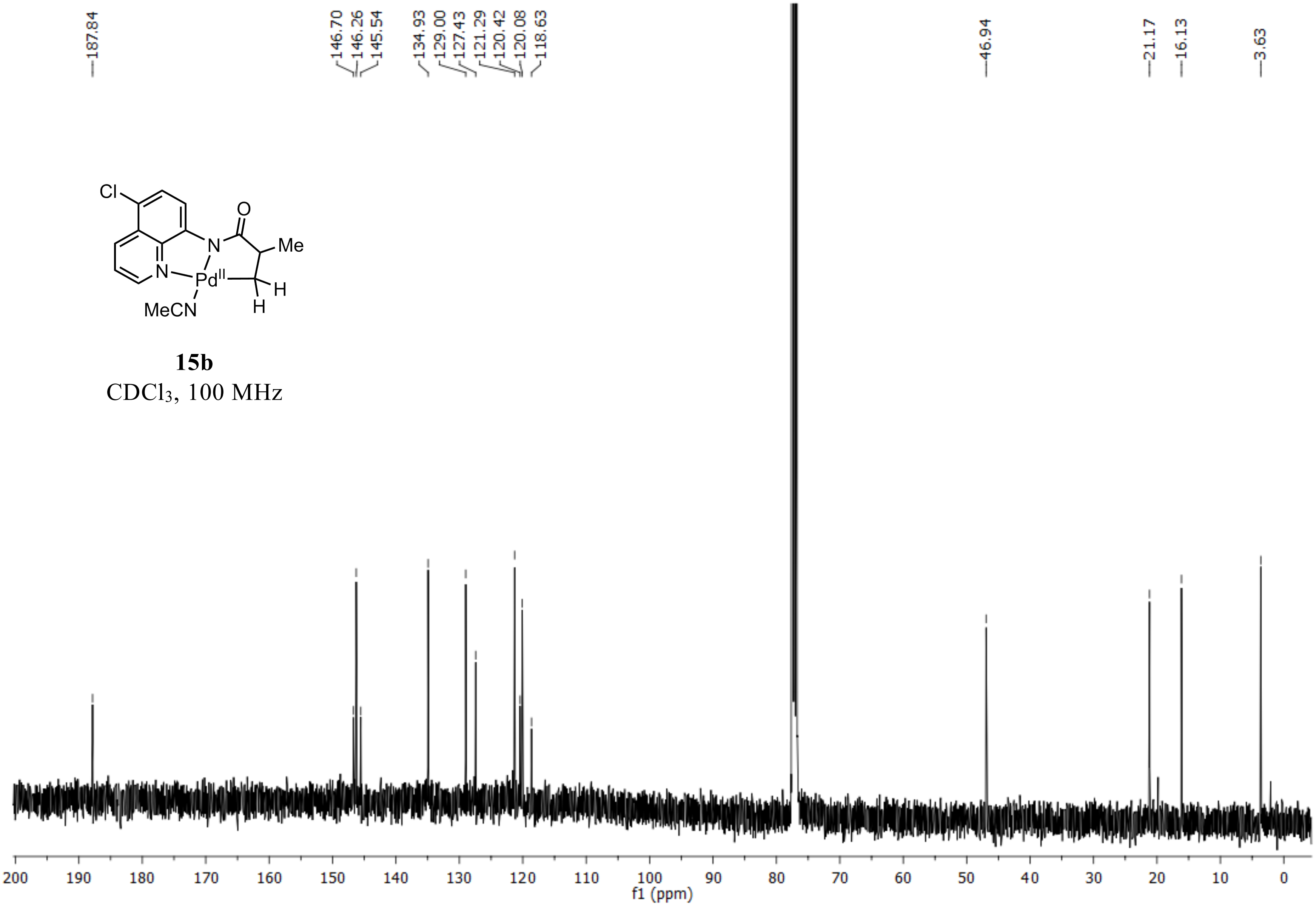

15b

$CDCl_3$, 100 MHz

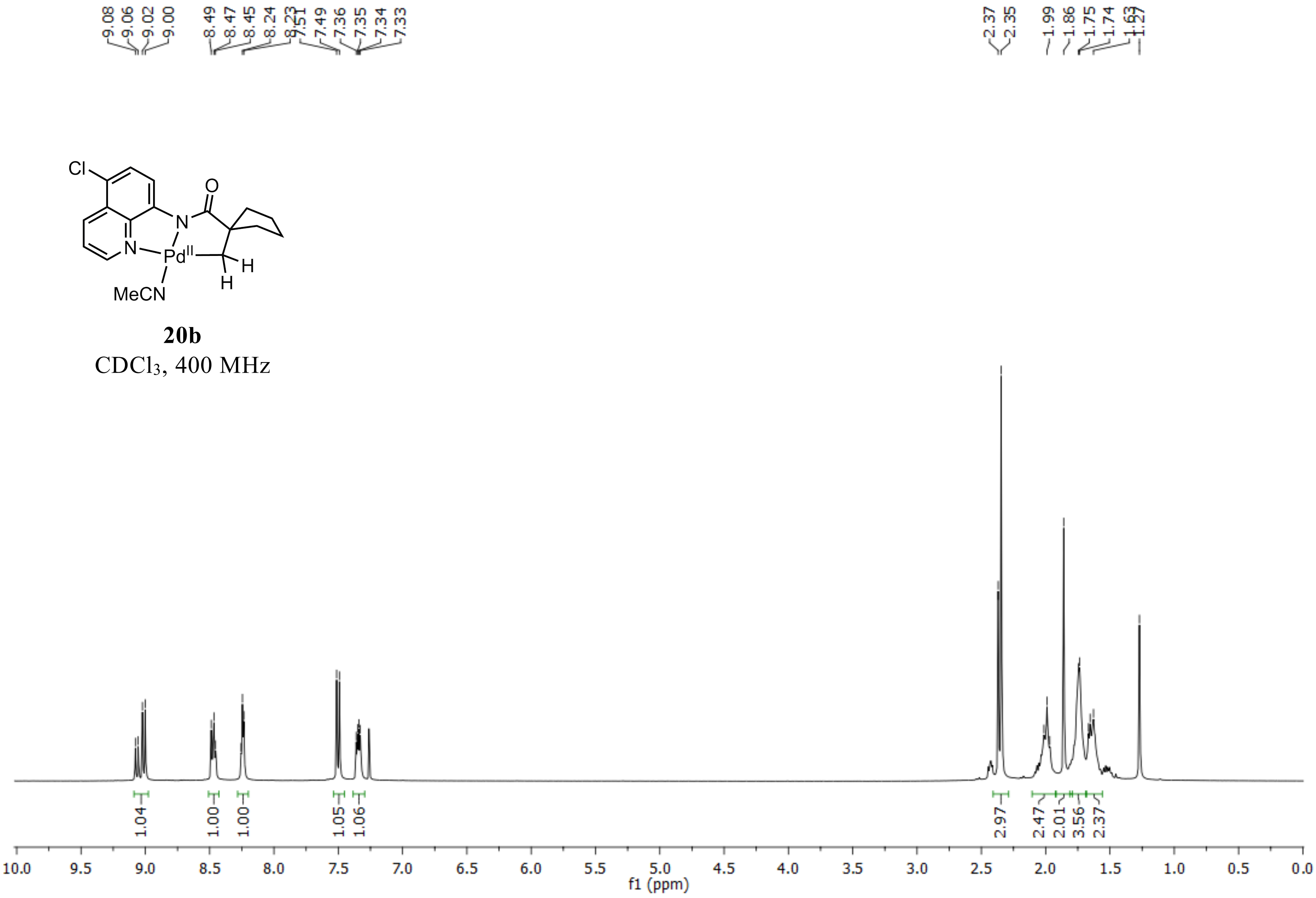

20b
$CDCl_3$, 400 MHz

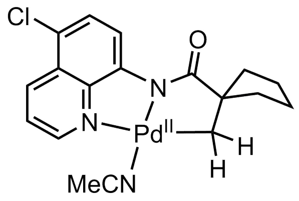

20b

$CDCl_3$, 100 MHz

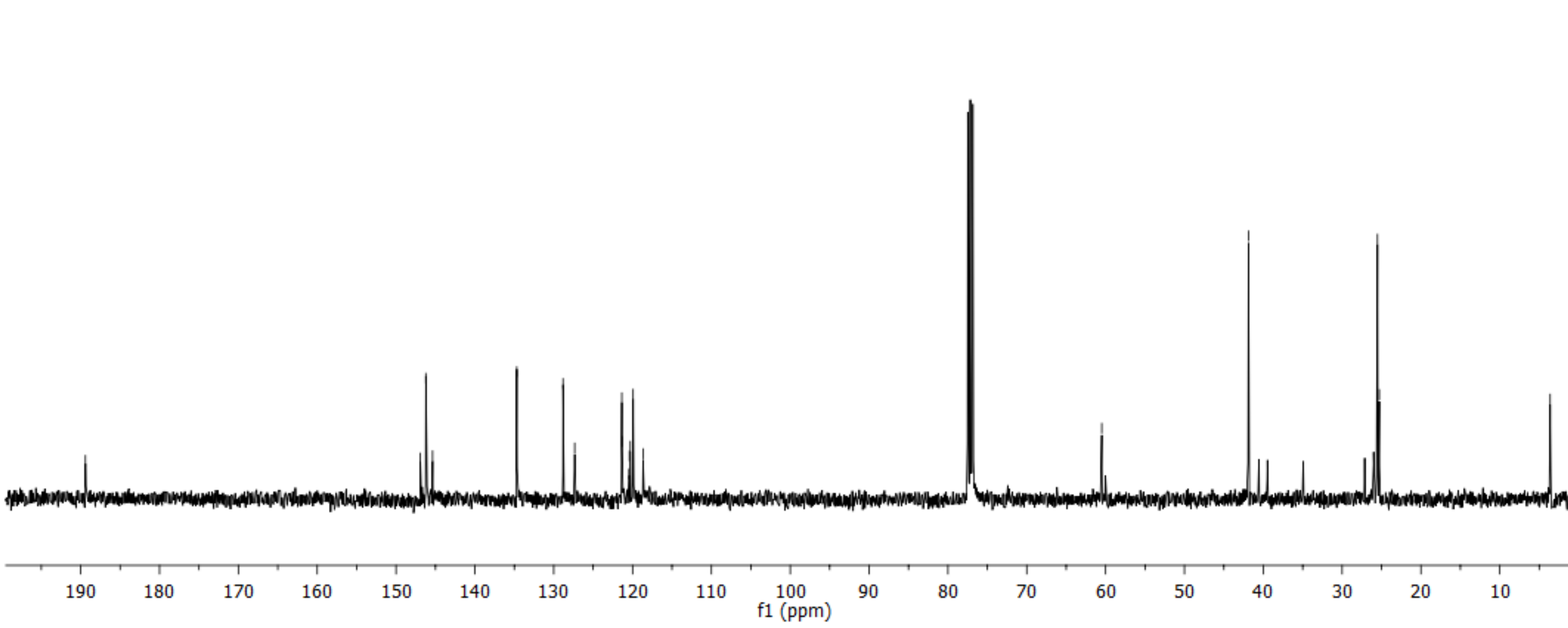

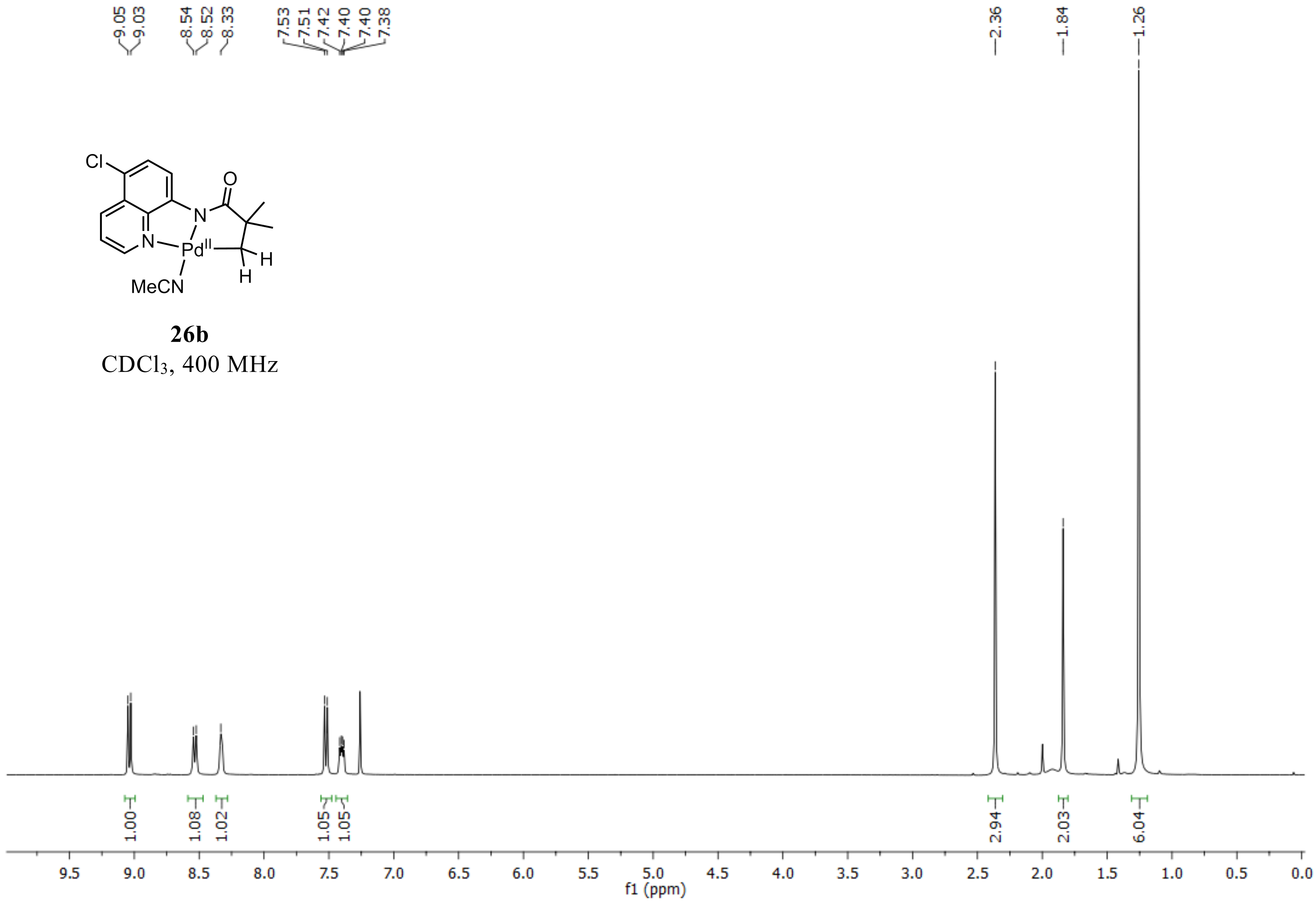

26b
$CDCl_3$, 400 MHz

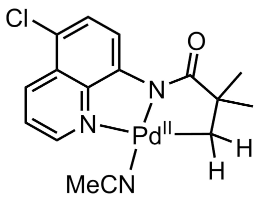

26b
$CDCl_3$, 100 MHz

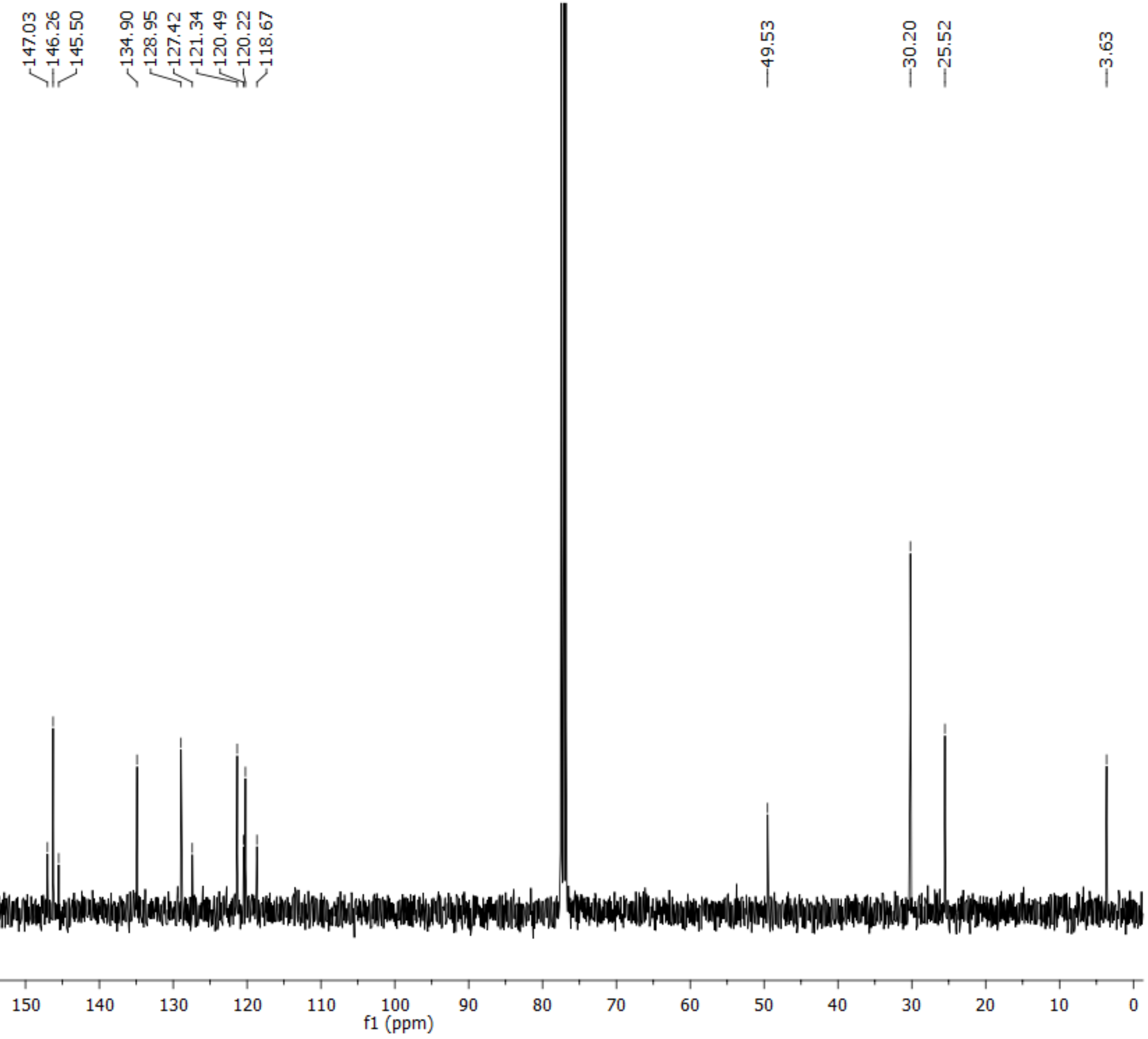

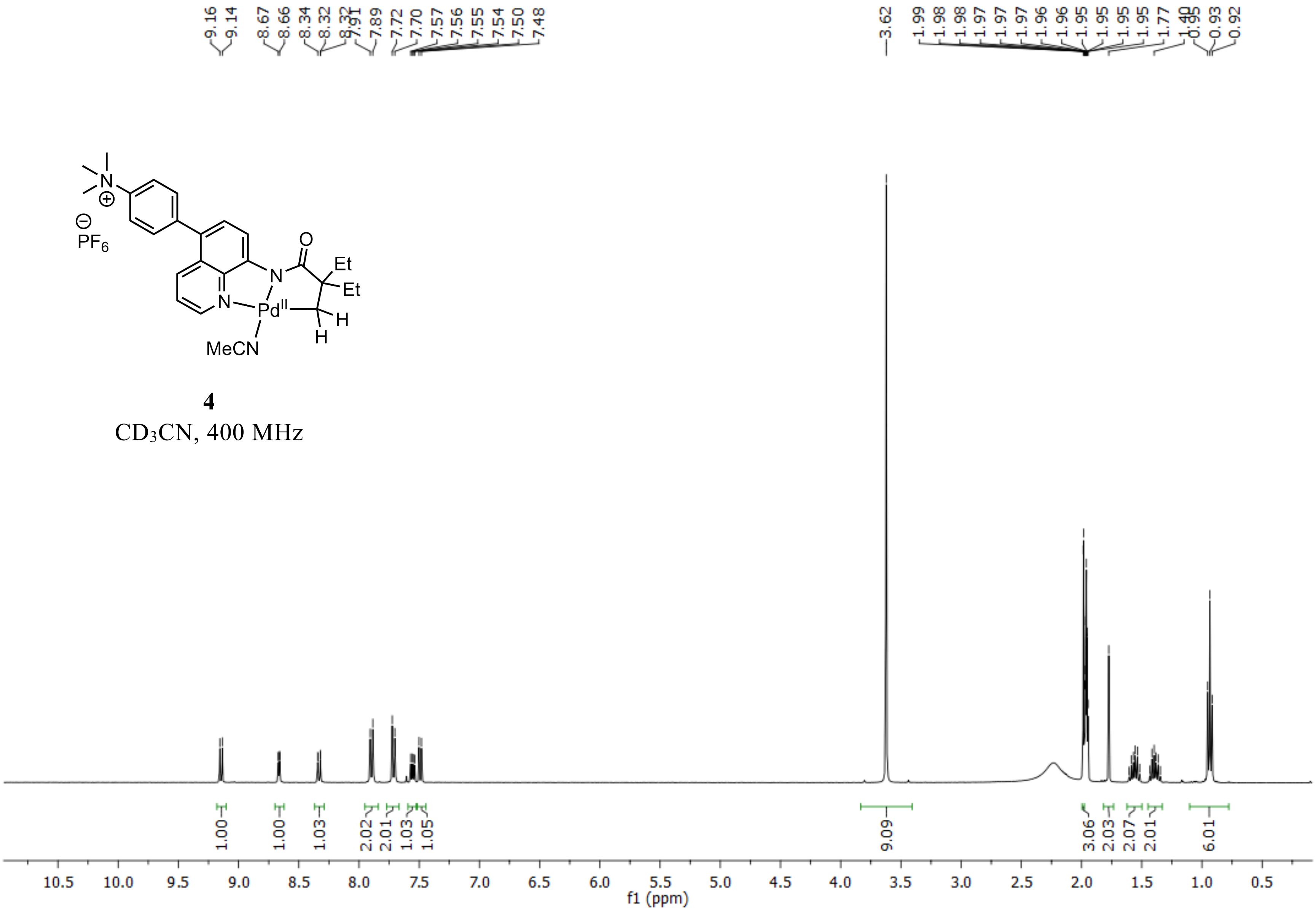

4

CD_3CN, 400 MHz

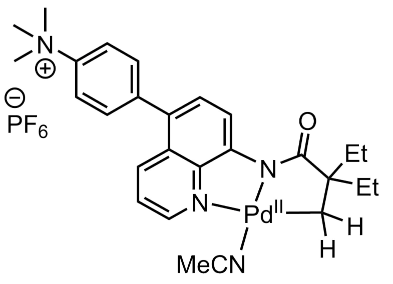

4

CD_3CN, 100 MHz

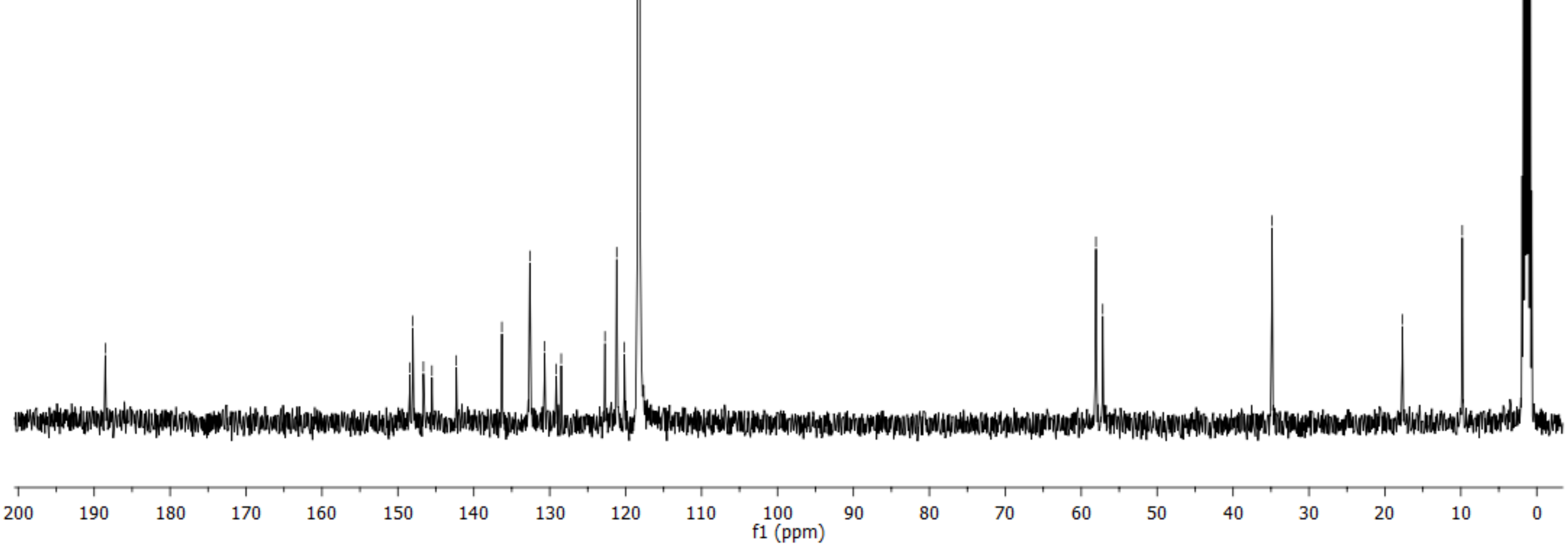

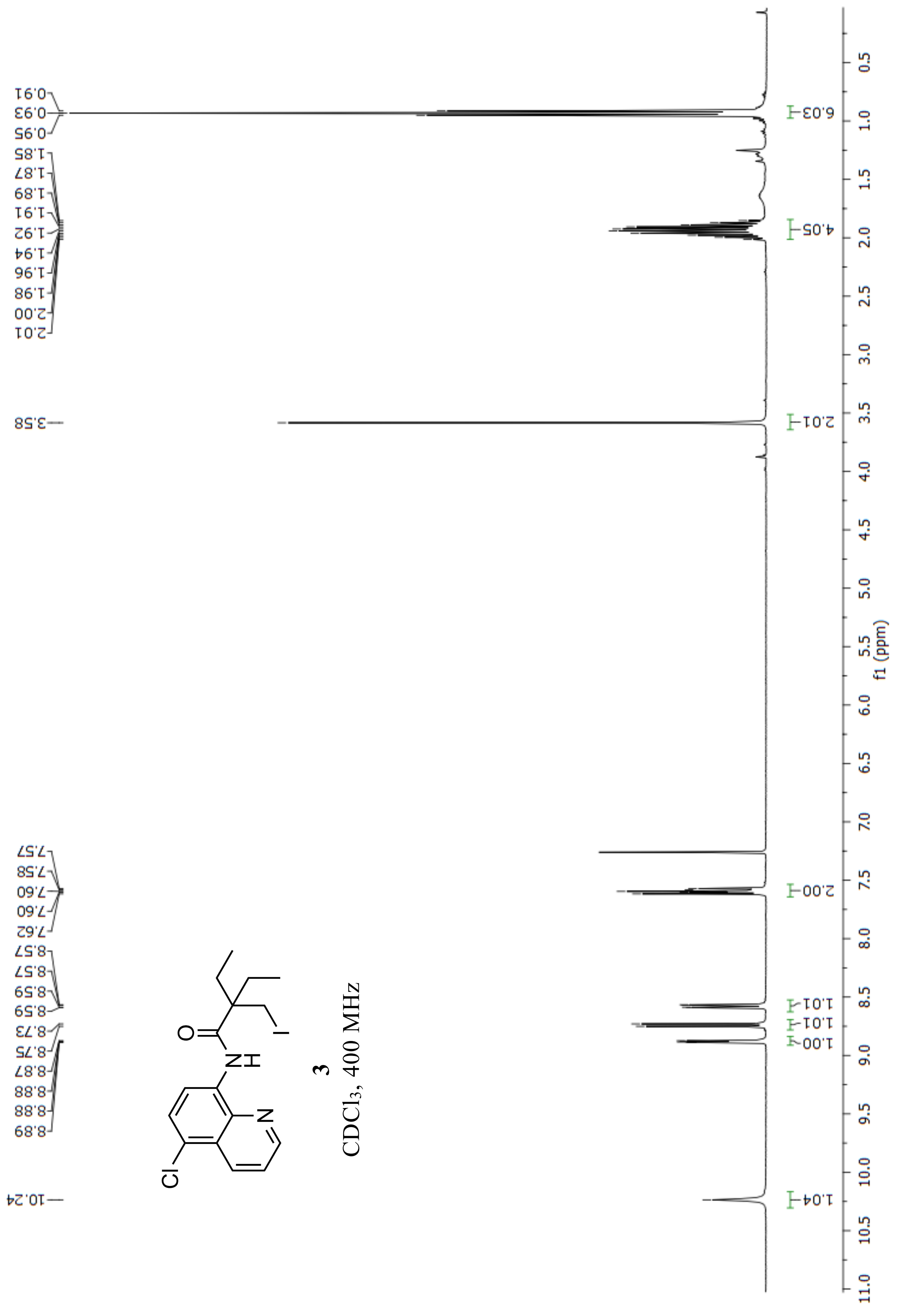

3

$CDCl_3$, 400 MHz

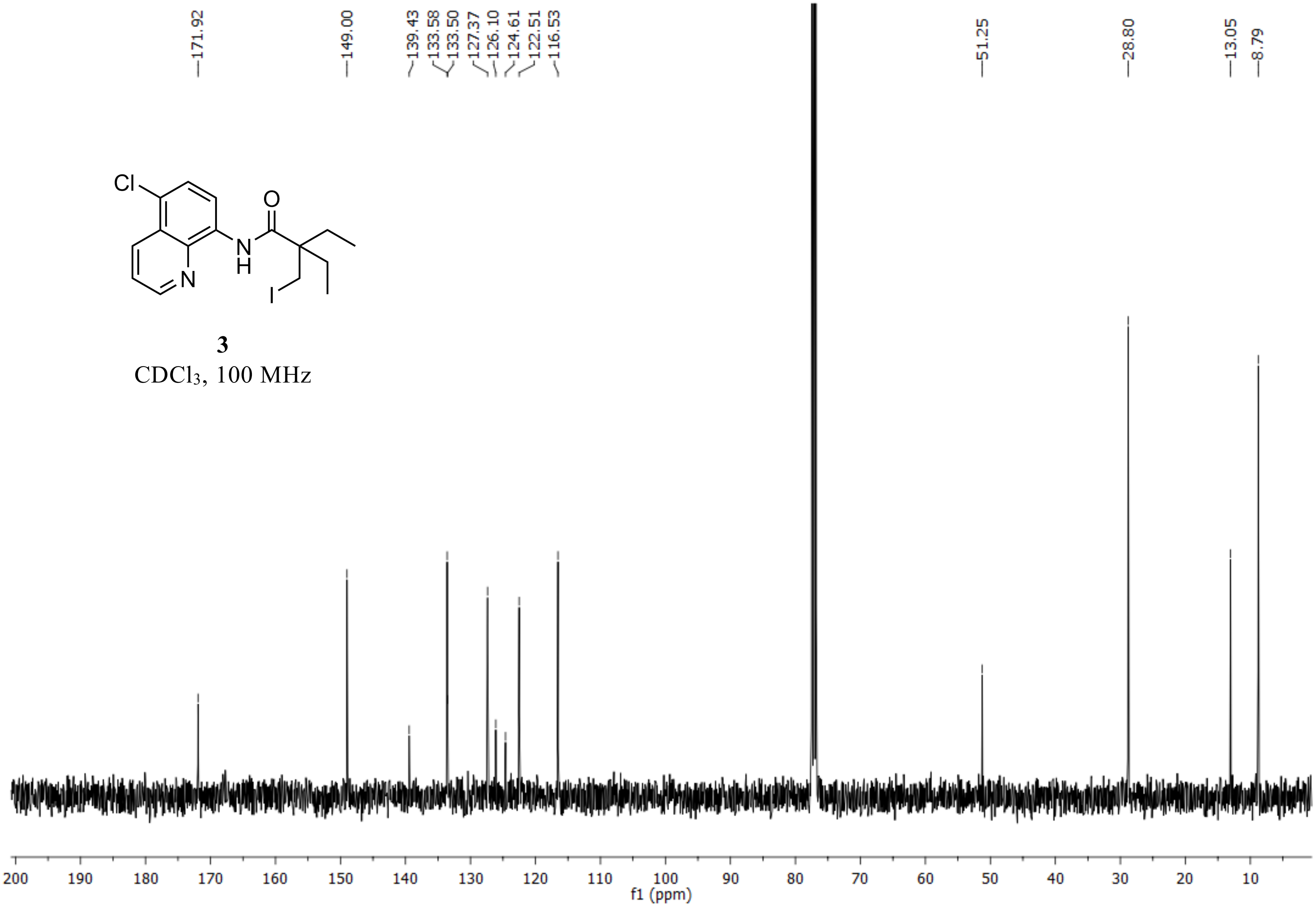

3
$CDCl_3$, 100 MHz

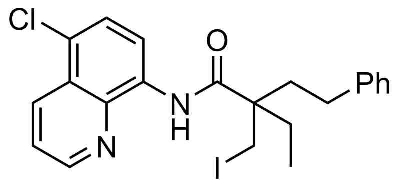

13
$CDCl_3$, 400 MHz

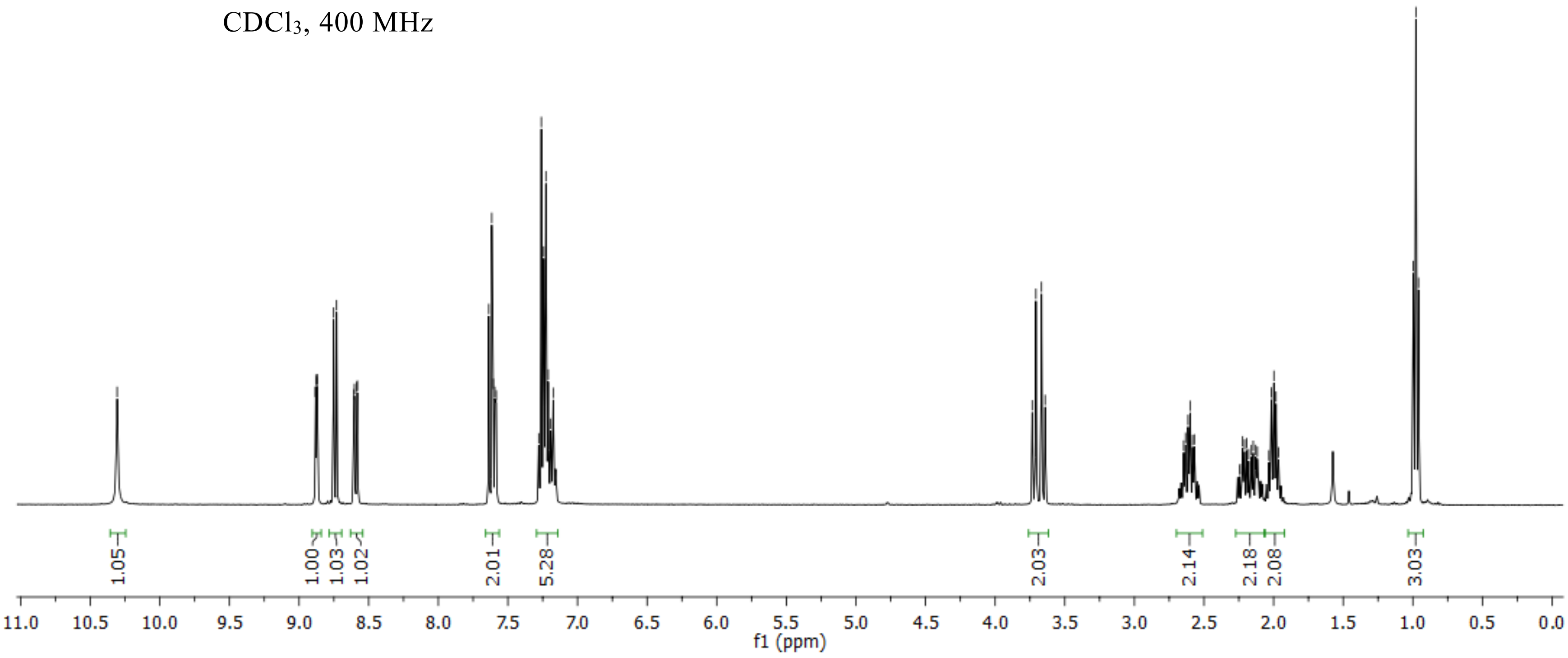

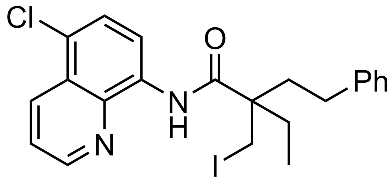

13
$CDCl_3$, 100 MHz

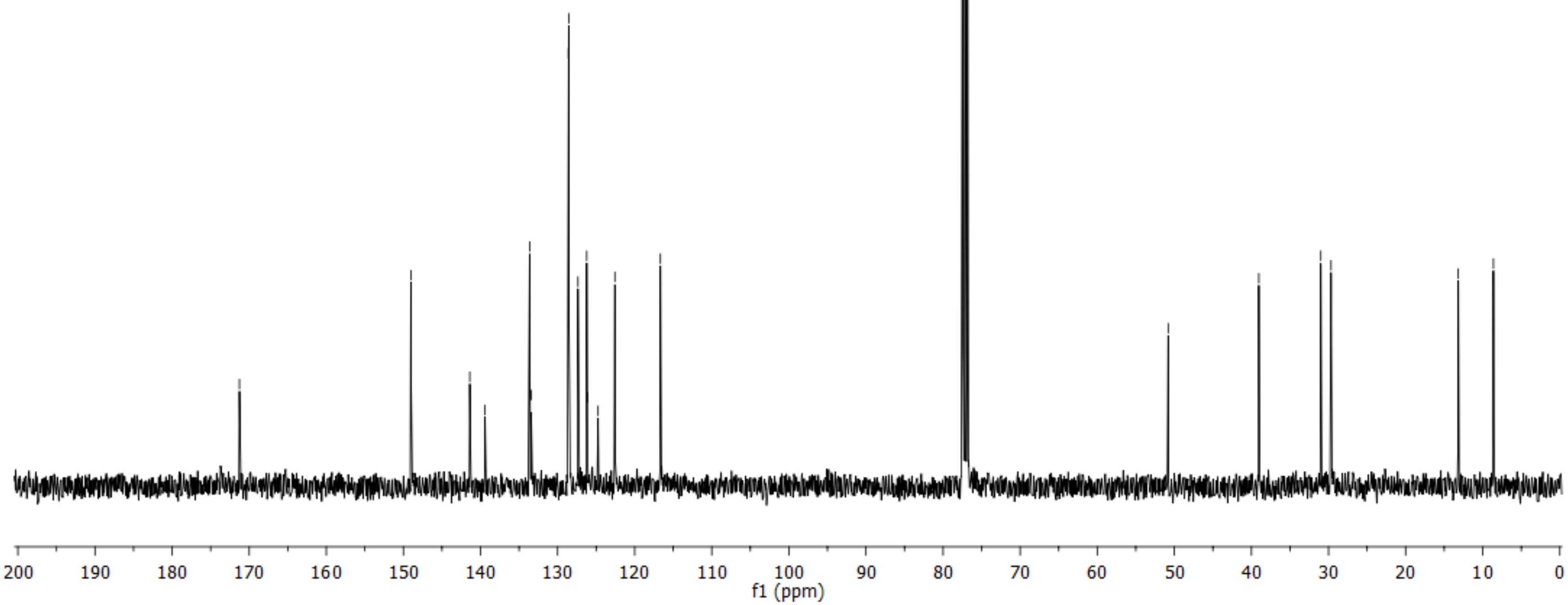

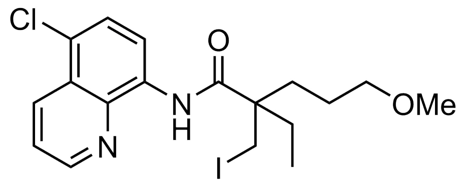

14
$CDCl_3$, 400 MHz

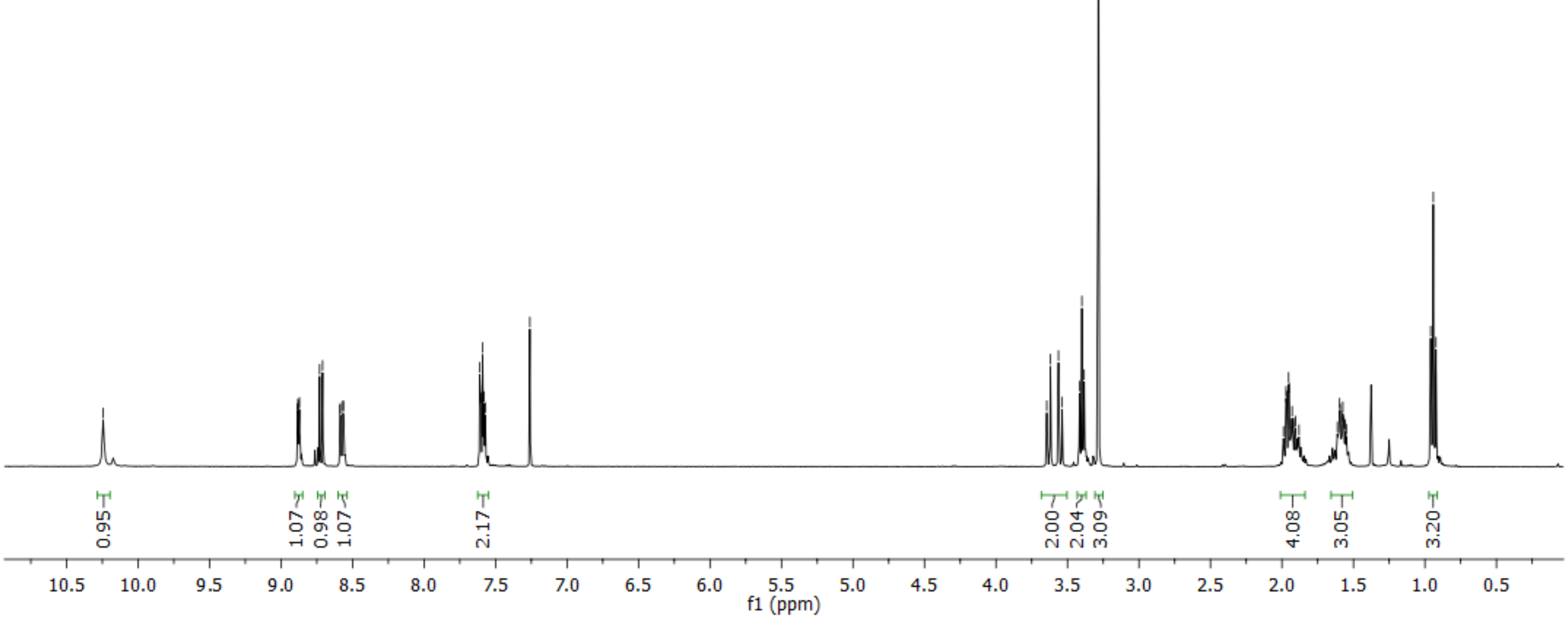

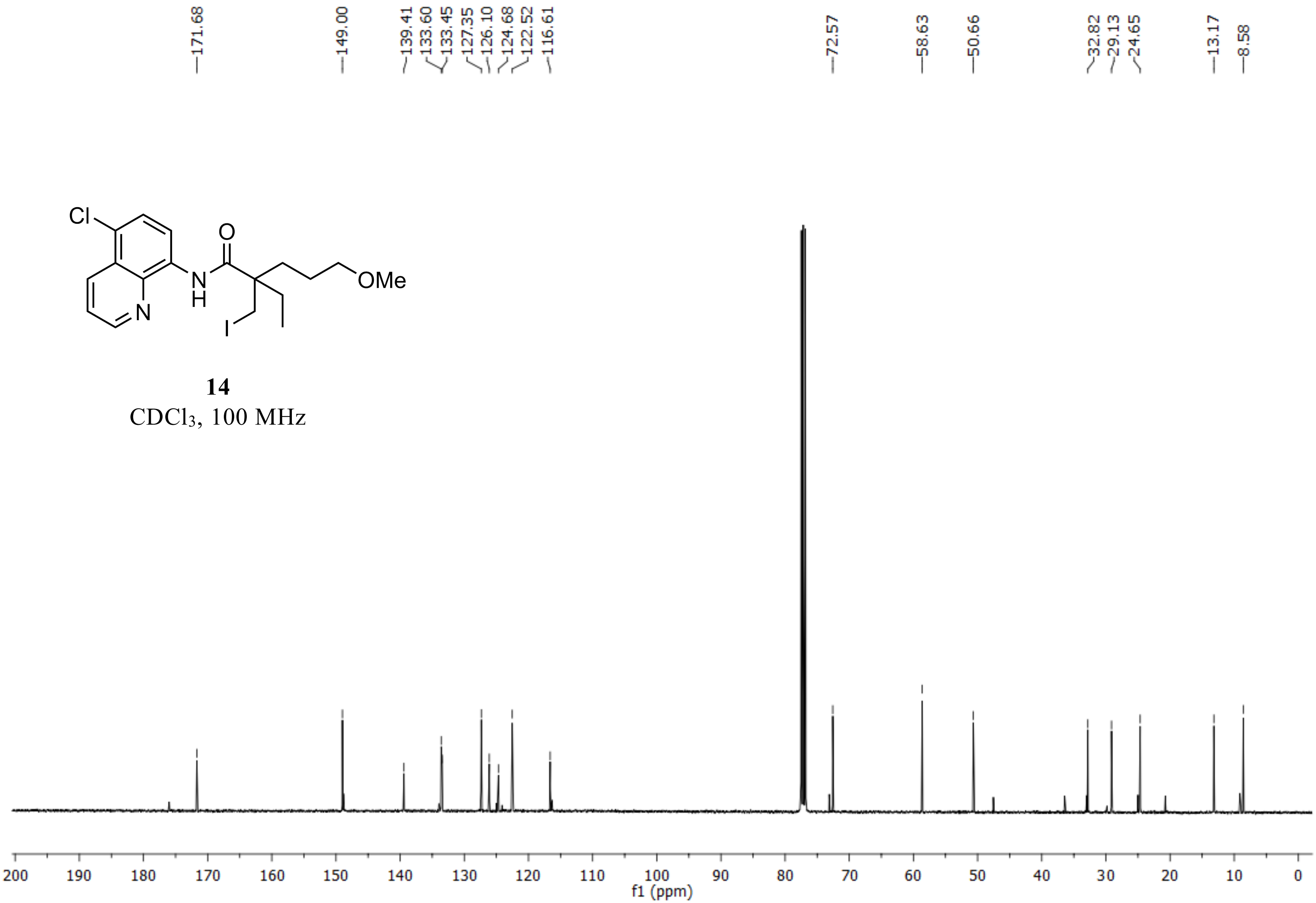

14
$CDCl_3$, 100 MHz

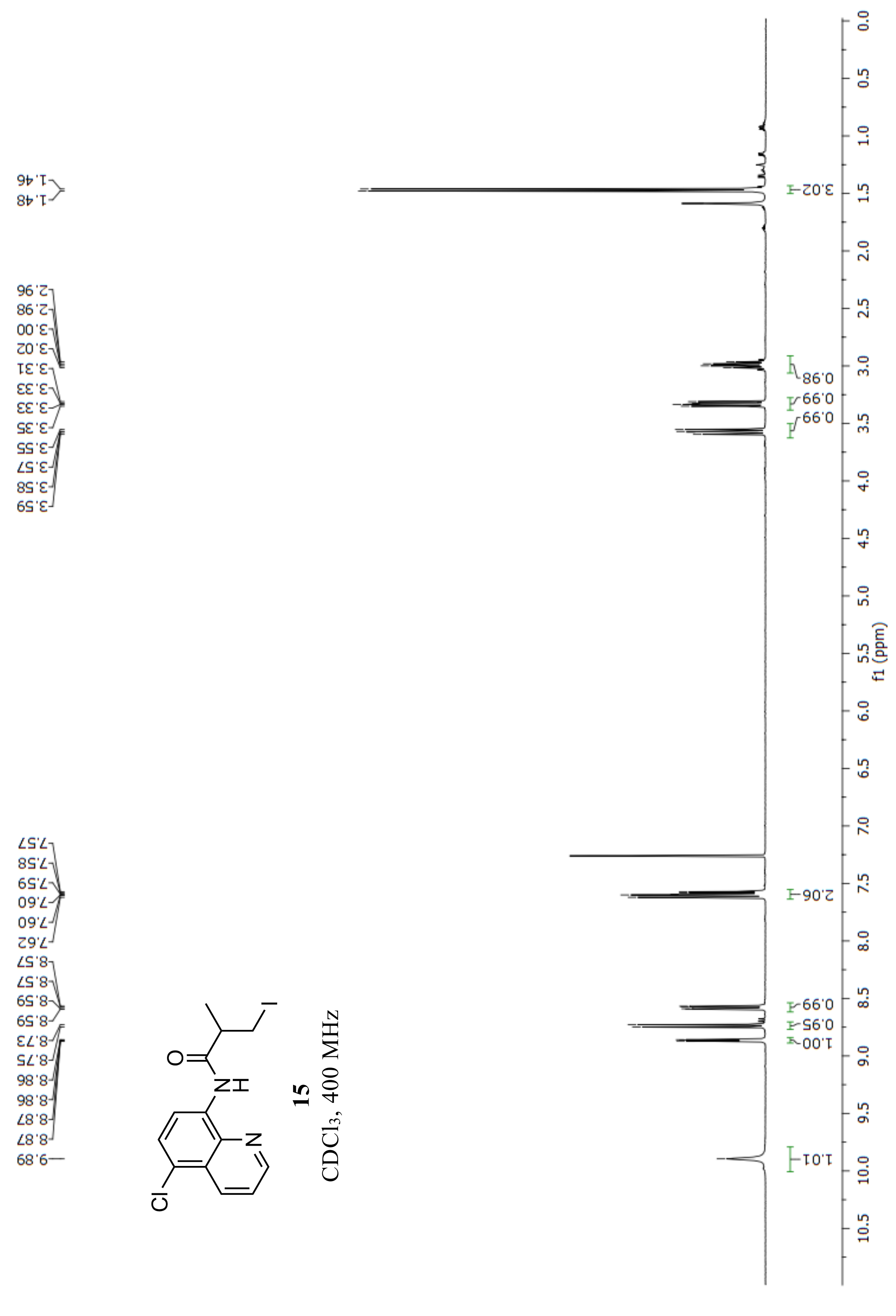

9.89
8.87
8.87
8.86
8.86
8.75
8.73
8.59
8.59
8.57
8.57
7.62
7.60
7.60
7.59
7.58
7.57
3.59
3.58
3.57
3.55
3.35
3.33
3.33
3.31
3.02
3.00
2.98
2.96
1.48
1.46
Cl
N
H
N
O
I
15
CDCl3, 400 MHz
1.01
1.00
0.95
0.99
2.06
0.99
0.99
0.98
3.02
f1 (ppm)

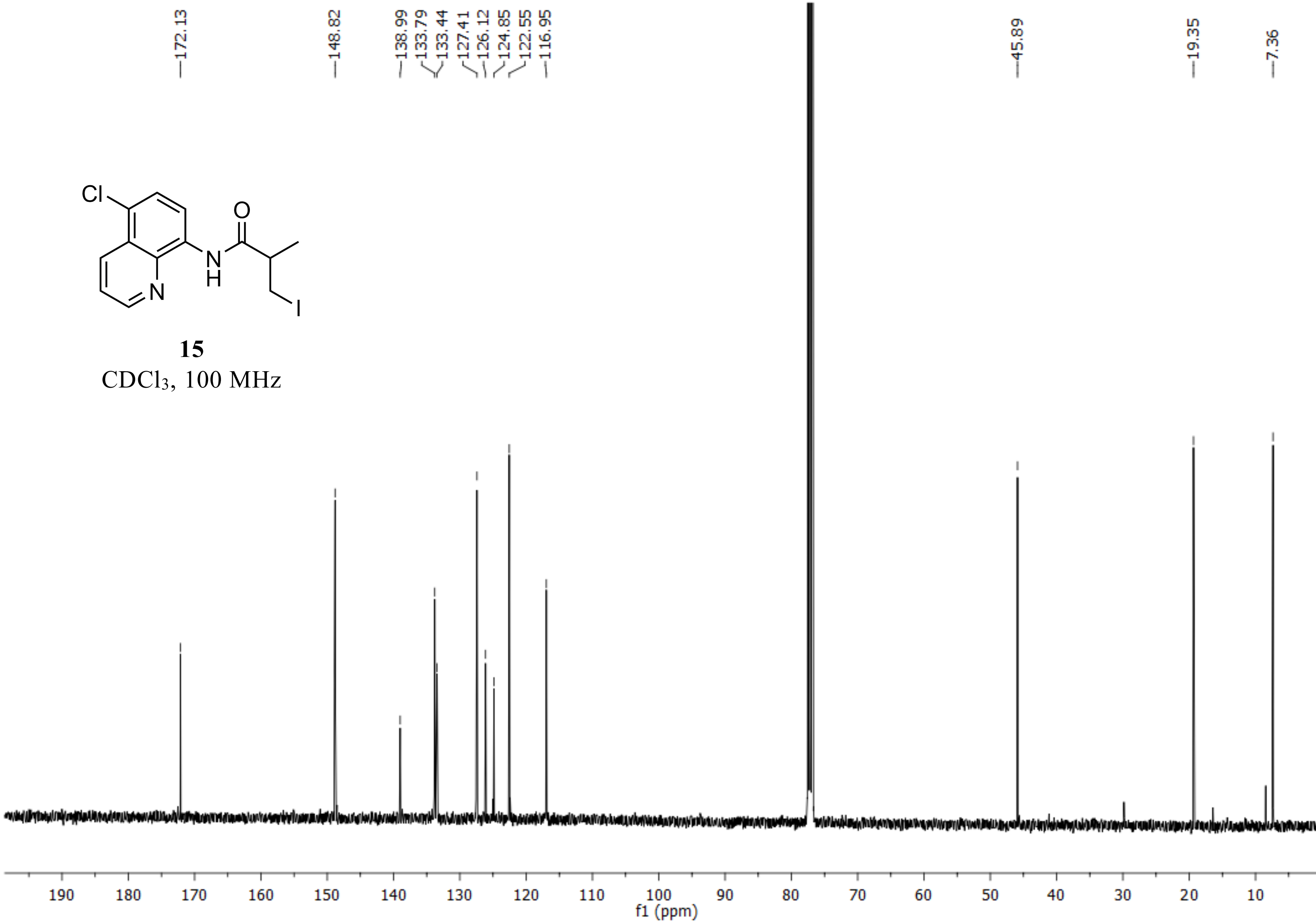

15

$CDCl_3$, 100 MHz

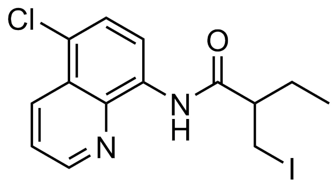

16

$CDCl_3$, 400 MHz

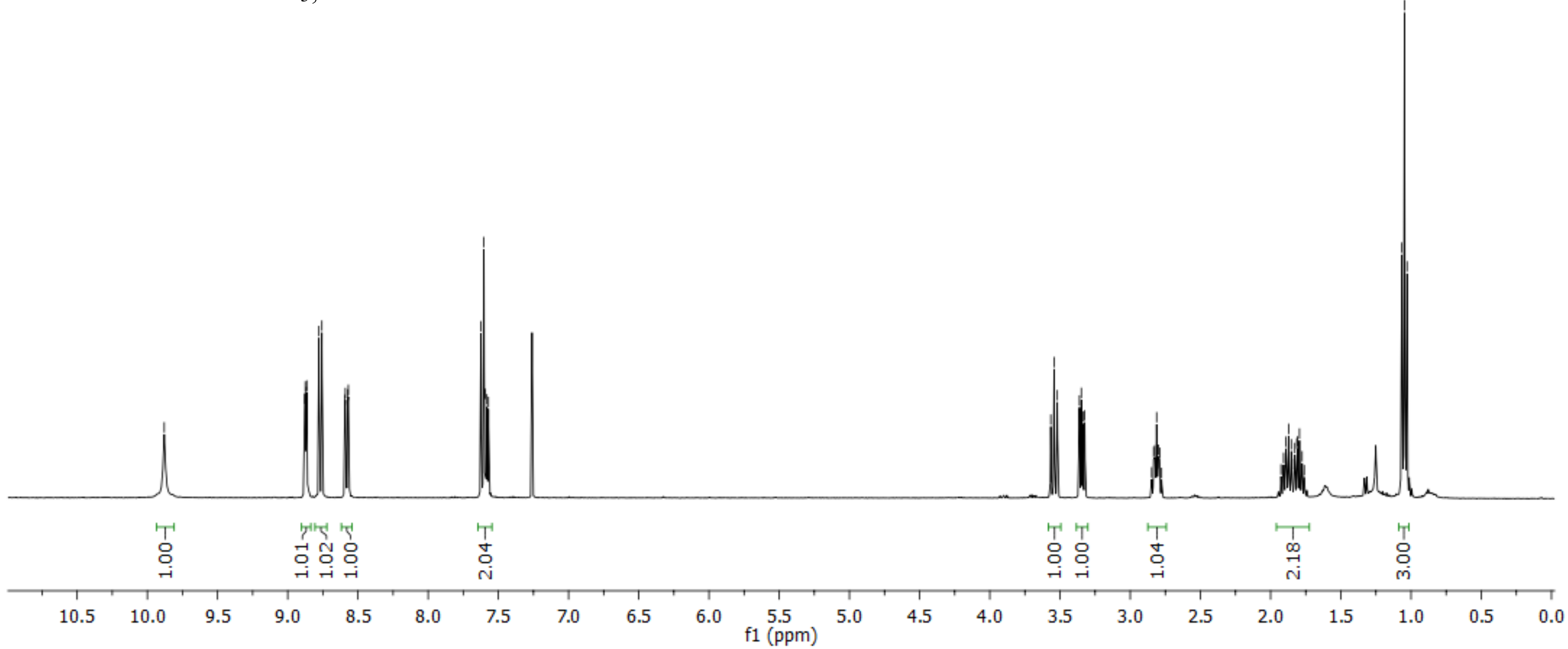

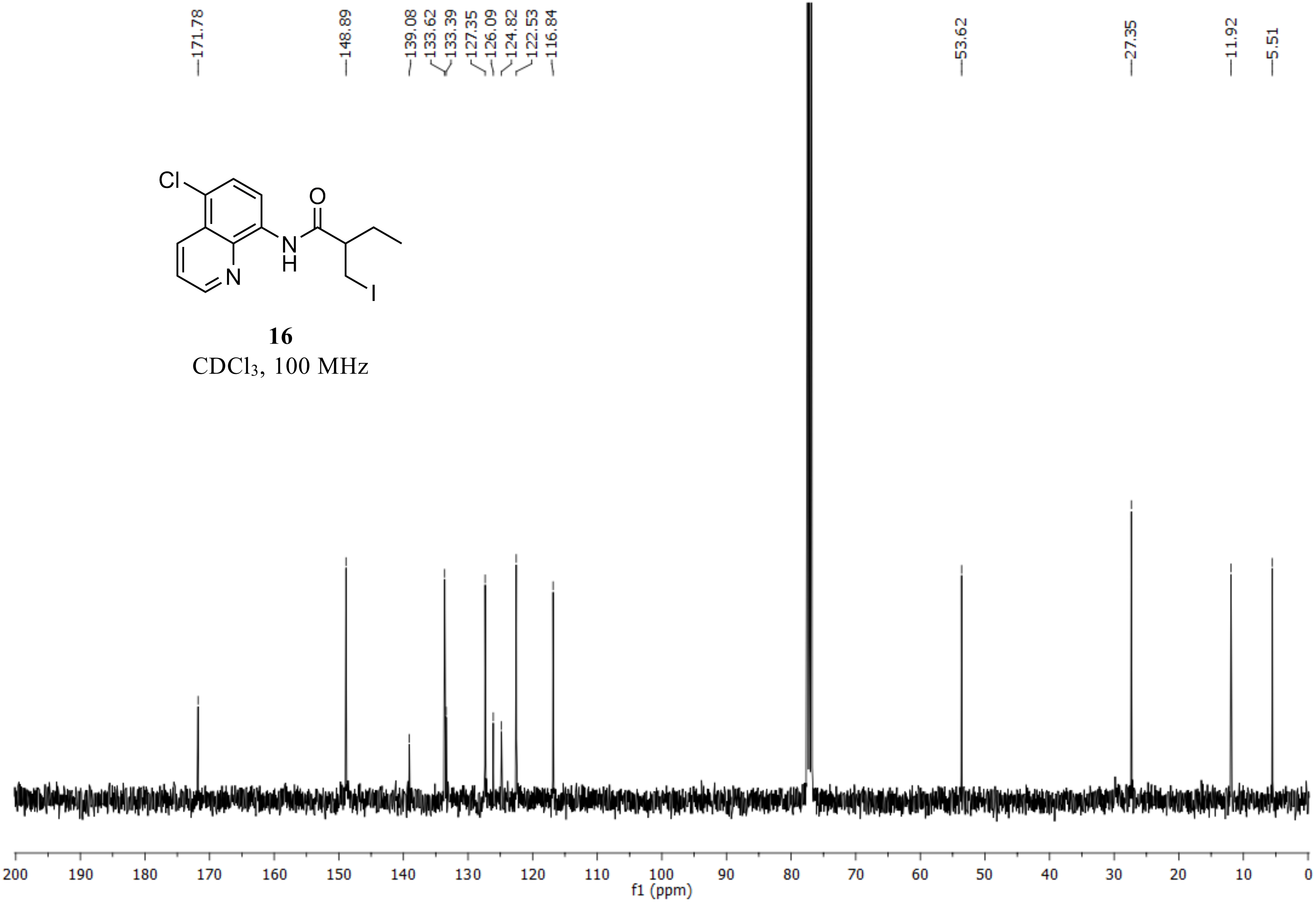

16
$CDCl_3$, 100 MHz

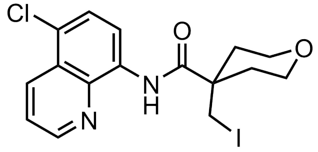

17

$CDCl_3$, 400 MHz

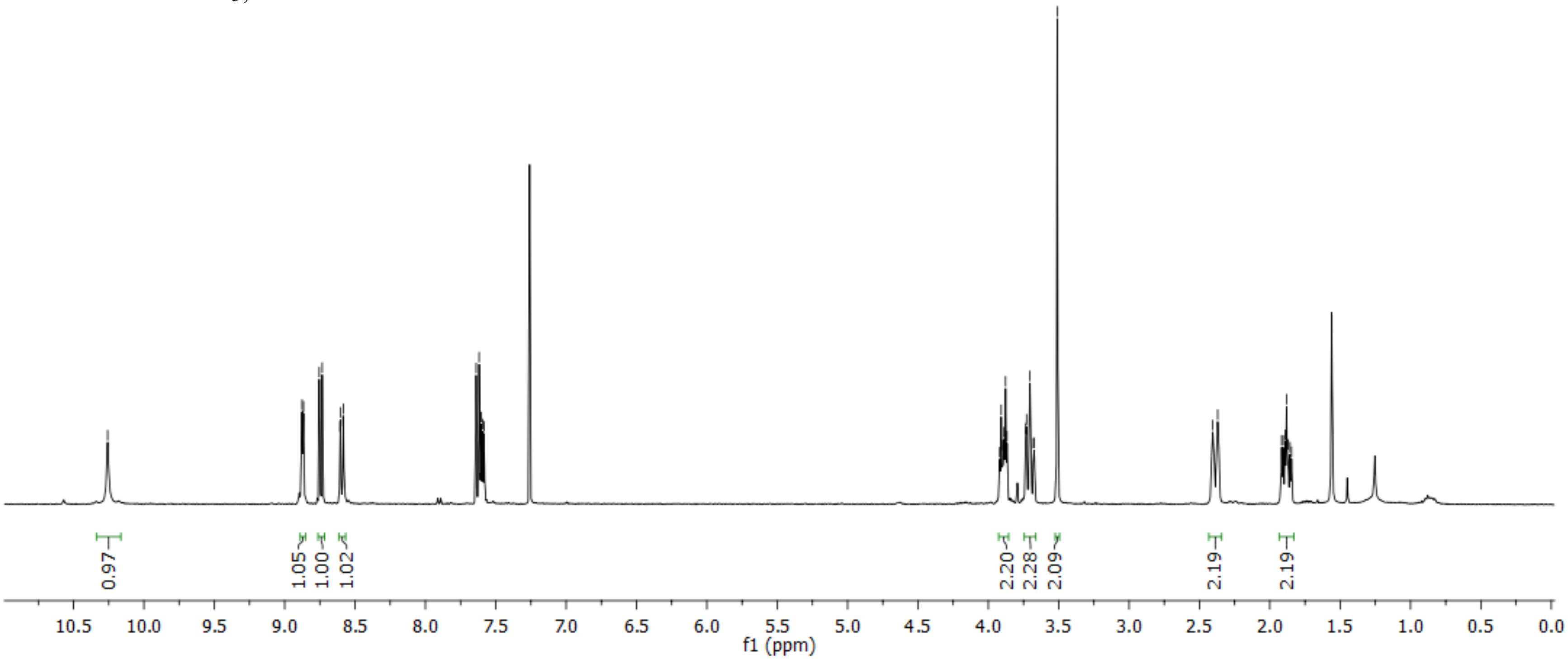

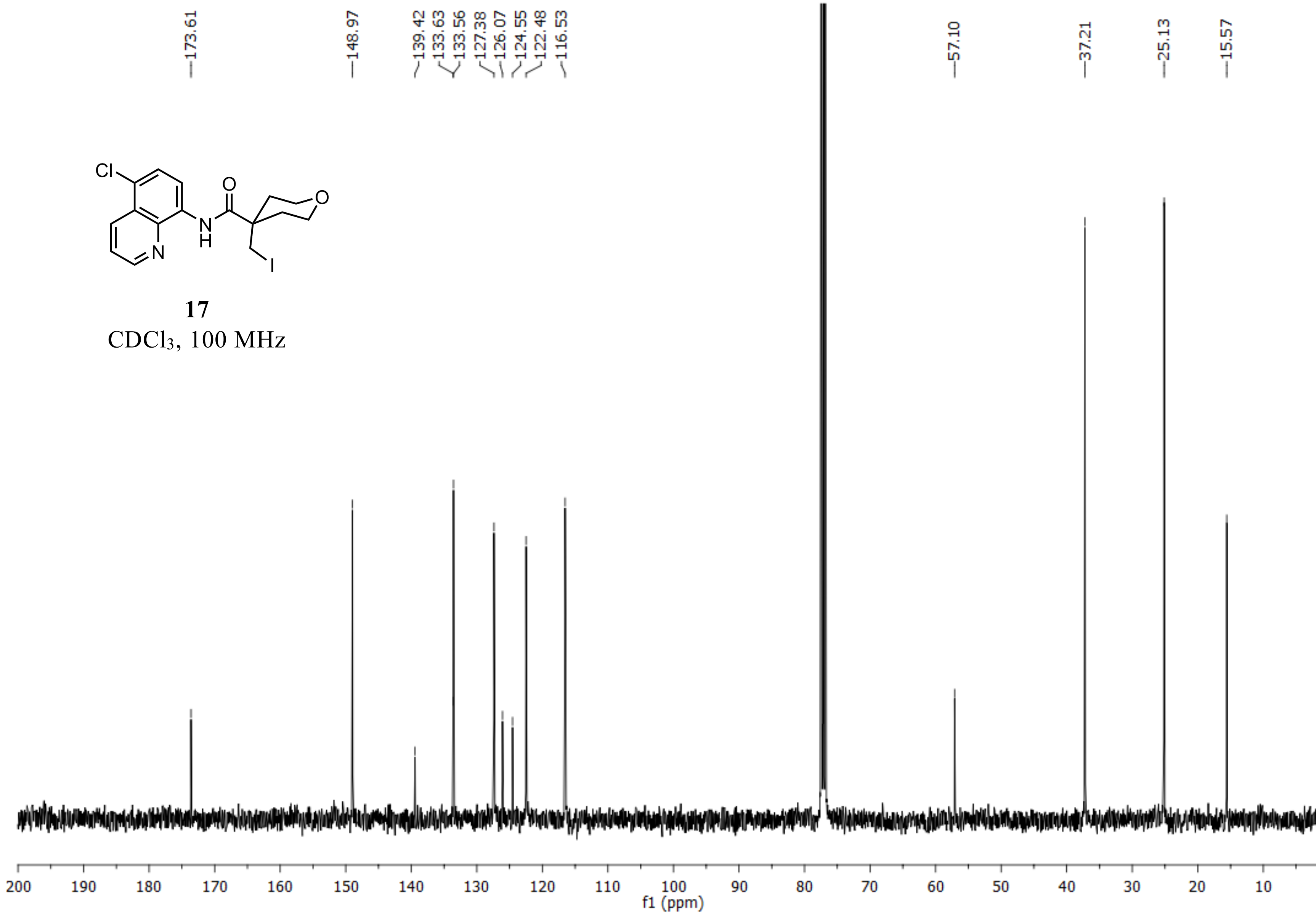

17
$CDCl_3$, 100 MHz

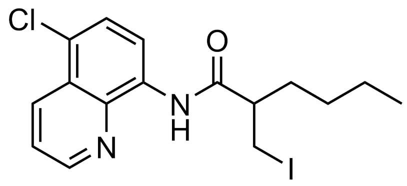

18
$CDCl_3$, 400 MHz

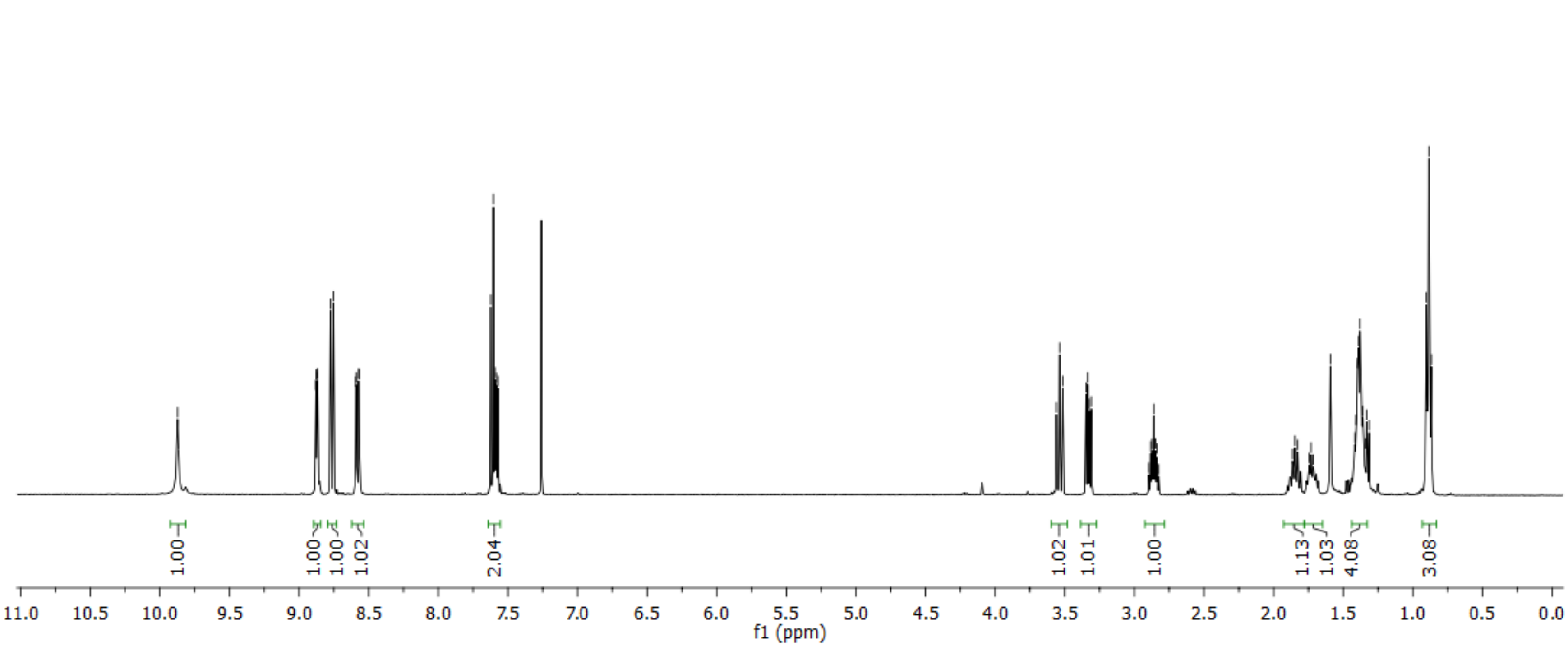

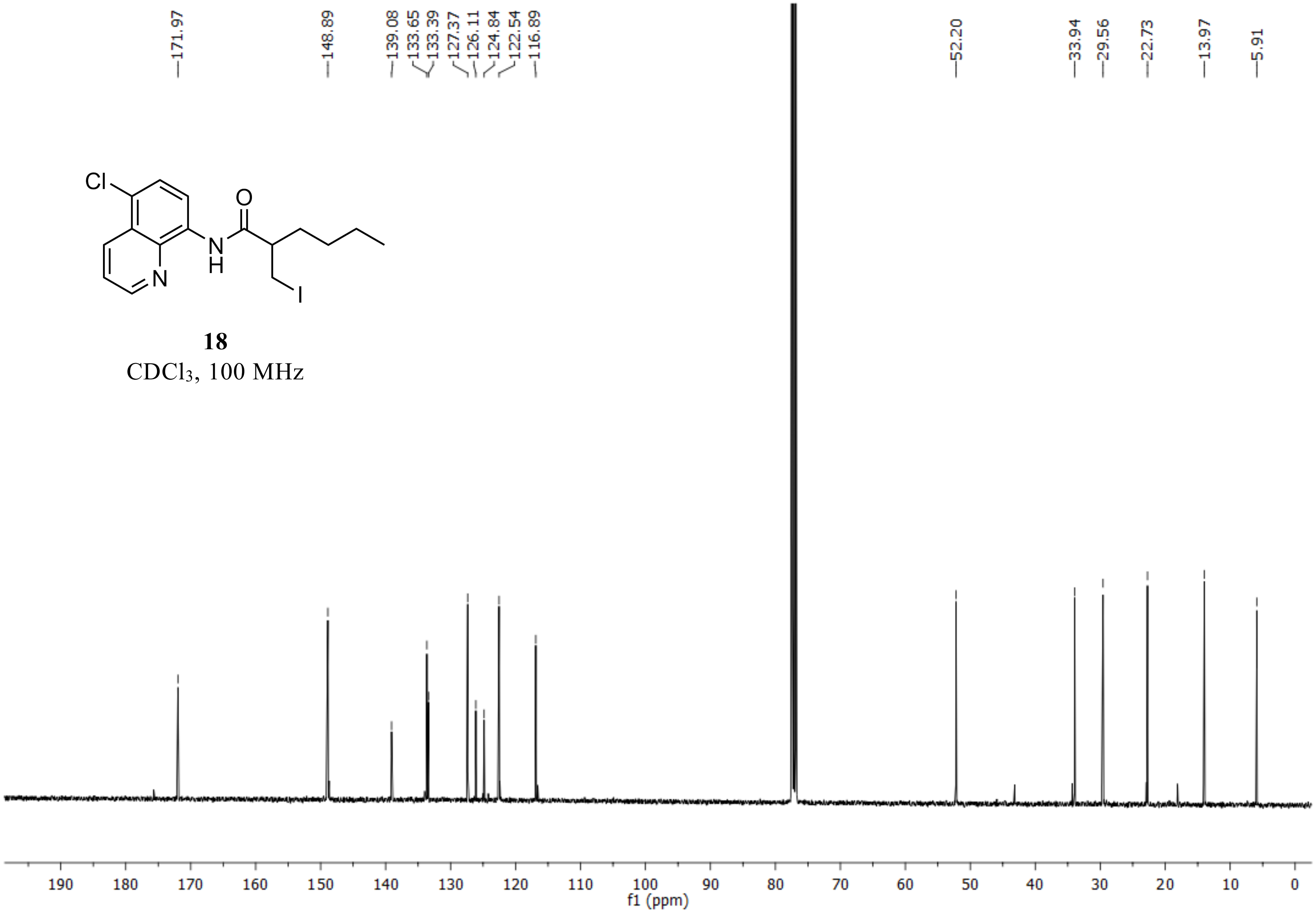

18

$CDCl_3$, 100 MHz

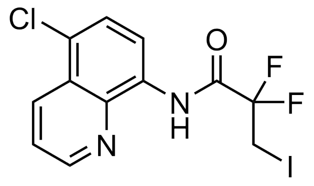

19
$CDCl_3$, 400 MHz

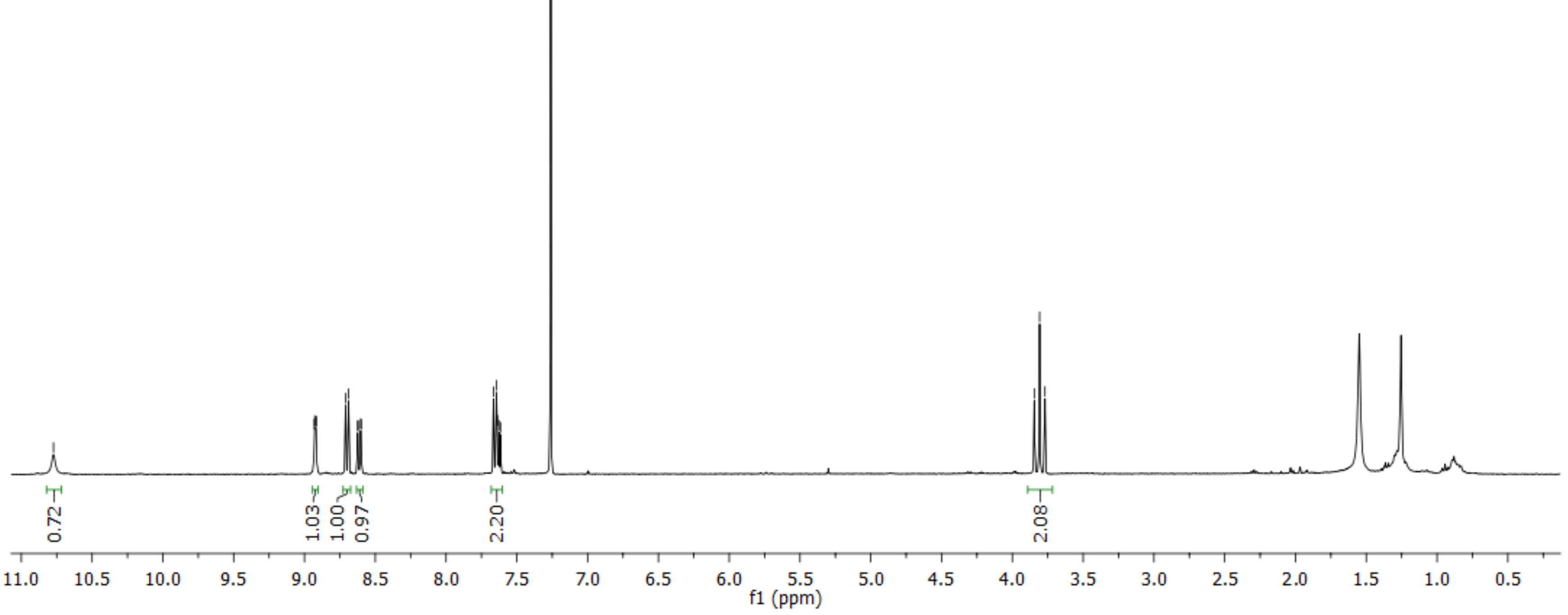

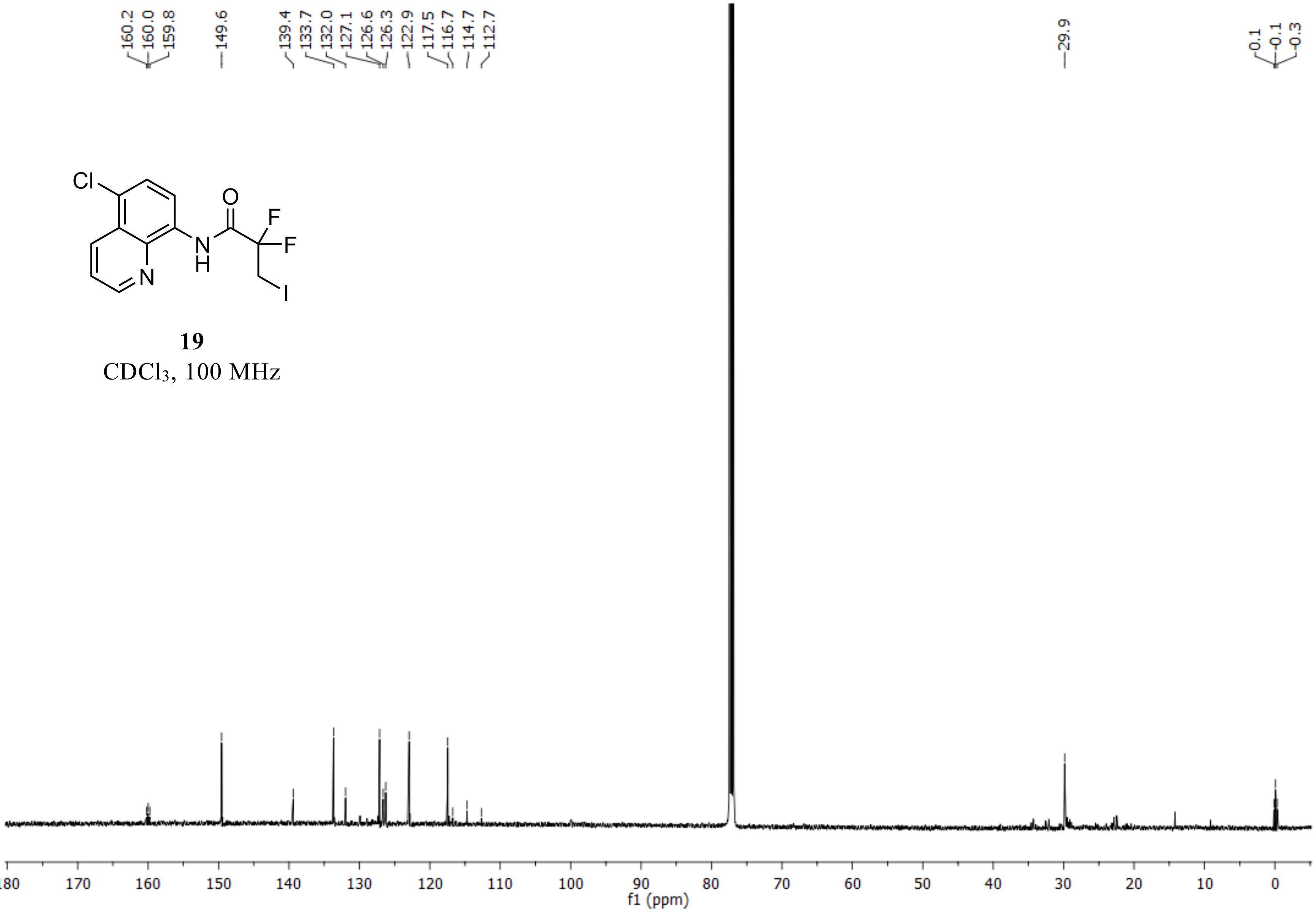

19

CDCl$_3$, 100 MHz

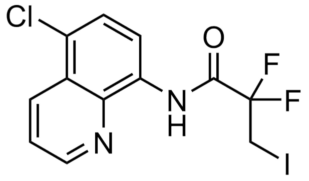

19

$CDCl_3$, 376 MHz

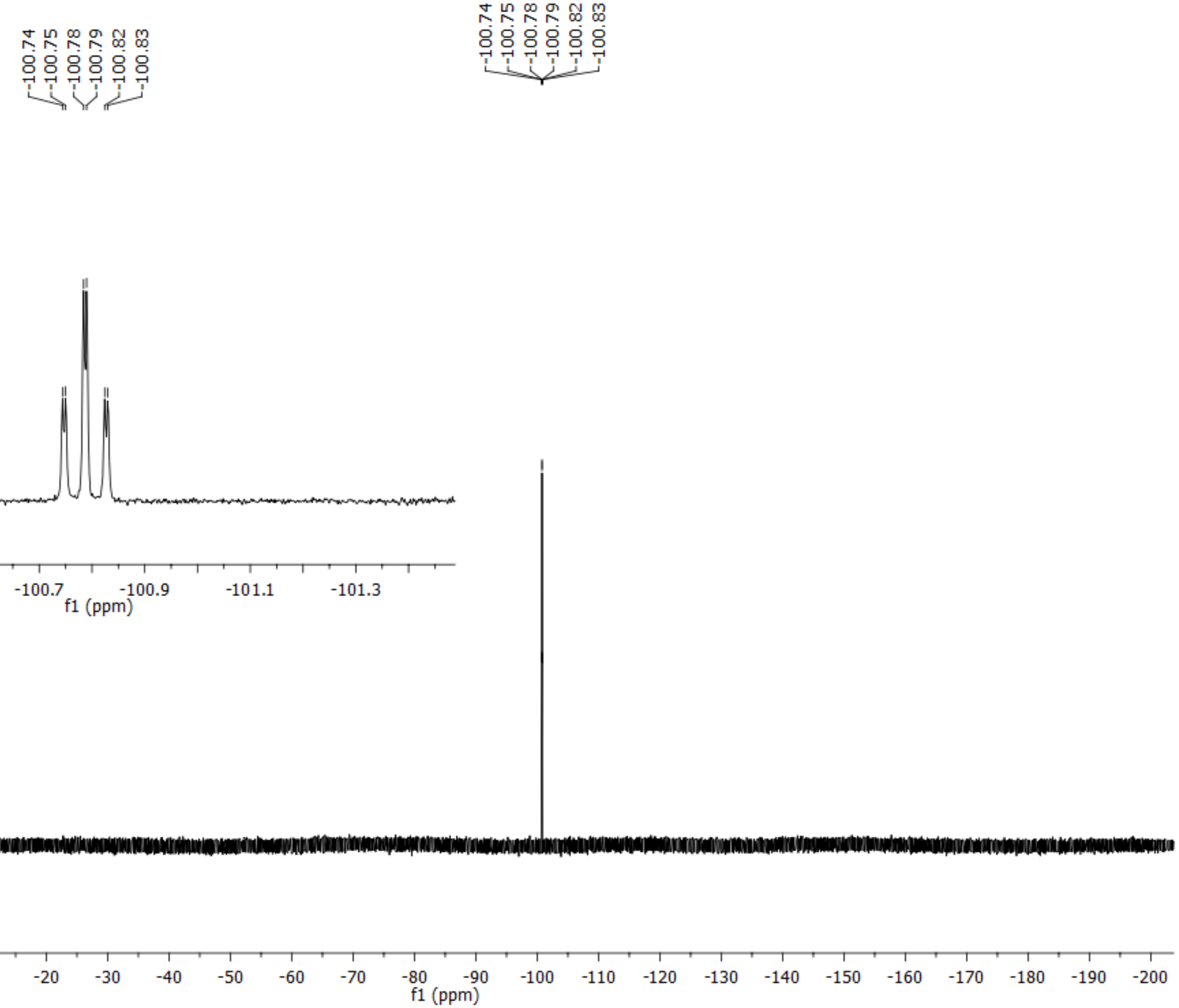

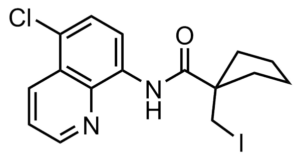

20

$CDCl_3$, 400 MHz

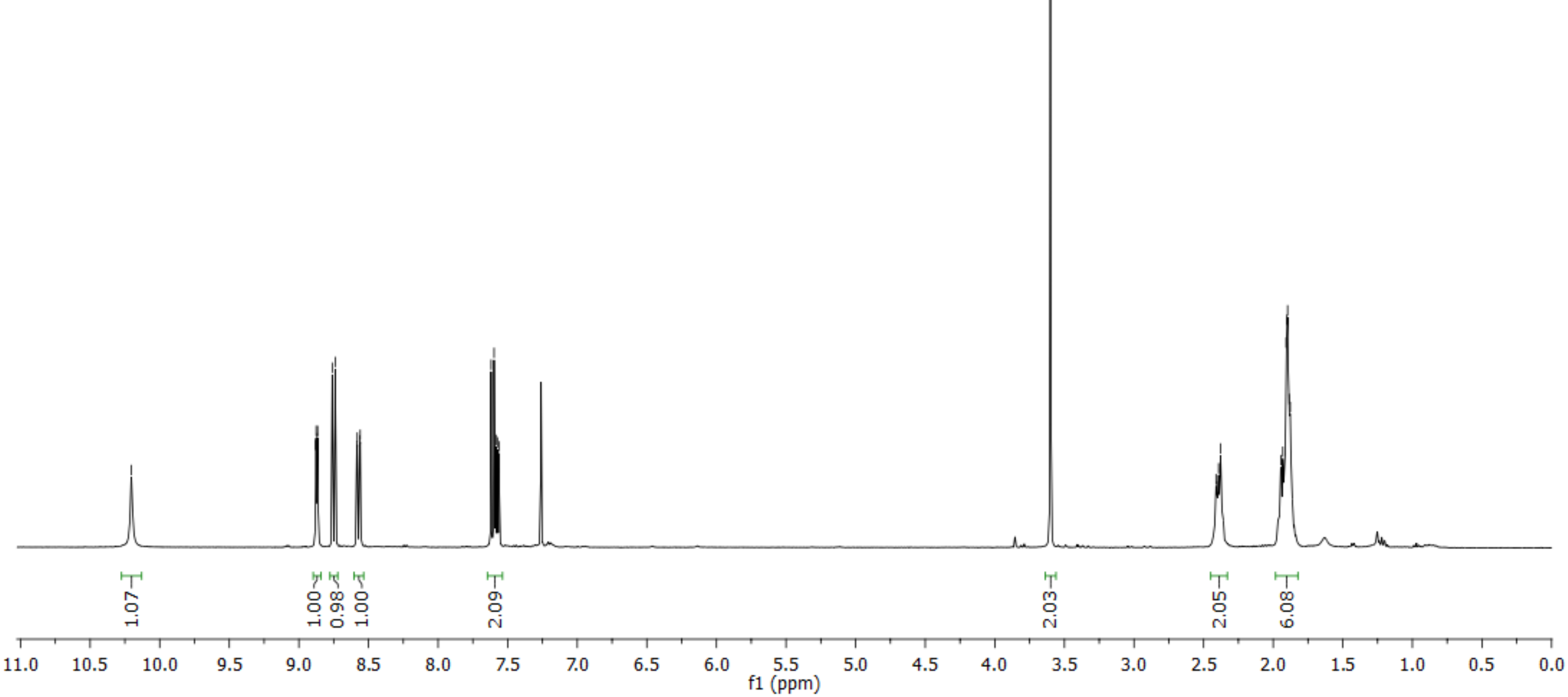

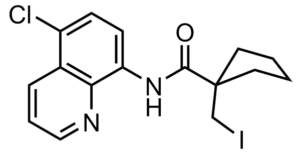

20

$CDCl_3$, 100 MHz

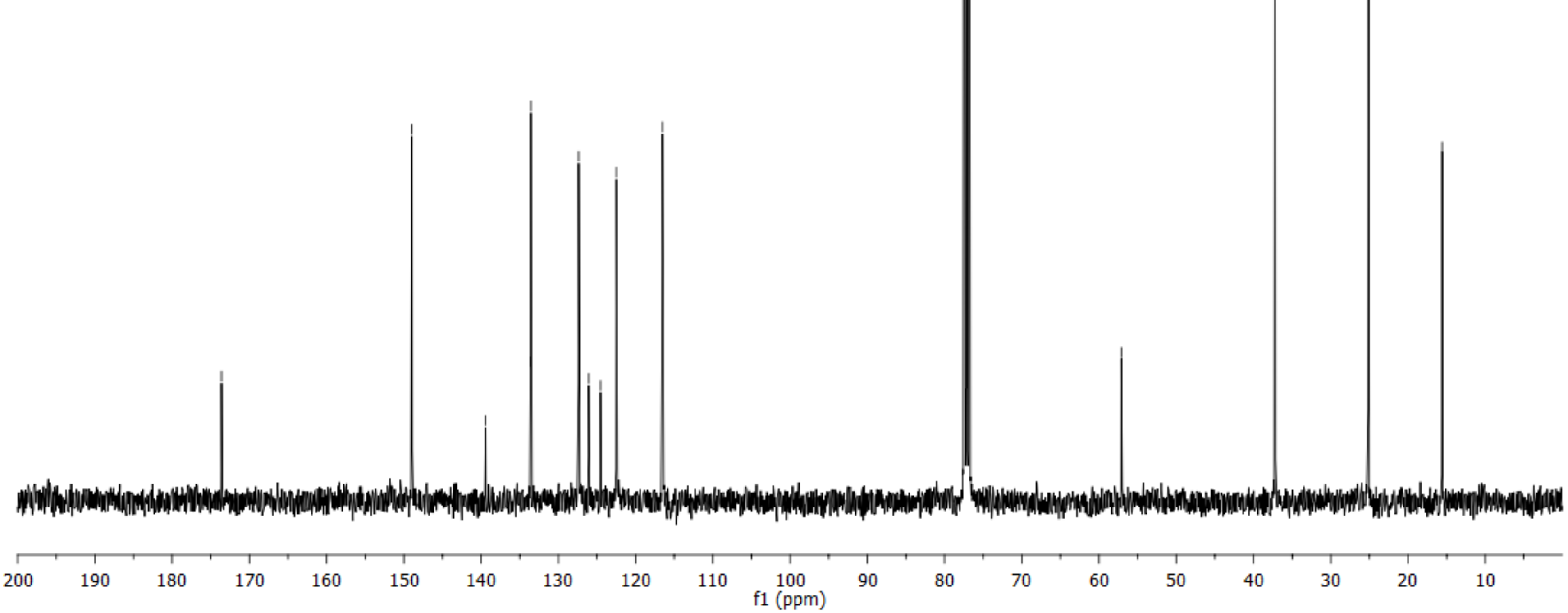

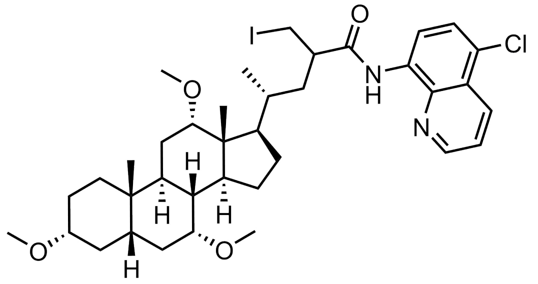

21

$CDCl_3$, 400 MHz

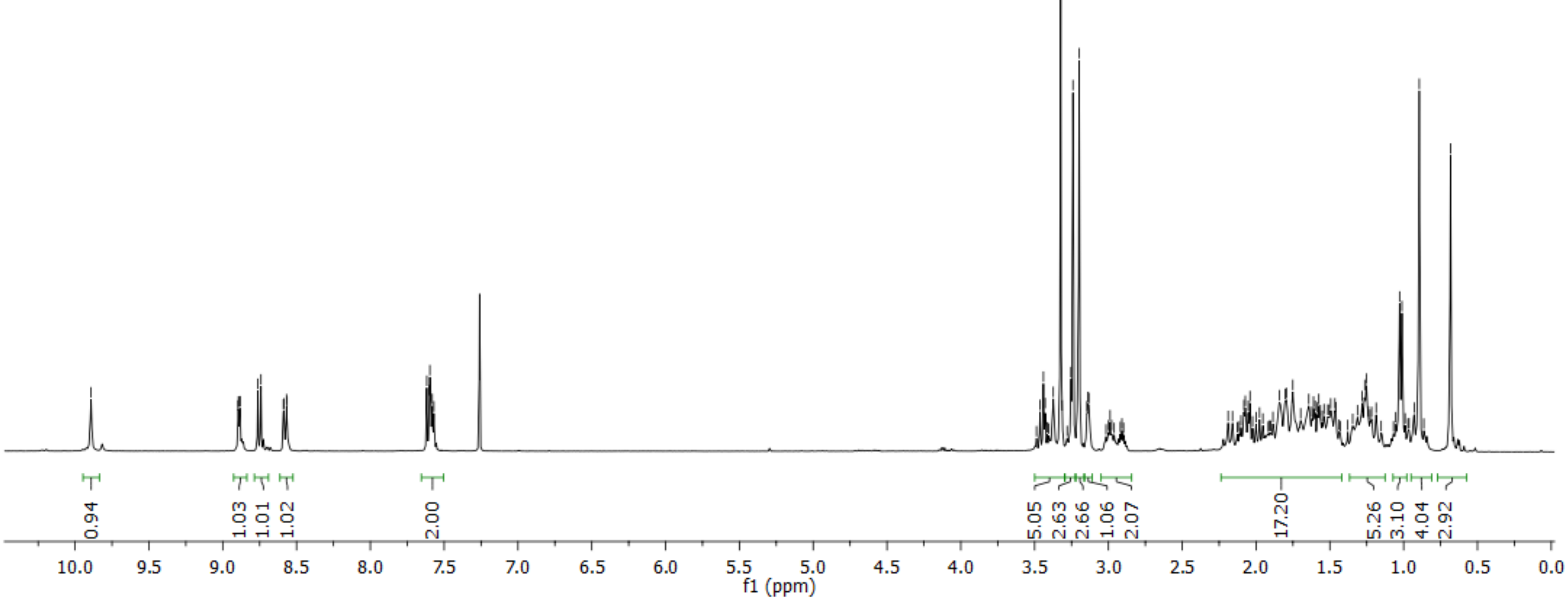

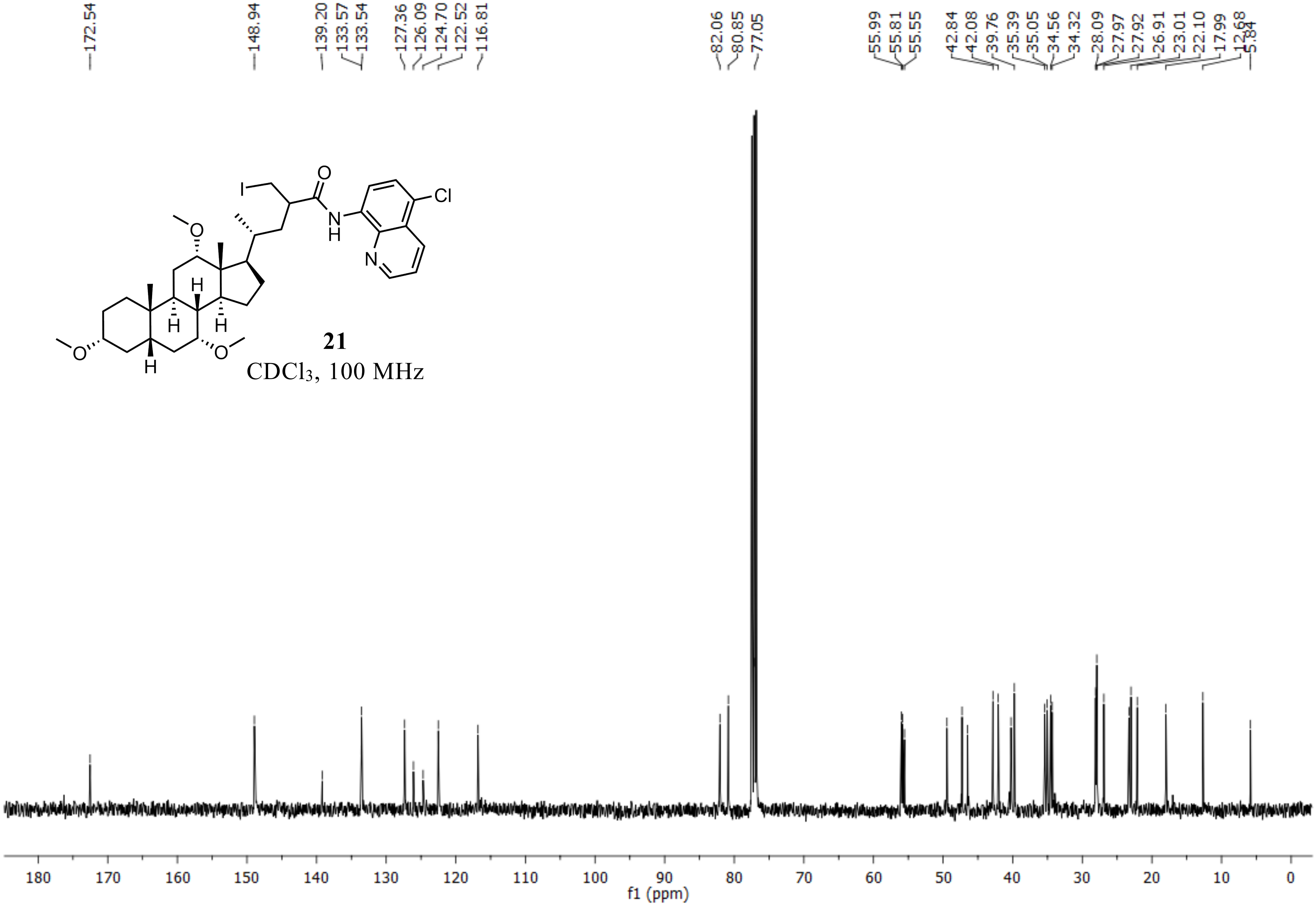

172.54
148.94
139.20
133.57
133.54
127.36
126.09
124.70
122.52
116.81
82.06
80.85
77.05
55.99
55.81
55.55
42.84
42.08
39.76
35.39
35.05
34.56
34.32
28.09
27.97
27.92
26.91
23.01
22.10
17.99
12.68
5.84
O
I
N
H
Cl
N
H
H
H
O
O
O
H
21
$CDCl_3$, 100 MHz
180 170 160 150 140 130 120 110 100 90 80 70 60 50 40 30 20 10 0
f1 (ppm)

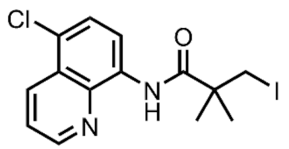

37

CDCl$_3$, 400 MHz

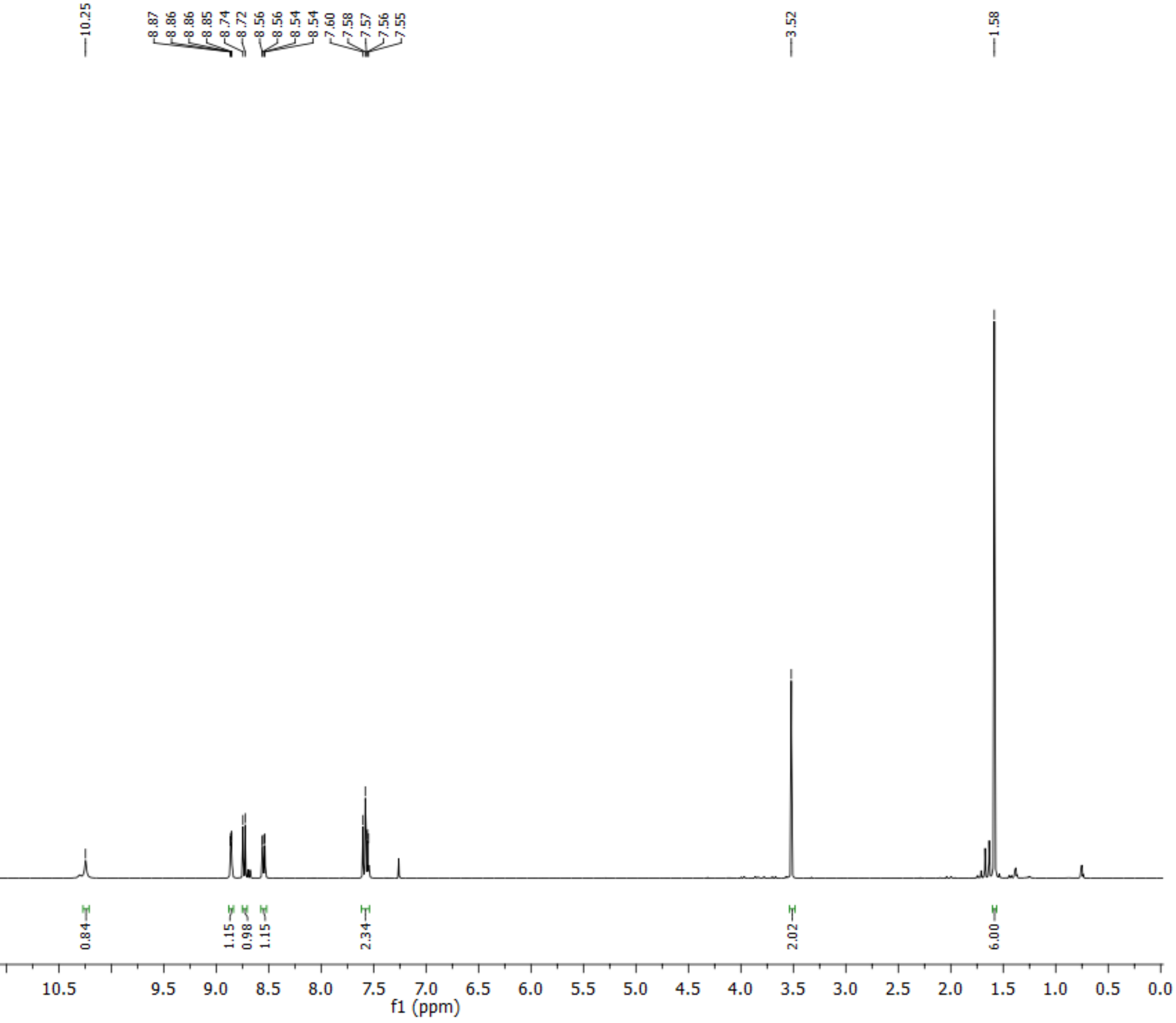

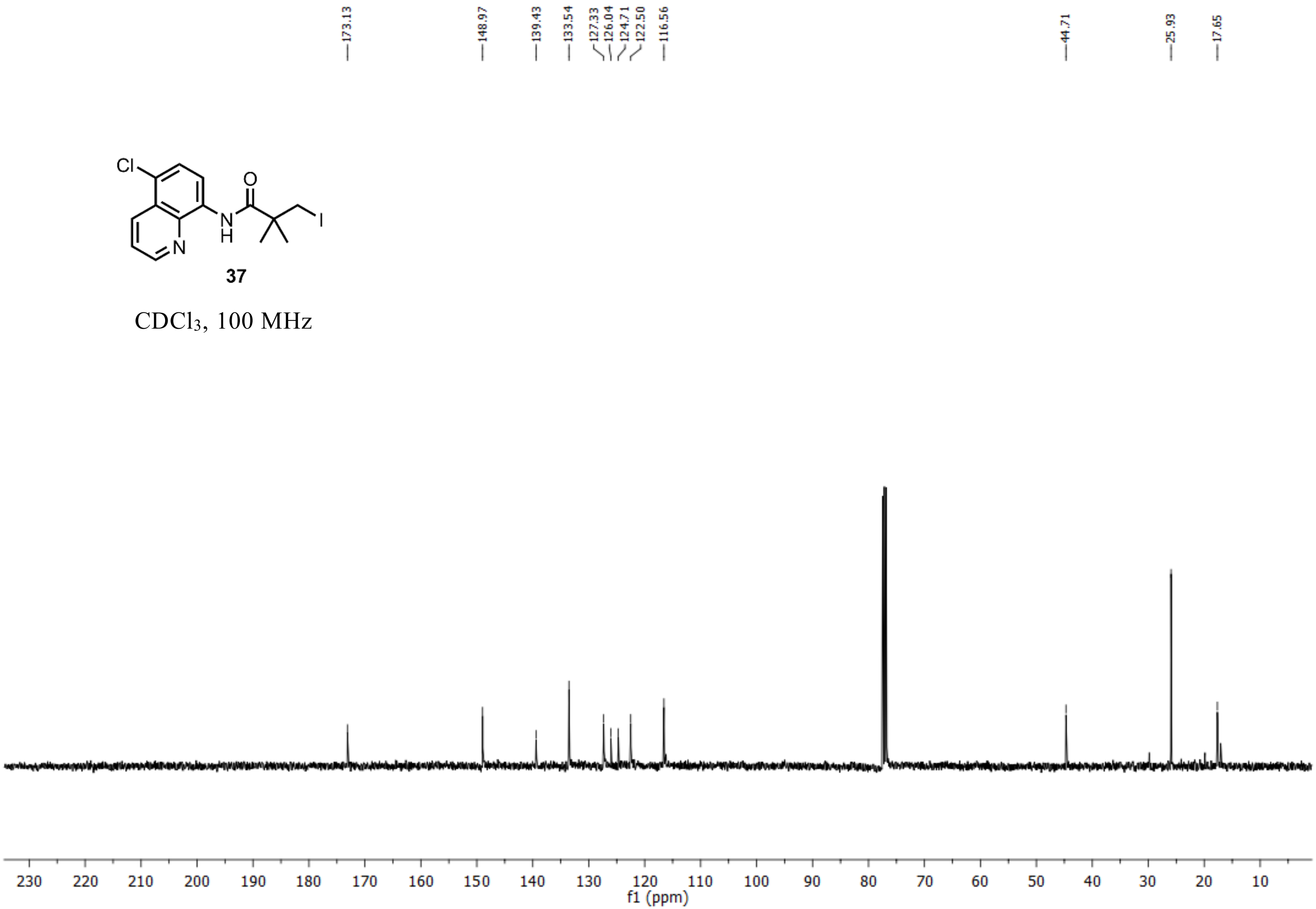
173.13
148.97
139.43
133.54
127.33
126.04
124.71
122.50
116.56
44.71
25.93
17.65
Cl
O
N
H
I
N
37
CDCl3, 100 MHz
230 220 210 200 190 180 170 160 150 140 130 120 110 100 90 80 70 60 50 40 30 20 10
f1 (ppm)

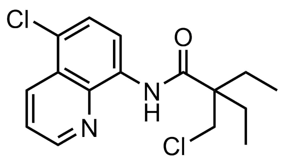

22

$CDCl_3$, 400 MHz

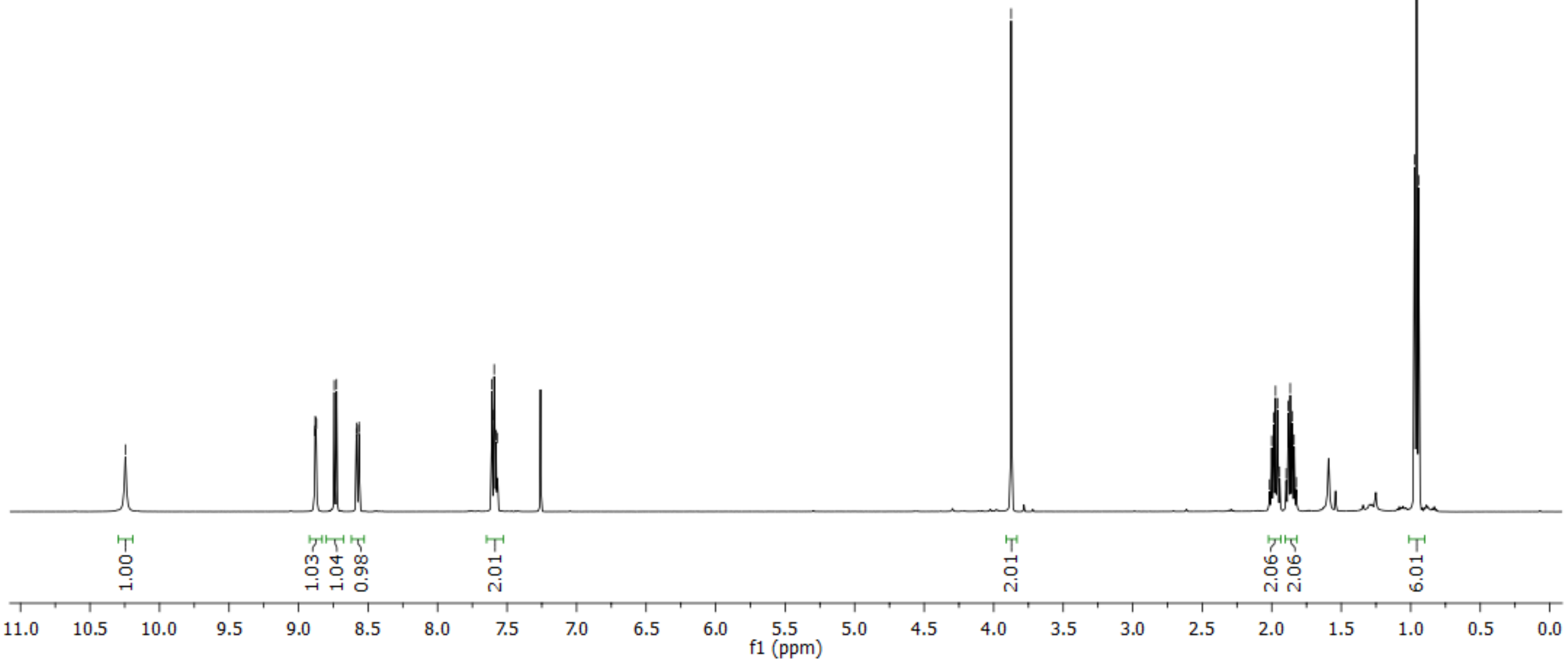

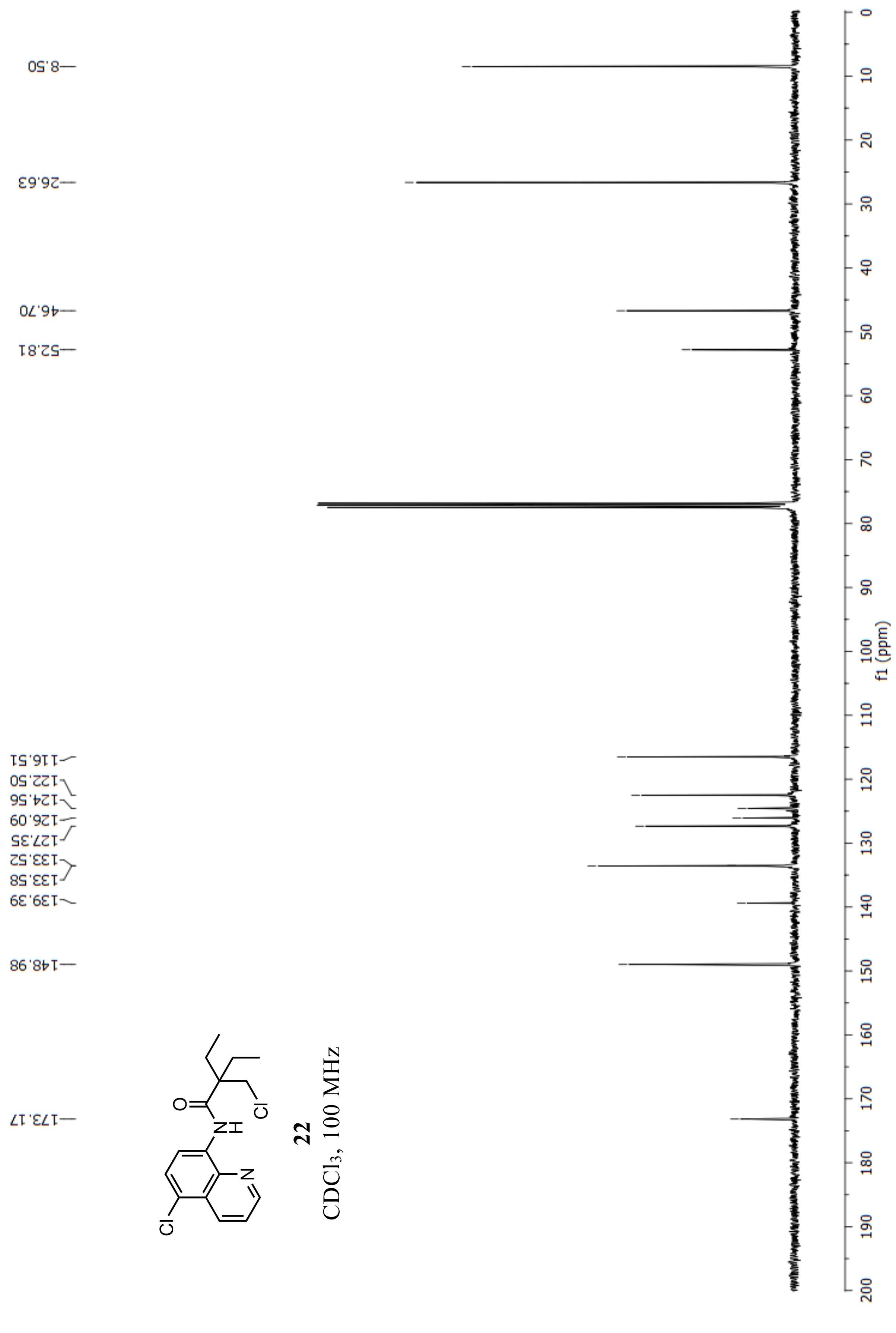

8.50
26.63
46.70
52.81
116.51
122.50
124.56
126.09
127.35
133.52
133.58
139.39
148.98
173.17
22
CDCl3, 100 MHz
f1 (ppm)

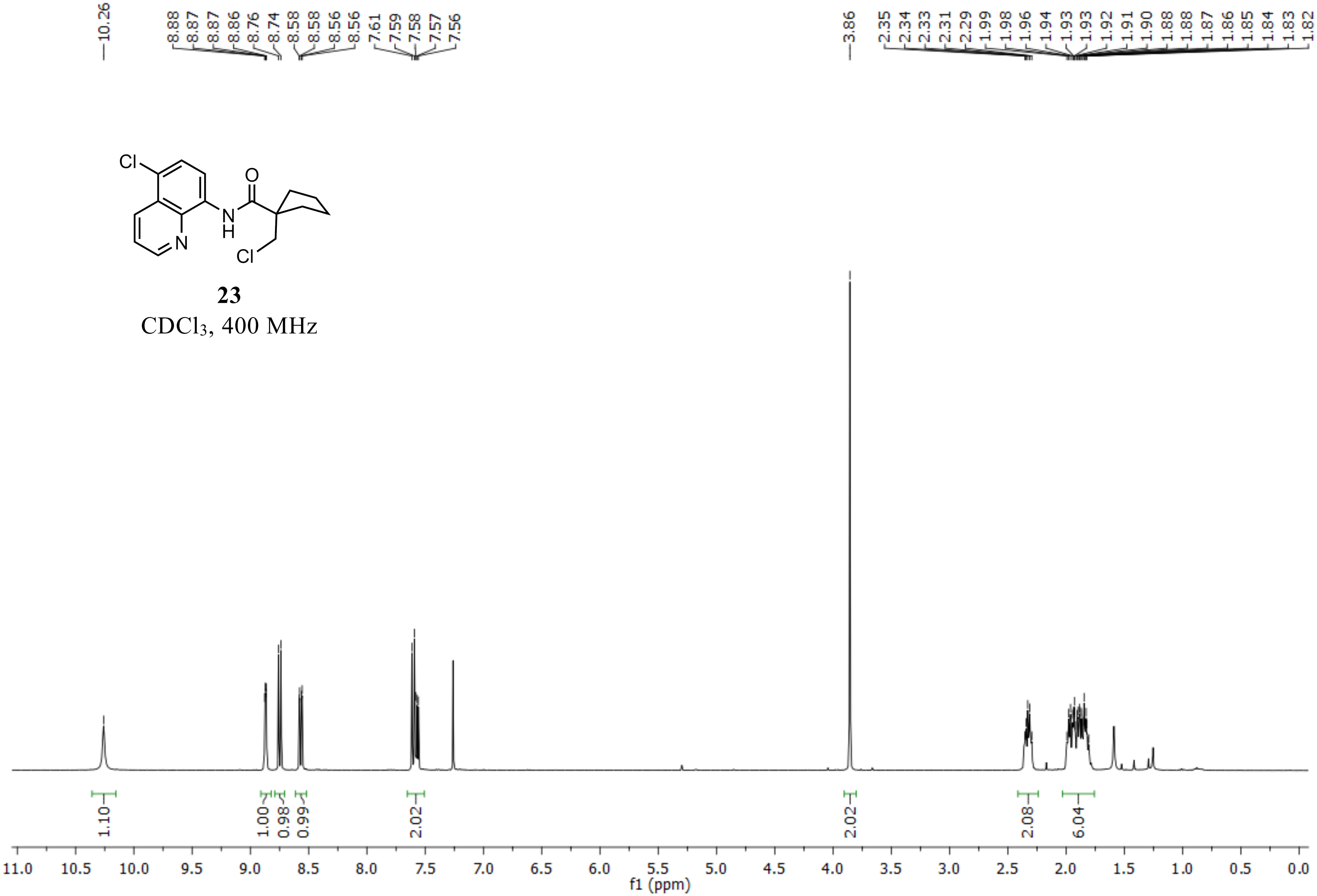

23

$CDCl_3$, 400 MHz

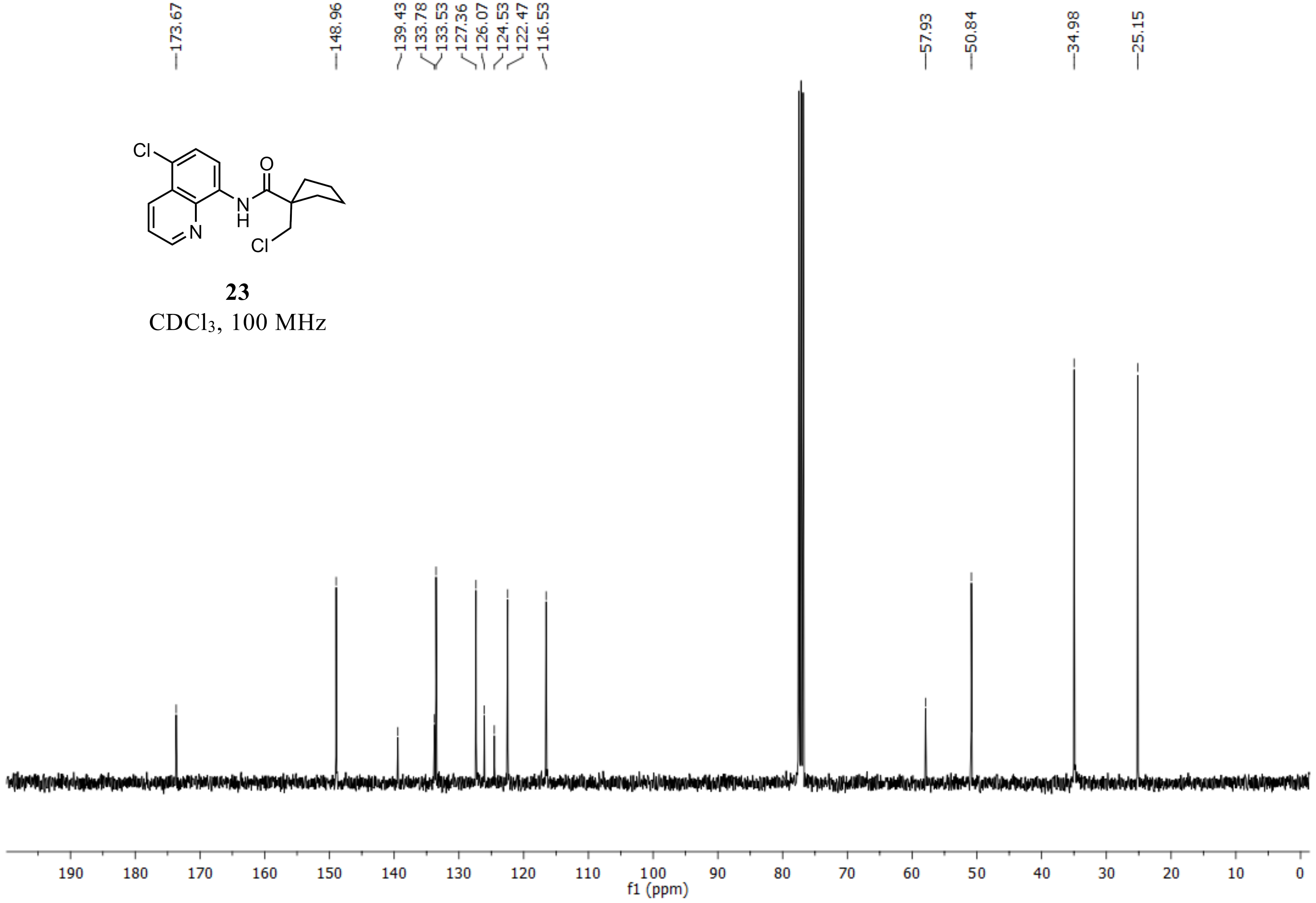

23
$CDCl_3$, 100 MHz

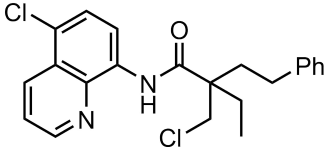

24
$CDCl_3$, 400 MHz

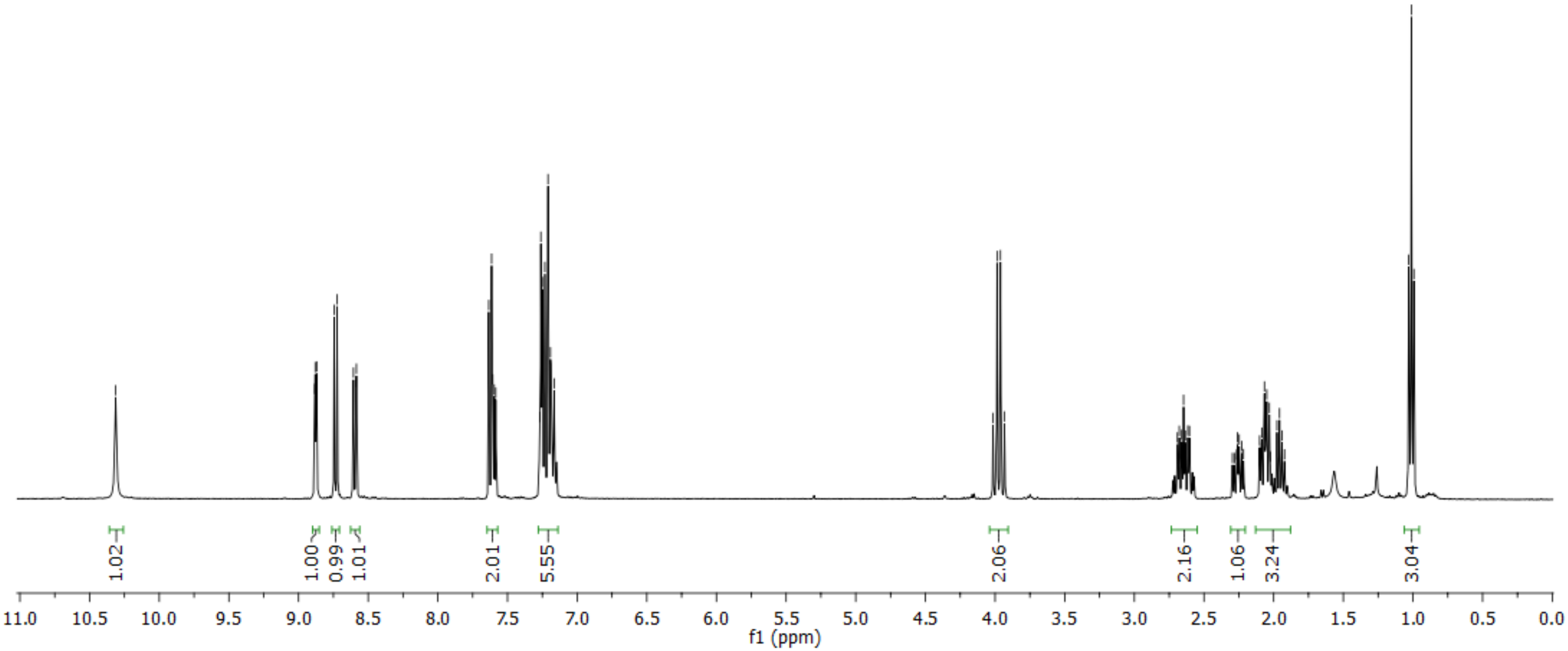

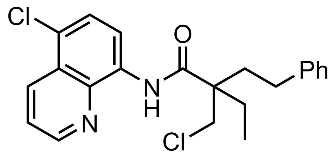

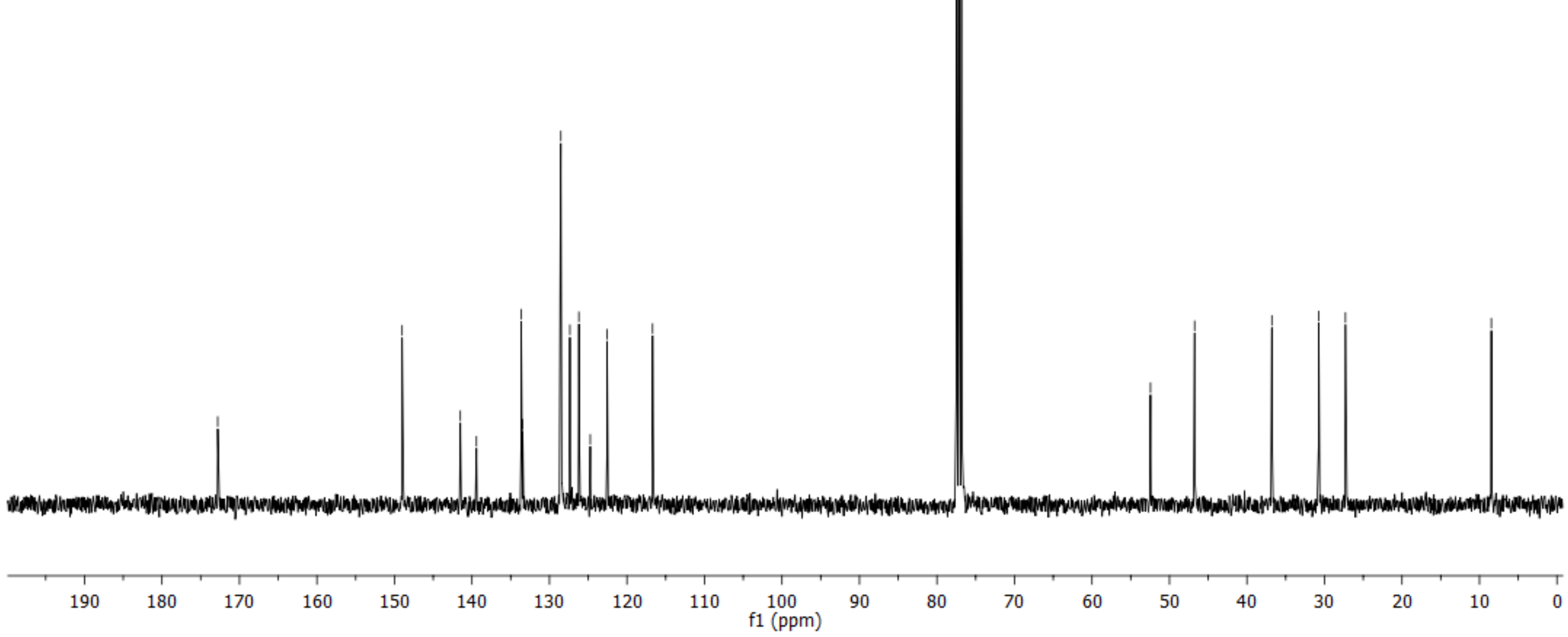

24

$CDCl_3$, 100 MHz

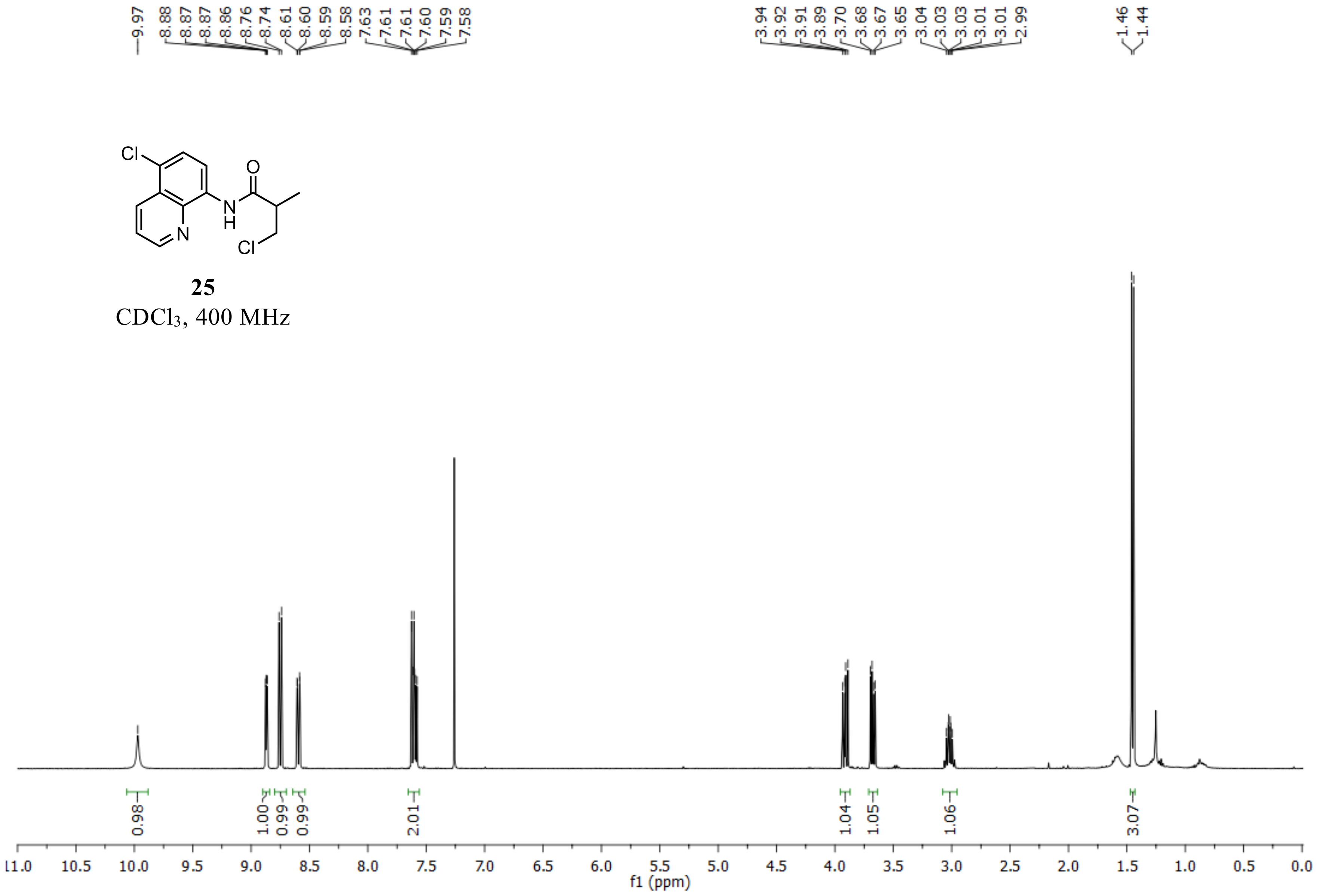

25
$CDCl_3$, 400 MHz

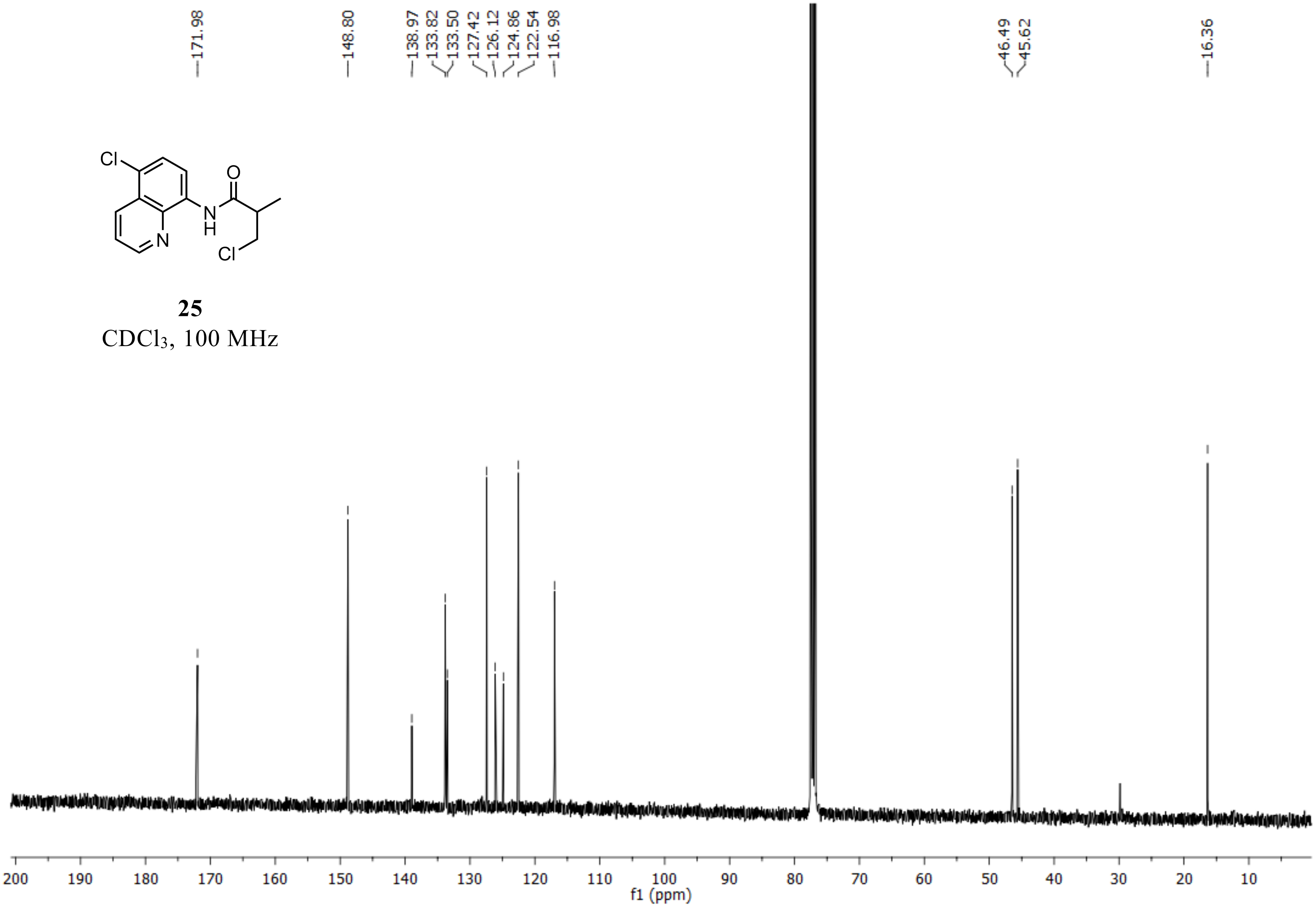

25

$CDCl_3$, 100 MHz

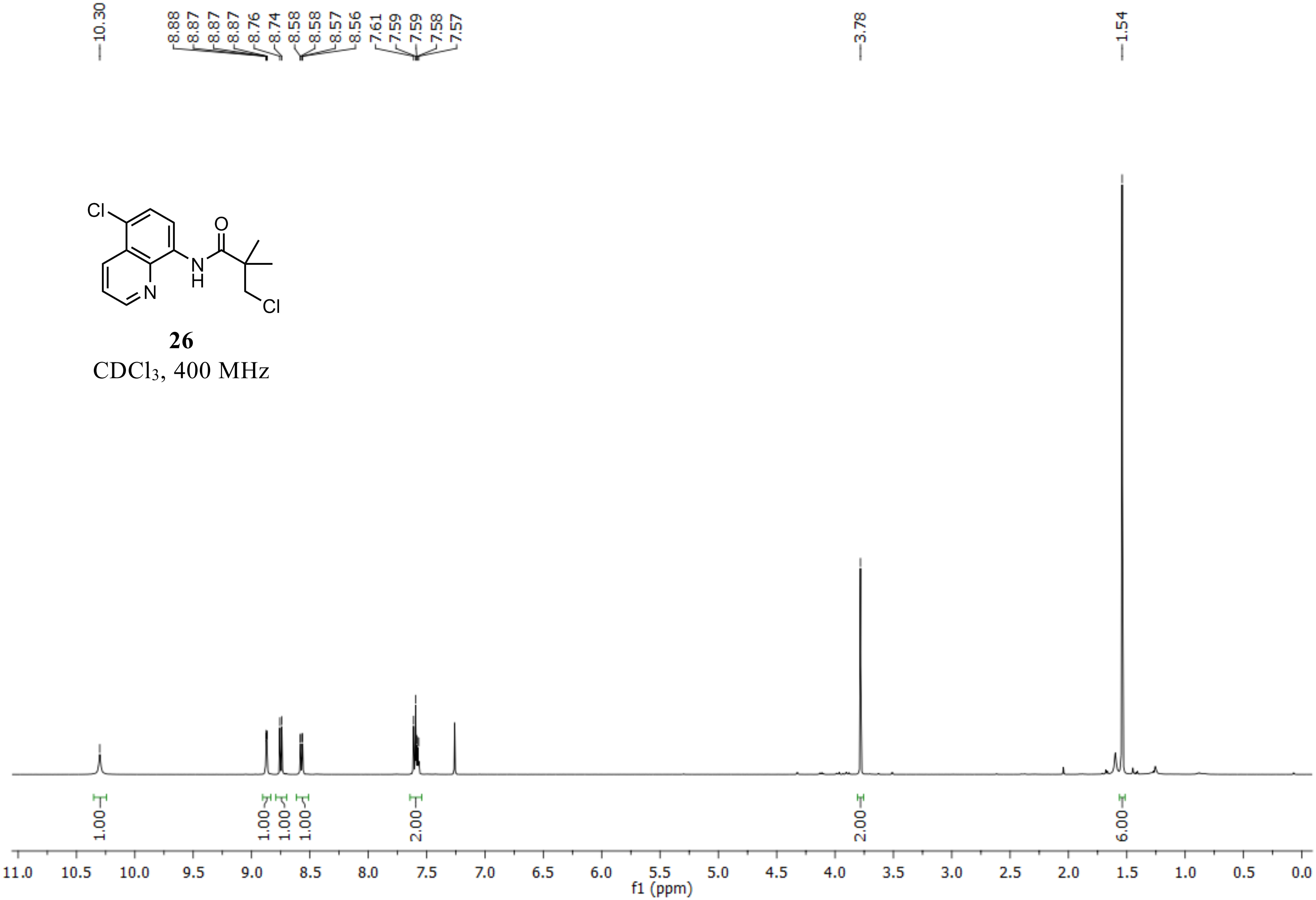

26

$CDCl_3$, 400 MHz

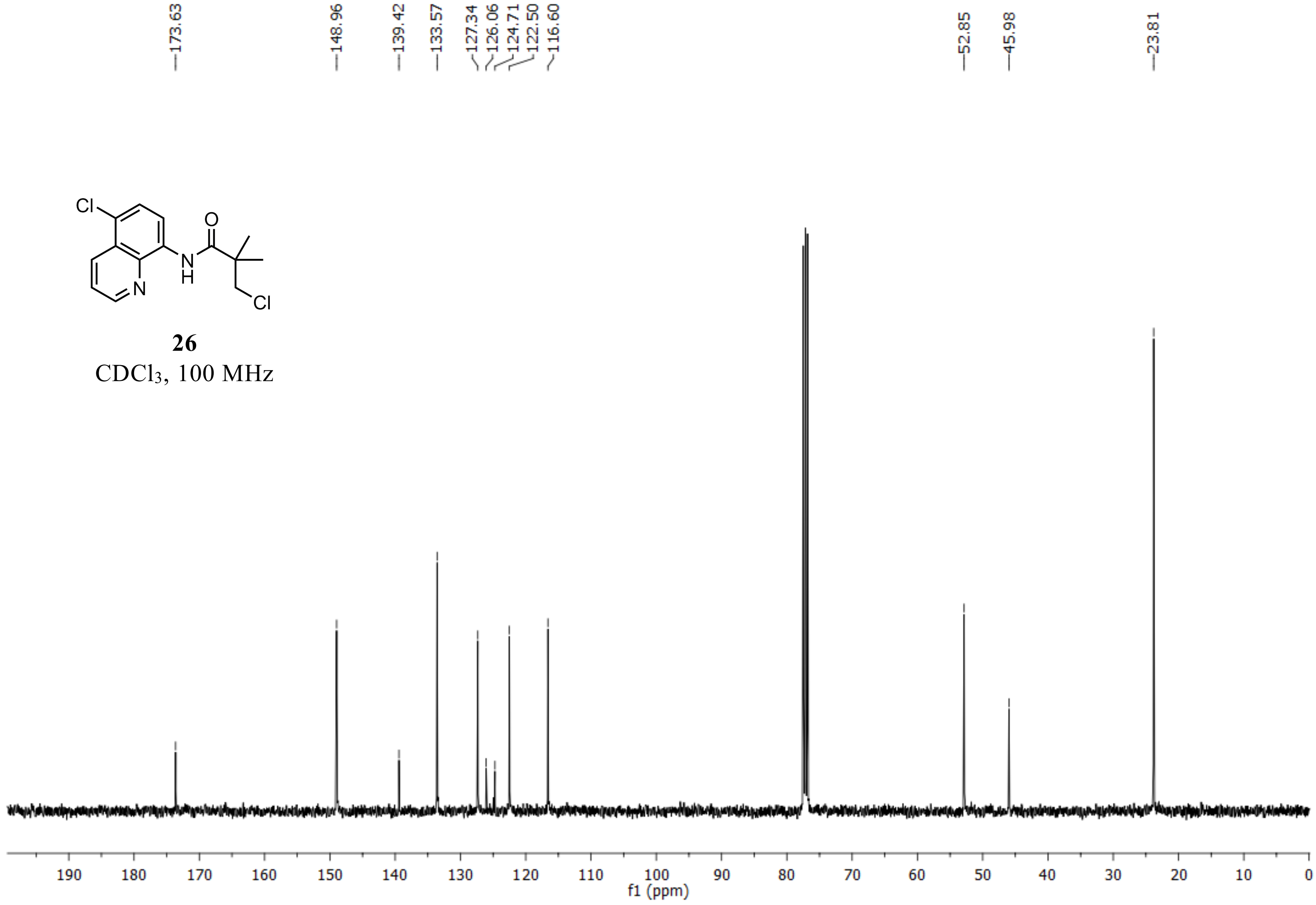

26

$CDCl_3$, 100 MHz

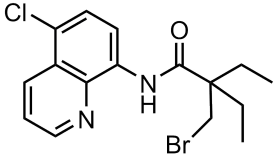

27
$CDCl_3$, 400 MHz

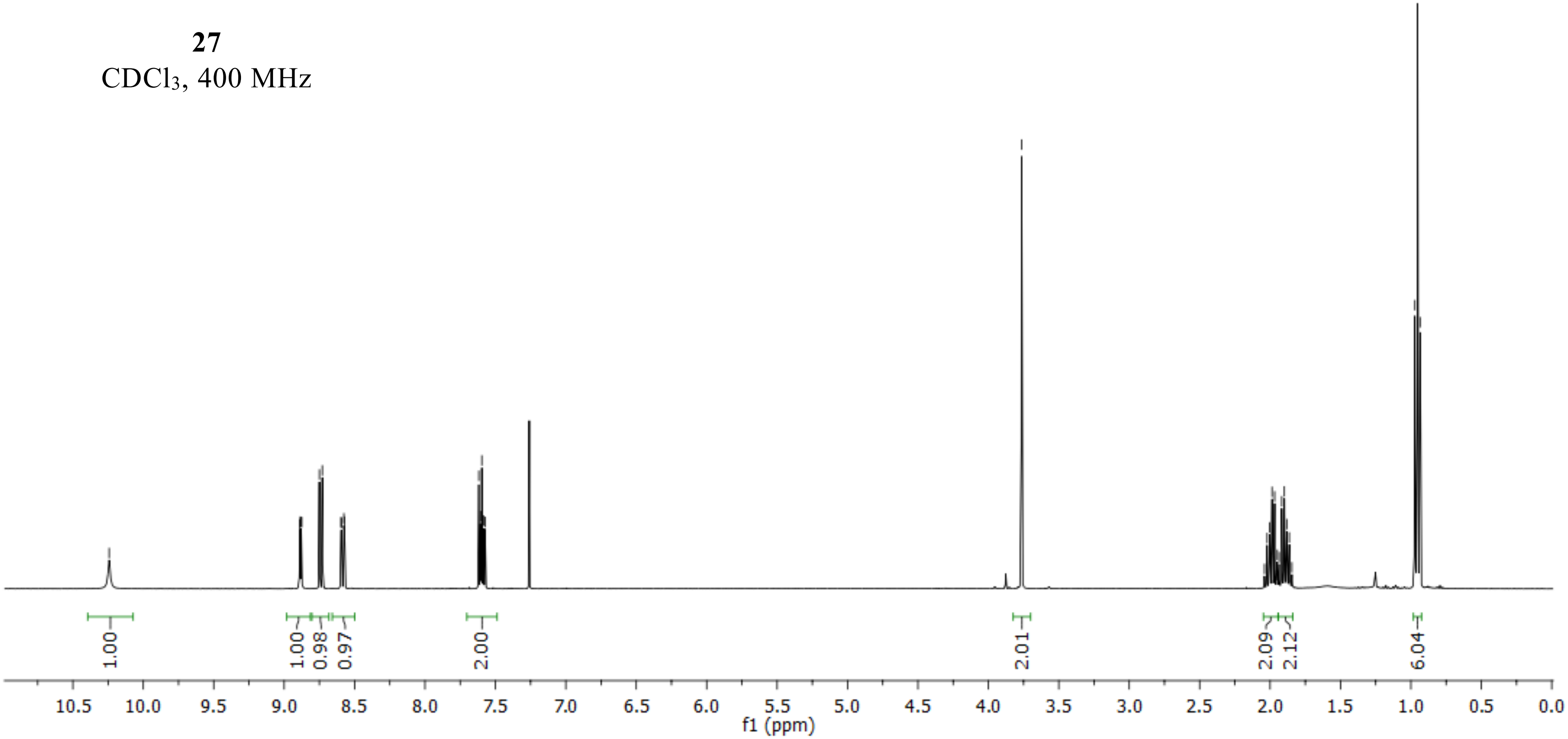

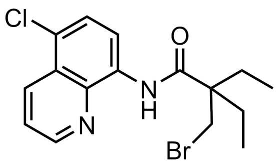

27

$CDCl_3$, 100 MHz

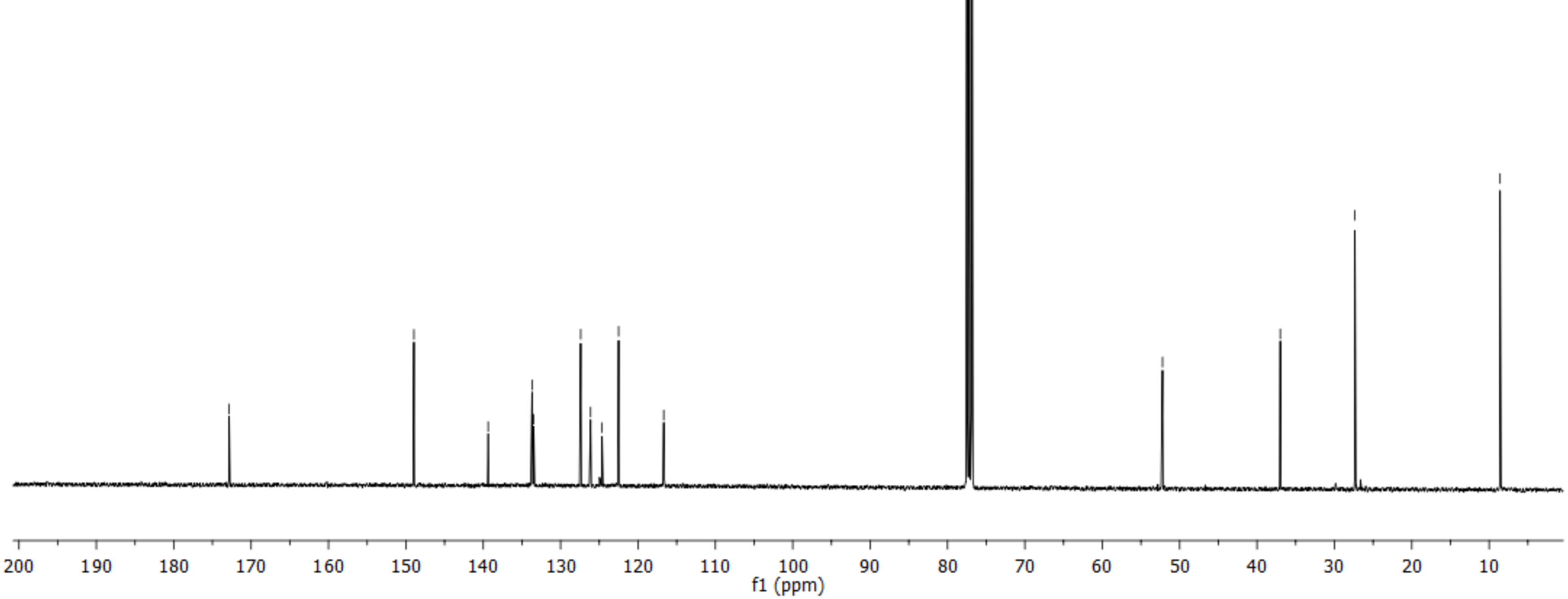

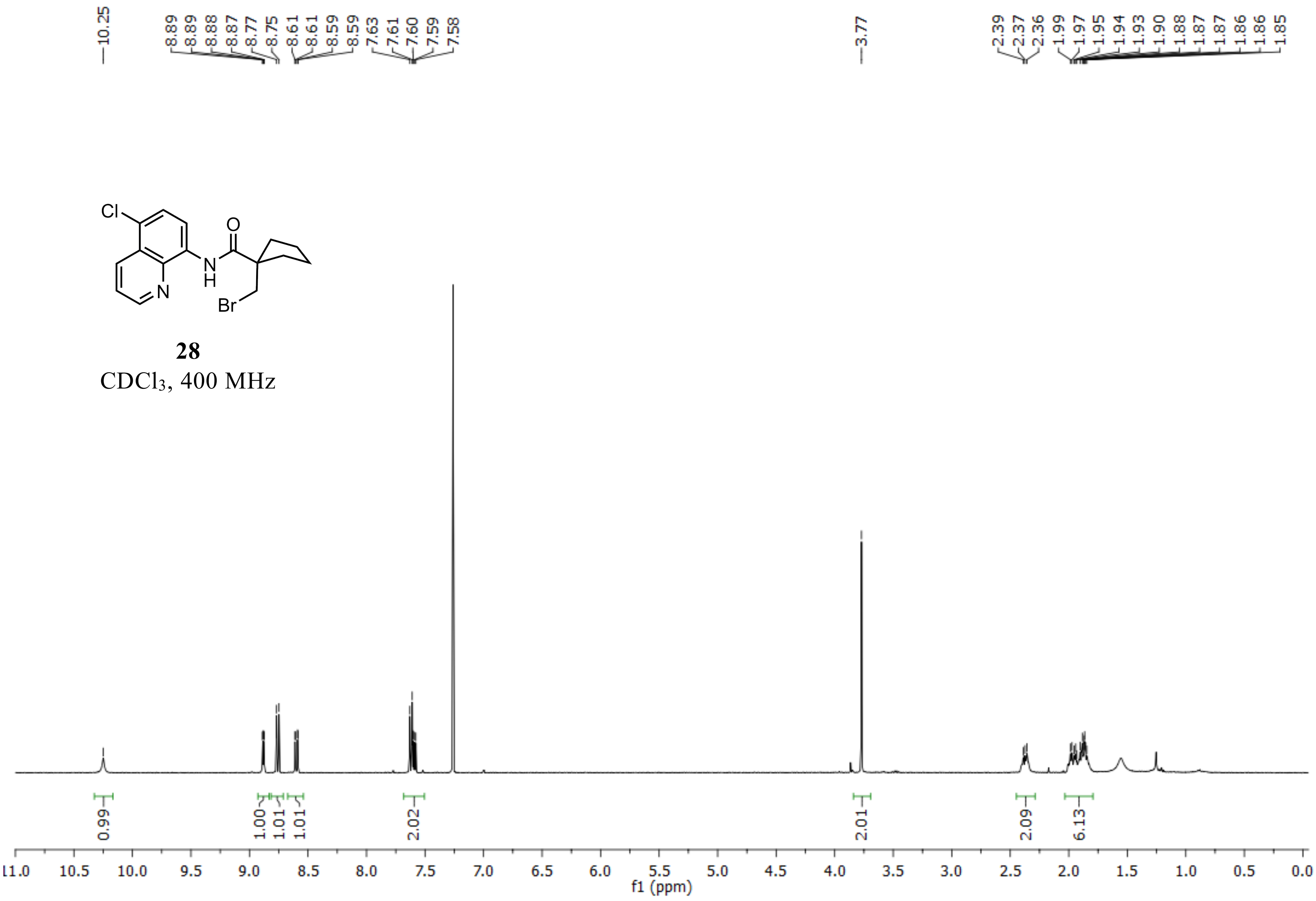

28

$CDCl_3$, 400 MHz

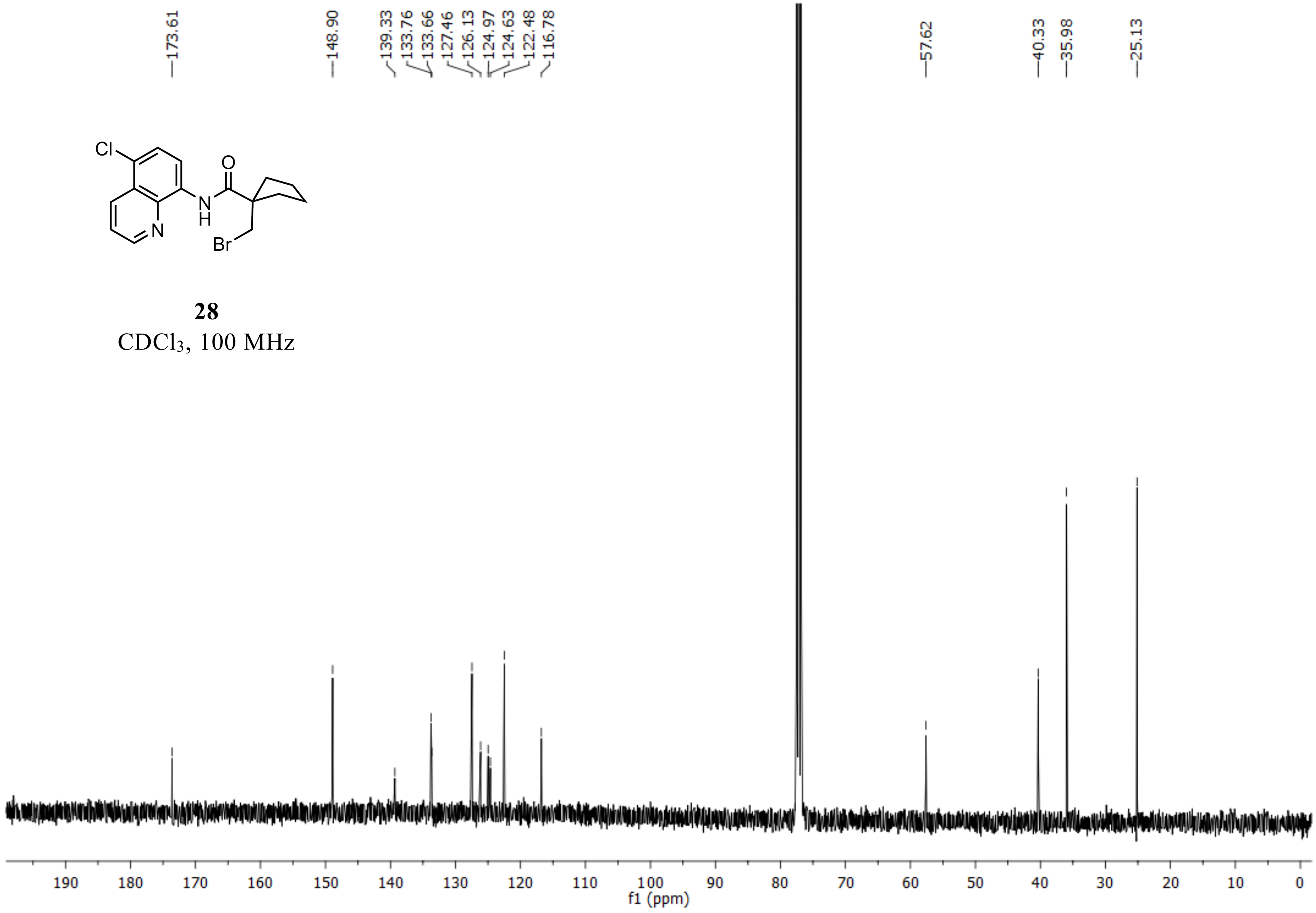

28
$CDCl_3$, 100 MHz

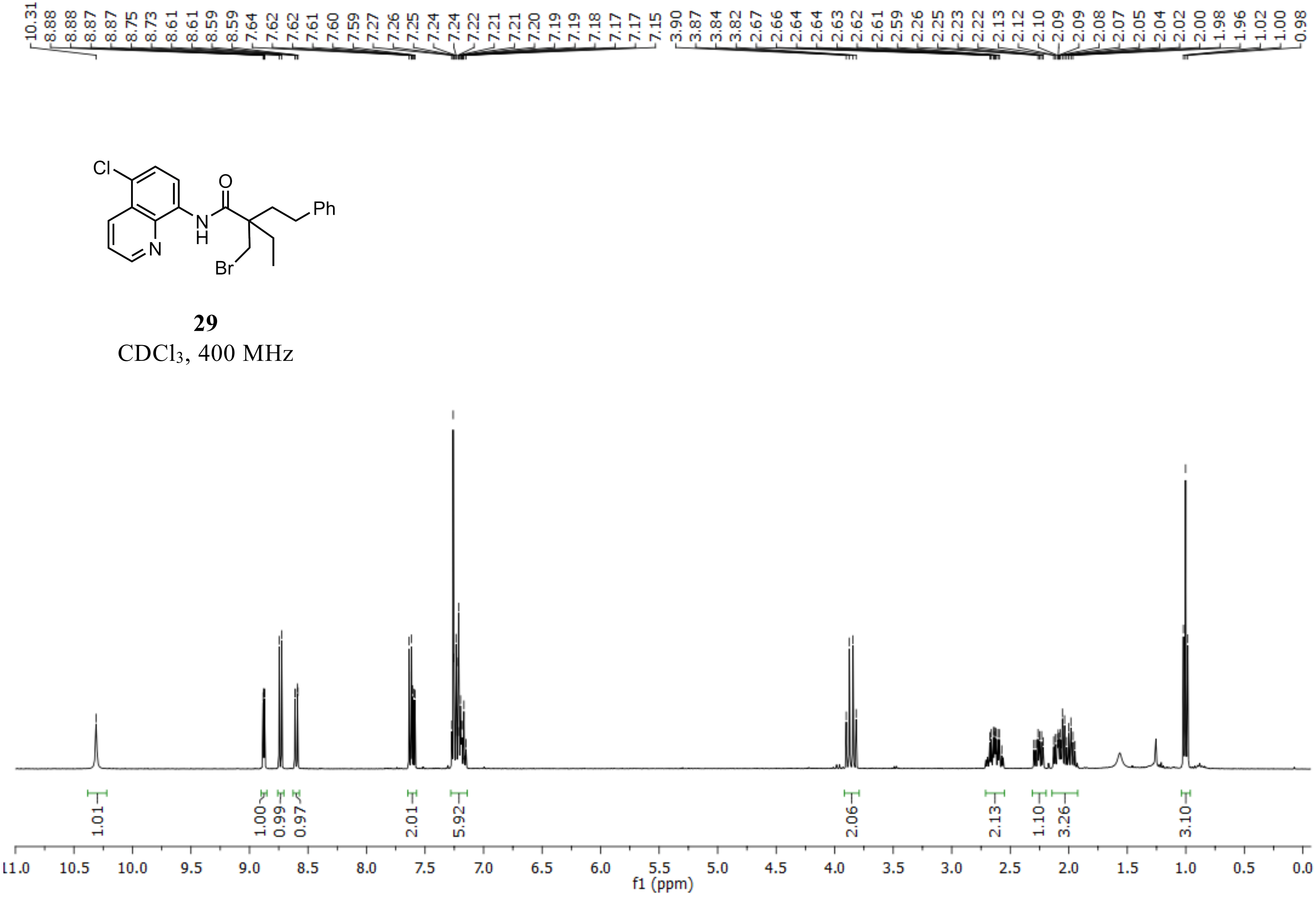

29
$CDCl_3$, 400 MHz

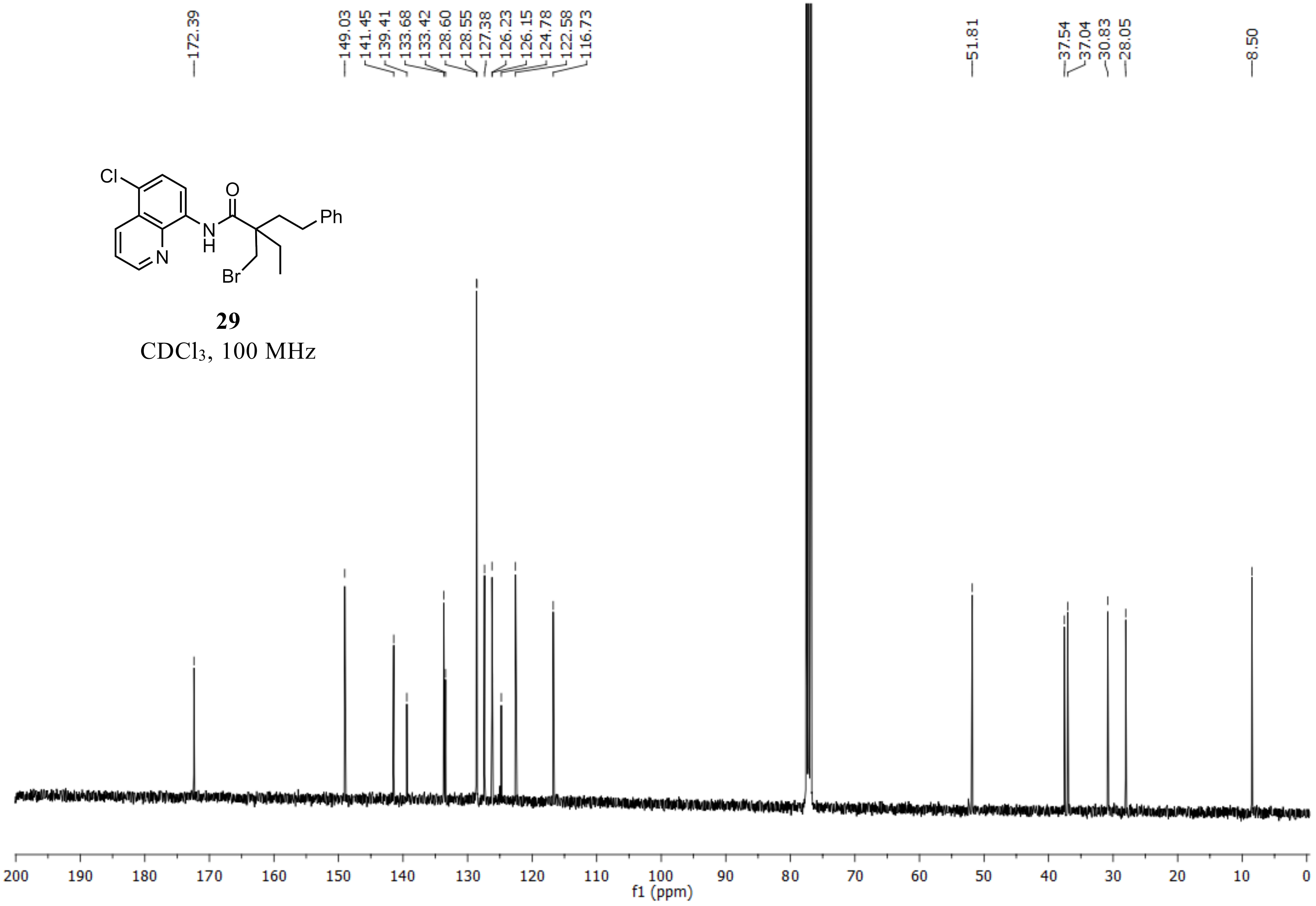

29

$CDCl_3$, 100 MHz

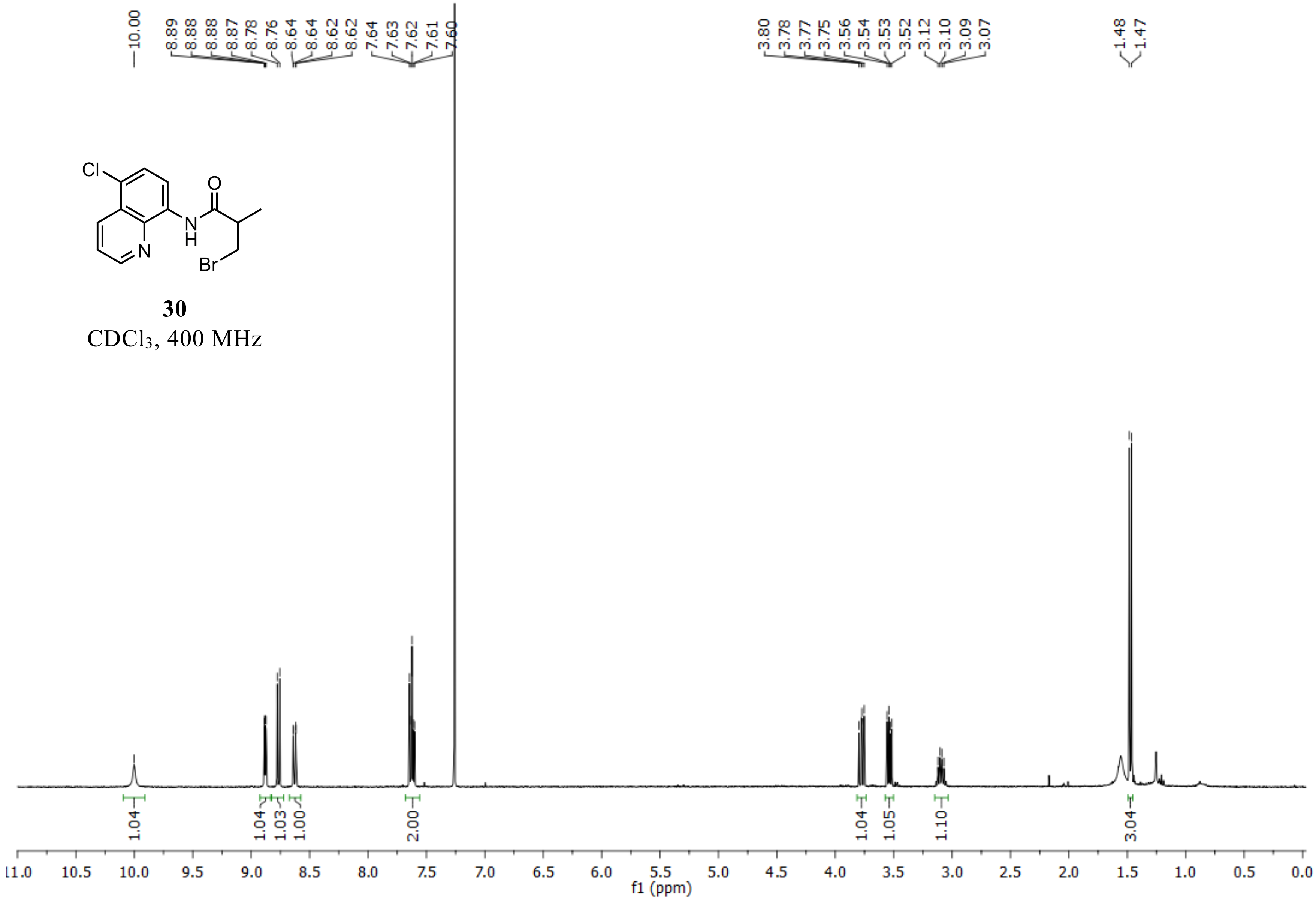

30
$CDCl_3$, 400 MHz

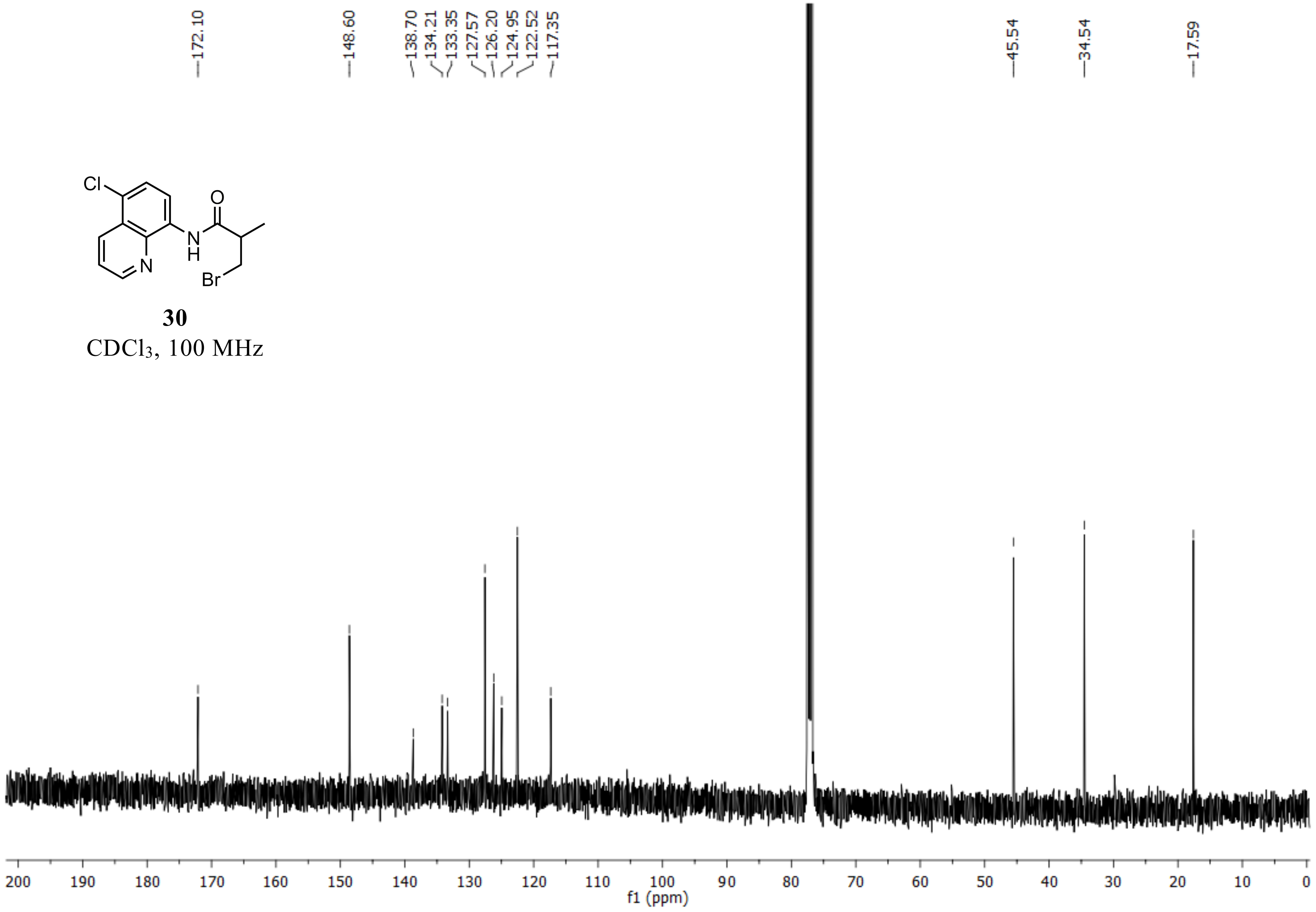

30
$CDCl_3$, 100 MHz

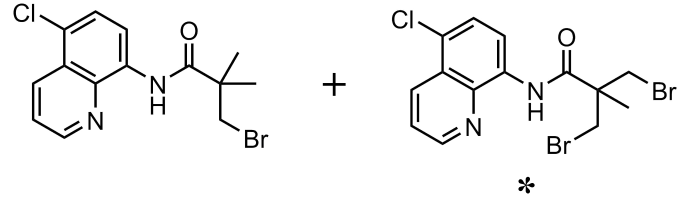

31 + 31c
$CDCl_3$, 400 MHz

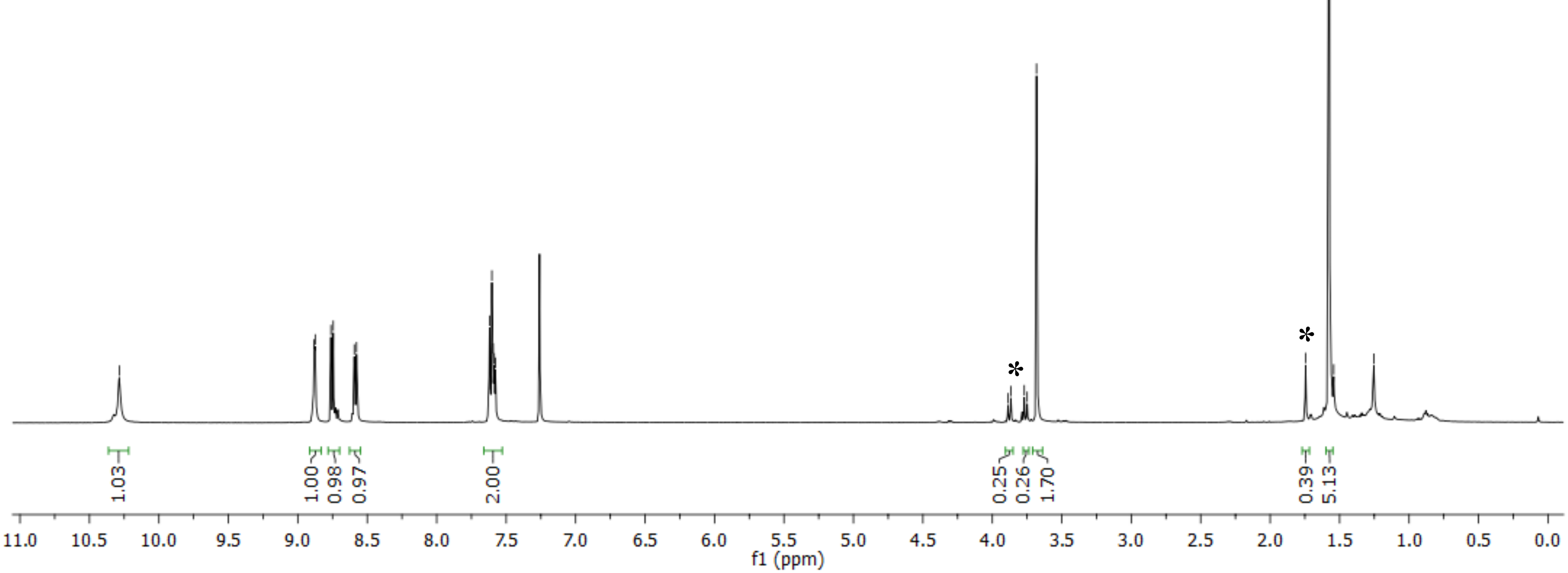

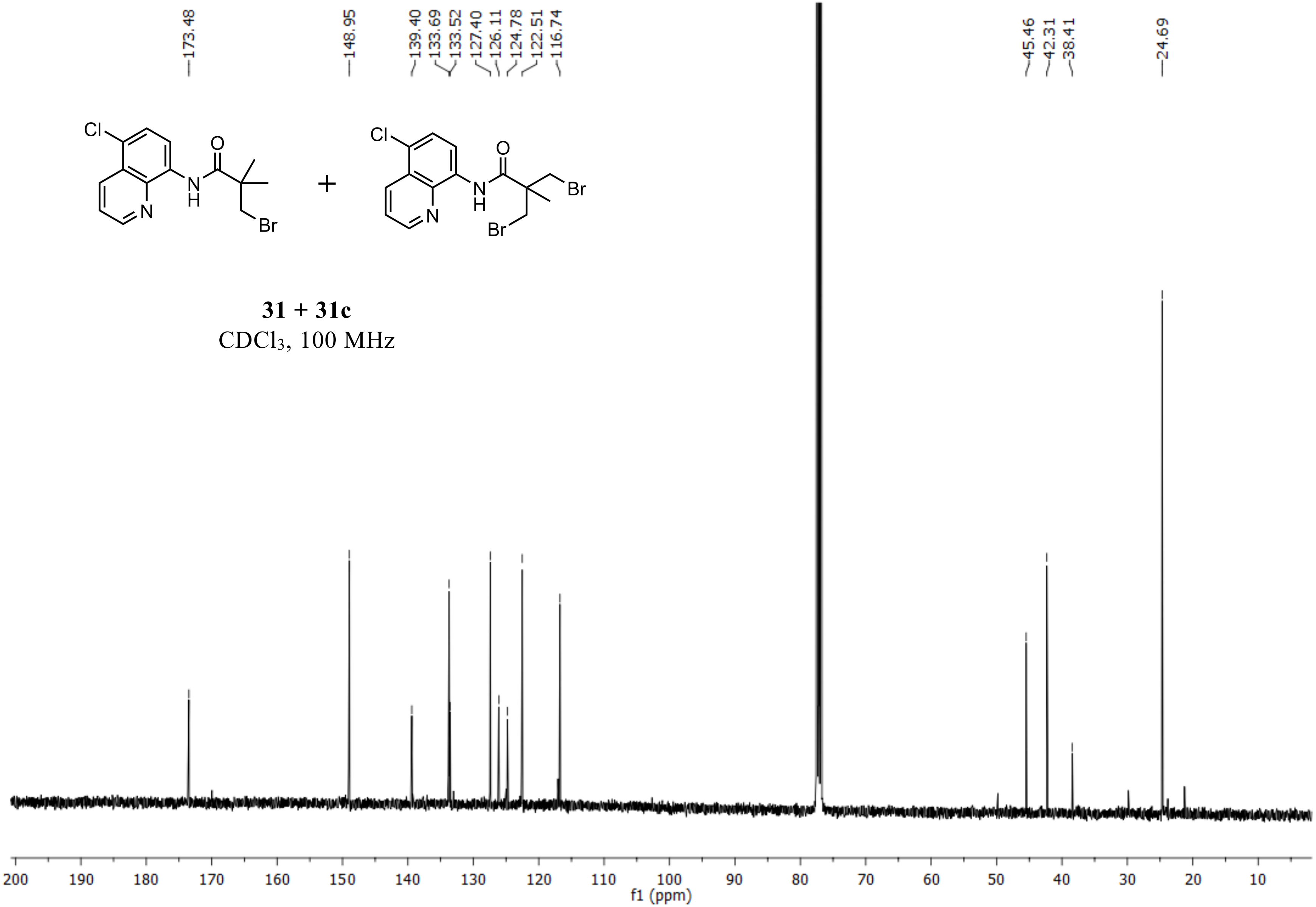

31 + 31c
$CDCl_3$, 100 MHz

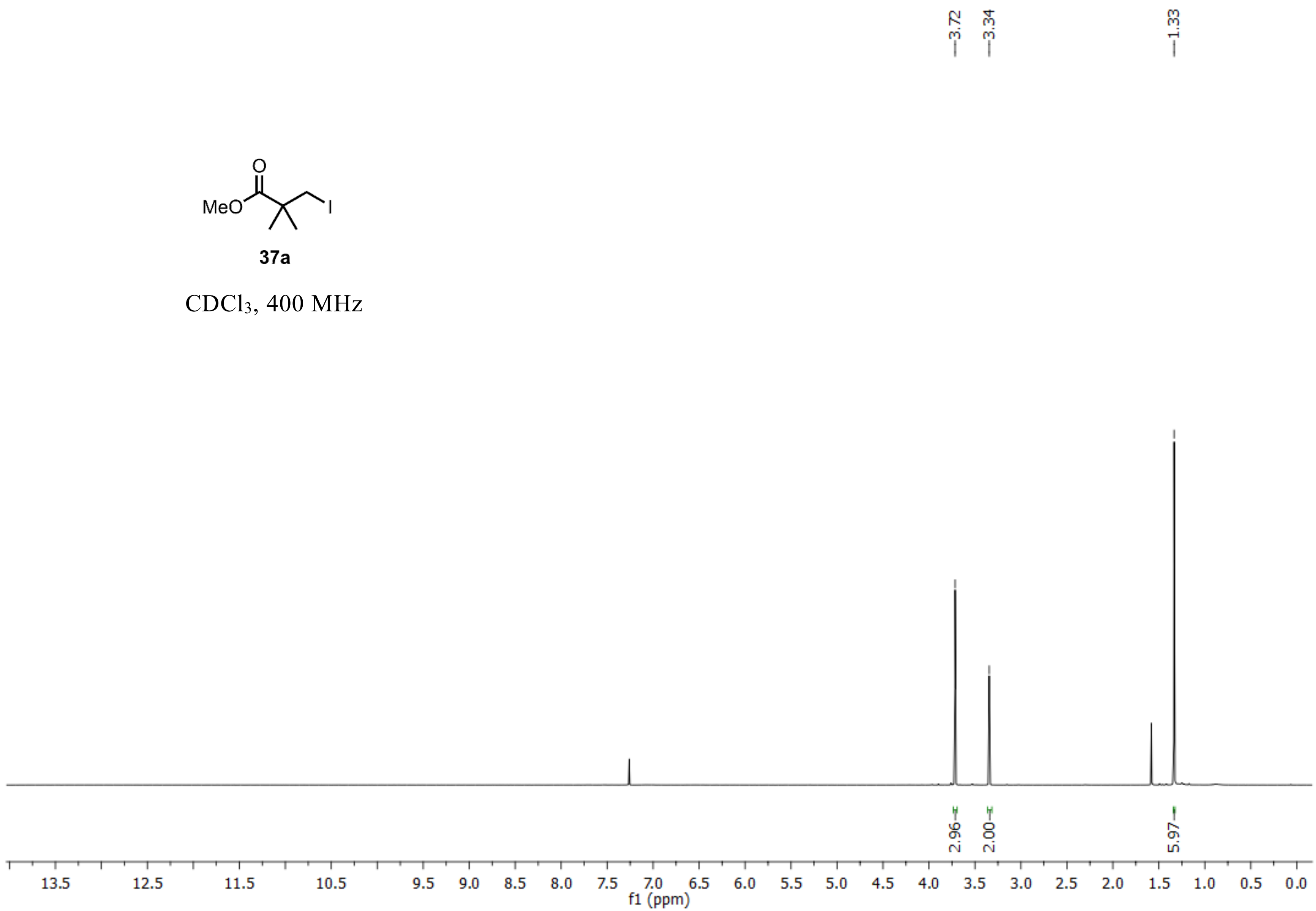

$CDCl_3$, 400 MHz

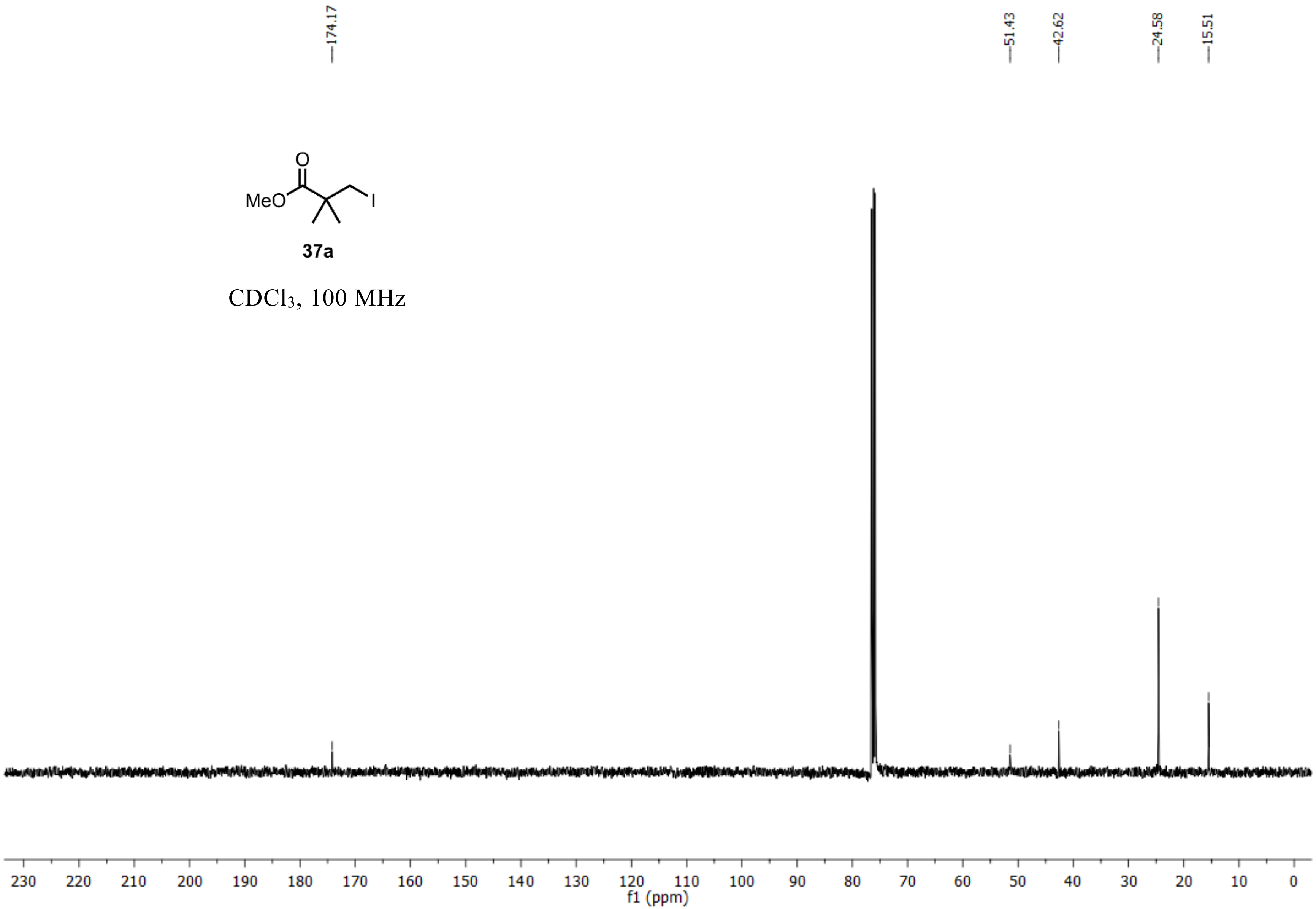
174.17
51.43
42.62
24.58
15.51
MeO
O
I
37a
CDCl3, 100 MHz
230 220 210 200 190 180 170 160 150 140 130 120 110 100 90 80 70 60 50 40 30 20 10 0
f1 (ppm)

X-Ray Data

A yellow plate like crystal was mounted on a XtaLAB Synergy, Dualflex, HyPix diffractometer employing mirror monochromated Mo Kα radiation generated from a micro-focus sealed X-ray tube. Cell constants were obtained from a least squares refinement against 7049 reflections located between 5.53 and 70.69° 2θ. Data were collected at 99.95(15) Kelvin.

The structure was solved in the triclinic space group *P*-1 by direct methods with SHELXT,[9] and extended and refined with SHELXL-2016/6.[9] The non-hydrogen atoms in the asymmetric unit were modelled with anisotropic displacement parameters. A riding atom model with group displacement parameters was used for the hydrogen atoms.

Table S6. Crystal data and structure refinement details for 1.

Identification code	shelx	
Empirical formula	C18 H20 Cl N3 O Pd	
Formula weight	436.22	
Temperature	99.95(15) K	
Wavelength	0.71073 Å	
Crystal system	Triclinic	
Space group	P -1	
Unit cell dimensions	a = 7.5074(4) Å	α= 70.420(4)°.
	b = 11.2005(6) Å	β= 78.131(4)°.
	c = 11.2581(5) Å	γ = 84.462(4)°.
Volume	872.46(8) Å^3	
Z	2	
Density (calculated)	1.661 Mg/m^3	
Absorption coefficient	1.226 mm^{-1}	
F(000)	440	
Crystal size	0.306 x 0.198 x 0.016 mm^3	
Theta range for data collection	2.774 to 37.213°.	
Index ranges	-12<=h<=12, -18<=k<=15, -18<=l<=19	
Reflections collected	19203	
Independent reflections	8018 [R(int) = 0.0455]	
Completeness to theta = 25.242°	99.7 %	
Absorption correction	Gaussian	

Max. and min. transmission	1.000 and 0.580
Refinement method	Full-matrix least-squares on F^2
Data / restraints / parameters	8018 / 0 / 220
Goodness-of-fit on F^2	1.060
Final R indices [I>2sigma(I)]	R1 = 0.0500, wR2 = 0.1073
R indices (all data)	R1 = 0.0686, wR2 = 0.1152
Extinction coefficient	n/a
Largest diff. peak and hole	1.720 and -2.367 e.Å^{-3}

Table S7. Atomic coordinates (x 10^4) and equivalent isotropic displacement parameters (Å^{2}x 10^3) for 1. U(eq) is defined as one third of the trace of the orthogonalized U^{ij} tensor.

	x	y	z	U(eq)
C(1)	8244(4)	4777(2)	4072(3)	25(1)
C(2)	9285(4)	5007(2)	2841(3)	29(1)
C(3)	10031(4)	6159(2)	2213(3)	26(1)
C(4)	9752(3)	7120(2)	2810(2)	23(1)
C(5)	10449(3)	8344(2)	2251(2)	24(1)
C(6)	10179(4)	9199(2)	2918(3)	25(1)
C(7)	9156(4)	8867(2)	4172(2)	24(1)
C(8)	8379(3)	7693(2)	4771(2)	21(1)
C(9)	8705(3)	6800(2)	4070(2)	20(1)
C(10)	6708(4)	7984(2)	6768(2)	23(1)
C(11)	5349(4)	7302(2)	7968(2)	24(1)
C(12)	5253(4)	5880(2)	8140(3)	29(1)
C(13)	3466(4)	7980(2)	7799(3)	26(1)
C(14)	2860(4)	8037(3)	6571(3)	31(1)
C(15)	5890(4)	7497(3)	9140(2)	28(1)
C(16)	7816(4)	7014(3)	9358(3)	38(1)
C(17)	5181(4)	2706(2)	7304(3)	25(1)
C(18)	4708(4)	1388(2)	7605(3)	33(1)
N(1)	7947(3)	5630(2)	4681(2)	23(1)
N(2)	7277(3)	7260(2)	5977(2)	22(1)
N(3)	5549(3)	3736(2)	7070(2)	24(1)
O(1)	7166(3)	9077(2)	6544(2)	29(1)
Pd(1)	6418(1)	5526(1)	6511(1)	21(1)
Cl(1)	11694(1)	8788(1)	688(1)	31(1)

Table S8. Bond lengths [Å] and angles [°] for 1.

C(1)-N(1)	1.330(3)
C(1)-C(2)	1.398(4)
C(1)-H(1)	0.9300
C(2)-C(3)	1.357(4)
C(2)-H(2)	0.9300
C(3)-C(4)	1.428(3)
C(3)-H(3)	0.9300
C(4)-C(5)	1.404(4)
C(4)-C(9)	1.419(4)
C(5)-C(6)	1.381(3)
C(5)-Cl(1)	1.745(3)
C(6)-C(7)	1.406(4)
C(6)-H(6)	0.9300
C(7)-C(8)	1.384(3)
C(7)-H(7)	0.9300
C(8)-N(2)	1.391(3)
C(8)-C(9)	1.445(3)
C(9)-N(1)	1.376(3)
C(10)-O(1)	1.233(3)
C(10)-N(2)	1.377(3)
C(10)-C(11)	1.530(4)
C(11)-C(15)	1.545(3)
C(11)-C(12)	1.546(3)
C(11)-C(13)	1.555(4)
C(12)-Pd(1)	2.012(3)
C(12)-H(12A)	0.9700
C(12)-H(12B)	0.9700
C(13)-C(14)	1.522(4)
C(13)-H(13A)	0.9700
C(13)-H(13B)	0.9700
C(14)-H(14A)	0.9600
C(14)-H(14B)	0.9600
C(14)-H(14C)	0.9600
C(15)-C(16)	1.532(4)
C(15)-H(15A)	0.9700
C(15)-H(15B)	0.9700

C(16)-H(16A)	0.9600
C(16)-H(16B)	0.9600
C(16)-H(16C)	0.9600
C(17)-N(3)	1.141(3)
C(17)-C(18)	1.462(3)
C(18)-H(18A)	0.9600
C(18)-H(18B)	0.9600
C(18)-H(18C)	0.9600
N(1)-Pd(1)	2.113(2)
N(2)-Pd(1)	1.963(2)
N(3)-Pd(1)	2.020(2)

N(1)-C(1)-C(2)	123.4(2)
N(1)-C(1)-H(1)	118.3
C(2)-C(1)-H(1)	118.3
C(3)-C(2)-C(1)	119.3(2)
C(3)-C(2)-H(2)	120.4
C(1)-C(2)-H(2)	120.4
C(2)-C(3)-C(4)	120.1(3)
C(2)-C(3)-H(3)	119.9
C(4)-C(3)-H(3)	119.9
C(5)-C(4)-C(9)	118.0(2)
C(5)-C(4)-C(3)	125.0(2)
C(9)-C(4)-C(3)	117.0(2)
C(6)-C(5)-C(4)	121.3(2)
C(6)-C(5)-Cl(1)	119.3(2)
C(4)-C(5)-Cl(1)	119.42(19)
C(5)-C(6)-C(7)	120.2(2)
C(5)-C(6)-H(6)	119.9
C(7)-C(6)-H(6)	119.9
C(8)-C(7)-C(6)	121.8(2)
C(8)-C(7)-H(7)	119.1
C(6)-C(7)-H(7)	119.1
C(7)-C(8)-N(2)	127.9(2)
C(7)-C(8)-C(9)	117.2(2)
N(2)-C(8)-C(9)	114.9(2)
N(1)-C(9)-C(4)	121.8(2)
N(1)-C(9)-C(8)	116.8(2)

C(4)-C(9)-C(8)	121.4(2)
O(1)-C(10)-N(2)	125.3(2)
O(1)-C(10)-C(11)	122.2(2)
N(2)-C(10)-C(11)	112.5(2)
C(10)-C(11)-C(15)	108.4(2)
C(10)-C(11)-C(12)	111.9(2)
C(15)-C(11)-C(12)	111.6(2)
C(10)-C(11)-C(13)	106.48(19)
C(15)-C(11)-C(13)	107.9(2)
C(12)-C(11)-C(13)	110.3(2)
C(11)-C(12)-Pd(1)	110.67(18)
C(11)-C(12)-H(12A)	109.5
Pd(1)-C(12)-H(12A)	109.5
C(11)-C(12)-H(12B)	109.5
Pd(1)-C(12)-H(12B)	109.5
H(12A)-C(12)-H(12B)	108.1
C(14)-C(13)-C(11)	114.4(2)
C(14)-C(13)-H(13A)	108.7
C(11)-C(13)-H(13A)	108.7
C(14)-C(13)-H(13B)	108.7
C(11)-C(13)-H(13B)	108.7
H(13A)-C(13)-H(13B)	107.6
C(13)-C(14)-H(14A)	109.5
C(13)-C(14)-H(14B)	109.5
H(14A)-C(14)-H(14B)	109.5
C(13)-C(14)-H(14C)	109.5
H(14A)-C(14)-H(14C)	109.5
H(14B)-C(14)-H(14C)	109.5
C(16)-C(15)-C(11)	114.8(2)
C(16)-C(15)-H(15A)	108.6
C(11)-C(15)-H(15A)	108.6
C(16)-C(15)-H(15B)	108.6
C(11)-C(15)-H(15B)	108.6
H(15A)-C(15)-H(15B)	107.5
C(15)-C(16)-H(16A)	109.5
C(15)-C(16)-H(16B)	109.5
H(16A)-C(16)-H(16B)	109.5
C(15)-C(16)-H(16C)	109.5

H(16A)-C(16)-H(16C)	109.5
H(16B)-C(16)-H(16C)	109.5
N(3)-C(17)-C(18)	179.9(4)
C(17)-C(18)-H(18A)	109.5
C(17)-C(18)-H(18B)	109.5
H(18A)-C(18)-H(18B)	109.5
C(17)-C(18)-H(18C)	109.5
H(18A)-C(18)-H(18C)	109.5
H(18B)-C(18)-H(18C)	109.5
C(1)-N(1)-C(9)	118.4(2)
C(1)-N(1)-Pd(1)	130.61(19)
C(9)-N(1)-Pd(1)	110.98(15)
C(10)-N(2)-C(8)	124.3(2)
C(10)-N(2)-Pd(1)	119.30(17)
C(8)-N(2)-Pd(1)	116.36(15)
C(17)-N(3)-Pd(1)	173.9(2)
N(2)-Pd(1)-C(12)	83.74(10)
N(2)-Pd(1)-N(3)	179.56(8)
C(12)-Pd(1)-N(3)	96.12(10)
N(2)-Pd(1)-N(1)	81.00(8)
C(12)-Pd(1)-N(1)	164.65(10)
N(3)-Pd(1)-N(1)	99.13(8)

Table S9. Anisotropic displacement parameters (Å^{2}x 10^3) for 1. The anisotropic displacement factor exponent takes the form: $-2\pi^2[h^2a^{*2}U^{11} + ... + 2 h k a^* b^* U^{12}]$

	U^{11}	U^{22}	U^{33}	U^{23}	U^{13}	U^{12}
C(1)	26(1)	21(1)	35(1)	-15(1)	-13(1)	5(1)
C(2)	33(1)	28(1)	34(1)	-21(1)	-13(1)	9(1)
C(3)	25(1)	32(1)	29(1)	-19(1)	-11(1)	9(1)
C(4)	20(1)	25(1)	30(1)	-14(1)	-11(1)	7(1)
C(5)	19(1)	30(1)	25(1)	-12(1)	-6(1)	2(1)
C(6)	25(1)	25(1)	29(1)	-12(1)	-7(1)	1(1)
C(7)	26(1)	22(1)	28(1)	-12(1)	-7(1)	2(1)
C(8)	23(1)	19(1)	26(1)	-13(1)	-9(1)	5(1)
C(9)	20(1)	21(1)	25(1)	-12(1)	-10(1)	6(1)
C(10)	25(1)	22(1)	26(1)	-13(1)	-8(1)	3(1)
C(11)	27(1)	21(1)	26(1)	-11(1)	-6(1)	2(1)
C(12)	34(1)	23(1)	27(1)	-9(1)	-4(1)	4(1)
C(13)	27(1)	23(1)	29(1)	-11(1)	-5(1)	1(1)
C(14)	31(1)	30(1)	36(1)	-14(1)	-12(1)	2(1)
C(15)	30(1)	31(1)	24(1)	-12(1)	-6(1)	1(1)
C(16)	35(2)	47(2)	36(2)	-16(1)	-14(1)	4(1)
C(17)	24(1)	21(1)	31(1)	-10(1)	-10(1)	5(1)
C(18)	31(1)	20(1)	52(2)	-13(1)	-15(1)	3(1)
N(1)	24(1)	19(1)	31(1)	-13(1)	-11(1)	5(1)
N(2)	25(1)	17(1)	25(1)	-10(1)	-4(1)	2(1)
N(3)	24(1)	20(1)	30(1)	-11(1)	-10(1)	5(1)
O(1)	38(1)	22(1)	31(1)	-16(1)	-2(1)	-4(1)
Pd(1)	22(1)	16(1)	27(1)	-10(1)	-8(1)	3(1)
Cl(1)	29(1)	36(1)	26(1)	-11(1)	-4(1)	4(1)

Table S10. Hydrogen coordinates (x 104) and isotropic displacement parameters (Å2x 103) for 1.

	x	y	z	U(eq)
H(1)	7732	3986	4487	30
H(2)	9466	4378	2455	34
H(3)	10724	6322	1393	32
H(6)	10675	9998	2537	31
H(7)	8998	9452	4610	29
H(12A)	3990	5637	8373	34
H(12B)	5872	5378	8829	34
H(13A)	2557	7543	8525	31
H(13B)	3516	8838	7810	31
H(14A)	3597	8620	5849	46
H(14B)	1606	8317	6610	46
H(14C)	2996	7210	6479	46
H(15A)	5789	8395	9036	33
H(15B)	5027	7069	9902	33
H(16A)	7931	6123	9473	57
H(16B)	8034	7158	10109	57
H(16C)	8689	7460	8627	57
H(18A)	3618	1364	7294	49
H(18B)	4511	979	8517	49
H(18C)	5685	956	7201	49

Table S11: Torsion angles [°] for 1

N(1)-C(1)-C(2)-C(3)	-0.6(4)
C(1)-C(2)-C(3)-C(4)	0.1(4)
C(2)-C(3)-C(4)-C(5)	-179.7(2)
C(2)-C(3)-C(4)-C(9)	1.0(3)
C(9)-C(4)-C(5)-C(6)	2.1(3)
C(3)-C(4)-C(5)-C(6)	-177.2(2)
C(9)-C(4)-C(5)-Cl(1)	-178.37(17)
C(3)-C(4)-C(5)-Cl(1)	2.4(3)
C(4)-C(5)-C(6)-C(7)	-1.4(4)
Cl(1)-C(5)-C(6)-C(7)	179.02(19)
C(5)-C(6)-C(7)-C(8)	-0.6(4)
C(6)-C(7)-C(8)-N(2)	-177.0(2)
C(6)-C(7)-C(8)-C(9)	1.8(4)
C(5)-C(4)-C(9)-N(1)	179.0(2)
C(3)-C(4)-C(9)-N(1)	-1.7(3)
C(5)-C(4)-C(9)-C(8)	-0.8(3)
C(3)-C(4)-C(9)-C(8)	178.5(2)
C(7)-C(8)-C(9)-N(1)	179.1(2)
N(2)-C(8)-C(9)-N(1)	-1.9(3)
C(7)-C(8)-C(9)-C(4)	-1.1(3)
N(2)-C(8)-C(9)-C(4)	177.9(2)
O(1)-C(10)-C(11)-C(15)	-46.9(3)
N(2)-C(10)-C(11)-C(15)	135.4(2)
O(1)-C(10)-C(11)-C(12)	-170.4(2)
N(2)-C(10)-C(11)-C(12)	11.9(3)
O(1)-C(10)-C(11)-C(13)	69.0(3)
N(2)-C(10)-C(11)-C(13)	-108.7(2)
C(10)-C(11)-C(12)-Pd(1)	-15.1(3)
C(15)-C(11)-C(12)-Pd(1)	-136.78(19)
C(13)-C(11)-C(12)-Pd(1)	103.3(2)
C(10)-C(11)-C(13)-C(14)	55.5(3)
C(15)-C(11)-C(13)-C(14)	171.8(2)
C(12)-C(11)-C(13)-C(14)	-66.1(3)
C(10)-C(11)-C(15)-C(16)	-58.6(3)
C(12)-C(11)-C(15)-C(16)	65.1(3)
C(13)-C(11)-C(15)-C(16)	-173.5(2)

C(2)-C(1)-N(1)-C(9)	-0.1(4)
C(2)-C(1)-N(1)-Pd(1)	179.16(18)
C(4)-C(9)-N(1)-C(1)	1.3(3)
C(8)-C(9)-N(1)-C(1)	-178.9(2)
C(4)-C(9)-N(1)-Pd(1)	-178.10(17)
C(8)-C(9)-N(1)-Pd(1)	1.7(2)
O(1)-C(10)-N(2)-C(8)	-3.7(4)
C(11)-C(10)-N(2)-C(8)	173.9(2)
O(1)-C(10)-N(2)-Pd(1)	179.3(2)
C(11)-C(10)-N(2)-Pd(1)	-3.1(3)
C(7)-C(8)-N(2)-C(10)	2.9(4)
C(9)-C(8)-N(2)-C(10)	-175.9(2)
C(7)-C(8)-N(2)-Pd(1)	180.0(2)
C(9)-C(8)-N(2)-Pd(1)	1.2(3)
